# Gold(I)-Catalyzed Reactivity of Furan-ynes with *N*-Oxides: Synthesis of Substituted Dihydropyridinones and Pyranones

**DOI:** 10.1021/acs.joc.1c00746

**Published:** 2021-06-08

**Authors:** Stefano Nejrotti, Francesco Marra, Emanuele Priola, Andrea Maranzana, Cristina Prandi

**Affiliations:** Dipartimento di Chimica, Università degli Studi di Torino, via Pietro Giuria 7, I-10125 Torino, Italy

## Abstract

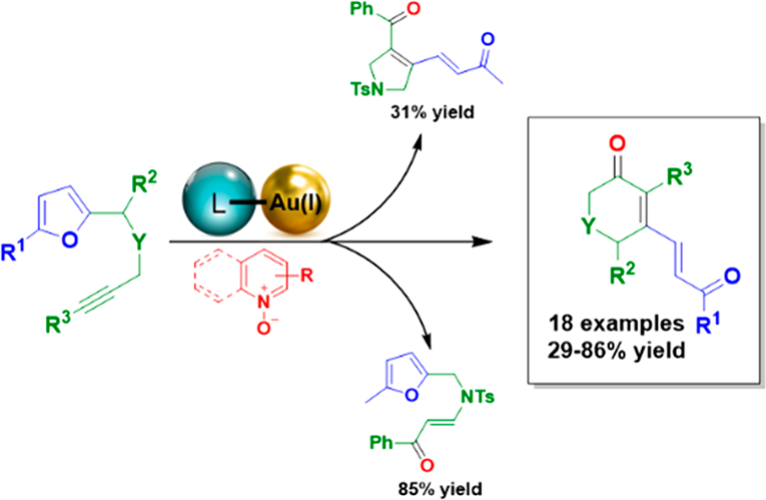

The reactivity of “furan-ynes” in combination with pyridine and quinoline *N*-oxides in the presence of a Au(I) catalyst, has been studied, enabling the synthesis of three different heterocyclic scaffolds. Selective access to two out of the three possible products, a dihydropyridinone and a furan enone, has been achieved through the fine-tuning of the reaction conditions. The reactions proceed smoothly at room temperature and open-air, and were further extended to a broad substrate scope, thus affording functionalized dihydropyridinones and pyranones.

## Introduction

**Scheme 1:**
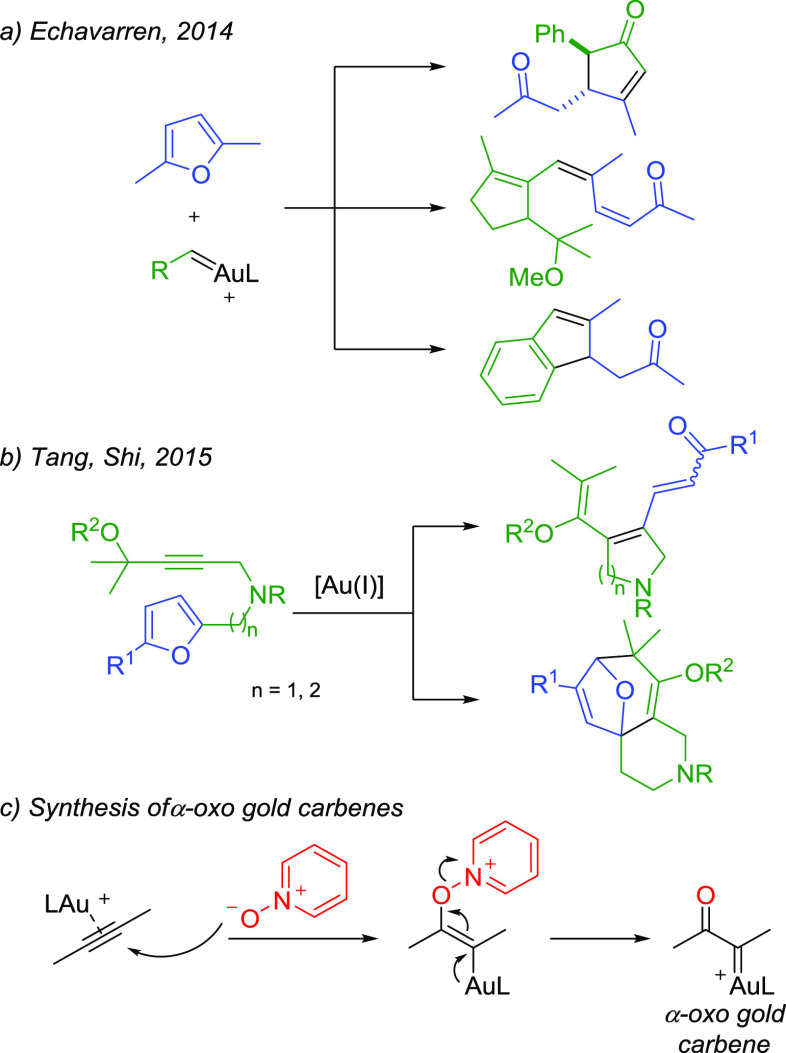
Background of the Work

The activation of alkynes toward the attack of a nucleophile by means of gold catalysis is a well-established tool in modern organic synthesis.^1^ Since the beginning of this century, the furan ring has emerged as a versatile candidate for both Au(I)- and Au(III)-catalyzed transformations, by virtue of its nucleophilicity.^2^ A successful example in this regard is represented by the synthesis of phenols from furan-tethered alkynes (“furan-ynes”), which has been widely explored throughout the years, mainly by Hashmi and his group, enabling the synthesis of a variety of molecular scaffolds.^3^ More recently, the combination of furans with Au(I) carbene chemistry has disclosed intriguing synthetic possibilities. In 2014, Echavarren reported that three different types of Au(I) carbenes, generated respectively by rearrangement of propargyl esters, cycloisomerization of 1,6-enynes, and *retro*-Buchner reaction, underwent intermolecular reaction with furans through related mechanistic pathways, thus giving access to an array of diverse molecular frames (Scheme 1a).^4^ Gold(I) carbenes obtained from propargyl esters were later employed by Tang and Shi in intramolecular reactions with furans, achieving the synthesis of functionalized *N*-heterocycles and *O*-bridged tricyclic scaffolds (Scheme 1b).^5^ Another relevant class of Au(I) carbenes is represented by α-oxo gold(I) carbenes. These reactive intermediates can be accessed by treatment of an alkyne with either a pyridine or quinoline *N*-oxide in the presence of a Au(I) complex, through nucleophilic attack of the O atom of the *N*-oxide onto the gold-activated triple bond and subsequent cleavage of the pyridine or quinoline (Scheme 1c).^6^

α-Oxo gold(I) carbenes can undergo several reaction pathways, including cyclopropanations, migrations, ring expansions, insertions, and reactions with nucleophiles.^7^

On these premises, following our interest for the synthetic manipulation of heterocycles through gold catalysis,^8^ we decided to investigate the reactivity of furan-ynes with *N*-oxides, in the presence of a gold catalyst.

## Results and Discussion

To start the investigation, furan-yne **1a**, provided with a phenyl terminus at the alkyne moiety,^9^ was reacted with the Au(I) complex [(IPr)Au(NTf_2_)] and 2,6-dichlopyridine *N*-oxide **A**, at room temperature in 1,2-dichloroethane (Table 1, entry 1). Under these conditions, the complete consumption of the starting material and the formation of the 6-membered dihydropyridinone **2a** in 81% yield were observed after 6 h. The structure of **2a** was unequivocally identified through X-ray diffraction.^10^ On the other hand, when [((2,4-^*t*^Bu_2_C_6_H_3_O)_3_P)AuCl] was used as the Au(I) complex, in combination with AgNTf_2_, the formation of two other products was observed: the 5-membered dihydropyrrole **3a** and the furan enone **4a**, whose structures were again confirmed by XRD.^10^ However, in this case the yields of all three products were quite low (entry 2).

**Table 1:**
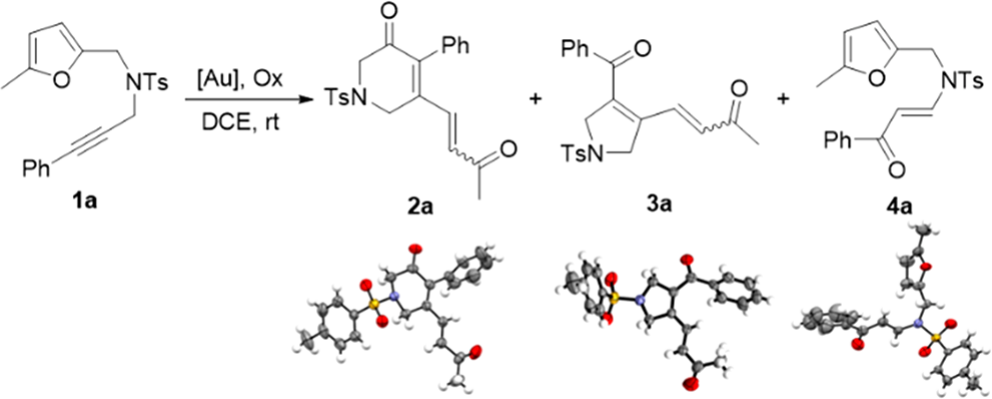
Optimization of the Reaction Conditions^a^

				yield %^b^		
entry	[Au]	Ox	time	**2a** (*E*/*Z*)	**3a** (*E*/*Z*)	**4a**
1	[(IPr)Au(NTf_2_)]	**A**	6 h	81 (12/88)	–	–
2	[((2,4-^*t*^Bu_2_C_6_H_3_O)_3_P)AuCl]/AgNTf_2_	**A**	6 h	15 (0/100)	6 (0/100)	11
3	[((*p*-CF_3_Ph)_3_P)AuCl]/AgNTf_2_	**A**	6 h	23 (0/100)	6 (0/100)	8
4	[(JohnPhos)AuCl]/AgNTf_2_	**A**	6 h	77 (6/94)	–	–
5	[(JohnPhos)Au(NCMe)]SbF_6_	**A**	6 h	40 (25/75)	–	–
6	[(^*t*^BuXPhos)AuCl]/AgNTf_2_	**A**	6 h	67 (10/90)	–	–
7	[(IPr)Au(NTf_2_)]	**B**	6 h	–	31 (0/100)	60
8	[(IPr)Au(NTf_2_)]	**C**	6 h	48 (10/90)	–	–
9	[(IPr)Au(NTf_2_)]	**D**	6 h	56 (14/86)	17 (0/100)	27
10	[(IPr)Au(NTf_2_)]	**E**	6 h	–	–	22
11	[(IPr)Au(NTf_2_)]	**F**	6 h	68 (1/99)	–	–
12	[(IPr)Au(NTf_2_)]	**G**	6 h	–	–	–
13	[(IPr)Au(NTf_2_)]	**F**	20 h	96 (2/98)	–	–
14	[(MorDalPhos)Au(NCMe)]SbF_6_	**B**	20 h	–	4 (0/100)	85^c^
15	[((2,4-^*t*^Bu_2_C_6_H_3_O)_3_P)AuCl]/AgNTf_2_	**B**	20 h	–	13 (0/100)	29
16	[(JohnPhos)AuCl]/AgNTf_2_	**B**	20 h	–	15 (0/100)	60
17	[((C_6_F_5_)_3_P)AuCl]/AgNTf_2_	**B**	20 h	–	4 (0/100)	11

a Conditions: 0.1 mmol of **1a**, 0.12 mmol of Ox, 0.005 mmol of Au(I) complex, and, when specified, 0.005 mmol of Ag salt, in 1.0 mL of DCE.

b Determined by ^1^H NMR with *n*-heptane as internal standard.

c 74% yield of isolated product.
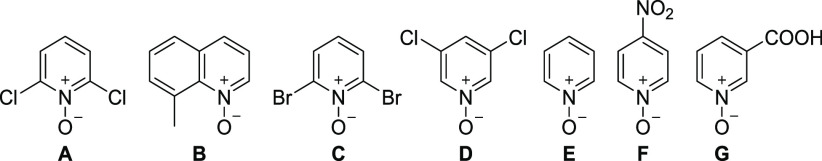

With the aim of optimizing and driving the selectivity of the reaction, several conditions and gold complexes were tested (entries 3–6; see Table S1 in the Supporting Information (SI) for the complete list of experiments), but none of them outdid [(IPr)Au(NTf_2_)] in terms of the yield of **2a**. It should also be noted that the reaction proceeded with good stereoselectivity for the configuration of the exocyclic C–C double bond in **2a** and **3a**, observing a general preference for the *Z* isomer. With the best performing Au(I) complex, [(IPr)Au(NTf_2_)], we shifted our attention to a screening of *N*-oxides (entries 7–12), and we found that the selectivity of the reaction was sensitively affected by the choice of this reagent. With 8-methylquinoline *N*-oxide **B**, the formation of **2a** was completely suppressed in favor of a 1/2 ratio of **3a** and **4a** (entry 7). Other *N*-oxides were either less selective (**D**, entry 9) or less active (entries 8, 10–12). However, 4-nitropyridine *N*-oxide **F** afforded **2a** with a remarkably high *Z* selectivity (entry 11). Eventually, we identified the most suitable conditions for the synthesis of **2a** with oxidant **F**, by extending the reaction time up to 20 h (entry 13).

We then considered the outcome of the reaction with **B** and [(IPr)Au(NTf_2_)] (entry 7), and we deemed it worthwhile to further explore the reactivity of this oxidant with different Au(I) complexes. With [(MorDalPhos)Au(NCMe)]SbF_6_ the selective synthesis of the furan enone **4a** was achieved (entry 14). Unfortunately, we were not able to increase the yield of **3a** beyond 31%, which remained as the best result for this product (entry 7). With the conditions in entry 13 (Table 1), product **2a** was isolated in good yields, however we found that the *Z* isomer slowly isomerized into (*E*)-**2a** over time. On this basis, we decided to direct the reaction toward complete *E* selectivity, by a further optimization of the conditions described in entry 13. As shown in Table 2, the first parameter we investigated was the temperature, which was raised to 80 °C (Table 2, entry 1). This resulted indeed in an improvement of the *E*/*Z* ratio, but with a decrease in the overall yield. A different approach, based on the addition of a Brønsted acid as promoter of the isomerization, afforded (*E*)-**2a** with complete selectivity, but the yields were again not satisfactory (*p*-toluenesulfonic acid, *p*-TsOH, and methanesulfonic acid, MsOH, entries 3 and 4). Also attempts to perform the reaction in more acidic solvents, such as chloroform and hexafuoroisopropanol (entries 5 and 6), were met with failure. The decrease in the yield may be attributed to the possible degradation of the starting material **1a** in the presence of a Brønsted acid. On this basis, we decided to add the acid only at the end of the Au(I)-catalyzed step, *i.e*. after 20 h (entries 7–9): under these conditions, with MsOH a remarkable 80% NMR yield of (*E*)-**2a** (72% after isolation) was obtained by adjusting the amount of MsOH to 5.0 equiv (entry 9; see Table S2 in the SI for the complete list of experiments).

**Table 2:**
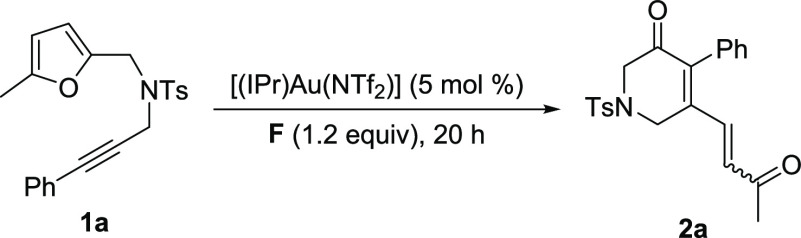
Optimization of the *E*/*Z* Ratio in the Synthesis of **2a**^a^

entry	*T* (°C)	solvent	additive	yield %^b^ (*E/Z*)
1	80 °C	DCE	–	66 (23/77)
2	rt	DCE	TFA (2.0 equiv)	73 (16/84)
3	rt	DCE	*p*-TsOH (2.0 equiv)	43 (100/0)
4	rt	DCE	MsOH (2.0 equiv)	58 (100/0)
5	rt	CHCl_3_	–	63 (3/97)
6	rt	HFIP	–	–
7	rt	DCE	*p*-TsOH (2.0 equiv)^c^	89 (11/89)
8	rt	DCE	MsOH (2.0 equiv)^c^	94 (17/83)
9	rt	DCE	MsOH (5.0 equiv)^c^	80^d^ (100/0)

a TFA = trifluoroacetic acid; HFIP = hexafluoroisopropanol.

b Determined by ^1^H NMR with *n*-heptane as internal standard.

c Added after 20 h, then stirred for one additional hour.

d 72% yield of isolated product.

**Scheme 2:**
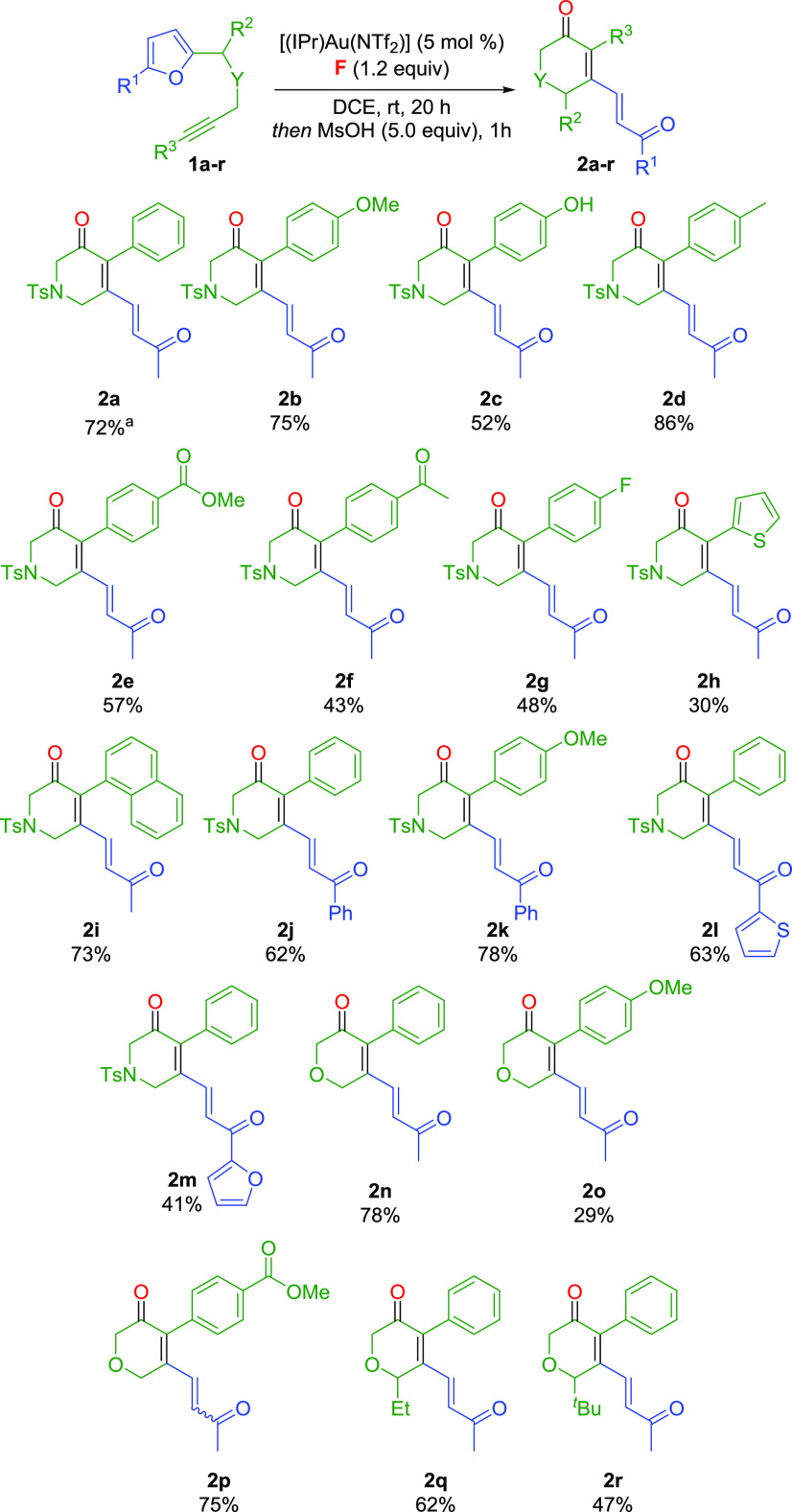
Substrate Scope for the Synthesis of Dihydropyridinones and Pyranones Yield unchanged at 1.3 mmol scale. Reactions performed at a 0.2 mmol scale. Yields of isolated products. *E*/*Z* ratio 100/0 in all cases, except for **2p** (*E*/*Z* 40/60).

Having identified the most suitable conditions to orient the divergent reactivity of **1a** toward the exclusive formation of dihydropyridinone **2a** as the *E* isomer, we investigated the possible extension of the reaction substrate scope. Furan-ynes **1b**–**r** were synthesized and reacted with 4-nitropyridine *N*-oxide **F** and [(IPr)Au(NTf_2_)] for 20 h, after which 5.0 equiv of MsOH were added, and the mixture was stirred for one additional hour (Scheme 2). Variations on the terminal aromatic ring were introduced, proving that a variety of electron-donating (**2b**–**d**) and electron-withdrawing (**2e**–**g**) functional groups are well tolerated. Apart from substituted phenyl rings, thiophene and naphthalene were also suitable (**2h**–**i**). Conversely, in the presence of an alkyl chain (R^3^ = *n*-Bu) the reaction did not occur, and degradation of the starting material was observed. The methyl group on the furan ring in the starting material was replaced with phenyl, thienyl, and furyl groups, obtaining aryl ketones **2j**–**m** as product. Unfortunately, with R^1^ = H (**1s**; see the SI), the reaction was troublesome and hardly reproducible. On the other hand, replacing the *N*-Ts linker with an *O* linker resulted in the synthesis of pyranones **2n**–**p**. Substituents on the heterocyclic ring were also successfully introduced, affording pyranones **2q**–**r**. In all cases, apart from product **2p**, complete *E* selectivity was observed, and the yields ranged from moderate to high.

**Scheme 3:**
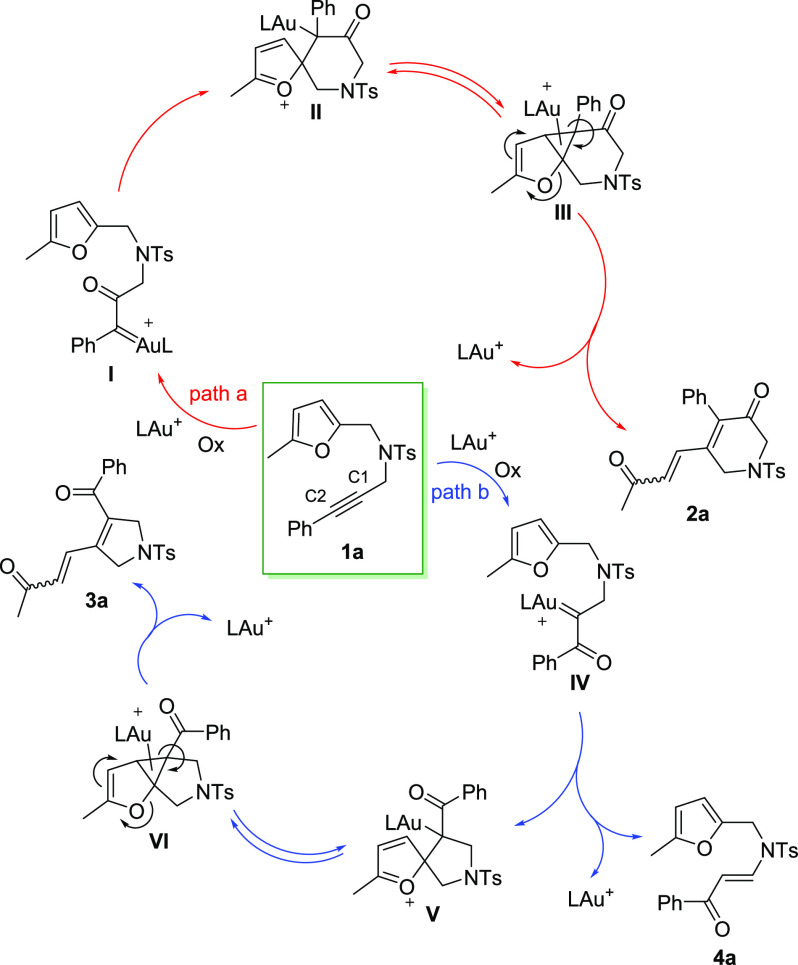
Mechanistic Pathways for **2a**, **3a**, and **4a**

A plausible mechanistic picture for the formation of products **2a**, **3a**, and **4a** from furan-yne **1a** is depicted in Scheme 3.^5,6,11^ Upon activation of the alkyne moiety in substrate **1a** by the Au(I) complex, the attack of the *N*-oxide onto the C1 atom of the triple bond leads to α-oxo gold(I) carbene **I** (see Scheme 1c), which then undergoes cyclization through nucleophilic attack by the furan ring, affording the spirocyclic intermediate **II** (path a). A cyclopropanation/cyclopropane opening sequence eventually determines the opening of the furan ring, leading to the formation of **2a**. Intermediate **II** might also convert directly to **2a** through an elimination step. Based on the results presented in Tables 1 and 2, the *Z* isomer is likely first formed and then converted into (*E*)-**2a**.^12^ If the regioselectivity of the *N*-oxide attack is switched, the spirocyclic intermediate **V** is formed upon attack of the furan onto Au(I) carbene **IV** (path b). Then, the final product **3a** is obtained through a mechanism analogous to the one described for **2a**. Alternatively, the α-oxo gold(I) carbene intermediate **IV** may also undergo a 1,2-H shift with the neighboring CH_2_ group, leading to the formation of furan enone **4a**.^8c,13^

It should be pointed out that the best selectivity for **4a** was obtained with the gold(I) complex [(MorDalPhos)Au(NCMe)]SbF_6_ (Table 1, entry 14). The MorDalPhos ligand has been reported to temper the electrophilicity of the Au(I) carbene through bidentate coordination from both the P and the N atom.^14^ This would be consistent what was observed in our case, as the less electrophilic carbene is likely less prone to get attacked by the nucleophilic furan ring, and thus more available to selectively undergo a 1,2-H shift. However, when more electrophilic Au(I) complexes were employed, the expected enhancement in the selectivity toward **3a** was not observed (Table 1, entries 15 and 17).

As highlighted by the study on the reaction conditions (Table 1), the C1 vs C2 regioselectivity of the nucleophilic attack is highly dependent on the nature of the *N*-oxide reagent, while the electronic effects of the alkyne substituent R^3^ are not relevant in modifying this selectivity (Scheme 2). To better elucidate the influence of the electronic and steric factors, we studied the attack of *N*-oxides **F** and **B** onto the [LAu]^+^-**1a** π-complex through DFT calculation (Table 3). The Natural Bond Analysis of this intermediate revealed that the charge density values on C1 and C2 are very small, being slightly negative on C1 (−0.10) and slightly positive on C2 (+0.04). On the other hand, the partial negative charge on the O atom in the *N*-oxide is larger in **B** (−0.55) than in **F** (−0.49). By considering the charge density only, **B** would be expected to be more reactive than **F** and C2 would be the preferential site of reaction. Thus, a more detailed study on the transition states for the attack of **B** and **F** onto C1 and C2 was carried out, and the results are reported in Table 3. The addition of **B** to C1 is preferred to the addition to C2: the free energy barriers (Δ*G*^‡^) are 20.2 and 25.5 kcal mol^–1^, respectively. On the other hand, **F** displays opposite behavior, with a smaller barrier for the addition to C1 (26.3 kcal mol^–1^). **B** appears to be more reactive than **F**. To explain the regioselectivity, Shubin Liu’s energy decomposition analysis (EDA-SBL) was carried out. The EDA-SBL method splits the total energy (*E*_tot_) in three components: *E*_e_ (sum of nuclear–nuclear, electron–electron, and nuclear-electron electrostatic interactions), *E*_q_ (quantum interaction: sum of exchange-correlation and Pauli energies, associated to the repulsion between filled orbitals), and *E*_s_ (steric energy repulsion). The larger values of the three components indicate the main effects responsible for the barriers. In all the four reactions, the barriers are dominated by change of steric (Δ*E*^‡^_s_) and quantum (Δ*E*^‡^_q_) effects. The quantum effect contributes positively to the height of the barrier, and the steric effect, negatively. Electrostatic effects (Δ*E*^‡^_e_) are relevant but less important. By comparing the Δ*E*^‡^_s_ for C1 and C2, the addition to the former displays less negative values. The difference Δ*E*^‡^_s_(C2)-Δ*E*^‡^_s_(C1) is 64.4 and 75.4 for **B** and **F** respectively, which could indicate a larger steric effect when addition occurs to C2. The regioselectivity is supposed to depend on a delicate balance between density of charge on the oxidant, responsible for the positive contribution to the barrier, and steric effect. By comparison, *N*-oxide **D** shows a charge density on the O atom of −0.52, intermediate between **B** and **F**, and its attack on both C1 and C2 (Table 1, entry 9). Noncovalent interaction plots (Figure S5 in the SI) visualize noncovalent interactions from the topological analysis of the electron density. These highlight the presence of van der Waals attractive interactions between the phenyl group and the pyridine or quinoline rings, while strong steric repulsion between the *N*-oxide and the reactant are lacking in the transition structures.

**Table 3:**
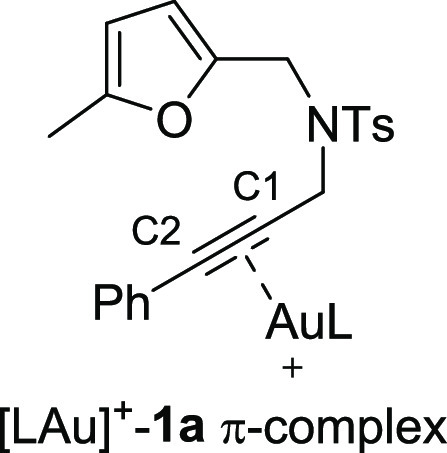
Free Energy Barriers and Energy Decomposition Analysis,^a^ in kcal mol^–1^, for the Addition of **B** and **F** to C1 and C2

Ox	carbon	Δ*G*^‡^	Δ*E*^‡^_s_	Δ*E*^‡^_e_	Δ*E*^‡^_q_	Δ*E*^‡^_tot_
**B**	C1	25.5	–552.4	104.2	444.0	–4.2
**B**	C2	20.2	–487.8	99.0	382.7	–6.8
**F**	C1	26.3	–448.6	104.8	346.2	2.3
**F**	C2	26.9	–373.2	84.2	292.5	3.8

a Calculated at M06/def2-SVP level. L = IPr.

**Scheme 4:**
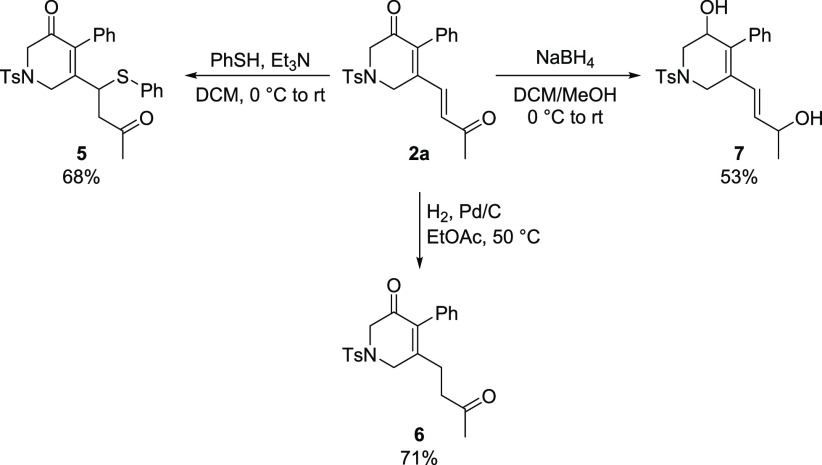
Synthetic Manipulations of **2a**

To provide a glimpse of the synthetic value of the products of our Au(I)-catalyzed methodology, we subjected **2a** to the synthetic manipulations reported in Scheme 4. Remarkably, the Michael addition of thiophenol, catalyzed by Et_3_N,^15^ proceeded smoothly at room temperature with complete selectivity for the exocyclic double bond over the endocyclic one, thus obtaining sulfide **5**. Similarly to the Michael addition, also the hydrogenation over Pd/C was selective for the exocyclic double bond, affording the partially saturated compound **6**. On the other hand, reduction with NaBH_4_ did not discriminate between the two carbonyl groups, and diol **7** was obtained as the product.

## Conclusion

In conclusion, the study of the reactivity of furan-ynes with *N*-oxides, in the presence of a Au(I) catalyst, has disclosed an intriguing divergent mechanistic picture, with the formation of three possible densely functionalized heterocyclic scaffolds. This work contributes to extend the wide landscape of synthetic opportunities offered by the powerful combination of gold catalysis and furans, enabling the straightforward synthesis of dihydropyridinones and pyranones through control over the product selectivity and the diastereoselectivity of the reaction. Moreover, the good tolerance of structural variations in the substrate, as well as of the presence of either nucleophilic or electrophilic functional groups, allows for a tailored application of the methodology, in view of its exploitation in synthesis. The theoretical investigation on the regioselectivity contributes to the understanding of the role of electronic and steric effects in the mechanism of this class of reactions, and could potentially prompt a systematic more-in-detail analysis of previously reported similar methodologies.

## Experimental Section

### Computational Method

The stationary points were optimized in the gas phase with the DFT M06 functional,^16^ with the ONIOM procedure.^17^ In this procedure, the molecule is divided into two layers treated with different levels of calculations. This approach has been found to be useful for modeling large molecular systems. In this work, the higher layer was treated at the M06/def2-SVP^18^ level, whereas for the lower layer the chosen level was M06/STO-3G^19^ (for Au only, effective core potential LanL2DZ was used). This procedure was labeled as ONIOM(M06/def2-SVP:M06/STO-3G). The figures showing the layers assignment of the atoms are reported in the Supporting Information (Figure S4). The energies were refined by single-point energy calculations at level ONIOM(M06/def2-TZVP:M06/STO-3G) including the solvent effect (dichloroethane) by the Solvation Model based on Density (SMD).^20^ The relative Gibbs free energies in solution (Δ*G*) were estimated at *T* = 298 K. Multiwfn software^21^ was used for the noncovalent interaction analysis (NCI)^22^ and for Shubin Liu’s energy decomposition analysis (EDA-SBL).^23^ Geometry optimizations and thermochemistry calculations were carried out by using the Gaussian 16 programs.

### General Information

Flasks and all equipment used for the generation and reaction of moisture-sensitive compounds were dried by electric heat gun under nitrogen. Reaction heating was provided through an oil bath. Reagents and solvents were purchased from Merck, Acros Organics, TCI or Alfa Aesar. Anhydrous THF was obtained by distillation over LiAlH_4_, followed by distillation over Na-benzophenone; Et_3_N was distilled over CaH_2_. All other reagents were used as received, without further purification. Flash column chromatography was performed over silica gel (40–63 μm, 230–400 mesh); *R*_*f*_ values refer to TLC carried out on silica gel plates. ^1^H NMR and ^13^C NMR spectra were recorded on a Jeol ECZR600, in CDCl_3_ or in CD_3_OD, using the residual solvent peak as an internal reference (CHCl_3_, ^1^H: 7.26 ppm; CDCl_3_, ^13^C: 77.16 ppm; CH_3_OH, ^1^H: 3.34 ppm; CD_3_OD, ^13^C: 49.86 ppm). Multiplicity is reported as follows: s (singlet), d (doublet), t (triplet), q (quartet), m (multiplet), br (broad). GC-MS spectra were recorded at an ionizing voltage of 70 eV. HRMS analysis were run on a high resolving power hybrid mass spectrometer (HRMS) Orbitrap Fusion (Thermo Scientific, Rodano, Italy), equipped with an a ESI ion source. The samples were analyzed in acetonitrile solution using a syringe pump at a flow rate of 5 μL/min. The tuning parameters adopted for the ESI source were as follows: source voltage 5.0 kV, source current 0.5 μA, capillary voltage 32 V, tube lens voltage 75 V. The heated capillary temperature was maintained at 275 °C. Crystals of **2a**, **3a**, and **4a** were obtained by slow evaporation from a MeCN solution at 4–6 °C. Reaction schemes for the synthesis of the substrates, as well as details about XRD analysis, are available in the Supporting Information.

### Synthesis of Furan-2-ylboronic Acid, int-1

This is a modified literature procedure.^24^ A solution of furan (1.0 equiv, 20 mmol) in anhydrous THF (1.0 M), under a N_2_ atmosphere, was cooled down to −10 °C. *n*-BuLi (1.0 equiv, 2.5 M solution in hexane) was added, and the mixture was allowed to warm to 0 °C and stirred for 1 h. Then, triisopropylborate (2.0 equiv) was added, and the mixture was allowed to warm to room temperature and stirred for 30 min, before addition of 50 mL of a 3 M HCl solution. The mixture was extracted three times with Et_2_O, and the combined organic layers were extracted three times with a 1 M NaOH solution. The combined NaOH acqueous layers were brought to acidic pH with a 6 M HCl solution and extracted three times with Et_2_O; the combined organic layers were dried over anhydrous Na_2_SO_4_ and filtered, and the volatiles wee evaporated under reduced pressure to afford furan-2-ylboronic acid as a yellow solid, which was used for the next step without further purification (1.320 g, 59% yield). ^1^H NMR (600 MHz, dmso-d6) δ (ppm): 8.21 (br s, 2H), 7.84 (d, 1H, *J* = 1.7 Hz), 7.09 (d, 1H, *J* = 3.3 Hz), 6.50 (dd, 1H, *J* = 3.3, 1.7 Hz).

### General Procedure for the Suzuki Coupling of 5-Bromo-2-furaldehyde (GP1)^25^

Into a flask, 2-bromofuradelhyde (1.0 equiv, 15 mmol), arylboronic acid (1.1 equiv), palladium(II) acetate (0.02 equiv), potassium carbonate (2.5 equiv), and tetrabutylammonium bromide (1.0 equiv) were added, then deionized H_2_O (0.5 M with respect to 2-bromofuraldehyde) was added, and the mixture was vigorously stirred for 2 h at room temperature. Then, the mixture was extracted three times with EtOAc; the combined organic layers were dried over anhydrous Na_2_SO_4_ and filtered, and the volatiles were evaporated under reduced pressure. The crude product was purified by flash column chromatography to obtain the pure 5-aryl substituted furfural.

### 5-Phenylfuran-2-carbaldehyde,^26^ int-2

Synthesized according to the general procedure GP1. Yellow oil, 1.93 g, 75% yield. *R*_*f*_ 0.46 (9/1 PE/EtOAc). ^1^H NMR (600 MHz, CDCl_3_) δ (ppm): 9.66 (s, 1H), 7.85–7.82 (m, 2H), 7.47–7.44 (m, 2H), 7.42–7.39 (m, 1H), 7.33 (d, 1H, *J* = 3.7 Hz), 6.85 (d, 1H, *J* = 3.7 Hz). GC-MS *m*/*z* (%): 172 [M]^+^ (100), 171 (45), 115 (77).

### 5-(Thiophen-2-yl)furan-2-carbaldehyde,^27^ int-3

Synthesized according to the general procedure GP1. Yellow oil, 2.54 g, 95% yield. *R*_*f*_ 0.46 (9/1 PE/EtOAc). ^1^H NMR (600 MHz, CDCl_3_) δ (ppm): 9.62 (s, 1H), 7.53 (dd, 1H, *J* = 3.7, 1.2 Hz), 7.41 (dd, 1H, *J* = 5.0, 1.2 Hz), 7.29 (d, 1H, *J* = 3.8 Hz), 7.11 (dd, 1H, *J* = 5.0, 3.7 Hz), 6.68 (d, 1H, *J* = 3.7 Hz). GC-MS *m*/*z* (%): 178 [M]^+^ (100), 121 (72).

### [2,2′-Bifuran]-5-carbaldehyde,^28^ int-4

Synthesized according to the general procedure GP1. Yellow oil, 2.06 g, 85% yield. *R*_*f*_ 0.36 (9/1 PE/EtOAc). ^1^H NMR (600 MHz, CDCl_3_) δ (ppm): 9.62 (s, 1H), 7.52 (dd, 1H, *J* = 1.8, 0.8 Hz), 7.30 (d, 1H, *J* = 3.7 Hz), 6.91 (dd, 1H, *J* = 3.4, 0.8 Hz), 6.73 (d, 1H, *J* = 3.7 Hz), 6.53 (dd, 1H, *J* = 3.5, 1.8 Hz). GC-MS *m*/*z* (%): 162 [M]^+^ (100), 105 (65).

### General Procedure for the Synthesis of Terminal Alkynes with *N*-Ts Linker (GP2)^29^

To a 1.0 M solution of furaldehyde (1.0 equiv, 10–20 mmol) in DCM, MgSO_4_ (1.1 equiv) and propargylamine (1.0 equiv) were added, and the mixture was stirred at room temperature for 24–48 h (GC-MS monitoring). The mixture was filtered over silica with EtOAc, and the volatiles were evaporated under reduced pressure to obtain the crude imine, which was used for the next step without further purification.

To a 1.0 M solution of the crude furanyl imine in MeOH, NaBH_4_ (1.0 equiv) was added portionwise at 0 °C. The mixture was allowed to reach room temperature and stirred for 5–10 min (GC-MS monitoring) until complete conversion. Then, MeOH was evaporated to half of the initial volume under reduced pressure. Water was added, and the mixture was extracted three times with EtOAc; the combined organic layers were dried over Na_2_SO_4_ and filtered, and the volatiles were evaporated under reduced pressure to obtain the crude amine, which was used for the next step without further purification.

To a 1.0 M solution of the crude amine in DCM, Et_3_N (1.0 equiv) was added, then *p*-toluenesulfonyl chloride (1.0 equiv) was added portionwise, and the mixture was stirred at room temperature overnight. Then, water was added, and the mixture was extracted three times with DCM; the combined organic layers were dried over Na_2_SO_4_ and filtered, and the volatiles were evaporated under reduced pressure. The crude product was purified by flash column chromatography to obtain the pure *N*-Ts product.

### *N*-(Furan-2-ylmethyl)-4-methyl-*N*-(prop-2-yn-1-yl)benzenesulfonamide,^30^ int-5

Synthesized according to the general procedure GP2. White solid, 1.46 g, 51% yield (3 steps). *R*_*f*_ 0.47 (80/15/5 PE/DCM/EtOAc). ^1^H NMR (600 MHz, CDCl_3_) δ (ppm): 7.75–7.73 (m, 2H), 7.35 (dd, 1H, *J* = 1.8, 0.9 Hz), 7.31–7.28 (m, 2H), 6.31 (dd, 1H, *J* = 3.3, 1.8 Hz) superimposed to 6.30 (dd, 1H, *J* = 6.3, 0.8 Hz), 4.43 (s, 2H), 4.02 (d, 2H, *J* = 2.5 Hz), 2.43 (s, 3H), 2.07 (t, 1H, *J* = 2.5 Hz). GC-MS *m*/*z* (%): 289 [M]^+^ (1), 134 (100), 106 (32), 91 (31), 81 (17).

### 4-Methyl-*N*-((5-methylfuran-2-yl)methyl)-*N*-(prop-2-yn-1-yl)benzenesulfonamide,^29^ int-6

Synthesized according to the general procedure GP2. White solid, 3.82 g, 63% yield (3 steps). *R*_*f*_ 0.31 (9/1 PE/EtOAc). ^1^H NMR (600 MHz, CDCl_3_) δ (ppm): 7.75–7.73 (m, 2H), 7.30–7.28 (m, 2H), 6.16 (d, 1H, *J* = 3.0 Hz), 5.86 (dq, 1H, *J* = 3.0 Hz, 1.0 Hz), 4.37 (s, 2H), 4.02 (d, 2H, *J* = 2.5 Hz), 2.43 (s, 3H), 2.20 (d, 3H, *J* = 1.0 Hz), 2.05 (t, 1H, *J* = 2.5 Hz). GC-MS *m*/*z* (%): 303 [M]^+^ (3), 148 (100), 147 (36), 120 (77), 95 (82), 91 (42).

### 4-Methyl-*N*-((5-phenylfuran-2-yl)methyl)-*N*-(prop-2-yn-1-yl)benzenesulfonamide, int-7

Synthesized according to the general procedure GP2. White solid, 2.33 g, 64% yield (3 steps), mp 89–91 °C (Et_2_O). *R*_*f*_ 0.38 (9/1 PE/EtOAc). ^1^H NMR (600 MHz, CDCl_3_) δ (ppm): 7.76–7.74 (m, 2H), 7.56–7.54 (m, 2H), 7.37–7.34 (m, 2H), 7.28–7.24 (m, 3H), 6.55 (d, 1H, *J* = 3.3 Hz), 6.37 (d, 1H, *J* = 3.3 Hz), 4.52 (s, 2H), 4.09 (d, 2H, *J* = 2.5 Hz), 2.38 (s, 3H), 2.11 (t, 1H, *J* = 2.5 Hz). ^13^C{^1^H} NMR (150 MHz, CDCl_3_) δ (ppm): 154.5 (Cq), 148.2 (Cq), 143.8 (Cq), 136.2 (Cq), 130.5 (Cq), 129.7 (CH), 128.7 (CH), 127.8 (CH), 127.7 (CH), 123.9 (CH), 112.3 (CH), 105.7 (CH), 76.7 (Cq), 74.1 (CH), 43.1 (CH_2_), 36.5 (CH_2_), 21.6 (CH_3_). HRMS (ESI) *m*/*z*: [M + Na]^+^ calcd for C_21_H_19_NO_3_SNa^+^ 388.0978; found 388.0974.

### 4-Methyl-*N*-(prop-2-yn-1-yl)-*N*-((5-(thiophen-2-yl)furan-2-yl)methyl)benzenesulfonamide, int-8

Synthesized according to the general procedure GP2. White solid, 2.42 g, 65% yield (3 steps), mp 127–131 °C (decomposition). *R*_*f*_ 0.46 (8/2 DCM/PE). ^1^H NMR (600 MHz, CDCl_3_) δ (ppm): 7.74 (d, 2H, *J* = 8.2 Hz), 7.27 (d, 2H, *J* = 8.3 Hz), 7.22 (d, 1H, *J* = 5.0 Hz), 7.16 (d, 1H, *J* = 3.5 Hz), 7.03–7.00 (m, 1H), 6.38 (d, 1H, *J* = 3.3 Hz), 6.34 (d, 1H, *J* = 3.3 Hz), 4.49 (s, 2H), 4.08 (d, 2H, *J* = 2.4 Hz), 2.39 (s, 3H), 2.11 (t, 1H, *J* = 2.5 Hz). ^13^C{^1^H} NMR (150 MHz, CDCl_3_) δ (ppm): 149.9 (Cq), 147.9 (Cq), 143.8 (Cq), 136.1 (Cq), 133.4 (Cq), 129.7 (CH), 127.8 (CH), 127.7 (CH), 124.5 (CH), 123.0 (CH), 112.2 (CH), 105.8 (CH), 76.7 (Cq), 74.1 (CH), 43.0 (CH_2_), 36.5 (CH_2_), 21.6 (CH_3_). HRMS (ESI) *m*/*z*: [M + Na]^+^ calcd for C_19_H_17_NO_3_S_2_Na^+^ 394.0542; found 394.0528.

### *N*-([2,2′-Bifuran]-5-ylmethyl)-4-methyl-*N*-(prop-2-yn-1-yl)benzenesulfonamide, int-9

Synthesized according to the general procedure GP2. White solid, 2.22 g, 63% yield (3 steps), mp 136–138 °C (EtOAc). *R*_*f*_ 0.43 (7/3 DCM/PE). ^1^H NMR (600 MHz, CDCl_3_) δ (ppm): 7.76–7.73 (m, 2H), 7.39 (dd, 1H, *J* = 1.7, 0.7 Hz), 7.28 (d, 2H, *J* = 8.0 Hz), 3.47 (d, 1H, *J* = 3.1 Hz), 6.45–6.43 (m, 2H), 6.35 (d, 1H, *J* = 3.4 Hz), 4.48 (s, 2H), 4.06 (d, 2H, *J* = 2.4 Hz), 2.40 (s, 3H), 2.09 (t, 1H, *J* = 2.5 Hz). ^13^C{^1^H} NMR (150 MHz, CDCl_3_) δ (ppm): 147.9 (Cq), 147.1 (Cq), 146.3 (Cq), 143.8 (Cq), 142.0 (CH), 136.0 (Cq), 129.6 (CH), 127.8 (CH), 112.0 (CH), 111.5 (CH), 105.8 (CH), 105.6 (CH), 76.6 (Cq), 74.2 (CH), 43.0 (CH_2_), 36.4 (CH_2_), 21.7 (CH_3_). HRMS (ESI) *m*/*z*: [M + Na]^+^ calcd for C_19_H_17_NO_4_SNa^+^ 378.0770; found 378.0781.

### General Procedure A for the Synthesis of Terminal Alkynes with *O* Linker (GP3A)^31^

To a 1.0 M solution of furaldehyde (1.0 equiv, 10 mmol) in MeOH, NaBH_4_ (1.0 equiv) was added portionwise at 0 °C. The mixture was allowed to reach room temperature and stirred overnight. Then, MeOH was evaporated to half of the initial volume under reduced pressure. Water was added, and the mixture was extracted three times with DCM; the combined organic layers were dried over Na_2_SO_4_ and filtered, and the volatiles were evaporated under reduced pressure to obtain the crude furyl alcohol, which was used for the next step without further purification.

To a 1.0 M solution of the crude furanyl alcohol in anhydrous DMF, at 0 °C under a N_2_ atmosphere, NaH (1.5 equiv) was added portionwise. The mixture was stirred at 0 °C for 15 min, then propargyl bromide (1.5 equiv) was added, and the mixture was allowed to reach room temperature and stirred overnight. Then, the mixture was cooled down to 0 °C, water was added, and the mixture was extracted three times with DCM; the combined organic layers were washed three times with water, dried over anhydrous Na_2_SO_4_, and filtered, and the volatiles were removed under reduced pressure. The crude product was purified by flash column chromatography to obtain the pure *O*-propargyl product.

### General Procedure B for the Synthesis of Terminal Alkynes with *O* Linker (GP3B)^32^

To a 0.5 M solution of furaldehyde (1.0 equiv, 10 mmol) in anhydrous THF, at 0 °C under a N_2_ atmosphere, a solution of Grignard reagent (1.2 equiv) was added. The mixture was allowed to reach room temperature and stirred for 1–2 h (GC-MS monitoring). Then, a saturated NH_4_Cl solution was added, and the mixture was extracted three times with DCM; the combined organic layers were dried over Na_2_SO_4_, filtered and the volatiles were evaporated under reduced pressure to obtain the crude furyl alcohol, which was used for the next step without further purification.

To a 1.0 M solution of the crude furanyl alcohol in anhydrous DMF, at 0 °C under a N_2_ atmosphere, NaH (1.5 equiv) was added portionwise. The mixture was stirred at 0 °C for 15 min, then propargyl bromide (1.5 equiv) was added, and the mixture was allowed to reach room temperature and stirred overnight. Then, the mixture was cooled down to 0 °C, water was added, and the mixture was extracted three times with DCM; the combined organic layers were washed three times with water, dried over anhydrous Na_2_SO_4_, and filtered, and the volatiles were removed under reduced pressure. The crude product was purified by flash column chromatography to obtain the pure *O*-propargyl product.

### 2-Methyl-5-((prop-2-yn-1-yloxy)methyl)furan,^31^ int-10

Synthesized according to the general procedure GP3A. Pale yellow oil, 1.01 g, 67% yield (2 steps). *R*_*f*_ 0.18 (99/1 PE/EtOAc). ^1^H NMR (600 MHz, CDCl_3_) δ (ppm): 6.25 (dd, 1H, *J* = 3.0, 0.6 Hz), 5.92 (dq, 1H, *J* = 3.1 Hz, 1.1 Hz), 4.50 (s, 2H), 4.16 (d, 2H, *J* = 2.4 Hz), 2.43 (s, 3H), 2.29 (d, 3H, *J* = 1.0 Hz). GC-MS *m*/*z* (%): 150 [M]^+^ (30), 110 (21), 95 (100).

### 2-Methyl-5-(1-(prop-2-yn-1-yloxy)propyl)furan,^32^ int-11

Synthesized according to the general procedure GP3B with EtMgBr (3.0 M in Et_2_O). Yellow oil, 820 mg, 46% yield (2 steps). *R*_*f*_ 0.29 (98/2 PE/EtOAc). ^1^H NMR (600 MHz, CDCl_3_) δ (ppm): 6.17 (d, 1H, *J* = 3.0 Hz), 5.89 (dq, 1H, *J* = 3.1, 1.0 Hz), 4.34 (t, 1H, *J* = 7.1 Hz), 4.13 (dd, 1H, *J* = 15.8, 2.4 Hz), 3.95 (dd, 1H, *J* = 15.8, 2.4 Hz), 2.38 (t, 1H, *J* = 2.4 Hz), 2.27 (d, 3H, *J* = 1.0 Hz), 1.95–1.81 (m, 2H), 0.90 (t, 3H, *J* = 7.5 Hz). GC-MS *m*/*z* (%): 178 [M]^+^ (5), 149 (100), 123 (38), 120 (24), 109 (36), 91 (22), 77 (33), 43 (41).

### 2-(2,2-Dimethyl-1-(prop-2-yn-1-yloxy)propyl)-5-methylfuran, int-12

Synthesized according to the general procedure GP3B with *t*-BuMgCl (1.0 M in THF). Yellow oil, 720 mg, 35% yield (2 steps). *R*_*f*_ 0.45 (99/1 PE/EtOAc). ^1^H NMR (600 MHz, CDCl_3_) δ (ppm): 6.13 (d, 1H, *J* = 3.0 Hz), 5.91 (dd, 1H, *J* = 3.0, 1.0 Hz), 4.16 (dd, 1H, *J* = 15.6, 2.4 Hz), 4.12 (s, 1H), 3.87 (dd, 1H, *J* = 16.0, 2.3 Hz), 2.37 (t, 1H, *J* = 2.4 Hz), 2.28 (d, 3H, *J* = 0.8 Hz), 0.96 (s, 9H). ^13^C{^1^H} NMR (150 MHz, CDCl_3_) δ (ppm): 151.8 (Cq), 151.0 (Cq), 110.4 (CH), 105.9 (CH), 82.1 (CH), 80.3 (Cq), 74.0 (CH), 56.0 (CH_2_), 35.3 (Cq), 26.4 (CH_3_), 13.8 (CH_3_). HRMS (ESI) *m*/*z*: [M + Na]^+^ calcd for C_13_H_18_O_2_Na^+^ 229.1199; found 229.1193.

### General Procedure for the Sonogashira Coupling (GP4)^33^

In a vial under a N_2_ atmosphere, a 2.0 M solution of the terminal alkyne (1.0 equiv, 1.0–2.0 mmol) in anhydrous THF was prepared. The aryl iodide or aryl bromide (1.5 equiv) and Et_3_N (2.0 equiv) were added, and the mixture was degassed for 10–15 min. Then, [(Ph_3_P)_2_PdCl_2_] (0.02 equiv) and CuI (0.04 equiv) were added, and the mixture was stirred at room temperature (for ArI) or at 60 °C (for ArBr) overnight. After that time, a saturated NH_4_Cl solution was added, and the mixture was extracted three times with EtOAc; the combined organic layers were dried over Na_2_SO_4_ and filtered, and the volatiles were removed under reduced pressure. The crude product was purified by flash column chromatography to obtain pure aryl alkynes **1** as the product.

### 4-Methyl-*N*-((5-methylfuran-2-yl)methyl)-*N*-(3-phenylprop-2-yn-1-yl)benzenesulfonamide, **1a**

Synthesized according to the general procedure GP4 at rt with PhI. White solid, 252 mg, 68% yield, mp 81–83 °C (Et_2_O). *R*_*f*_ 0.17 (95/5 PE/EtOAc). ^1^H NMR (600 MHz, CDCl_3_) δ (ppm): 7.78 (d, 2H, *J* = 8.2 Hz), 7.31–7.23 (m, 5H), 7.09 (d, 2H, *J* = 8.1 Hz), 6.20 (d, 1H, *J* = 2.8 Hz), 5.88 (d, 1H, *J* = 2.0 Hz), 4.42 (s, 2H), 4.24 (s, 2H), 2.34 (s, 3H), 2.22 (s, 3H). ^13^C{^1^H} NMR (150 MHz, CDCl_3_) δ (ppm): 153.0 (Cq), 146.7 (Cq), 143.6 (Cq), 136.2 (Cq), 131.7 (CH), 129.6 (CH), 128.5 (CH), 128.3 (CH), 128.0 (CH), 122.4 (Cq), 111.2 (CH), 106.4 (CH), 85.9 (Cq), 81.9 (Cq), 43.4 (CH_2_), 37.1 (CH_2_), 21.6 (CH_3_), 13.7 (CH_3_). HRMS (ESI) *m*/*z*: [M + Na]^+^ calcd for C_22_H_21_NO_3_SNa^+^ 402.1134; found 402.1127.

### *N*-(3-(4-Methoxyphenyl)prop-2-yn-1-yl)-4-methyl-*N*-((5-methylfuran-2-yl)methyl)benzenesulfonamide, **1b**

Synthesized according to the general procedure GP4 at rt with *p*-iodoanisole. White solid, 568 mg, 69% yield, mp 92–94 °C (Et_2_O). *R*_*f*_ 0.28 (9/1 PE/EtOAc). ^1^H NMR (600 MHz, CDCl_3_) δ (ppm): 7.79–7.77 (m, 2H), 7.27–7.25 (m, 2H), 7.06–7.03 (m, 2H), 6.79–6.76 (m, 2H), 6.19 (d, 1H, *J* = 3.0 Hz), 5.87 (dq, 1H, *J* = 3.0, 1.0 Hz), 4.41 (s, 2H9, 4.22 (s, 2H), 3.80 (s, 3H), 2.36 (s, 3H), 2.22 (d, 3H, *J* = 0.9 Hz). ^13^C{^1^H} NMR (150 MHz, CDCl_3_) δ (ppm): 159.8 (Cq), 153.0 (Cq), 146.8 (Cq), 143.5 (Cq), 136.3 (Cq), 133.2 (CH), 129.6 (CH), 128.0 (CH), 114.5 (Cq), 113.9 (CH), 111.1 (CH), 106.4 (CH), 85.8 (Cq), 80.4 (Cq), 55.4 (CH_3_), 43.3 (CH_2_), 37.2 (CH_2_), 21.6 (CH_3_), 13.7 (CH_3_). HRMS (ESI) *m*/*z*: [M + Na]^+^ calcd for C_23_H_23_NO_4_SNa^+^ 432.1240; found 432.1230.

### *N*-(3-(4-Hydroxyphenyl)prop-2-yn-1-yl)-4-methyl-*N*-((5-methylfuran-2-yl)methyl)benzenesulfonamide, **1c**

Synthesized according to the general procedure GP4 at rt with *p*-iodophenol. White solid, 236 mg, 60% yield, mp 100–103 °C (DCM/pentane). *R*_*f*_ 0.27 (8/2 PE/EtOAc). ^1^H NMR (600 MHz, CDCl_3_) δ (ppm): 7.79 (m, 2H), 7.27–7.24 (m, 2H), 7.01–6.98 (m, 2H), 6.73–6.70 (m, 2H), 6.19 (d, 1H, *J* = 3.1 Hz), 5.87 (dd, 1H, *J* = 3.0, 1.0 Hz), 5.03 (br s, 1H), 4.41 (s, 2H), 4.21 (s, 3H), 2.36 (s, 3H), 2.22 (s, 3H). ^13^C{^1^H} NMR (150 MHz, CDCl_3_) δ (ppm): 156.1 (Cq), 153.0 (Cq), 146.7 (Cq), 143.7 (Cq), 136.1 (Cq), 133.3 (CH), 129.6 (CH), 128.0 (CH), 115.4 (CH), 114.5 (Cq), 111.2 (CH), 106.4 (CH), 85.9 (Cq), 80.2 (Cq), 43.2 (CH_2_), 37.2 (CH_2_), 21.6 (CH_3_), 13.7 (CH_3_). HRMS (ESI) *m*/*z*: [M + H]^+^ calcd for C_22_H_22_NO_4_S^+^ 396.1264; found 396.1265.

### 4-Methyl-*N*-((5-methylfuran-2-yl)methyl)-*N*-(3-(p-tolyl)prop-2-yn-1-yl)benzenesulfonamide, **1d**

Synthesized according to the general procedure GP5 at rt with *p*-iodotoluene. White solid, 272 mg, 69% yield, mp 58–60 °C (Et_2_O). *R*_*f*_ 0.18 (93/7 PE/EtOAc). ^1^H NMR (600 MHz, CDCl_3_) δ (ppm): 7.79–7.77 (m, 2H), 7.26 (d, 2H, *J* = 7.9 Hz), 7.05 (d, 2H, *J* = 7.8 Hz), 6.99–6.97 (m, 2H), 6.19 (d, 1H, *J* = 3.0 Hz), 5.88–5.87 (m, 1H), 4.41 (s, 2H), 4.22 (s, 2H), 2.36 (s, 3H), 2.33 (s, 3H), 2.22 (d, 3H, *J* = 1.0 Hz). ^13^C{^1^H} NMR (150 MHz, CDCl_3_) δ (ppm): 153.0 (Cq), 146.7 (Cq), 143.6 (Cq), 138.7 (Cq), 136.2 (Cq), 131.6 (CH), 129.6 (CH), 129.0 (CH), 128.0 (CH), 119.3 (Cq), 111.1 (CH), 106.4 (CH), 86.0 (Cq), 81.1 (Cq), 43.3 (CH_2_), 37.1 (CH_2_), 21.6 (2 x CH_3_), 13.7 (CH_3_). HRMS (ESI) *m*/*z*: [M + Na]^+^ calcd for C_23_H_23_NO_3_SNa^+^ 416.1291; found 416.1281.

### Methyl 4-(3-((4-Methyl-*N*-((5-methylfuran-2-yl)methyl)phenyl)sulfonamido)prop-1-yn-1-yl)benzoate, **1e**

Synthesized according to the general procedure GP4 at rt with methyl *p*-iodobenzoate. White solid, 373 mg, 85% yield, mp 133–135 °C (Et_2_O). *R*_*f*_ 0.26 (9/1 PE/EtOAc). ^1^H NMR (600 MHz, CDCl_3_) δ (ppm): 7.93–7.90 (m, 2H), 7.79–7.76 (m, 2H), 7.26–7.24 (m, 2H), 7.15–7.13 (m, 2H), 6.19 (d, 1H, *J* = 3.1 Hz), 5.89–5.87 (dq, 1H, *J* = 3.1, 1.2 Hz), 4.42 (s, 2H), 4.25 (s, 2H), 3.92 (d, 3H, *J* = 0.7 Hz), 2.34 (s, 3H), 2.21 (s, 3H). ^13^C{^1^H} NMR (150 MHz, CDCl_3_) δ (ppm): 166.5 (Cq), 153.1 (Cq), 146.6 (Cq), 143.7 (Cq), 136.1 (Cq), 131.6 (CH), 129.8 (Cq), 129.7 (CH), 129.4 (CH), 128.0 (CH), 127.0 (Cq), 111.2 (CH), 106.5 (CH), 85.1 (Cq), 85.1 (Cq), 52.4 (CH_3_), 43.6 (CH_2_), 37.1 (CH_2_), 21.6 (CH_3_), 13.7 (CH_3_). HRMS (ESI) *m*/*z*: [M + Na]^+^ calcd for C_24_H_23_NO_5_SNa^+^ 460.1189; found 460.1180.

### *N*-(3-(4-Acetylphenyl)prop-2-yn-1-yl)-4-methyl-*N*-((5-methylfuran-2-yl)methyl)benzenesulfonamide, **1f**

Synthesized according to the general procedure GP4 at rt with 4′-iodoacetophenone. White solid, 307 mg, 73% yield, mp 91–93 °C (Et_2_O). *R*_*f*_ 0.32 (8/2 PE/EtOAc). ^1^H NMR (600 MHz, CDCl_3_) δ (ppm): 7.85–7.83 (m, 2H), 7.79–7.77 (m, 2H), 7.28–7.25 (m, 2H), 7.19–7.16 (m, 2H), 6.18 (d, 1H, *J* = 3.0 Hz), 5.88 (dq, 1H, *J* = 3.0, 1.0 Hz), 4.42 (s, 2H), 4.26 (s, 2H), 2.59 (s, 3H), 2.35 (s, 3H), 2.21 (s, 3H). ^13^C{^1^H} NMR (150 MHz, CDCl_3_) δ (ppm): 197.3 (Cq), 153.1 (Cq), 146.6 (Cq), 143.7 (Cq), 136.5 (Cq), 136.2 (Cq), 131.8 (CH), 129.7 (CH), 128.2 (CH), 128.0 (CH), 127.2 (Cq), 111.2 (CH), 106.5 (CH), 85.6 (Cq), 85.0 (Cq), 43.6 (CH_2_), 37.1 (CH_2_), 26.7 (CH_3_), 21.6 (CH_3_), 13.7 (CH_3_). HRMS (ESI) *m*/*z*: [M + H]^+^ calcd for C_24_H_24_NO_4_S^+^ 422.1421; found 422.1416.

### *N*-(3-(4-fluorophenyl)prop-2-yn-1-yl)-4-methyl-*N*-((5-methylfuran-2-yl)methyl)benzenesulfonamide, **1g**

Synthesized according to the general procedure GP4 at rt with *p*-fluoroiodobenzene. White solid, 339 mg, 86% yield, mp 86–88 °C (Et_2_O). *R*_*f*_ 0.36 (9/1 PE/EtOAc). ^1^H NMR (600 MHz, CDCl_3_) δ (ppm): 7.78 (d, 2H, *J* = 8.3 Hz), 7.27–7.25 (m, 2H), 7.10–7.06 (m, 2H), 6.97–6.92 (m, 2H), 6.18 (d, 1H, *J* = 3.0 Hz), 5.88 (d, 1H, *J* = 2.9 Hz), 4.41 (s, 2H), 4.22 (s, 2H), 2.36 (s, 3H), 2.21 (s, 3H). ^13^C{^1^H} NMR (150 MHz, CDCl_3_) δ (ppm): 162.6 (Cq, d, *J* = 250.0 Hz), 153.0 (Cq), 146.7 (Cq), 143.6 (Cq), 136.2 (Cq), 133.6 (CH, d, *J* = 8.6 Hz), 129.6 (CH), 128.0 (CH), 118.5 (Cq, d, *J* = 3.3 Hz), 115.6 (CH, d, *J* = 21.6 Hz), 111.1 (CH), 106.4 (CH), 84.8 (Cq), 81.7 (Cq), 43.4 (CH_2_), 37.1 (CH_2_), 21.6 (CH_3_), 13.7 (CH_3_). ^**19**^**F NMR** (564 MHz, CDCl_3_) δ (ppm): – 110.4 (tt, *J* = 8.6, 5.4 Hz). HRMS (ESI) *m*/*z*: [M + Na]^+^ calcd for C_22_H_20_FNO_3_SNa^+^ 420.1040; found 420.1027.

### 4-methyl-*N*-((5-methylfuran-2-yl)methyl)-*N*-(3-(thiophen-2-yl)prop-2-yn-1-yl)benzenesulfonamide, **1h**

Synthesized according to the general procedure GP4 at 60 °C with 2-bromothiophene. White solid, 166 mg, 43% yield, mp 87–89 °C (Et_2_O). *R*_*f*_ 0.34 (9/1 PE/EtOAc). ^1^H NMR (600 MHz, CDCl_3_) δ (ppm): 7.79–7.76 (m, 2H), 7.30–7.27 (m, 2H), 7.21 (dd, 1H, *J* = 5.1, 1.2 Hz), 6.25 (dd, 1H, *J* = 3.6, 1.2 Hz), 6.92 (dd, 1H, *J* = 5.2, 3.6 Hz), 6.19 (d, 1H, *J* = 3.0 Hz), 5.88 (dq, 1H, *J* = 3.0, 1.0 Hz), 4.39 (s, 2H), 4.25 (s, 2H), 2.38 (s, 3H), 2.22 (d, 3H, *J* = 1.1 Hz). ^13^C{^1^H} NMR (150 MHz, CDCl_3_) δ (ppm): 153.1 (Cq), 146.6 (Cq), 143.7 (Cq), 136.0 (Cq), 132.4 (CH), 129.7 (CH), 127.9 (CH), 127.4 (CH), 126.9 (CH), 122.3 (Cq), 111.2 (CH), 106.5 (CH), 85.9 (Cq), 79.1 (Cq), 43.5 (CH_2_), 37.3 (CH_2_), 21.7 (CH_3_), 13.7 (CH_3_). HRMS (ESI) *m*/*z*: [M + H]^+^ calcd for C_20_H_20_NO_3_S_2_^+^ 386.0879; found 386.0879.

### 4-Methyl-*N*-((5-methylfuran-2-yl)methyl)-*N*-(3-(naphthalen-1-yl)prop-2-yn-1-yl)benzenesulfonamide, **1i**

Synthesized according to the general procedure GP4 at rt with 1-iodonaphthalene. White solid, 324 mg, 75% yield, mp 107–109 °C (Et_2_O). *R*_*f*_ 0.41 (9/1 PE/EtOAc). ^1^H NMR (600 MHz, CDCl_3_) δ (ppm): 7.90 (d, 1H, *J* = 7.7 Hz), 7.84–7.79 (m, 4H), 7.53–7.47 (m, 2H), 7.17 (d, 2H, *J* = 8.0 Hz), 6.25 (d, 1H, *J* = 3.0 Hz), 5.91–5.89 (m, 1H), 4.52 (s, 2H), 4.41 (s, 2H), 2.24 (s, 3H), 2.17 (s, 3H). ^13^C{^1^H} NMR (150 MHz, CDCl_3_) δ (ppm): 153.1 (Cq), 146.7 (Cq), 143.7 (Cq), 136.1 (Cq), 133.2 (Cq), 130.7 (CH), 129.7 (CH), 129.0 (CH), 128.4 (CH), 128.1 (Cq), 127.9 (CH), 126.9 (CH), 126.5 (CH), 126.1 (CH), 125.1 (CH), 120.1 (Cq), 11.2 (CH), 106.5 (CH), 86.8 (Cq), 84.2 (Cq), 43.5 (CH_2_), 37.3 (CH_2_), 21.4 (CH_3_), 13.8 (CH_3_). HRMS (ESI) *m*/*z*: [M + Na]^+^ calcd for C_26_H_23_NO_3_SNa^+^ 452.1291; found 452.1278.

### 4-Methyl-*N*-((5-phenylfuran-2-yl)methyl)-*N*-(3-phenylprop-2-yn-1-yl)benzenesulfonamide, **1j**

Synthesized according to the general procedure GP4 at rt with PhI. White solid, 357 mg, 81% yield, 110–111 °C (CHCl_3_). *R*_*f*_ 0.41 (9/1 PE/EtOAc). ^1^H NMR (600 MHz, CDCl_3_) δ (ppm): 7.80 (d, 2H, *J* = 8.3 Hz), 7.60–7.58 (m, 2H), 7.35 (t, 2H, *J* = 7.7 Hz), 7.31–7.29 (m, 1H), 7.26–7.22 (m, 5H), 7.12 (dd, 2H, *J* = 8.3, 1.2 Hz), 6.56 (d, 1H, *J* = 3.3 Hz), 6.41 (d, 1H, *J* = 3.3 Hz), 4.56 (s, 2H), 4.31 (s, 2H), 2.32 (s, 3H). ^13^C{^1^H} NMR (150 MHz, CDCl_3_) δ (ppm): 154.5 (Cq), 148.3 (Cq), 143.7 (Cq), 136.1 (Cq), 131.7 (CH), 130.6 (Cq), 129.7 (CH), 128.7 (Cq), 128.6 (CH), 128.3 (CH), 127.9 (CH), 127.7 (CH), 123.9 (CH), 122.3 (Cq), 112.3 (CH), 105.8 (CH), 86.0 (Cq), 81.9 (Cq), 43.5 (CH_2_), 37.5 (CH_2_), 21.5 (CH_3_). HRMS (ESI) *m*/*z*: [M + H]^+^ calcd for C_27_H_24_NO_3_S^+^ 442.1471; found 442.1463.

### *N*-(3-(4-Methoxyphenyl)prop-2-yn-1-yl)-4-methyl-*N*-((5-phenylfuran-2-yl)methyl)benzenesulfonamide, **1k**

Synthesized according to the general procedure GP4 at rt with *p*-iodoanisole. White solid, 435 mg, 93% yield, 130–132 °C (EtOAc). *R*_*f*_ 0.24 (9/1 PE/EtOAc). ^1^H NMR (600 MHz, CDCl_3_) δ (ppm): 7.81–7.79 (m, 2H), 7.59–7.57 (m, 2H), 7.37–7.34 (m, 2H), 7.27–7.24 (m, 3H), 7.08–7.05 (m, 2H), 6.78–6.75 (m, 2H), 6.56 (d, 1H, *J* = 3.3 Hz), 6.41 (d, 1H, *J* = 3.3 Hz), 4.55 (s, 2H), 4.29 (s, 2H), 3.80 (s, 3H), 2.33 (s, 3H). ^13^C{^1^H} NMR (150 MHz, CDCl_3_) δ (ppm): 159.8 (Cq), 154.5 (Cq), 148.4 (Cq), 143.6 (Cq), 136.2 (Cq), 133.2 (CH), 130.6 (Cq), 129.7 (CH), 128.7 (CH), 127.9 (CH), 127.6 (CH), 123.9 (CH), 114.4 (Cq), 113.9 (CH), 112.2 (CH), 105.8 (CH), 85.9 (Cq), 80.4 (Cq), 55.4 (CH_3_), 43.4 (CH_2_), 37.5 (CH_2_), 21.6 (CH_3_). HRMS (ESI) *m*/*z*: [M + Na]^+^ calcd for C_28_H_25_NO_4_SNa^+^ 494.1397; found 494.1385.

### 4-Methyl-*N*-(3-phenylprop-2-yn-1-yl)-*N*-((5-(thiophen-2-yl)furan-2-yl)methyl)benzenesulfonamide, **1l**

Synthesized according to the general procedure GP4 at rt with PhI. Yellow solid, 318 mg, 71%, mp 90–92 °C (Et_2_O). *R*_*f*_ 0.23 (9/1 PE/EtOAc). ^1^H NMR (600 MHz, CDCl_3_) δ (ppm): 7.80 (d, 2H, *J* = 8.3 Hz), 7.31–7.28 (m, 1H), 7.27–7.23 (m, 4H), 7.21 (dd, 1H, *J* = 5.0, 1.0 Hz), 7.19 (dd, 1H, *J* = 3.6, 1.0 Hz), 7.14–7.11 (m, 2H), 7.01 (dd, 1H, *J* = 5.0, 3.6 Hz), 6.41 (d, 1H, *J* = 3.3 Hz), 6.38 (d, 1H, *J* = 3.4 Hz), 4.53 (s, 2H), 4.30 (s, 2H), 2.32 (s, 3H). ^13^C{^1^H} NMR (150 MHz, CDCl_3_) δ (ppm): 150.0 (Cq), 148.0 (Cq), 143.8 (Cq), 136.0 (Cq), 133.4 (Cq), 131.7 (CH), 129.7 (CH), 128.6 (CH), 128.3 (CH), 127.9 (CH), 127.2 (CH), 124.5 (CH), 123.0 (CH), 122.3 (Cq), 112.2 (CH), 105.8 (CH), 86.1 (Cq), 81.8 (Cq), 43.4 (CH_2_), 37.4 (CH_2_), 21.6 (CH_3_). HRMS (ESI) *m*/*z*: [M + Na]^+^ calcd for C_25_H_21_NO_3_S_2_Na^+^ 470.0855; found 470.0840.

### *N*-([2,2′-Bifuran]-5-ylmethyl)-4-methyl-*N*-(3-phenylprop-2-yn-1-yl)benzenesulfonamide, **1m**

Synthesized according to the general procedure GP4 at rt with PhI. Yellow solid, 303 mg, 70% yield, mp 84–86 °C (CHCl_3_). *R*_*f*_ 0.54 (7/3 DCM/PE). ^1^H NMR (600 MHz, CDCl_3_) δ (ppm): 7.29 (d, 2H, *J* = 8.3 Hz), 7.40–7.39 (m, 1H), 7.31–7.28 (m, 1H), 7.27–7.23 (m, 4H), 7.11–7.09 (m, 2H), 6.49 (d, 1H, *J* = 3.4 Hz), 6.46 (d, 1H, *J* = 3.3 Hz), 6.44 (dd, 1H, *J* = 3.4, 1.8 Hz), 6.39 (d, 1H, *J* = 3.3 Hz), 4.53 (s, 2H), 4.38 (s, 2H), 2.33 (s, 3H). ^13^C{^1^H} NMR (150 MHz, CDCl_3_) δ (ppm): 148.1 (Cq), 147.1 (Cq), 146.3 (Cq), 143.8 (Cq), 142.0 (CH), 136.0 (Cq), 131.7 (CH), 129.7 (CH), 128.6 (CH), 128.3 (CH), 128.0 (CH), 122.3 (Cq), 112.0 (CH), 111.5 (CH), 105.9 (CH), 105.8 (CH), 86.1 (Cq), 81.7 (Cq), 43.4 (CH_2_), 37.4 (CH_2_), 21.6 (CH_3_). HRMS (ESI) *m*/*z*: [M + Na]^+^ calcd for C_25_H_21_NO_4_SNa^+^ 454.1083; found 454.1078.

### 2-Methyl-5-(((3-phenylprop-2-yn-1-yl)oxy)methyl)furan, **1n**

Synthesized according to the general procedure GP4 at rt with PhI. Colorless oil, 166 mg, 74% yield. *R*_*f*_ 0.21 (99/1 PE/EtOAc). ^1^H NMR (600 MHz, CDCl_3_) δ (ppm): 7.48–7.44 (m, 2H), 7.34–7.30 (m, 3H), 6.28 (d, 1H, *J* = 3.0 Hz), 5.94 (dq, 1H, *J* = 3.0, 1.0 Hz), 4.56 (s, 2H), 4.39 (s, 2H), 2.30 (d, 3H, *J* = 0.8 Hz). ^13^C{^1^H} NMR (150 MHz, CDCl_3_) δ (ppm): 153.1 (Cq), 149.2 (Cq), 131.9 (CH), 128.6 (CH), 128.4 (CH), 122.8 (Cq), 111.2 (CH), 106.4 (CH), 86.6 (Cq), 85.0 (Cq), 63.5 (CH_2_), 57.6 (CH_2_), 13.7 (CH_3_). HRMS (ESI) *m*/*z*: [M + H]^+^ calcd for C_15_H_15_O_2_^+^ 227.1067; found 227.1067.

### 2-(((3-(4-Methoxyphenyl)prop-2-yn-1-yl)oxy)methyl)-5-methylfuran, **1o**

Synthesized according to the general procedure GP4 at rt with *p*-iodoanisole. Colorless oil, 170 mg, 66% yield. *R*_*f*_ 0.28 (96/4 PE/EtOAc). ^1^H NMR (600 MHz, CDCl_3_) δ (ppm): 7.41–7.38 (m, 2H), 6.86–6.83 (m, 2H), 6.27 (d, 1H, *J* = 3.0 Hz), 5.93 (dd, 1H, *J* = 3.1, 1.0 Hz), 4.55 (s, 2H), 4.38 (s, 2H), 3.81 (s, 3H), 2.29 (s, 3H). ^13^C{^1^H} NMR (150 MHz, CDCl_3_) δ (ppm): 159.8 (Cq), 153.1 (Cq), 149.3 (Cq), 133.4 (CH), 114.9 (Cq), 114.0 (CH), 111.2 (CH), 106.4 (CH), 86.6 (Cq), 83.5 (Cq), 63.4 (CH_2_), 57.7 (CH_2_), 55.4 (CH_3_), 13.8 (CH_3_). HRMS (ESI) *m*/*z*: [M + H]^+^ calcd for C_16_H_17_O_3_^+^ 257.1172; found 257.1175.

### Methyl 4-(3-((5-Methylfuran-2-yl)methoxy)prop-1-yn-1-yl)benzoate, **1p**

Synthesized according to the general procedure GP4 at rt with methyl *p*-iodobenzoate. White solid, 211 mg, 75% yield, mp 45–47 °C (Et_2_O). *R*_*f*_ 0.15 (96/4 PE/EtOAc). ^1^H NMR (600 MHz, CDCl_3_) δ (ppm): 8.00–7.98 (m, 2H), 7.52–7.50 (m, 2H), 6.28 (d, 1H, *J* = 3.1 Hz), 5.94 (dq, 1H, *J* = 3.0, 1.1 Hz), 4.56 (s, 2H), 4.40 (s, 2H), 3.92 (s, 3H), 2.30 (d, 3H, *J* = 1.0 Hz). ^13^C{^1^H} NMR (150 MHz, CDCl_3_) δ (ppm): 166.6 (Cq), 153.2 (Cq), 149.1 (Cq), 131.8 (CH), 129.9 (Cq), 129.6 (CH), 127.5 (Cq), 111.4 (CH), 106.5 (CH), 88.1 (Cq), 85.9 (Cq), 63.7 (CH_2_), 57.5 (CH_2_), 52.4 (CH_3_), 13.8 (CH_3_). HRMS (ESI) *m*/*z*: [M + Na]^+^ calcd for C_17_H_16_O_4_Na^+^ 307.0941; found 307.0940.

### 2-Methyl-5-(1-((3-phenylprop-2-yn-1-yl)oxy)propyl)furan, **1q**

Synthesized according to the general procedure GP4 at rt with PhI. Colorless oil, 193 mg, 76% yield. *R*_*f*_ 0.34 (99/1 PE/EtOAc). ^1^H NMR (600 MHz, CDCl_3_) δ (ppm): 7.45–7.42 (m, 2H), 7.32–7.29 (m, 3H), 6.22 (d, 1H, *J* = 3.0 Hz), 5.93–5.91 (m, 1H), 4.42 (t, 1H, *J* = 7.1 Hz), 4.36 (d, 1H, *J* = 15.8 Hz), 4.21 (d, 1H, *J* = 15.8 Hz), 2.28 (d, 3H, *J* = 0.9 Hz), 2.00–1.85 (m, 2H), 0.93 (t, 3H, *J* = 7.5 Hz). ^13^C{^1^H} NMR (150 MHz, CDCl_3_) δ (ppm): 152.3 (Cq), 151.6 (Cq), 131.8 (CH), 128.4 (CH), 128.3 (CH), 122.9 (Cq), 109.9 (CH), 106.0 (CH), 85.9 (Cq), 85.6 (Cq), 75.3 (CH), 56.3 (CH_2_), 27.1 (CH_2_), 13.7 (CH_3_), 10.3 (CH_3_). HRMS (ESI) *m*/*z*: [M + Na]^+^ calcd for C_17_H_18_O_2_Na^+^ 277.1199; found 277.1197.

### 2-(2,2-Dimethyl-1-((3-phenylprop-2-yn-1-yl)oxy)propyl)-5-methylfuran, **1r**

Synthesized according to the general procedure GP4 at rt with PhI. Colorless oil, 165 mg, 58% yield. *R*_*f*_ 0.20 (99/1 PE/EtOAc). ^1^H NMR (600 MHz, CDCl_3_) δ (ppm): 7.44–7.42 (m, 2H), 7.32–7.29 (m, 3H), 6.16 (d, 1H, *J* = 3.0 Hz), 5.92 (dq, 1H, *J* = 3.0, 1.0 Hz), 4.38 (d, 1H, *J* = 16.1 Hz), 4.19 (s, 1H), 4.11 (d, 1H, *J* = 16.1 Hz), 2.28 (s, 3H), 0.98 (s, 9H). ^13^C{^1^H} NMR (150 MHz, CDCl_3_) δ (ppm): 151.9 (Cq), 151.2 (Cq), 131.8 (CH), 128.4 (CH), 128.4 8 (CH), 123.1 (Cq), 110.3 (CH), 105.9 (CH), 85.9 (Cq), 82.2 (Cq), 56.9 (CH_2_), 35.4 (Cq), 26.4 (CH_3_), 13.8 (CH_3_). HRMS (ESI) *m*/*z*: [M + Na]^+^ calcd for C_19_H_22_O_2_Na^+^ 305.1512; found 305.1500.

### *N*-(Furan-2-ylmethyl)-4-methyl-*N*-(3-phenylprop-2-yn-1-yl)benzenesulfonamide, **1s**

Synthesized according to the general procedure GP4 at rt with PhI. White solid, 280 mg, 77% yield, mp 68–70 °C (Et_2_O). *R*_*f*_ 0.18 (95/5 PE/EtOAc). ^1^H NMR (600 MHz, CDCl_3_) δ (ppm): 7.79 (d, 2H, *J* = 8.3 Hz), 7.38 (q, 1H, *J* = 0.9 Hz), 7.31–7.23 (m, 5H), 7.11–7.08 (m, 2H), 6.35–6.32 (m, 2H), 4.48 (s, 2H), 4.24 (s, 2H), 2.35 (s, 3H). ^13^C{^1^H} NMR (150 MHz, CDCl_3_) δ (ppm): 148.9 (Cq), 143.7 (Cq), 143.2 (CH), 136.1 (Cq), 131.7 (CH), 129.7 (CH), 128.6 (CH), 128.3 (CH), 128.0 (CH), 122.3 (Cq), 110.6 (CH), 110.1 (CH), 86.0 (Cq), 81.7 (Cq), 43.2 (CH_2_), 37.3 (CH_2_), 21.6 (CH_3_). HRMS (ESI) *m*/*z*: [M + Na]^+^ calcd for C_21_H_19_NO_3_SNa^+^ 388.0978; found 388.0974.

### General Procedure for Gold(I)-Catalyzed Synthesis of Dihydropyridinones and Pyranones (GP5)

A solution of the furan-yne substrate (1.0 equiv, 0.2 mmol) and 4-nitropyridine *N*-oxide (1.2 equiv) in DCE (0.1 M with respect to the furan-yne) was prepared, then [(IPr)Au(NTf_2_)] (0.05 equiv) was added, and the mixture was stirred at room temperature for 20 h. Then, MsOH (5.0 equiv) was added, and the mixture was stirred at room temperature for 1 h. A few drops of Et_3_N were added, and the solvent was evaporated under reduced pressure. The crude product was purified by flash column chromatography to afford pure dihydropyridinones or pyranones **2** as the product.

### (*E*)-5-(3-Oxobut-1-en-1-yl)-4-phenyl-1-tosyl-1,6-dihydropyridin-3(2*H*)-one, **2a**

Synthesized according to the general procedure GP5. Yellow solid, 57 mg, 72% yield, mp 176–177 °C (MeCN, decomposition). The reaction was also repeated at 1.3 mmol scale, obtaining 367 mg (71% yield) of product. *R*_*f*_ 0.21 (75/25 PE/EtOAc). ^1^H NMR (600 MHz, CDCl_3_) δ (ppm): 7.73–7.69 (m, 2H), 7.39–7.34 (m, 5H), 7.03 (d, 1H, *J* = 16.6 Hz), 6.87–6.84 (m, 2H), 6.46 (d, 1H, *J* = 16.6 Hz), 4.31 (s, 2H), 4.04 (s, 2H), 2.44 (s, 3H), 2.19 (s, 3H). ^13^C{^1^H} NMR (150 MHz, CDCl_3_) δ (ppm): 197.3 (Cq), 190.4 (Cq), 144.9 (Cq), 144.1 (Cq), 141.4 (Cq), 137.9 (CH), 133.4 (Cq), 132.2 (CH), 131.3 (Cq), 130.5 (CH), 130.4 (CH), 129.2 (CH), 128.3 (CH), 127.8 (CH), 53.3 (CH_2_), 45.1 (CH_2_), 28.0 (CH_3_), 21.7 (CH_3_). HRMS (ESI) *m*/*z*: [M + H]^+^ calcd for C_22_H_22_NO_4_S^+^ 396.1264; found 396.1262.

### (*E*)-4-(4-Methoxyphenyl)-5-(3-oxobut-1-en-1-yl)-1-tosyl-1,6-dihydropyridin-3(2*H*)-one, **2b**

Synthesized according to the general procedure GP5. Yellow solid, 64 mg, 75% yield, mp 150–153 °C (decomposition). *R*_*f*_ 0.24 (7/3 PE/EtOAc). ^1^H NMR (600 MHz, CDCl_3_) δ (ppm): 7.69 (d, 2H, *J* = 8.3 Hz), 7.35 (d, 2H, *J* = 8.1 Hz), 7.09 (d, 1H, *J* = 16.6 Hz), 6.90–6.87 (m, 2H), 6.82–6.79 (m, 2H), 6.45 (d, 1H, *J* = 16.6 Hz), 4.29 (s, 2H), 4.03 (s, 2H), 3.83 (s, 3H), 2.44 (s, 3H), 2.22 (s, 3H). ^13^C{^1^H} NMR (150 MHz, CDCl_3_) δ (ppm): 197.4 (Cq), 190.7 (Cq), 160.3 (Cq), 144.8 (Cq), 143.5 (Cq), 140.9 (Cq), 138.2 (CH), 133.3 (Cq), 132.0 (CH), 131.8 (CH), 130.4 (CH), 127.8 (CH), 123.3 (Cq), 113.8 (CH), 55.4 (CH_3_), 53.3 (CH_2_), 45.2 (CH_2_), 28.1 (CH_3_), 21.7 (CH_3_). HRMS (ESI) *m*/*z*: [M + Na]^+^ C_23_H_23_NO_5_SNa^+^ 448.1189; found 448.1094.

### (*E*)-4-(4-Hydroxyphenyl)-5-(3-oxobut-1-en-1-yl)-1-tosyl-1,6-dihydropyridin-3(2*H*)-one, **2c**

Synthesized according to the general procedure GP5. Yellow solid, 42 mg, 52% yield, mp 170–174 °C (decomposition). *R*_*f*_ 0.25 (9/1 DCM/EtOAc). ^1^H NMR (600 MHz, CD_3_OD) δ (ppm): 7.74–7.72 (m, 2H), 7.42 (d, 2H, *J* = 7.9 Hz), 7.07 (d, 1H, *J* = 16.4 Hz), 6.78–6.75 (m, 2H), 6.69 (d, 1H, *J* = 16.4 Hz), 6.64–6.62 (m, 2H), 4.51 (s, 2H), 4.17 (s, 2H), 2.45 (s, 3H), 2.24 (s, 3H), 2.19 (s, 1H). ^13^C{^1^H} NMR (150 MHz, CD_3_OD) δ (ppm): 200.5 (Cq), 193.7 (Cq), 160.2 (Cq), 146.9 (Cq), 145.9 (Cq), 143.0 (Cq), 140.2 (CH), 136.9 (Cq), 134.1 (CH), 133.4 (CH), 132.3 (CH), 129.6 (CH), 124.6 (Cq), 116.5 (CH), 55.2 (CH_2_), 47.1 (CH_2_), 29.1 (CH_3_), 22.3 (CH_3_). HRMS (ESI) *m*/*z*: [M + Na]^+^ calcd for C_22_H_21_NO_5_SNa^+^ 434.1033; found 434.1024.

### (*E*)-5-(3-Oxobut-1-en-1-yl)-4-(*p*-tolyl)-1-tosyl-1,6-dihydropyridin-3(2*H*)-one, **2d**

Synthesized according to the general procedure GP5. Yellow solid, 71 mg, 86% yield, mp 166–167 °C (Et_2_O). *R*_*f*_ 0.25 (75/25 PE/EtOAc). ^1^H NMR (600 MHz, CDCl_3_) δ (ppm): 7.71–7.69 (m, 2H), 7.35 (d, 2H, *J* = 8.0 Hz), 7.16 (d, 2H, *J* = 7.8 Hz), 7.07 (d, 1H, *J* = 16.6 Hz), 6.75–6.72 (m, 2H), 6.45 (d, 1H, *J* = 16.5 Hz), 4.30 (s, 2H), 4.04 (s, 2H), 2.44 (s, 3H), 2.36 (s, 3H), 2.21 (s, 3H). ^13^C{^1^H} NMR (150 MHz, CDCl_3_) δ (ppm): 197.4 (Cq), 190.6 (Cq), 144.8 (Cq), 143.7 (Cq), 141.4 (Cq), 139.3 (Cq), 138.1 (CH), 133.4 (Cq), 131.9 (CH), 130.4 (CH), 129.1 (CH), 128.2 (Cq), 127.8 (CH), 53.3 (CH_2_), 45.1 (CH_2_), 28.1 (CH_3_), 21.7 (CH_3_), 21.5 (CH_3_). HRMS (ESI) *m*/*z*: [M + H]^+^ calcd for C_23_H_24_NO_4_S^+^ 410.1421; found 410.1415.

### Methyl (*E*)-4-(3-Oxo-5-(3-oxobut-1-en-1-yl)-1-tosyl-1,2,3,6-tetrahydropyridin-4-yl)benzoate, **2e**

Synthesized according to the general procedure GP5. Light orange solid, 52 mg, 57% yield, mp 186–191 °C (decomposition). *R*_*f*_ 0.21 (65/35 PE/EtOAc). ^1^H NMR (600 MHz, CDCl_3_) δ (ppm): 8.04–8.02 (m, 2H), 7.71–7.69 (m, 2H), 7.36 (d, 2H, *J* = 8.0 Hz), 6.95 (d, 1H, *J* = 16.6 Hz) superimposed to 6.94–6.92 (m, 2H), 6.50 (d, 1H, *J* = 16.5 Hz), 4.34 (s, 2H), 4.06 (s, 2H), 3.93 (s, 3H), 2.45 (s, 3H), 2.20 (s, 3H). ^13^C{^1^H} NMR (150 MHz, CDCl_3_) δ (ppm): 197.0 (Cq), 190.0 (Cq), 166.6 (Cq), 145.0 (Cq), 144.8 (Cq), 140.4 (Cq), 137.0 (CH), 136.0 (Cq), 133.4 (Cq), 132.7 (CH), 130.7 (Cq), 130.6 (CH), 130.5 (CH), 129.5 (CH), 127.8 (CH), 53.2 (CH_2_), 52.5 (CH_3_), 45.1 (CH_2_), 28.2 (CH_3_), 21.7 (CH_3_). HRMS (ESI) *m*/*z*: [M + H]^+^ calcd for C_24_H_24_NO_6_S^+^ 454.1319; found 454.1307.

### (*E*)-4-(4-Acetylphenyl)-5-(3-oxobut-1-en-1-yl)-1-tosyl-1,6-dihydropyridin-3(2*H*)-one, **2f**

Synthesized according to the general procedure GP5. White solid, 37 mg, 43% yield, mp 161–164 °C (decomposition). *R*_*f*_ 0.24 (6/4 PE/EtOAc). ^1^H NMR (600 MHz, CDCl_3_) δ (ppm): 7.96–7.93 (m, 2H), 7.71 (d, 2H, *J* = 8.3 Hz), 7.37 (d, 2H, *J* = 8.0 Hz), 6.96 (d, 1H, *J* = 16.4 Hz) superimposed to 6.98–6.95 (m, 2H), 6.51 (d, 1H, *J* = 16.4 Hz), 4.34 (s, 2H), 4.06 (s, 2H), 2.62 (s, 3H), 2.45 (s, 3H), 2.22 (s, 3H). ^13^C{^1^H} NMR (150 MHz, CDCl_3_) δ (ppm): 197.6 (Cq), 196.9 (Cq), 190.0 (Cq), 145.0 (Cq), 144.8 (Cq), 140.4 (Cq), 137.4 (Cq), 136.9 (CH), 136.2 (Cq), 133.4 (Cq), 132.7 (CH), 130.8 (CH), 130.5 (CH), 128.2 (CH), 127.8 (CH), 53.2 (CH_2_), 45.1 (CH_2_), 28.4 (CH_3_), 26.8 (CH_3_), 21.7 (CH_3_). HRMS (ESI) *m*/*z*: [M + Na]^+^ calcd for C_24_H_23_NO_5_SNa^+^ 460.1189; found 460.1182.

### (*E*)-4-(4-Fluorophenyl)-5-(3-oxobut-1-en-1-yl)-1-tosyl-1,6-dihydropyridin-3(2*H*)-one, **2g**

Synthesized according to the general procedure GP5. Yellow solid, 40 mg, 48% yield, mp 149–153 °C (decomposition). *R*_*f*_ 0.16 (7/3 PE/EtOAc). ^1^H NMR (600 MHz, CDCl_3_) δ (ppm): 7.70 (d, 2H, *J* = 8.3 Hz), 7.36 (d, 2H, *J* = 7.9 Hz), 7.08–7.05 (m, 2H), 7.01 (d, 1H, *J* = 16.4 Hz), 6.87–6.83 (m, 2H), 6.49 (d, 1H, *J* = 16.5 Hz), 4.31 (s, 2H), 4.04 (s, 2H), 2.44 (s, 3H), 2.22 (s, 3H). ^13^C{^1^H} NMR (150 MHz, CDCl_3_) δ (ppm): 197.1 (Cq), 190.4 (Cq), 163.2 (Cq, d, *J* = 250.1 Hz), 144.9 (Cq), 144.4 (Cq), 140.3 (Cq), 137.4 (CH), 133.3 (Cq), 132.4 (CH, d, *J* = 8.7 Hz), 132.3 (CH), 130.4 (CH), 127.8 (CH), 127.1 (Cq), 115.5 (CH, d, *J* = 21.5 Hz), 53.3 (CH_2_), 45.1 (CH_2_), 28.3 (CH_3_), 21.7 (CH_3_). ^19^F NMR (564 MHz, CDCl_3_) δ (ppm): (−111.4)–(−112.0) (m). HRMS (ESI) *m*/*z*: [M + Na]^+^ calcd for C_22_H_20_FNO_4_SNa^+^ 436.0989; found 436.0969.

### (*E*)-5-(3-Oxobut-1-en-1-yl)-4-(thiophen-2-yl)-1-tosyl-1,6-dihydropyridin-3(2*H*)-one, **2h**

Synthesized according to the general procedure GP5. Yellow solid, 24 mg, 30% yield, mp 146–148 (Et_2_O, decomposition). *R*_*f*_ 0.23 (7/3 PE/EtOAc). ^1^H NMR (600 MHz, CDCl_3_) δ (ppm): 7.70–7.67 (m, 2H), 7.52 (dd, 1H, *J* = 5.2, 1.2 Hz), 7.36 (d, 1H, *J* = 16.5 Hz), 7.32 (d, 2H, *J* = 8.0 Hz), 7.06 (dd, 1H, *J* = 5.1, 3.6 Hz), 6.81 (dd, 1H, *J* = 3.6, 1.2 Hz), 6.55 (d, 1H, *J* = 16.5 Hz), 4.31 (s, 2H), 4.1 (s, 2H), 2.39 (s, 3H), 2.29 (s, 3H). ^13^C{^1^H} NMR (150 MHz, CDCl_3_) δ (ppm): 197.3 (Cq), 189.9 (Cq), 144.9 (Cq), 143.9 (Cq), 138.0 (CH), 134.4 (Cq), 133.2 (Cq), 132.4 (CH), 131.8 (CH), 131.1(Cq), 130.5 (CH), 130.4 (CH), 127.7 (CH), 126.7 (CH), 53.3 (CH_2_), 45.7 (CH_2_), 28.3 (CH_3_), 21.7 (CH_3_). HRMS (ESI) *m*/*z*: [M + H]^+^ calcd for C_20_H_20_NO_4_S_2_^+^ 402.0828; found 402.0827.

### (*E*)-4-(Naphthalen-1-yl)-5-(3-oxobut-1-en-1-yl)-1-tosyl-1,6-dihydropyridin-3(2*H*)-one, **2i**

Synthesized according to the general procedure GP5. Yellow solid, 65 mg, 73% yield, mp 131–141 °C (decomposition). *R*_*f*_ 0.16 (7/3 PE/EtOAc). ^1^H NMR (600 MHz, CDCl_3_) δ (ppm): 7.89 (d, 1H, *J* = 8.3 Hz), 7.87 (d, 1H, *J* = 8.2 Hz), 7.77 (d, 2H, *J* = 8.4 Hz), 7.49–7.44 (m, 2H), 7.42 (d, 2H, *J* = 7.9 Hz), 7.38–7.35 (m, 1H), 7.20 (d, 1H, *J* = 8.4 Hz), 6.91 (dd, 1H, *J* = 7.0, 1.0 Hz), 6.79 (d, 1H, *J* = 16.6 Hz), 6.45 (d, 1H, *J* = 16.6 Hz), 4.42 (d, 1H, *J* = 17.2 Hz), 4.34 (d, 1H, *J* = 17.4 Hz), 4.14 (dd, 1H, *J* = 16.7, 1.3 Hz), 4.04 (dd, 1H, *J* = 16.7, 1.4 Hz), 2.49 (s, 3H), 2.01 (s, 3H). ^13^C{^1^H} NMR (150 MHz, CDCl_3_) δ (ppm): 197.3 (Cq), 190.3 (Cq), 152.2 (CH), 146.4 (Cq), 145.0 (Cq), 140.8 (Cq), 137.5 (CH), 133.6 (Cq), 133.0 (Cq), 132.5 (CH), 132.0 (Cq), 130.5 (CH), 129.8 (CH), 129.4 (Cq), 128.8 (CH), 128.4 (CH), 127.9 (CH), 126.8 (CH), 126.4 (CH), 125.1 (CH), 125.0 (CH), 53.3 (CH_2_), 44.9 (CH_2_), 27.7 (CH_3_), 21.8 (CH_3_). HRMS (ESI) *m*/*z*: [M + H]^+^ calcd for C_26_H_24_NO_4_S^+^ 446.1421; found 446.1410.

### (*E*)-5-(3-Oxo-3-phenylprop-1-en-1-yl)-4-phenyl-1-tosyl-1,6-dihydropyridin-3(2*H*)-one, **2j**

Synthesized according to the general procedure GP5. Yellow solid, 57 mg, 62% yield, mp 175–178 °C (decomposition). *R*_*f*_ 0.23 (8/2 PE/EtOAc). ^1^H NMR (600 MHz, CDCl_3_) δ (ppm): 7.92 (d, 2H, *J* = 7.2 Hz), 7.72 (d, 2H, *J* = 8.3 Hz), 7.64–7.60 (m, 1H), 7.51 (t, 2H, *J* = 7.8 Hz), 7.37–7.34 (m, 5H), 7.29 (d, 2H, *J* = 1.3 Hz), 6.82 (dd, 2H, *J* = 7.7, 1.6 Hz), 4.49 (s, 2H), 4.11 (s, 2H), 2.44 (s, 3H). ^13^C{^1^H} NMR (150 MHz, CDCl_3_) δ (ppm): 190.6 (Cq), 189.3 (Cq), 144.9 (Cq), 144.1 (Cq), 141.5 (Cq), 139.2 (CH), 137.2 (Cq), 133.7 (CH), 133.7 (Cq), 131.3 (Cq), 130.5 (CH), 130.5 (CH), 129.1 (CH), 129.0 (CH), 128.7 (CH), 128.3 (CH), 127.8 (CH), 127.7 (CH), 53.3 (CH_2_), 45.2 (CH_2_), 21.7 (CH_3_). HRMS (ESI) *m*/*z*: [M + Na]^+^ calcd for C_27_H_23_NO_4_SNa^+^ 480.1240; found 480.1220.

### (*E*)-4-(4-Methoxyphenyl)-5-(3-oxo-3-phenylprop-1-en-1-yl)-1-tosyl-1,6-dihydropyridin-3(2*H*)-one, **2k**

Synthesized according to the general procedure GP5. Yellow solid, 77 mg, 78% yield, mp 159–163 °C (decomposition). *R*_*f*_ 0.40 (99/1 DCM/EtOAc). ^1^H NMR (600 MHz, CDCl_3_) δ (ppm): 7.96–7.94 (m, 2H), 7.73–7.70 (m, 2H), 7.63 (tt, 1H, *J* = 7.1, 1.2 Hz), 7.54–7.51 (m, 2H), 7.36 (d, 1H, *J* = 16.0 Hz) superimposed to 7.34 (d, 2H, *J* = 7.9 Hz), 7.28 (d, 1H, *J* = 16.0 Hz), 6.88–6.86 (m, 2H), 6.79–6.77 (m, 2H), 4.47 (s, 2H), 4.09 (s, 2H), 3.82 (s, 3H), 2.43 (s, 3H). ^13^C{^1^H} NMR (150 MHz, CDCl_3_) δ (ppm): 190.9 (Cq), 189.3 (Cq), 160.3 (Cq), 144.9 (Cq), 143.4 (Cq), 141.1 (Cq), 139.7 (CH), 137.7 (Cq), 133.7 (CH), 133.7 (Cq), 132.1 (CH), 130.5 (CH), 129.1 (CH), 128.8 (CH), 127.7 (CH), 127.3 (CH), 123.4 (Cq), 113.8 (CH), 55.4 (CH_3_), 53.4 (CH_2_), 45.4 (CH_2_), 21.7 (CH_3_). HRMS (ESI) *m*/*z*: [M + Na]^+^ calcd for C_28_H_25_NO_5_SNa^+^ 510.1346; found 510.1324.

### (*E*)-5-(3-Oxo-3-(thiophen-2-yl)prop-1-en-1-yl)-4-phenyl-1-tosyl-1,6-dihydropyridin-3(2*H*)-one, **2l**

Synthesized according to the general procedure GP5. Yellow solid, 58 mg, 63% yield, mp 145–149 °C (decomposition). *R*_*f*_ 0.24 (7/3 PE/EtOAc). ^1^H NMR (600 MHz, CDCl_3_) δ (ppm): 7.82 (dd, 1H, *J* = 3.8, 1.0 Hz), 7.74 (dd, 1H, *J* = 4.9, 1.0 Hz), 7.72–7.70 (m, 2H), 7.37–7.32 (m, 5H) superimposed to 7.32 (d, 1H, *J* = 15.6 Hz), 7.21 (dd, 1H, *J* = 4.9, 3.9 Hz), 7.14 (d, 1H, *J* = 15.8 Hz), 6.80–8.78 (m, 2H), 4.49 (s, 2H), 4.12 (s, 2H), 2.44 (s, 3H). ^13^C{^1^H} NMR (150 MHz, CDCl_3_) δ (ppm): 190.6 (Cq), 180.9 (Cq), 144.9 (Cq), 144.8 (Cq), 143.8 (Cq), 141.6 (Cq), 138.6 (CH), 135.4 (CH), 133.8 (Cq), 132.8 (CH), 131.2 (Cq), 130.5 (CH), 130.5 (CH), 129.2 (CH), 128.7 (CH), 128.3 (CH), 127.7 (CH), 127.5 (CH), 53.4 (CH_2_), 45.3 (CH_2_), 21.7 (CH_3_). HRMS (ESI) *m*/*z*: [M + Na]^+^ calcd for C_25_H_21_NO_4_S_2_Na^+^ 486.0804; found 486.0784.

### (*E*)-5-(3-(Furan-2-yl)-3-oxoprop-1-en-1-yl)-4-phenyl-1-tosyl-1,6-dihydropyridin-3(2*H*)-one, **2m**

Synthesized according to the general procedure GP5. Yellow solid, 37 mg, 41% yield, mp 159–162 °C (decomposition). *R*_*f*_ 0.20 (99/1 DCM/EtOAc). ^1^H NMR (600 MHz, CDCl_3_) δ (ppm): 7.73–7.71 (m, 2H), 7.68 (dd, 1H, *J* = 1.7, 0.8 Hz), 7.37–7.31 (m, 7H), 7.22 (d, 1H, *J* = 16.1 Hz), 6.80–6.78 (m, 2H), 6.63 (dd, 1H, *J* = 3.6, 1.7 Hz), 4.50 (s, 2H), 4.12 (s, 2H), 2.44 (s, 3H). ^13^C{^1^H} NMR (150 MHz, CDCl_3_) δ (ppm): 190.6 (Cq), 176.7 (Cq), 153.3 (Cq), 147.3 (CH), 144.9 (Cq), 143.9 (Cq), 141.6 (Cq), 138.5 (CH), 133.9 (Cq), 131.2 (Cq), 130.6 (CH), 130.5 (CH), 129.2 (CH), 128.3 (CH), 127.7 (CH), 127.2 (CH), 118.7 (CH), 113.2 (CH), 53.4 (CH_2_), 45.2 (CH_2_), 21.7 (CH_3_). HRMS (ESI) *m*/*z*: [M + H]^+^ calcd for C_25_H_22_NO_5_S^+^ 448.1213; found 448.1202.

### (*E*)-5-(3-Oxobut-1-en-1-yl)-4-phenyl-2*H*-pyran-3(6*H*)-one, **2n**

Synthesized according to the general procedure GP5. Yellow solid, 38 mg, 78% yield, mp 104–106 °C (Et_2_O). *R*_*f*_ 0.44 (6/4 PE/EtOAc). ^1^H NMR (600 MHz, CDCl_3_) δ (ppm): 7.46–7.41 (m, 3H), 7.20–7.17 (m, 2H) superimposed to 7.15 (d, 1H, *J* = 16.7 Hz), 6.32 (d, 1H, *J* = 16.7 Hz), 4.72 (s, 2H), 4.34 (s, 2H), 2.19 (s, 3H). ^13^C{^1^H} NMR (150 MHz, CDCl_3_) δ (ppm): 197.6 (Cq), 193.2 (Cq), 146.6 (Cq), 140.1 (Cq), 137.5 (CH), 132.0 (CH), 131.3 (Cq), 130.7 (CH), 129.1 (CH), 128.4 (CH), 72.5 (CH_2_), 65.6 (CH_2_), 27.6 (CH_3_). HRMS (ESI) *m*/*z*: [M + H]^+^ calcd for C_15_H_15_O_3_^+^ 243.1016; found 243.1017.

### (*E*)-4-(4-Methoxyphenyl)-5-(3-oxobut-1-en-1-yl)-2*H*-pyran-3(6*H*)-one, **2o**

Synthesized according to the general procedure GP5. Yellow solid, 16 mg, 29% yield. *R*_*f*_ 0.35 (75/25 PE/EtOAc). ^1^H NMR (600 MHz, CDCl_3_) δ (ppm): 7.21 (d, 1H, *J* = 16.8 Hz), 7.15–7.12 (m, 2H), 6.98–6.95 (m, 2H), 6.32 (d, 1H, *J* = 16.7 Hz), 4.70 (s, 2H), 4.33 (s, 2H), 3.86 (s, 3H), 2.21 (s, 3H). ^13^C{^1^H} NMR (150 MHz, CDCl_3_) δ (ppm): 197.7 (Cq), 193.6 (Cq), 160.3 (Cq), 145.0 (Cq), 139.7 (Cq), 137.9 (CH), 132.3 (CH), 131.6 (CH), 123.4 (Cq), 113.9 (CH), 72.6 (CH_2_), 65.7 (CH_2_), 55.5 (CH_3_), 27.7 (CH_3_). HRMS (ESI) *m*/*z*: [M + H]^+^ calcd for C_16_H_17_O_4_^+^ 273.1121; found 273.1124.

### Methyl 4-(3-Oxo-5-(3-oxobut-1-en-1-yl)-3,6-dihydro-2*H*-pyran-4-yl)benzoate, **2p**

Synthesized according to the general procedure GP5. Yellow solid, 45 mg, 75% yield, *E/Z* ratio 40/60. *R*_*f*_ 0.13 (7/3 PE/EtOAc). **(*****E*****)-2p**^1^H NMR (600 MHz, CDCl_3_) δ (ppm): 8.11 (d, 2H, *J* = 8.1 Hz), 7.27 (d, 2H, *J* = 8.3 Hz), 7.07 (d, 1H, *J* = 16.6 Hz), 6.35 (d, 1H, *J* = 16.7 Hz), 4.73 (s, 2H), 4.35 (s, 2H), 3.95 (s, 3H), 2.19 (s, 3H). ^13^C{^1^H} NMR (150 MHz, CDCl_3_) δ (ppm): 197.2 (Cq), 192.7 (Cq), 166.7 (Cq), 147.3 (Cq), 139.2 (Cq), 136.6 (CH), 136.0 (Cq), 132.5 (CH), 130.8 (CH), 130.7 (Cq), 129.6 (CH), 72.4 (CH_2_), 65.5 (CH_2_), 52.4 (CH_3_), 27.8 (CH_3_). **(*****Z*****)-2p**^1^H NMR (600 MHz, CDCl_3_) δ (ppm): 8.02 (d, 2H, *J* = 8.2 Hz), 7.20 (d, 2H, *J* = 8.3 Hz), 6.25 (d, 1H, *J* = 12.3 Hz), 6.21 (d, 1H, *J* = 12.3 Hz), 4.56 (s, 2H), 4.35 (s, 2H), 3.92 (s, 3H), 2.23 (s, 3H). ^13^C{^1^H} NMR (150 MHz, CDCl_3_) δ (ppm): 198.3 (Cq), 193.0 (Cq), 166.8 (Cq), 153.2 (Cq), 137.3 (Cq), 135.9 (Cq), 135.3 (CH), 131.2 (CH), 130.5 (CH), 130.2 (Cq), 129.3 (CH), 72.8 (CH_2_), 67.6 (CH_2_), 52.4 (CH_3_), 31.1 (CH_3_). HRMS (ESI) *m*/*z*: [M + Na]^+^ calcd for C_17_H_16_O_5_Na^+^ 323.0890; found 323.0873.

### (*E*)-6-Ethyl-5-(3-oxobut-1-en-1-yl)-4-phenyl-2*H*-pyran-3(6*H*)-one, **2q**

Synthesized according to the general procedure GP5. Yellow oil, 33 mg, 62% yield. *R*_*f*_ 0.12 (9/1 PE/EtOAc). ^1^H NMR (600 MHz, CDCl_3_) δ (ppm): 7.34–7.27 (m, 3H), 7.07–7.04 (m, 2H), 6.29 (dd, 1H, *J* = 12.2, 1.6 Hz), 6.10 (d, 1H, *J* = 12.2 Hz), 4.75–4.72 (m, 1H), 4.42 (d, 1H, *J* = 16.4 Hz), 4.32 (dd, 1H, *J* = 16.4, 1.3 Hz), 2.08 (s, 3H), 1.79–1.73 (m, 2H), 1.03 (t, 3H, *J* = 7.4 Hz). ^13^C{^1^H} NMR (150 MHz, CDCl_3_) δ (ppm): 197.7 (Cq), 193.3 (Cq), 156.9 (Cq), 136.8 (CH), 134.9 (Cq), 133.2 (Cq), 130.1 (CH), 129.6 (CH), 128.1 (CH), 128.1 (CH), 77.2 (CH), 70.5 (CH_2_), 30.9 (CH_3_), 25.9 (CH_2_), 10.1 (CH_3_). HRMS (ESI) *m*/*z*: [M + H]^+^ calcd for C_17_H_19_O_3_^+^ 271.1329; found 271.1338.

### (*E*)-6-(*tert*-Butyl)-5-(3-oxobut-1-en-1-yl)-4-phenyl-2*H*-pyran-3(6*H*)-one, **2r**

Synthesized according to the general procedure GP5. Brown oil, 28 mg, 47% yield. *R*_*f*_ 0.17 (95/5 PE/EtOAc). ^1^H NMR (600 MHz, CDCl_3_) δ (ppm): 7.34–7.27 (m, 3H), 7.05–7.02 (m, 2H), 6.41 (d, 1H, *J* = 12.1 Hz), 6.00 (d, 1H, *J* = 12.1 Hz), 4.48 (d, 1H, *J* = 16.5 Hz), 4.41 (s, 1H) superimposed to 4.39 (d, 1H, *J* = 16.7 Hz), 1.08 (s, 9H). ^13^C{^1^H} NMR (150 MHz, CDCl_3_) δ (ppm): 197.6 (Cq), 193.2 (Cq), 139.3 (Cq), 135.6 (Cq), 133.9 (Cq), 129.7 (CH), 129.7 (CH), 128.3 (CH), 128.3 (CH), 128.0 (CH), 84.2 (CH), 70.4 (CH_2_), 37.6 (Cq), 31.0 (CH_3_), 28.2 (CH_3_). HRMS (ESI) *m*/*z*: [M + Na]^+^ calcd for C_19_H_22_O_3_Na^+^ 321.1461; found 321.1443.

### Gold(I)-Catalyzed Synthesis of (*E*)-4-(4-Benzoyl-1-tosyl-2,5-dihydro-1*H*-pyrrol-3-yl)but-3-en-2-one, **3a**

A solution of **1a** (1.0 equiv, 0.2 mmol) and 8-methylquinoline *N*-oxide (1.2 equiv) in DCE (0.1 M with respect to the furan-yne) was prepared, then [(IPr)Au(NTf_2_)] (0.05 equiv) was added, and the mixture was stirred at 80 °C for 6 h. Then, a few drops of Et_3_N were added, and the solvent was evaporated under reduced pressure. The crude product was purified by flash column chromatography to afford pure dihydropyrrole **3a** as the product. Yellow solid, 21 mg, 27% yield, mp 139–140 °C (Et_2_O). *R*_*f*_ 0.24 (75/25 PE/EtOAc). ^1^H NMR (600 MHz, CDCl_3_) δ (ppm): 7.77–7.75 (m, 2H), 7.67–7.65 (m, 2H), 7.64–7.61 (m, 1H), 7.49–7.46 (m, 2H), 7.37 (d, 2H, *J* = 8.1 Hz), 6.93 (d, 1H, *J* = 16.4 Hz), 6.01 (d, 1H, *J* = 16.4 Hz), 4.56 (t, 2H, *J* = 4.1 Hz), 4.46 (t, 2H, *J* = 4.1 Hz), 2.46 (s, 3H), 2.06 (s, 3H). ^13^C{^1^H} NMR (150 MHz, CDCl_3_) δ (ppm): 197.7 (Cq), 191.8 (Cq), 144.4 (Cq), 140.3 (Cq), 138.5 (Cq), 137.2 (Cq), 134.3 (CH), 133.3 (Cq), 132.3 (CH), 132.2 (CH), 130.3 (CH), 129.3 (CH), 129.1 (CH), 127.7 (CH), 57.5 (CH_2_), 55.2 (CH_2_), 27.3 (CH_3_), 21.7 (CH_3_). HRMS (ESI) *m*/*z*: [M + Na]^+^ calcd for C_22_H_21_NO_4_SNa^+^ 418.1083; found 418.1057.

### Gold(I)-Catalyzed Synthesis of (*E*)-4-Methyl-*N*-((5-methylfuran-2-yl)methyl)-*N*-(3-oxo-3-phenylprop-1-en-1-yl)benzenesulfonamide, **4a**

A solution of **1a** (1.0 equiv, 0.2 mmol) and 8-methylquinoline *N*-oxide (1.2 equiv) in DCE (0.1 M with respect to the furan-yne) was prepared, then [(MorDalPhos)Au(NCMe)]SbF_6_ (0.05 equiv) was added, and the mixture was stirred at room temperature for 20 h. Then, a few drops of Et_3_N were added, and the solvent was evaporated under reduced pressure. The crude product was purified by flash column chromatography to afford pure vinyl ketone **4a** as the product. White solid, 59 mg, 74% yield, mp 127–128 °C (EtOAc). *R*_*f*_ 0.20 (9/1 PE/EtOAc). ^1^H NMR (600 MHz, CDCl_3_) δ (ppm): 8.26 (d, 1H, *J* = 13.6 Hz), 7.88–7.85 (m, 2H), 7.71 (d, 2H, *J* = 8.4 Hz), 7.54–7.51 (m, 1H), 7.46–7.42 (m, 2H), 7.29 (d, 2H, *J* = 8.0 Hz), 6.50 (d, 1H, *J* = 13.6 Hz), 6.07 (d, 1H, *J* = 3.1 Hz), 5.85–5.84 (m, 1H), 4.74 (s, 2H), 2.42 (s, 3H), 2.07 (d, 3H, *J* = 1.1 Hz). ^13^C{^1^H} NMR (150 MHz, CDCl_3_) δ (ppm): 189.4 (Cq), 152.4 (Cq), 146.0 (Cq), 144.9 (Cq), 142.6 (CH), 138.7 (Cq), 135.7 (Cq), 132.5 (CH), 130.1 (CH), 128.6 (CH), 128.2 (CH), 127.6 (CH), 110.7 (CH), 106.7 (CH), 104.0 (CH), 43.3 (CH_2_), 21.7 (CH_3_), 13.5 (CH_3_). HRMS (ESI) *m*/*z*: [M + H]^+^ calcd for C_22_H_22_NO_4_S^+^ 396.1264; found 396.1263.

### Synthesis of 5-(3-Oxo-1-(phenylthio)butyl)-4-phenyl-1-tosyl-1,6-dihydropyridin-3(2*H*)-one, **5**

A reported procedure was followed.^15^ To a 0.1 M solution of **2a** (1.0 equiv, 0.1 mmol) in DCM, at 0 °C under air, thiophenol (1.2 equiv) and Et_3_N (0.1 equiv) were added. The mixture was allowed to warm to room temperature and was stirred for 2 h. Then, the volatiles were removed under reduced pressure. The crude product was purified by flash column chromatography to obtain pure sulfide **5** as the product. Two diastereoisomers, arising from the chiral carbon and atropisomerism, could be separated. Colorless oil, 34 mg, 68% overall yield. **5-D1***R*_*f*_ 0.15 (8/2 PE/EtOAc). ^1^H NMR (600 MHz, CDCl_3_) δ (ppm): 7.70–7.68 (m, 2H), 7.34 (d, 2H, *J* = 7.9 Hz), 7.32–7.30 (m, 3H), 7.29–7.26 (m, 1H), 7.25–7.22 (m, 2H), 7.12–7.10 (m, 2H), 6.81–6.78 (m, 2H), 4.17 (dd, 1H, *J* = 18.4, 1.6 Hz), 4.06 (dd, 1H, *J* = 18.4, 1.6 Hz), 3.98 (dd, 1H, *J* = 16.7, 1.6 Hz), 3.79 (dd, 1H, *J* = 16.7, 1.7 Hz), 3.38 (t, 1H, *J* = 7.7 Hz), 2.65 (dd, 1H, *J* = 14.8,, 7.4 Hz), 2.59 (dd, 1H, *J* = 14.9, 8.1 Hz), 2.45 (s, 3H), 2.07 (s, 3H). ^13^C{^1^H} NMR (150 MHz, CDCl_3_) δ (ppm): 203.3 (Cq), 190.1 (Cq), 152.6 (Cq), 144.6 (Cq), 138.0 (Cq), 133.4 (Cq), 132.9 (CH), 132.7 (Cq), 131.8 (Cq), 130.3 (CH), 129.5 (CH), 129.5 (CH), 128.8 (CH), 128.7 (CH), 128.4 (CH), 127.8 (CH), 54.5 (CH), 52.8 (CH_2_), 49.2 (CH_2_), 34.1 (CH_2_), 27.6 (CH_3_), 21.7 (CH_3_). HRMS (ESI) *m*/*z*: [M + Na]^+^ calcd for C_28_H_27_NO_4_S_2_Na^+^ 528.1274, found 528.1265. **5-D2***R*_*f*_ 0.08 (8/2 PE/EtOAc). ^1^H NMR (600 MHz, CDCl_3_) δ (ppm): 7.75 (d, 2H, *J* = 8.2 Hz), 7.40–7.35 (m, 3H), 7.33–7.30 (m, 1H), 7.26–7.21 (m, 6H), 7.20–7.14 (m, 2H), 4.56 (dd, 1H, *J* = 17.1, 1.6 Hz), 4.32 (t, 1H, *J* = 7.6 Hz), 3.93 (dd, 1H, *J* = 17.0, 1.7 Hz) superimposed to 3.92 (dd, 1H, *J* = 16.8, 1.6 Hz), 3.79 (dd, 1H, *J* = 16.7, 1.7 Hz), 2.86 (dd, 1H, *J* = 17.1, 7.5 Hz), 2.65 (dd, 1H, *J* = 17.1, 7.5 Hz), 2.46 (s, 3H), 2.10 (s, 3H). ^13^C{^1^H} NMR (150 MHz, CDCl_3_) δ (ppm): 203.8 (Cq), 190.7 (Cq), 152.2 (Cq), 144.7 (Cq), 137.3 (Cq), 134.6 (CH), 133.0 (Cq), 131.9 (Cq), 131.8 (Cq), 130.3 (CH), 129.6 (CH), 129.5 (CH), 129.1 (CH), 128.3 (CH), 128.2 (CH), 128.1 (CH), 128.1 (CH), 53.4 (CH_2_), 46.7 (CH), 45.7 (CH_2_), 44.9 (CH_2_), 30.4 (CH_3_), 21.7 (CH_3_). HRMS (ESI) *m*/*z*: [M + Na]^+^ calcd for C_28_H_27_NO_4_S_2_Na^+^ 528.1274; found 528.1265.

### Synthesis of 5-(3-Oxobutyl)-4-phenyl-1-tosyl-1,6-dihydropyridin-3(2*H*)-one, **6**

To 0.1 M solution of **2a** (1.0 equiv, 0.1 mmol) in EtOAc, Pd/C (10% w/w) was added. The flask was evacuated and backfilled with H_2_ three times, and then the mixture was stirred at 50 °C under a H_2_ atmosphere for 2 h. After that time, the mixture was cooled down to room temperature and filtered over a Celite pad, and the volatiles were removed under reduced pressure. The crude product was purified by flash column chromatography to afford the partially hydrogenated product **6**. Colorless oil, 28 mg, 71% yield. *R*_*f*_ 0.22 (65/35 PE/EtOAc). ^1^H NMR (600 MHz, CDCl_3_) δ (ppm): 7.72–7.69 (m, 2H), 7.41–7.36 (m, 2H), 7.34–7.29 (m, 3H), 6.84–6.82 (m, 2H), 4.05(s, 2H), 3.91 (s, 2H), 2.46 (s, 3H), 2.43 (t, 2H, *J* = 7.7 Hz), 2.33 (t, 2H, *J* = 7.6 Hz), 2.05 (s, 3H). ^13^C{^1^H} NMR (150 MHz, CDCl_3_) δ (ppm): 206.2 (Cq), 190.2 (Cq), 154.7 (Cq), 144.7 (Cq), 137.3 (Cq), 133.1 (Cq), 132.9 (Cq), 130.3 (CH), 129.4 (CH), 128.6 (CH), 128.2 (CH), 127.9 (CH), 52.9 (CH_2_), 48.1 (CH_2_), 40.9 (CH_2_), 29.8 (CH_3_), 26.9 (CH_2_), 21.7 (CH_3_). HRMS (ESI) *m*/*z*: [M + H]^+^ calcd for C_22_H_24_NO_4_S^+^ 398.1421; found 398.1418.

### Synthesis of (*E*)-5-(3-Hydroxybut-1-en-1-yl)-4-phenyl-1-tosyl-1,2,3,6-tetrahydropyridin-3-ol, **7**

To 0.1 M solution of **2a** (1.0 equiv, 0.1 mmol) in DCM/MeOH 1/1 at 0 °C, NaBH_4_ (1.0 equiv) was added, and the mixture was allowed to warm to room temperature and stirred for 1.5 h. Then, the solvent was evaporated under reduced pressure to a quarter of the initial volume. Water was added, and the mixture was extracted three times with DCM; the combined organic layers were dried over anhydrous Na_2_SO_4_ and filtered, and the volatiles were removed under reduced pressure. The crude product was purified by flash column chromatography to afford the diol product **7** as a 1/1 mixture of diastereoisomers. Colorless oil, 21 mg, 53% yield. *R*_***f***_ 0.24 (6/4 PE/EtOAc). ^1^H NMR (600 MHz, CDCl_3_) δ (ppm): 7.76 (d, 2H, *J* = 8.2 Hz) superimposed to 7.76 (d, 2H, *J* = 8.3 Hz), 7.40–7.37 (m, 4H), 7.26–7.34 (m, 4H), 7.33–7.30 (m, 2H), 7.17–7.14 (m, 4H), 6.12 (d, 2H, *J* = 16.2 Hz), 5.71 (dd, 1H, *J* = 16.2, 6.4 Hz) superimposed to 5.71 (dd, 1H, *J* = 16.2, 6.7 Hz), 4.42–4.38 (m, 2H), 4.25–4.17 (m, 4H), 3.74 (dd, 1H, *J* = 11.8, 3.4 Hz), 3.68 (dd, 1H, *J* = 11.8, 3.6 Hz), 3.44 (d, 1H, *J* = 15.6 Hz), 3.40 (d, 1H, *J* = 15.6 Hz), 3.02 (dd, 1H, *J* = 11.9, 3.3 Hz), 2.95 (dd, 1H, *J* = 11.8, 3.2 Hz), 2.46 (s, 6H), 1.23 (d, 3H, *J* = 6.4 Hz), 1.20 (d, 3H, *J* = 6.3 Hz). ^13^C{^1^H} NMR (150 MHz, CDCl_3_) δ (ppm): 144.3 (Cq), 144.2 (Cq), 138.4 (Cq), 138.4 (Cq), 138.2 (Cq), 138.1 (Cq), 134.7 (CH), 134.6 (CH), 132.9 (Cq), 132.8 (Cq), 130.1 (CH), 129.3 (CH), 129.3 (CH), 128.8 (Cq), 128.8 (Cq), 128.6 (CH), 128.0 (CH), 128.0 (CH), 126.2 (CH), 125.9 (CH), 69.1 (CH), 69.0 (CH), 67.8 (CH), 67.8 (CH), 50.7 (CH_2_), 50.6 (CH_2_), 45.5 (CH_2_), 45.5 (CH_2_), 23.5 (CH_3_), 23.5 (CH_3_), 21.7 (CH_3_). HRMS (ESI) *m*/*z*: [M + Na]^+^ calcd for C_22_H_25_NO_4_SNa^+^ 422.4942; found 422.4946.

## Supporting Information Available

Complete list of optimization experiments, reaction schemes for the synthesis of substrates, crystallographic details, computational details, NMR spectra of new compounds (PDF)
FAIR data, including the primary NMR FID files, for compounds **1a**–**s**, **2a**–**r**, **int-7**, **int-8**, **int-9**, **int-12**, **5**, **6**, **7** (ZIP)

## Accession Codes

CCDC 2067578–2067580 contain the supplementary crystallographic data for this paper. These data can be obtained free of charge via www.ccdc.cam.ac.uk/data_request/cif, or by emailing data_request@ccdc.cam.ac.uk, or by contacting The Cambridge Crystallographic Data Centre, 12 Union Road, Cambridge CB2 1EZ, UK; fax: +44 1223 336033.
