# Supplementary material for: Gold(I)-Catalyzed Reactivity of Furan-ynes with N-Oxides: Synthesis of Substituted Dihydropyridinones and Pyranones
Source: J Org Chem. 2021 Jun 8;86(12):8295–307. doi: 10.1021/acs.joc.1c00746 (PMC8279485; doi:10.1021/acs.joc.1c00746)
Supplement: Supplementary file 2 — jo1c00746_si_002.pdf [file jo1c00746_si_002.pdf]

Gold(I)-catalyzed reactivity of furan-ynes with *N*-oxides: synthesis of substituted dihydropyridinones and pyranones

Stefano Nejrotti,* Francesco Marra, Emanuele Priola, Andrea Maranzana and Cristina Prandi*

Dipartimento di Chimica, Università degli Studi di Torino, via Pietro Giuria 7, *I*-10125 Torino, Italy.
stefano.nejrotti@unito.it, cristina.prandi@unito.it

Table of content

Tables S1 and S2	S1
Synthesis of the substrates: reaction schemes	S3
Crystallography	S5
NMR spectra of new compounds	S18
Computational details	S114

Table S1. Study of the reaction conditions – complete list of experiments. Entries 3, 8, 9,10 and 11 are not shown in the paper.

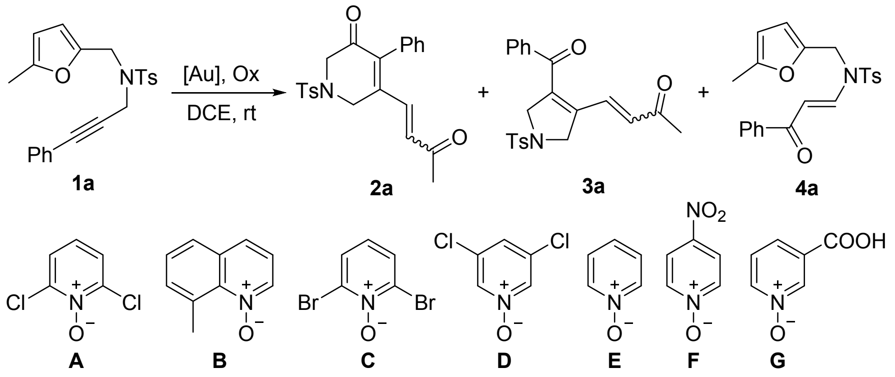

entry	[Au]	Ox	time	yield %[a]		
				2a (*E/Z*)	3a (*E/Z*)	4a
1	[(IPr)Au(NTf_2)]	**A**	6 h	81 (12/88)	–	–
2	[((2,4-$^tBu_2C_6H_3O)_3P$)AuCl] / $AgNTf_2$	**A**	6 h	15 (0/100)	6 (0/100)	11
3	[(Ph_3P)AuCl] / $AgNTf_2$	**A**	6 h	–	–	–
4	[((*p*-$CF_3Ph)_3P$)AuCl] / $AgNTf_2$	**A**	6 h	23 (0/100)	6 (0/100)	8
5	[(JohnPhos)AuCl] / $AgNTf_2$	**A**	6 h	77 (6/94)	–	–
6	[(JohnPhos)Au(NCMe)]SbF_6	**A**	6 h	40 (25/75)	–	–
7	[(^{t}BuXPhos)AuCl] / $AgNTf_2$	**A**	6 h	67 (10/90)	–	–
8	[(IPr)AuCl] / $AgNTf_2$	**A**	6 h	76 (7/93)	–	–
9	[(IMes)AuCl] / $AgNTf_2$	**A**	6 h	47 (13/87)	–	2
10	[(MorDalPhos)Au(NCMe)]SbF_6	**A**	6 h	54 (15/85)	–	–
11	[(($C_6F_5)_3P$)AuCl] / $AgNTf_2$	**A**	6 h	58 (8/92)	–	–
12	[(IPr)Au(NTf_2)]	**B**	6 h	–	30 (0/100)	60
13	[(IPr)Au(NTf_2)]	**C**	6 h	48 (10/90)	–	–
14	[(IPr)Au(NTf_2)]	**D**	6 h	56 (14/86)	17 (0/100)	27
15	[(IPr)Au(NTf_2)]	**E**	6 h	–	–	22
16	[(IPr)Au(NTf_2)]	**F**	6 h	68 (1/99)	–	–
17	[(IPr)Au(NTf_2)]	**G**	6 h	–	–	–
18	[(IPr)Au(NTf_2)]	**F**	20 h	96 (2/98)	–	–
19	[(MorDalPhos)Au(NCMe)]SbF_6	**B**	20 h	–	4 (0/100)	85[b]
20	[((2,4-$^tBu_2C_6H_3O)_3P$)AuCl] / $AgNTf_2$	**B**	20 h	–	13 (0/100)	29
21	[(JohnPhos)AuCl] / $AgNTf_2$	**B**	20 h	–	15 (0/100)	60
22	[(($C_6F_5)_3P$)AuCl] / $AgNTf_2$	**B**	20 h	–	4 (0/100)	11

Conditions: 0.1 mmol **1a**, 0.12 mmol Ox, 0.005 mmol Au(I) complex and, when specified, 0.005 mmol Ag salt, in 1.0 ml DCE. [a] Determined by ^1H NMR with *n*-heptane as internal standard. [b] 74% yield of isolated product.

Table S2. Optimization of the *E/Z* ratio in the synthesis of **2a** – complete list of experiments. Entries 2, 10 and 11 are not shown in the paper.

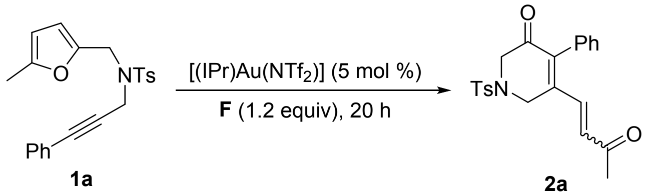

entry	T (°C)	solvent	additive	yield %[a] (*E/Z*)
1	80 °C	DCE	–	66 (23/77)
2[b]	110 °C	toluene	–	33 (52/48)
3	rt	DCE	TFA (2.0 equiv)	73 (16/84)
4	rt	DCE	*p*-TsOH (2.0 equiv)	43 (100/0)
5	rt	DCE	MsOH (2.0 equiv)	58 (100/0)
6	rt	$CHCl_3$	–	63 (3/97)
7	rt	HFIP	–	–
8	rt	DCE	*p*-TsOH (2.0 equiv)[c]	89 (11/89)
9	rt	DCE	MsOH (2.0 equiv)[c]	94 (17/83)
10	rt	DCE	TfOH (2.0 equiv)[c]	–
11[1]	rt	DCE	I_2 (one crystal)[c]	21 (86/14)
12	rt	DCE	MsOH (5.0 equiv)[c]	80[d] (100/0)

TFA = trifluoroacetic acid; HFIP = hexafluoroisopropanol. [a] Determined by ^{1}H NMR with *n*-heptane as internal standard. [b] 2 h instead of 20 h. [c] Added after 20 h, then stirred for one additional hour. [d] 72% yield of isolated product.

Synthesis of the substrates: reaction schemes

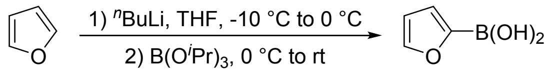

int-1

GP1

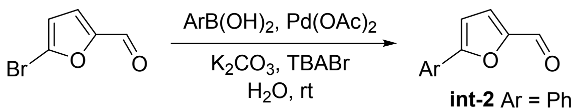

int-2 Ar = Ph
int-3 Ar = 2-thienyl
int-4 Ar = 2-furyl

GP2

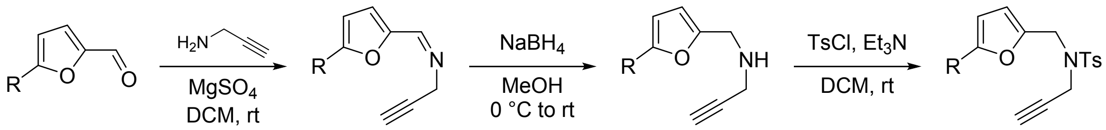

int-5 R = H
int-6 R = Me
int-7 R = Ph
int-8 R = 2-thienyl
int-9 R = 2-furyl

GP3A

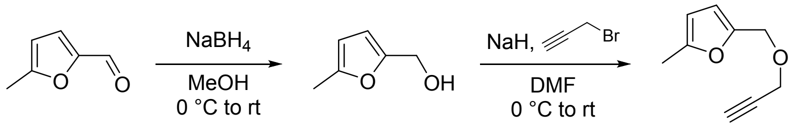

int-10

GP3B

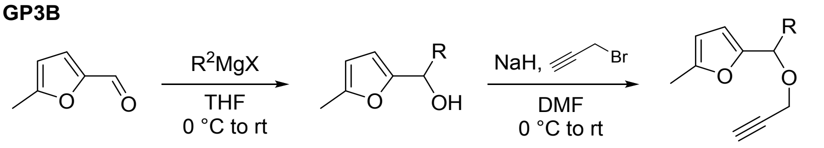

int-11 R = Et
int-12 R = *t*-Bu

GP4

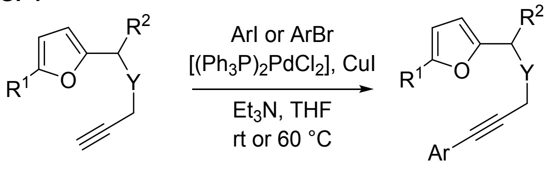

	R^1	R^2	Ar	Y
**1a**	Me	H	Ph	NTs
**1b**	Me	H	*p*-MeO(C_6H_4)	NTs
**1c**	Me	H	*p*-HO(C_6H_4)	NTs
**1d**	Me	H	*p*-Me(C_6H_4)	NTs
**1e**	Me	H	*p*-MeOCO(C_6H_4)	NTs
**1f**	Me	H	*p*-MeCO(C_6H_4)	NTs
**1g**	Me	H	*p*-F(C_6H_4)	NTs
**1h**	Me	H	2-thienyl	NTs
**1i**	Me	H	1-naphthyl	NTs
**1j**	Ph	H	Ph	NTs
**1k**	Ph	H	*p*-MeO(C_6H_4)	NTs
**1l**	2-thienyl	H	Ph	NTs
**1m**	2-furyl	H	Ph	NTs
**1n**	Me	H	Ph	O
**1o**	Me	H	*p*-MeO(C_6H_4)	O
**1p**	Me	H	*p*-MeOCO(C_6H_4)	O
**1q**	Me	Et	Ph	O
**1r**	Me	*t*-Bu	Ph	O
**1s**	H	H	Ph	NTs

Crystallography

Single-crystal data were collected with a Gemini R Ultra diffractometer with graphite-monochromated Mo-Kα radiation (λ = 0.71073 Å) for **3a** and **4a**, and Cu-Kα radiation (λ = 1.5406 Å) for **2a** by the ω-scan method. Cell parameters were retrieved with the CrysAlisPro software, and the same program was used to perform data reduction with corrections for Lorenz and polarizing effects. Scaling and absorption corrections were applied through the CrysAlisPro [CrysAlis PRO 1.171.38.46 (Rigaku OD, 2015).] multiscan technique. The structure for **2a** and **4a** was solved with Direct Methods.[2] All structures have been refined with full-matrix least-squares techniques on F^2 with SHELXL-14[3] using the program Olex2.[4] All non-hydrogen atoms were refined anisotropically. Hydrogen atoms were calculated and riding on the corresponding bonded atoms. The graphics of the crystal structure has been generated using Mercury 3.9.[5] CCDC codes 2067578-2067580 contain the supplementary crystallographic data for **2a**, **3a** and **4a**. These data can be obtained free of charge via http://www.ccdc.cam.ac. uk/conts/retrieving.html, or from the Cambridge Crystallographic Data Centre, 12 Union Road, Cambridge CB2 1EZ, UK; fax: (+44) 1223-336-033; or e-mail: deposit@ccdc.cam.ac.uk.

Figure S1: ORTEP plot of **2a** (50% probability).

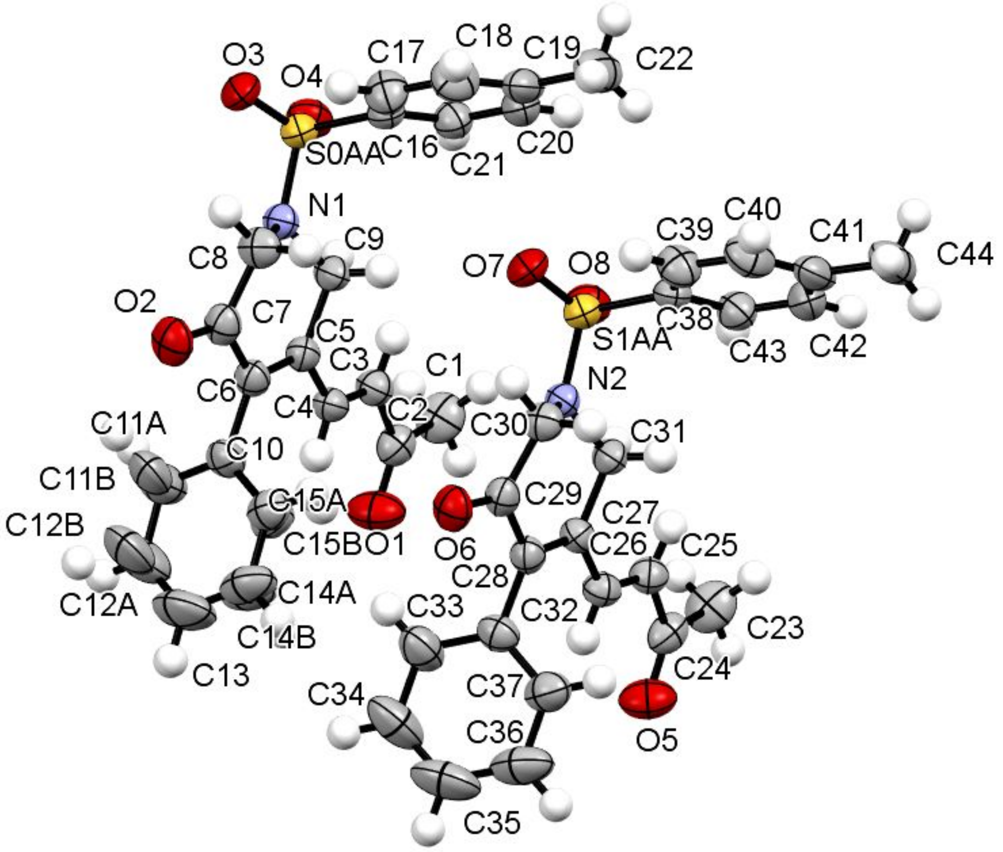

Table S3: Crystal data and structure refinement for **2a**.

Empirical formula	$C_{44}H_{42}N_2O_8S_2$
Formula weight	790.91
Temperature/K	298.00
Crystal system	triclinic
Space group	P-1
a/Å	11.8685(5)
b/Å	12.5043(5)
c/Å	14.4403(6)
α/°	75.343(4)
β/°	75.565(3)
γ/°	89.346(3)
Volume/Å^3	2004.79(14)
Z	2
ρ_{calc}g/cm^3	1.310
μ/mm^{-1}	1.666
F(000)	832.0
Crystal size/mm^3	0.14 × 0.1 × 0.09
Radiation	CuKα (λ = 1.54184)
2Θ range for data collection/°	7.318 to 135.082
Index ranges	-14 ≤ h ≤ 14, -14 ≤ k ≤ 14, -17 ≤ l ≤ 15
Reflections collected	21989
Independent reflections	6973 [R_{int} = 0.0451, R_{sigma} = 0.0365]
Data/restraints/parameters	6973/156/546
Goodness-of-fit on F^2	1.021
Final R indexes [I>=2σ (I)]	R_1 = 0.0469, wR_2 = 0.1168
Final R indexes [all data]	R_1 = 0.0726, wR_2 = 0.1339
Largest diff. peak/hole / e Å^{-3}	0.35/-0.34

Table S4: Bond Lengths for **2a**.

Atom	Atom	Length/Å	Atom	Atom	Length/Å
S0AA	O3	1.4225(18)	C41	C42	1.379(4)
S0AA	N1	1.6456(19)	C41	C44	1.513(4)
S0AA	O4	1.4275(18)	C21	C20	1.376(4)
S0AA	C16	1.754(2)	O5	C24	1.216(3)
S1AA	O7	1.4210(18)	C39	C40	1.373(4)
S1AA	O8	1.4288(18)	C32	C37	1.382(4)
S1AA	N2	1.6421(19)	C32	C33	1.382(4)
S1AA	C38	1.749(2)	C43	C42	1.374(4)
O2	C7	1.215(3)	C17	C18	1.376(4)
O6	C29	1.213(3)	C19	C18	1.382(4)
N1	C8	1.460(3)	C19	C20	1.382(4)
N1	C9	1.463(3)	C19	C22	1.510(4)
N2	C31	1.462(3)	C2	C3	1.483(3)
N2	C30	1.464(3)	C2	C1	1.492(4)
C16	C21	1.380(3)	C24	C25	1.478(3)
C16	C17	1.387(3)	C24	C23	1.495(4)
C38	C39	1.385(3)	C26	C25	1.325(3)
C38	C43	1.383(3)	C37	C36	1.383(4)
C4	C5	1.445(3)	C33	C34	1.388(4)
C4	C3	1.326(3)	C34	C35	1.365(6)
C29	C28	1.485(3)	C36	C35	1.374(6)
C29	C30	1.508(3)	C11A	C12A	1.384(17)
O1	C2	1.209(3)	C11A	C10	1.386(17)
C6	C7	1.475(3)	C12A	C13	1.353(17)
C6	C5	1.360(3)	C13	C14A	1.40(3)
C6	C10	1.494(3)	C13	C14B	1.25(6)
C7	C8	1.505(3)	C13	C12B	1.46(4)
C5	C9	1.510(3)	C14A	C15A	1.38(3)

C28	C27	1.357(3)	C15A	C10	1.37(2)
C28	C32	1.485(3)	C10	C15B	1.38(5)
C31	C27	1.503(3)	C10	C11B	1.38(4)
C27	C26	1.454(3)	C14B	C15B	1.38(6)
C41	C40	1.374(4)	C11B	C12B	1.42(4)

Table S5: Bond Angles for **2a**.

Atom	Atom	Atom	Angle/˚	Atom	Atom	Atom	Angle/˚
O3	S0AA	N1	106.21(10)	C40	C41	C42	118.4(3)
O3	S0AA	O4	119.68(12)	C40	C41	C44	121.2(3)
O3	S0AA	C16	108.44(11)	C42	C41	C44	120.5(3)
N1	S0AA	C16	107.67(11)	C20	C21	C16	119.5(2)
O4	S0AA	N1	105.79(10)	C40	C39	C38	119.4(3)
O4	S0AA	C16	108.49(11)	C37	C32	C28	119.9(2)
O7	S1AA	O8	119.52(12)	C37	C32	C33	118.9(2)
O7	S1AA	N2	106.25(10)	C33	C32	C28	121.2(2)
O7	S1AA	C38	108.97(11)	C42	C43	C38	119.4(2)
O8	S1AA	N2	105.99(10)	C18	C17	C16	119.4(3)
O8	S1AA	C38	108.19(11)	C18	C19	C20	118.1(3)
N2	S1AA	C38	107.30(10)	C18	C19	C22	121.1(3)
C8	N1	S0AA	116.82(15)	C20	C19	C22	120.8(3)
C8	N1	C9	113.72(19)	O1	C2	C3	121.0(2)
C9	N1	S0AA	115.75(14)	O1	C2	C1	122.2(2)
C31	N2	S1AA	116.08(14)	C3	C2	C1	116.7(2)
C31	N2	C30	113.10(19)	O5	C24	C25	121.1(2)
C30	N2	S1AA	117.53(14)	O5	C24	C23	122.2(2)
C21	C16	S0AA	119.70(19)	C25	C24	C23	116.7(2)
C21	C16	C17	120.0(2)	C17	C18	C19	121.5(3)
C17	C16	S0AA	120.29(19)	C25	C26	C27	126.5(2)
C39	C38	S1AA	120.3(2)	C21	C20	C19	121.5(3)

C43	C38	S1AA	119.77(19)	C4	C3	C2	121.8(2)
C43	C38	C39	119.9(2)	C39	C40	C41	121.5(3)
C3	C4	C5	127.6(2)	C32	C37	C36	120.9(3)
O6	C29	C28	122.7(2)	C43	C42	C41	121.4(3)
O6	C29	C30	119.9(2)	C32	C33	C34	120.1(3)
C28	C29	C30	117.40(19)	C26	C25	C24	122.4(2)
C7	C6	C10	118.0(2)	C35	C34	C33	120.0(4)
C5	C6	C7	120.0(2)	C35	C36	C37	119.2(4)
C5	C6	C10	121.9(2)	C34	C35	C36	120.7(3)
O2	C7	C6	122.3(2)	C12A	C11A	C10	121.4(13)
O2	C7	C8	120.4(2)	C13	C12A	C11A	119.2(13)
C6	C7	C8	117.21(19)	C12A	C13	C14A	119.8(12)
C4	C5	C9	117.03(19)	C14B	C13	C12B	120(3)
C6	C5	C4	121.1(2)	C15A	C14A	C13	120.9(18)
C6	C5	C9	121.9(2)	C10	C15A	C14A	119.3(17)
N1	C8	C7	110.22(19)	C11A	C10	C6	122.0(6)
C29	C28	C32	117.2(2)	C15A	C10	C6	118.5(8)
C27	C28	C29	119.4(2)	C15A	C10	C11A	119.3(10)
C27	C28	C32	123.4(2)	C15B	C10	C6	123.7(18)
N1	C9	C5	111.67(18)	C15B	C10	C11B	119(2)
N2	C31	C27	111.93(18)	C11B	C10	C6	117.3(15)
N2	C30	C29	109.28(19)	C13	C14B	C15B	119(4)
C28	C27	C31	121.9(2)	C14B	C15B	C10	124(4)
C28	C27	C26	122.0(2)	C10	C11B	C12B	115(3)
C26	C27	C31	116.10(19)	C11B	C12B	C13	122(3)

Figure S2: ORTEP plot of **3a** (50% probability).

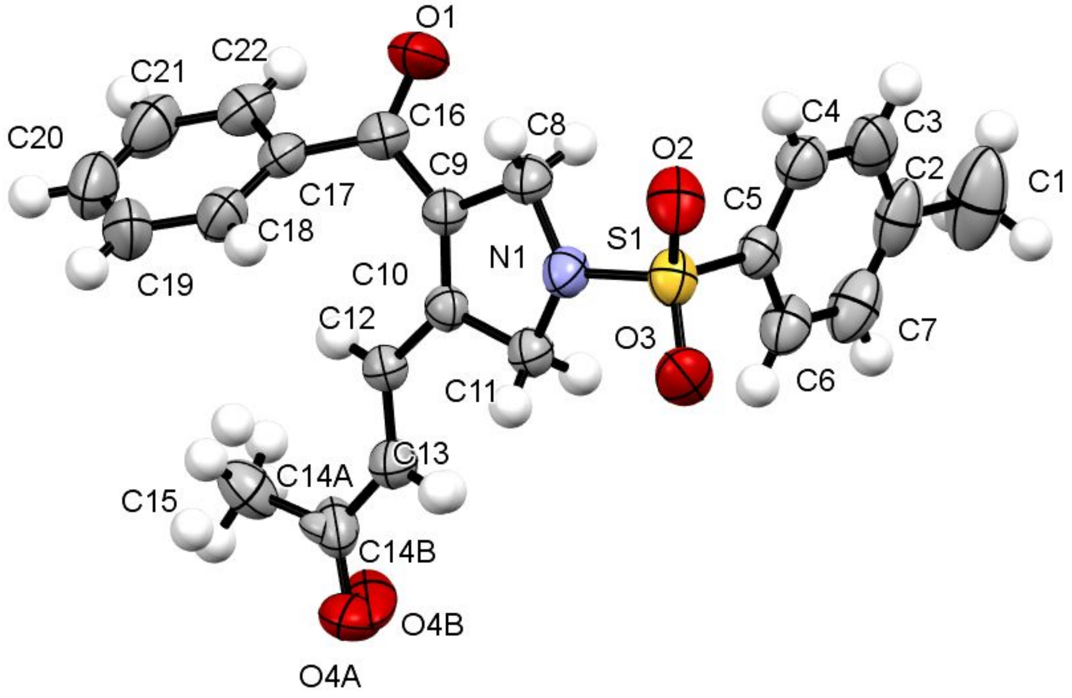

Table S6: Crystal data and structure refinement for **3a**.

Empirical formula	$C_{22}H_{21}NO_4S$
Formula weight	395.46
Temperature/K	298.0
Crystal system	monoclinic
Space group	$P2_1/c$
a/Å	13.4049(11)
b/Å	8.0643(7)
c/Å	18.8272(14)
α/°	90
β/°	91.556(7)
γ/°	90
Volume/Å^3	2034.5(3)
Z	4
ρ_{calc}g/cm^3	1.291
μ/mm^{-1}	0.186
F(000)	832.0

Crystal size/mm^3	0.15 × 0.11 × 0.1
Radiation	MoKα (λ = 0.71073)
2Θ range for data collection/°	7.268 to 58.978
Index ranges	-12 ≤ h ≤ 16, -9 ≤ k ≤ 10, -18 ≤ l ≤ 25
Reflections collected	12193
Independent reflections	4670 [R_{int} = 0.0394, R_{sigma} = 0.0529]
Data/restraints/parameters	4670/5/274
Goodness-of-fit on F^2	1.028
Final R indexes [I>=2σ (I)]	R_1 = 0.0566, wR_2 = 0.1141
Final R indexes [all data]	R_1 = 0.1019, wR_2 = 0.1368
Largest diff. peak/hole / e Å^{-3}	0.17/-0.26

Table S7: Bond Lengths for **3a**.

**Atom**	**Atom**	**Length/Å**	**Atom**	**Atom**	**Length/Å**
S1	C5	1.754(2)	C17	C22	1.390(3)
S1	N1	1.6302(19)	O4A	C14A	1.253(8)
S1	O3	1.4333(16)	C14A	C13	1.481(7)
S1	O2	1.4241(17)	C14A	C15	1.485(7)
C9	C10	1.346(3)	C13	C14B	1.472(12)
C9	C16	1.487(3)	C15	C14B	1.487(12)
C9	C8	1.501(3)	C18	C19	1.380(4)
C10	C12	1.451(3)	C4	C3	1.387(4)
C10	C11	1.505(3)	C2	C7	1.370(4)
C16	C17	1.478(3)	C2	C3	1.392(4)
C16	O1	1.222(3)	C2	C1	1.520(4)
C5	C4	1.375(3)	C22	C21	1.384(4)
C5	C6	1.388(3)	C6	C7	1.369(4)
N1	C8	1.473(3)	C20	C21	1.368(4)
N1	C11	1.465(3)	C20	C19	1.374(4)
C12	C13	1.325(3)	O4B	C14B	1.246(16)

C17	C18	1.382(3)

Table S8: Bond Angles for **3a**.

Atom	Atom	Atom	Angle/˚	Atom	Atom	Atom	Angle/˚
N1	S1	C5	108.14(9)	C18	C17	C22	119.1(2)
O3	S1	C5	108.41(10)	C22	C17	C16	119.2(2)
O3	S1	N1	105.36(10)	O4A	C14A	C13	117.5(6)
O2	S1	C5	107.60(11)	O4A	C14A	C15	120.9(6)
O2	S1	N1	106.02(10)	C13	C14A	C15	119.9(6)
O2	S1	O3	120.75(10)	C12	C13	C14A	125.8(4)
C10	C9	C16	130.2(2)	C12	C13	C14B	126.2(5)
C10	C9	C8	111.67(17)	N1	C11	C10	102.87(16)
C16	C9	C8	117.64(19)	C19	C18	C17	120.7(2)
C9	C10	C12	130.00(18)	C5	C4	C3	119.5(2)
C9	C10	C11	110.01(18)	C7	C2	C3	118.3(3)
C12	C10	C11	119.90(17)	C7	C2	C1	121.6(3)
C17	C16	C9	121.9(2)	C3	C2	C1	120.0(3)
O1	C16	C9	117.2(2)	C21	C22	C17	120.0(3)
O1	C16	C17	120.8(2)	C7	C6	C5	119.3(3)
C4	C5	S1	120.82(18)	C6	C7	C2	122.0(3)
C4	C5	C6	120.2(2)	C4	C3	C2	120.7(3)
C6	C5	S1	118.96(19)	C21	C20	C19	120.8(3)
C8	N1	S1	120.47(15)	C20	C21	C22	119.9(3)
C11	N1	S1	120.25(14)	C20	C19	C18	119.5(3)
C11	N1	C8	110.59(16)	C13	C14B	C15	120.4(9)
N1	C8	C9	101.80(17)	O4B	C14B	C13	115.2(16)
C13	C12	C10	123.72(19)	O4B	C14B	C15	112.9(19)
C18	C17	C16	121.7(2)				

Figure S3: ORTEP plot of **4a** (50% probability).

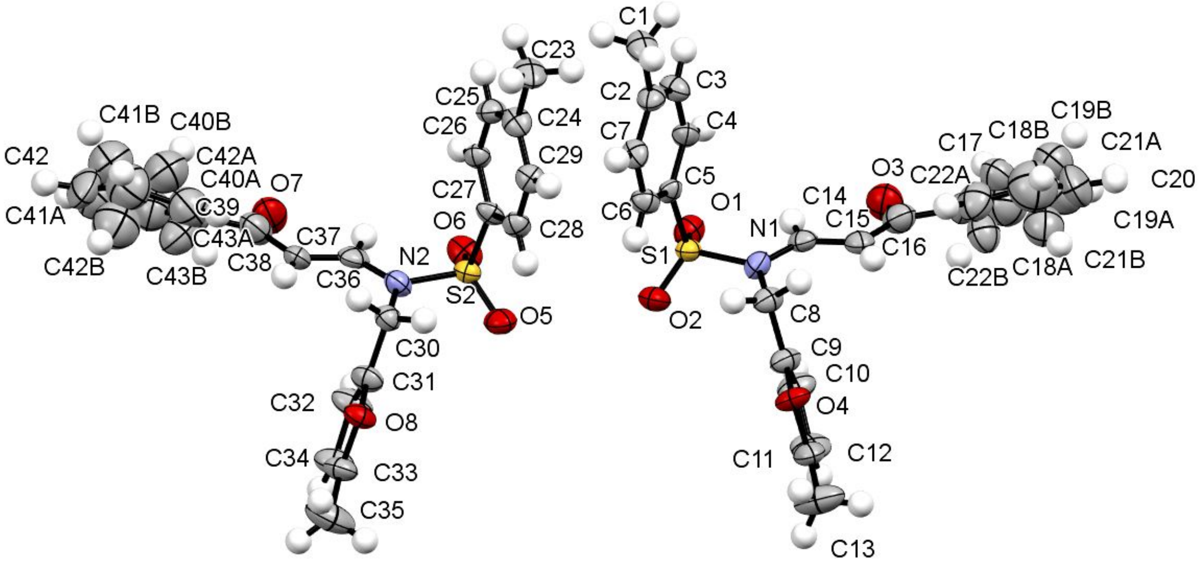

Table S9: Crystal data and structure refinement for **4a**.

Empirical formula	$C_{22}H_{21}NO_4S$
Formula weight	395.46
Temperature/K	298.00
Crystal system	monoclinic
Space group	Pc
a/Å	20.340(3)
b/Å	6.0668(6)
c/Å	16.5499(19)
α/°	90
β/°	101.749(12)
γ/°	90
Volume/Å^3	1999.4(4)
Z	4
ρ_{calc}g/cm^3	1.314
μ/mm^{-1}	0.190
F(000)	832.0
Crystal size/mm^3	0.21 × 0.2 × 0.08
Radiation	MoKα (λ = 0.71073)

2Θ range for data collection/°	6.716 to 52.734
Index ranges	-25 ≤ h ≤ 24, -6 ≤ k ≤ 7, -20 ≤ l ≤ 20
Reflections collected	19483
Independent reflections	7842 [R_{int} = 0.0682, R_{sigma} = 0.0871]
Data/restraints/parameters	7842/118/583
Goodness-of-fit on F^2	1.085
Final R indexes [I>=2σ (I)]	R_1 = 0.0997, wR_2 = 0.2540
Final R indexes [all data]	R_1 = 0.1352, wR_2 = 0.2854
Largest diff. peak/hole / e Å^{-3}	0.70/-0.33
Flack parameter	0.02(8)

Table S10: Bond Lengths for **4a**.

Atom	Atom	Length/Å	Atom	Atom	Length/Å
S1	O1	1.397(8)	C24	C23	1.498(15)
S1	C5	1.749(10)	N2	C30	1.460(13)
S1	O2	1.419(9)	N2	C36	1.343(15)
S1	N1	1.663(10)	C30	C31	1.512(15)
C5	C6	1.399(14)	C36	C37	1.324(17)
C5	C4	1.387(14)	C34	C35	1.479(19)
O4	C12	1.414(14)	C34	C33	1.326(18)
O4	C9	1.382(13)	C31	C32	1.308(16)
C6	C7	1.396(15)	C32	C33	1.438(18)
C3	C2	1.386(16)	C38	C37	1.457(19)
C3	C4	1.391(16)	C38	C39	1.50(2)
N1	C14	1.376(15)	C38	O7	1.255(17)
N1	C8	1.481(14)	C39	C43A	1.33(3)
C7	C2	1.383(16)	C39	C40A	1.39(3)
C2	C1	1.521(15)	C39	C43B	1.35(3)
C12	C11	1.337(19)	C39	C40B	1.35(3)
C12	C13	1.43(2)	C43A	C42A	1.49(4)

C11	C10	1.447(18)	C42	C41A	1.27(3)
C14	C15	1.334(16)	C42	C42A	1.36(3)
C8	C9	1.494(14)	C42	C42B	1.38(4)
C9	C10	1.349(16)	C42	C41B	1.25(3)
C16	C15	1.421(18)	C17	C18A	1.39(3)
C16	O3	1.237(16)	C17	C22A	1.26(3)
C16	C17	1.54(2)	C17	C22B	1.31(3)
S2	O6	1.405(8)	C17	C18B	1.36(3)
S2	O5	1.411(9)	C41A	C40A	1.38(4)
S2	C27	1.761(10)	C18A	C19A	1.35(3)
S2	N2	1.678(10)	C20	C21A	1.46(3)
C28	C27	1.362(14)	C20	C19A	1.34(3)
C28	C29	1.407(15)	C20	C21B	1.49(4)
C27	C26	1.390(14)	C20	C19B	1.32(3)
O8	C34	1.400(14)	C21A	C22A	1.43(4)
O8	C31	1.367(13)	C21B	C22B	1.44(4)
C26	C25	1.386(15)	C18B	C19B	1.35(4)
C24	C25	1.381(16)	C43B	C42B	1.50(4)
C24	C29	1.380(16)	C40B	C41B	1.36(4)

Table S11: Bond Angles for **4a**.

Atom	Atom	Atom	Angle/˚	Atom	Atom	Atom	Angle/˚
O1	S1	C5	108.9(5)	C24	C29	C28	119.3(10)
O1	S1	O2	121.4(5)	C30	N2	S2	118.1(7)
O1	S1	N1	105.9(5)	C36	N2	S2	120.4(8)
O2	S1	C5	107.9(5)	C36	N2	C30	121.2(10)
O2	S1	N1	106.9(5)	N2	C30	C31	110.1(8)
N1	S1	C5	104.7(5)	C37	C36	N2	129.2(12)
C6	C5	S1	119.6(8)	O8	C34	C35	115.8(12)
C4	C5	S1	118.9(8)	C33	C34	O8	108.6(11)

C4	C5	C6	121.2(10)	C33	C34	C35	135.5(13)
C9	O4	C12	107.2(9)	O8	C31	C30	115.1(9)
C7	C6	C5	118.2(10)	C32	C31	O8	111.7(10)
C2	C3	C4	120.8(10)	C32	C31	C30	133.1(11)
C14	N1	S1	120.3(8)	C31	C32	C33	106.1(11)
C14	N1	C8	122.3(10)	C37	C38	C39	118.5(14)
C8	N1	S1	117.2(8)	O7	C38	C37	120.3(13)
C2	C7	C6	121.2(10)	O7	C38	C39	121.2(14)
C3	C2	C1	120.2(11)	C36	C37	C38	120.6(12)
C7	C2	C3	119.4(10)	C43A	C39	C38	119.3(18)
C7	C2	C1	120.5(11)	C43A	C39	C40A	126(2)
O4	C12	C13	116.3(13)	C40A	C39	C38	115.1(19)
C11	C12	O4	107.1(11)	C43B	C39	C38	120.5(19)
C11	C12	C13	136.6(13)	C40B	C39	C38	117(2)
C5	C4	C3	119.0(10)	C40B	C39	C43B	121(3)
C12	C11	C10	110.0(11)	C39	C43A	C42A	113(3)
C15	C14	N1	127.1(11)	C34	C33	C32	107.8(12)
N1	C8	C9	109.1(9)	C41A	C42	C42A	117(3)
O4	C9	C8	115.2(10)	C41B	C42	C42B	117(3)
C10	C9	O4	110.8(10)	C18A	C17	C16	117.8(18)
C10	C9	C8	133.9(11)	C22A	C17	C16	122.2(17)
C15	C16	C17	117.4(13)	C22A	C17	C18A	120(2)
O3	C16	C15	123.4(13)	C22B	C17	C16	122.0(18)
O3	C16	C17	119.0(13)	C22B	C17	C18B	121(2)
C14	C15	C16	118.3(12)	C18B	C17	C16	117.1(19)
C9	C10	C11	104.8(11)	C42	C41A	C40A	128(3)
O6	S2	O5	120.8(6)	C19A	C18A	C17	123(3)
O6	S2	C27	109.5(5)	C42	C42A	C43A	121(3)
O6	S2	N2	106.2(5)	C19A	C20	C21A	124(2)
O5	S2	C27	107.6(5)	C19B	C20	C21B	124(3)

O5	S2	N2	107.6(5)	C22A	C21A	C20	112(3)
N2	S2	C27	103.9(5)	C41A	C40A	C39	113(3)
C27	C28	C29	119.6(10)	C20	C19A	C18A	116(3)
C28	C27	S2	119.7(8)	C17	C22A	C21A	124(3)
C28	C27	C26	122.0(10)	C22B	C21B	C20	111(3)
C26	C27	S2	118.2(8)	C17	C22B	C21B	123(3)
C31	O8	C34	105.7(9)	C19B	C18B	C17	122(3)
C25	C26	C27	117.8(10)	C20	C19B	C18B	119(3)
C25	C24	C23	121.0(11)	C39	C43B	C42B	117(3)
C29	C24	C25	120.1(10)	C42	C42B	C43B	117(3)
C29	C24	C23	119.0(11)	C39	C40B	C41B	115(3)
C24	C25	C26	121.3(10)	C42	C41B	C40B	130(4)

References

1. Brambilla, E.; Pirovano, V.; Giannangeli, M.; Abbiati, G.; Caselli, A.; Rossi, E. *Org. Chem. Front.* **2019,** *6*, 3078-3084.
2. Sheldrick, G. M. *Acta Crystallog. Sect. A: Found. Crystallogr.* **2008,** *64*, 112-122.
3. Sheldrick, G. M. *Acta Crystallogr. Sect. A: Found. Adv.* **2015,** *71*, 3-8.
4. Dolomanov, O. V.; Bourhis, L. J.; Gildea, R. J.; Howard, J. A.; Puschmann, H. *J. Appl. Crystallogr.* **2009,** *42*, 339-341.
5. Macrae, C. F.; Edgington, P. R.; McCabe, P.; Pidcock, E.; Shields, G. P.; Taylor, R.; Towler, M.; Streek, J. *J. Appl. Crystallogr.* **2006,** *39*, 453-457.

NMR Spectra of new compounds

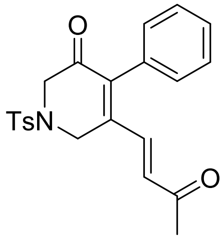

2a
^{1}H NMR (600 MHz)

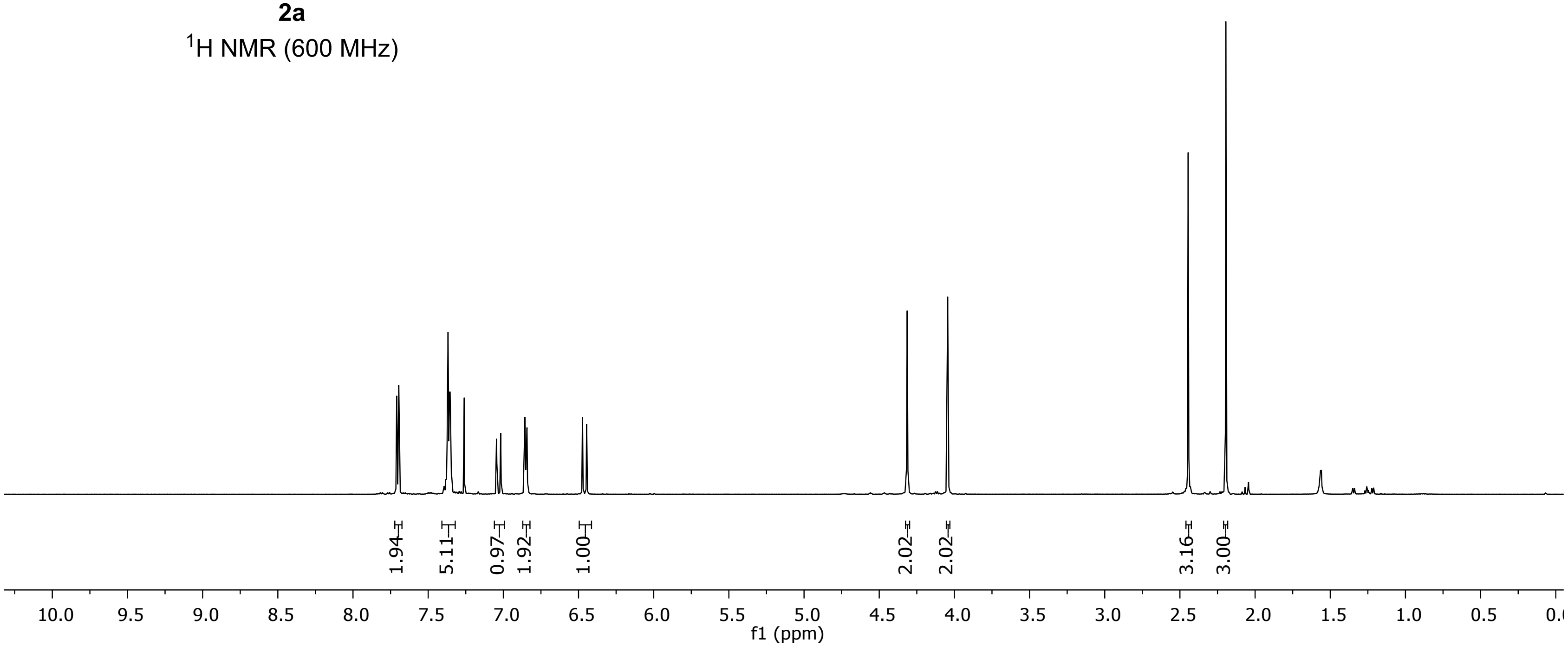

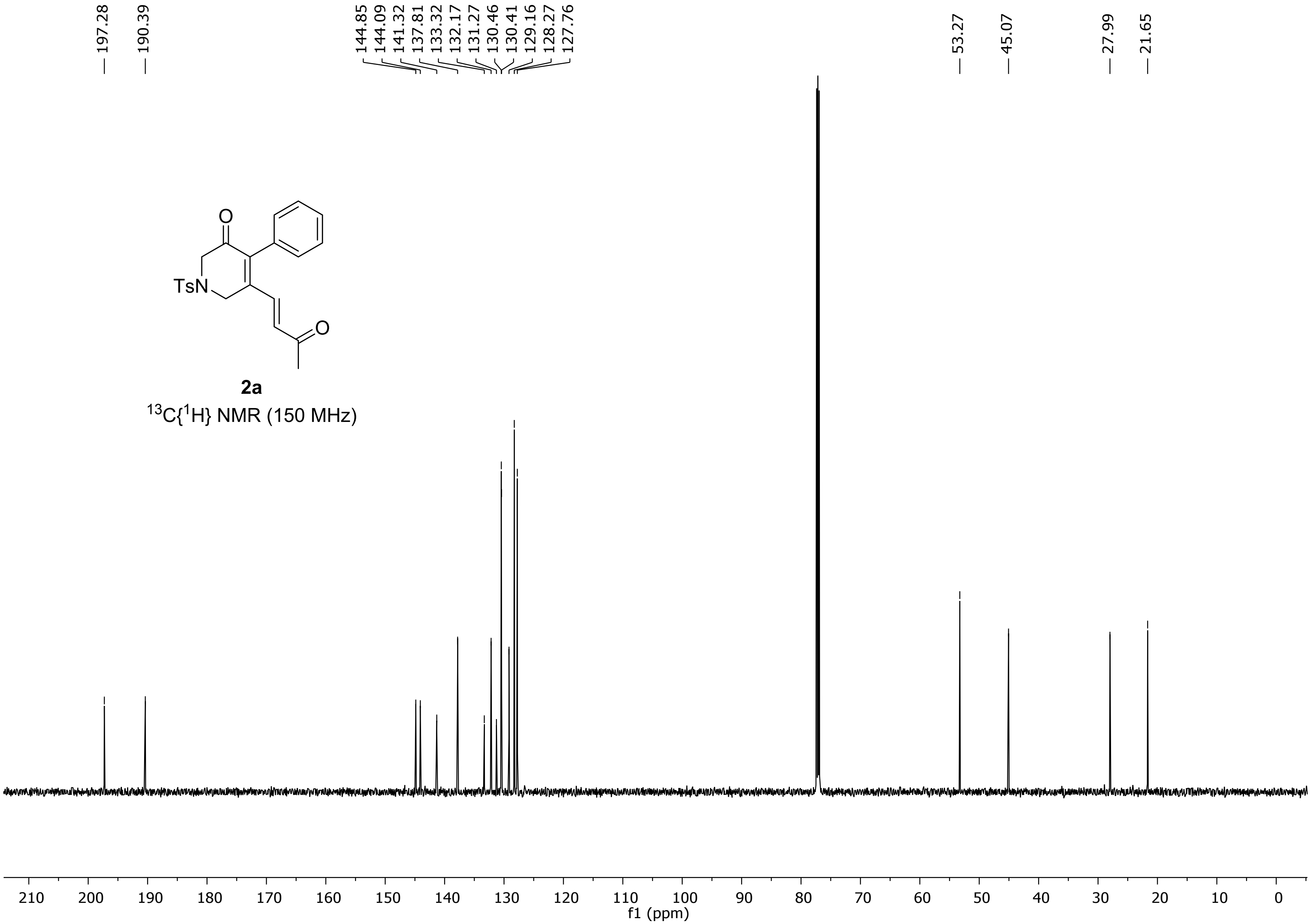
197.28
190.39
144.85
144.09
141.32
137.81
133.32
132.17
131.27
130.46
130.41
129.16
128.27
127.76
53.27
45.07
27.99
21.65
O
TsN
O
2a
$^{13}C\{^1H\}$ NMR (150 MHz)
210
200
190
180
170
160
150
140
130
120
110
100
90
80
70
60
50
40
30
20
10
0
f1 (ppm)

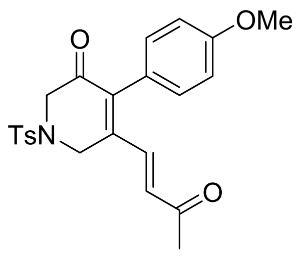

2b

^{1}H NMR (600 MHz)

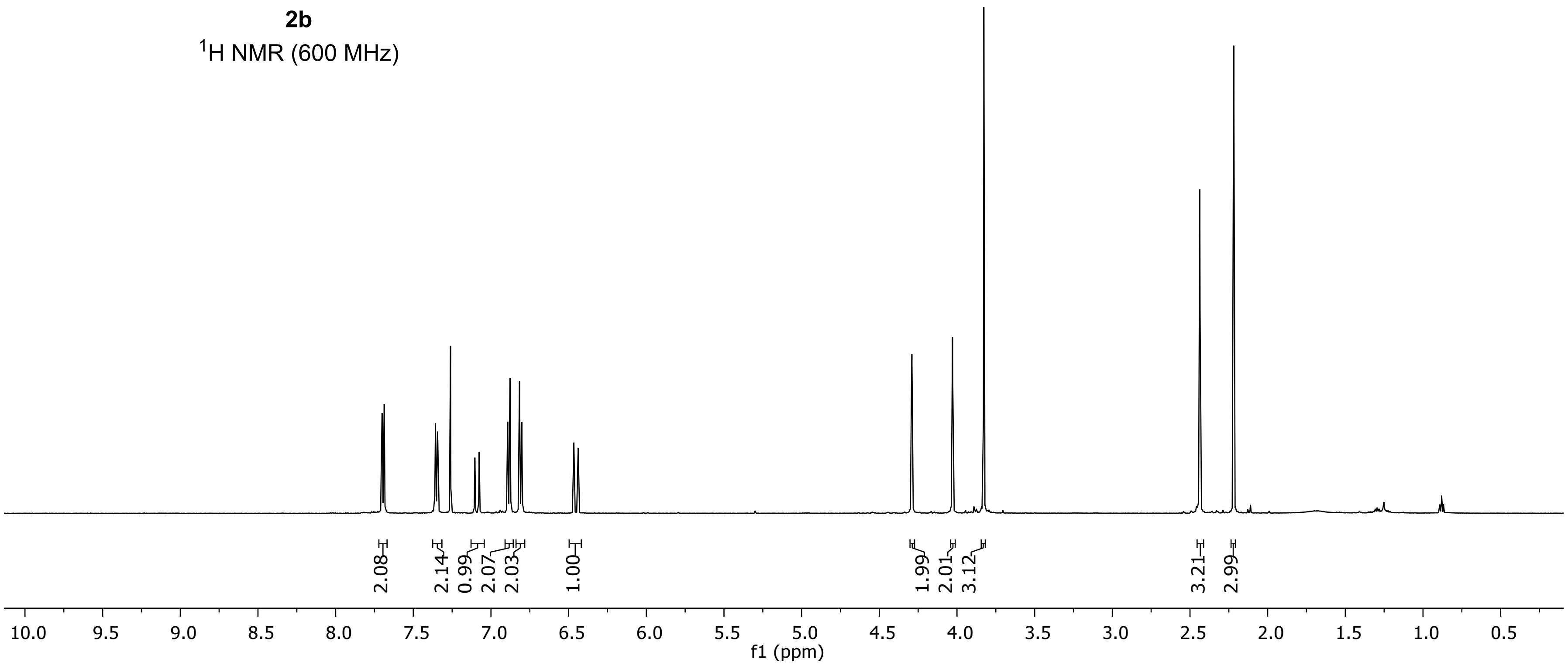

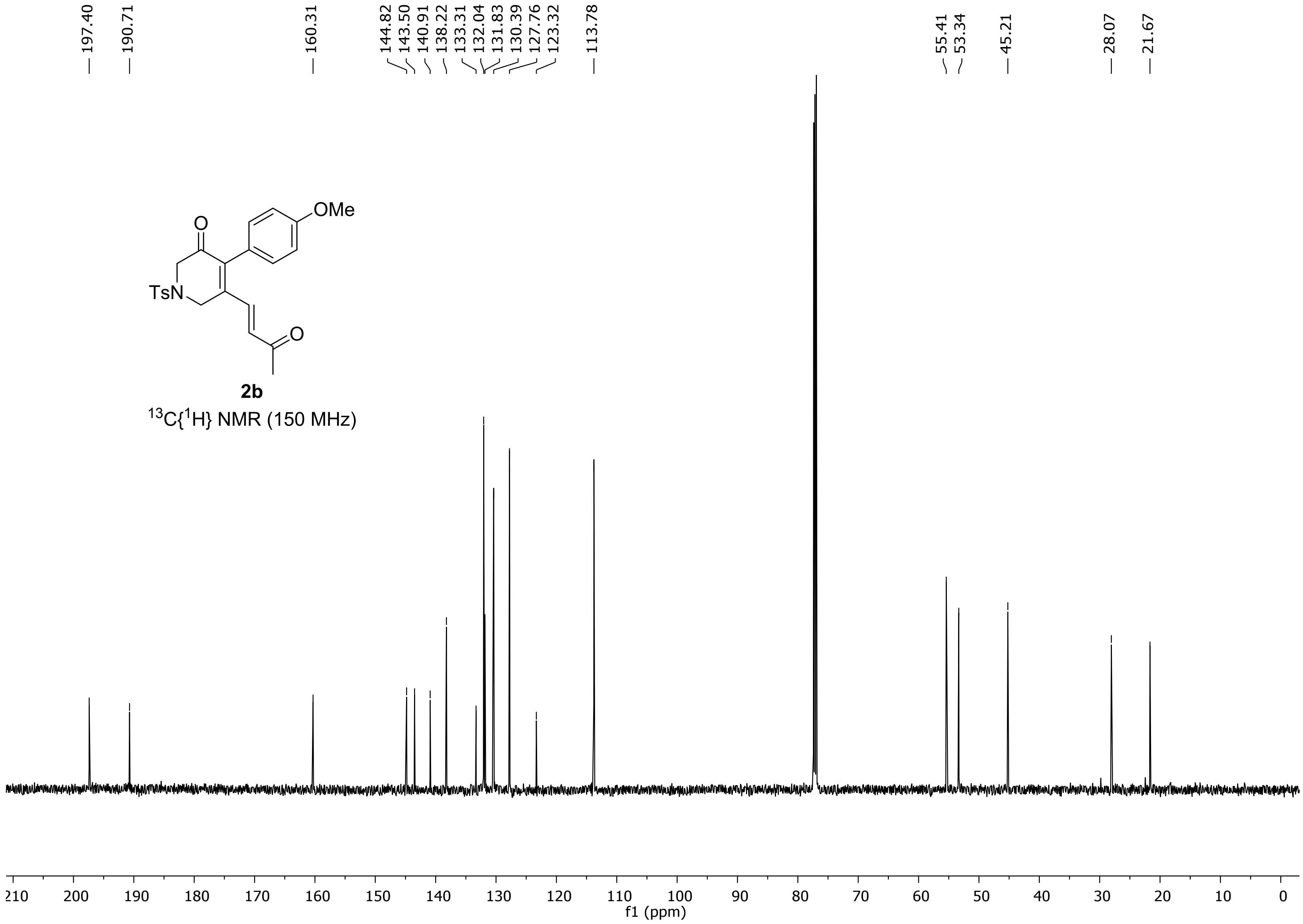
197.40
190.71
160.31
144.82
143.50
140.91
138.22
133.31
132.04
131.83
130.39
127.76
123.32
113.78
55.41
53.34
45.21
28.07
21.67
OMe
O
TsN
O
2b
13C{1H} NMR (150 MHz)
210 200 190 180 170 160 150 140 130 120 110 100 90 80 70 60 50 40 30 20 10 0
f1 (ppm)

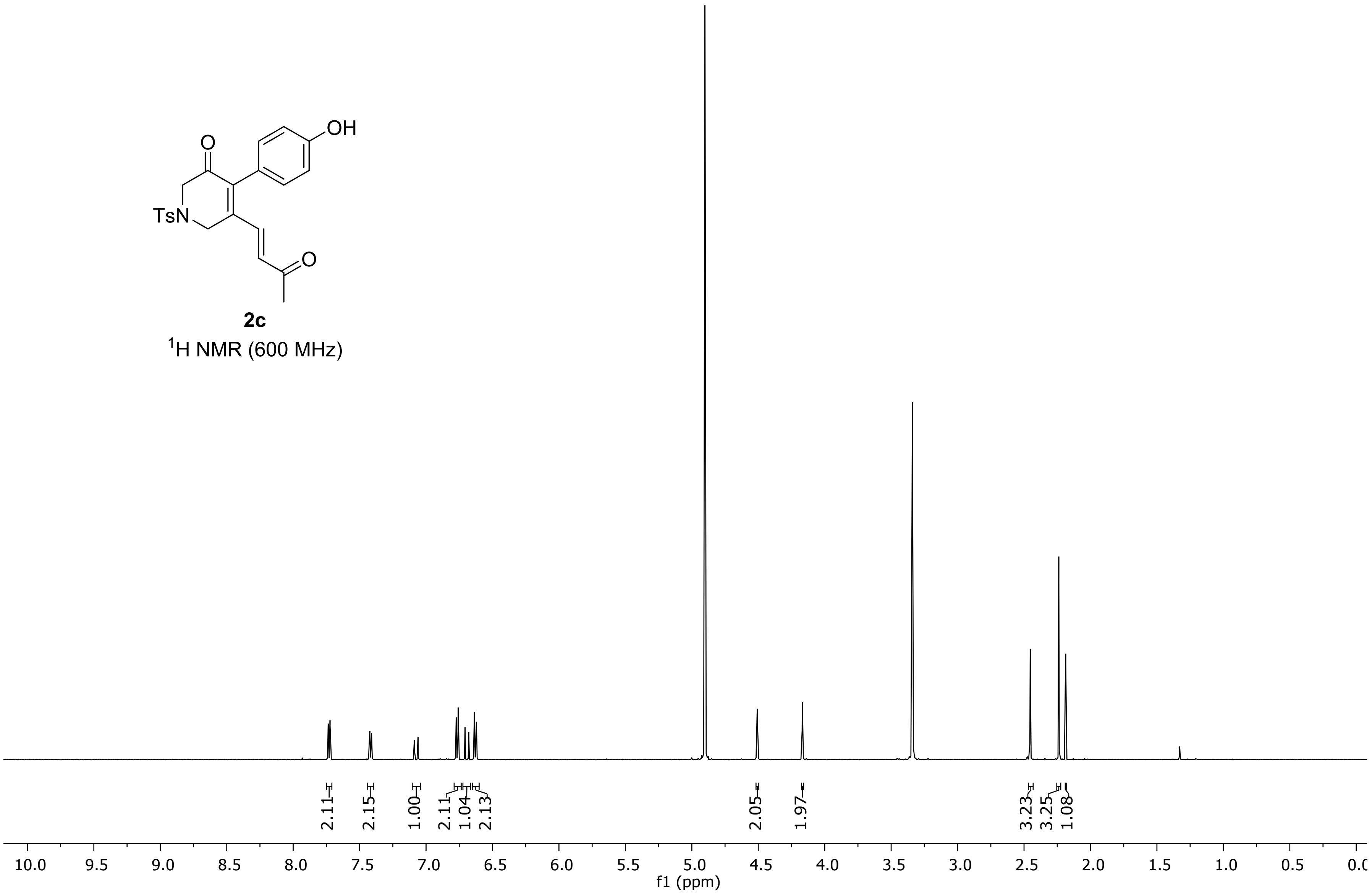
OH
O
TsN
O
2c
1H NMR (600 MHz)
2.11
2.15
1.00
2.11
1.04
2.13
2.05
1.97
3.23
3.25
1.08
10.0 9.5 9.0 8.5 8.0 7.5 7.0 6.5 6.0 5.5 5.0 4.5 4.0 3.5 3.0 2.5 2.0 1.5 1.0 0.5 0.0
f1 (ppm)

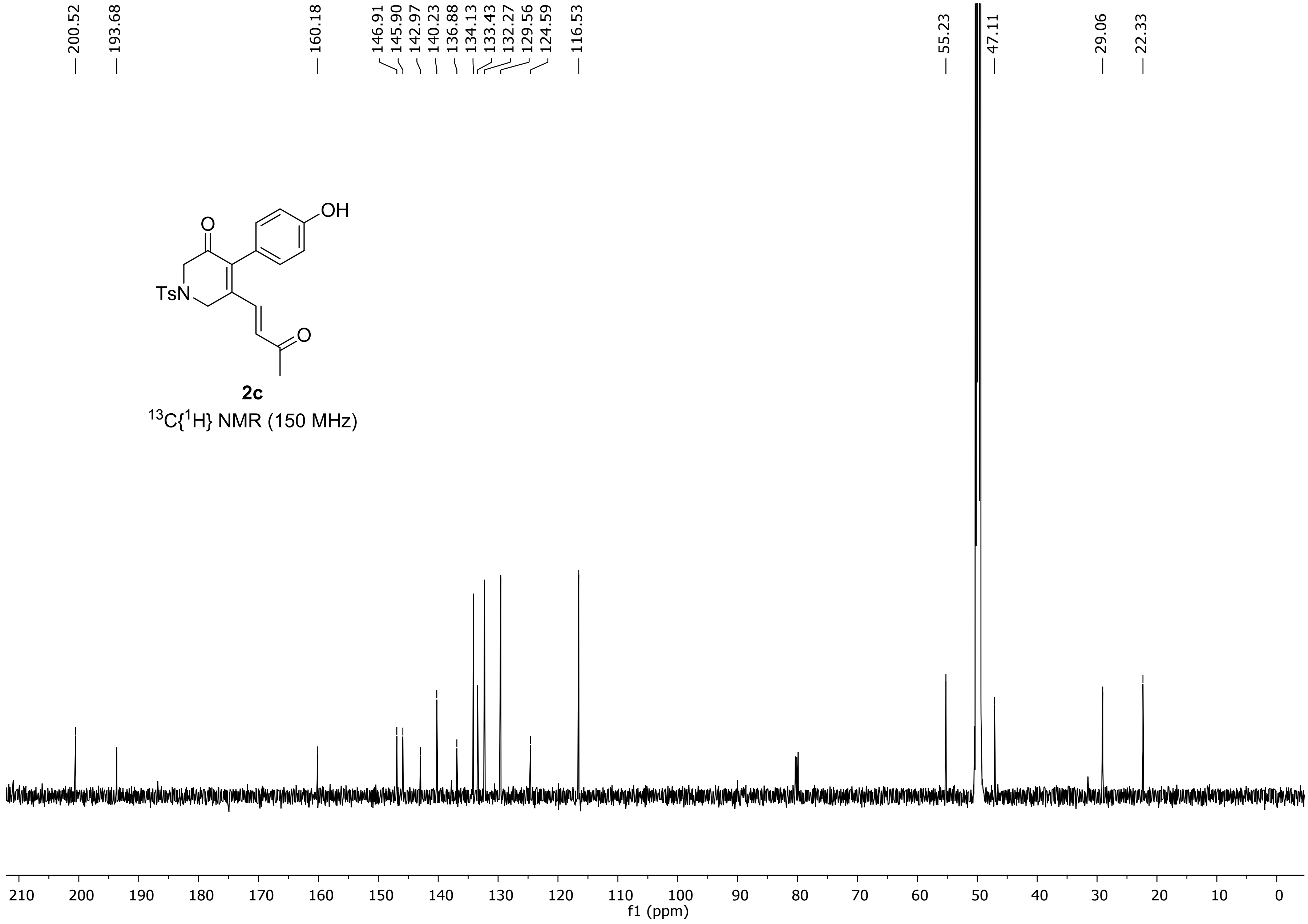

2c

$^{13}C\{^{1}H\}$ NMR (150 MHz)

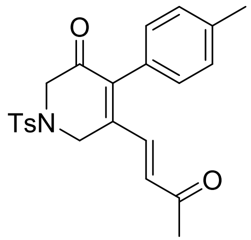

2d

^{1}H NMR (600 MHz)

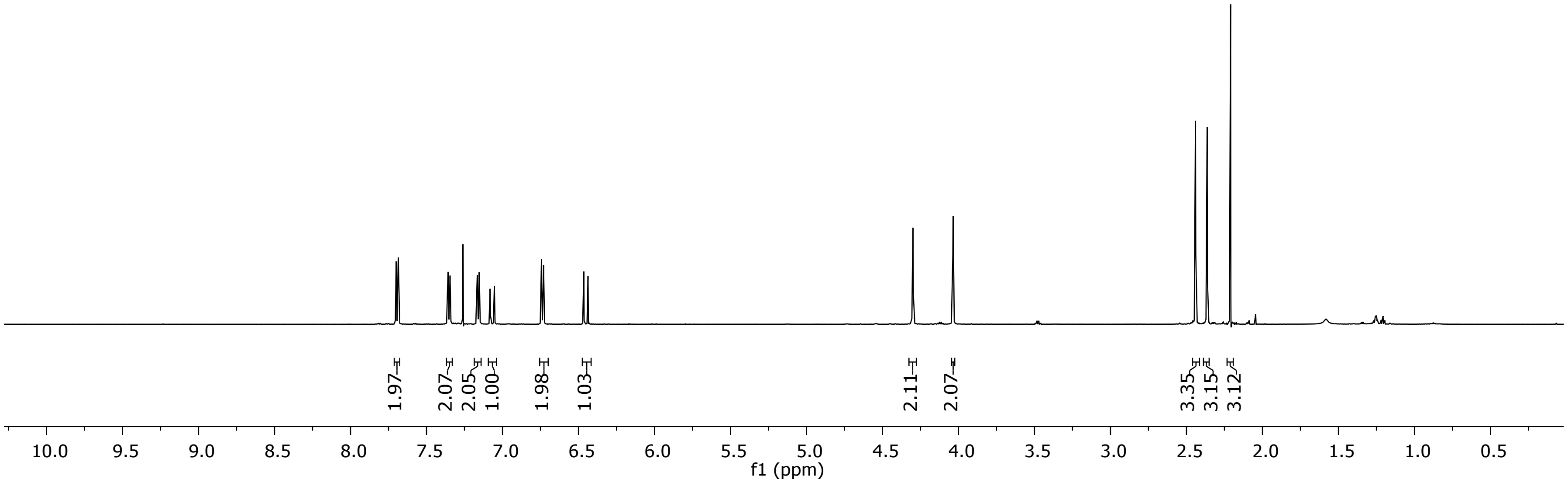

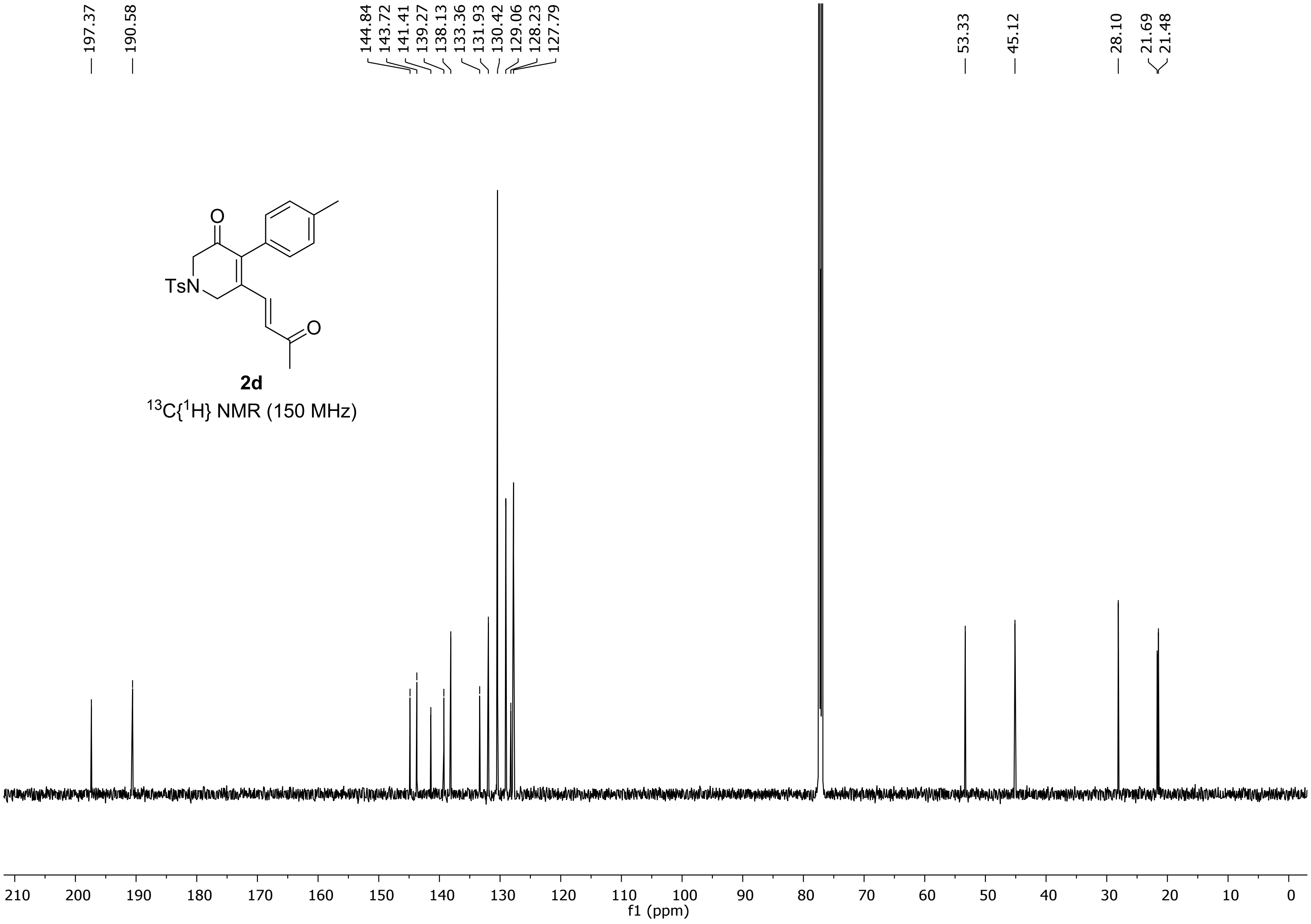
197.37
190.58
144.84
143.72
141.41
139.27
138.13
133.36
131.93
130.42
129.06
128.23
127.79
53.33
45.12
28.10
21.69
21.48
O
TsN
O
2d
$^{13}C\{^{1}H\}$ NMR (150 MHz)
210
200
190
180
170
160
150
140
130
120
110
100
90
80
70
60
50
40
30
20
10
0
f1 (ppm)

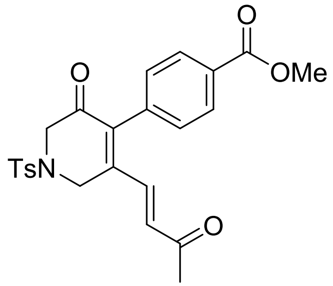

2e

^{1}H NMR (600 MHz)

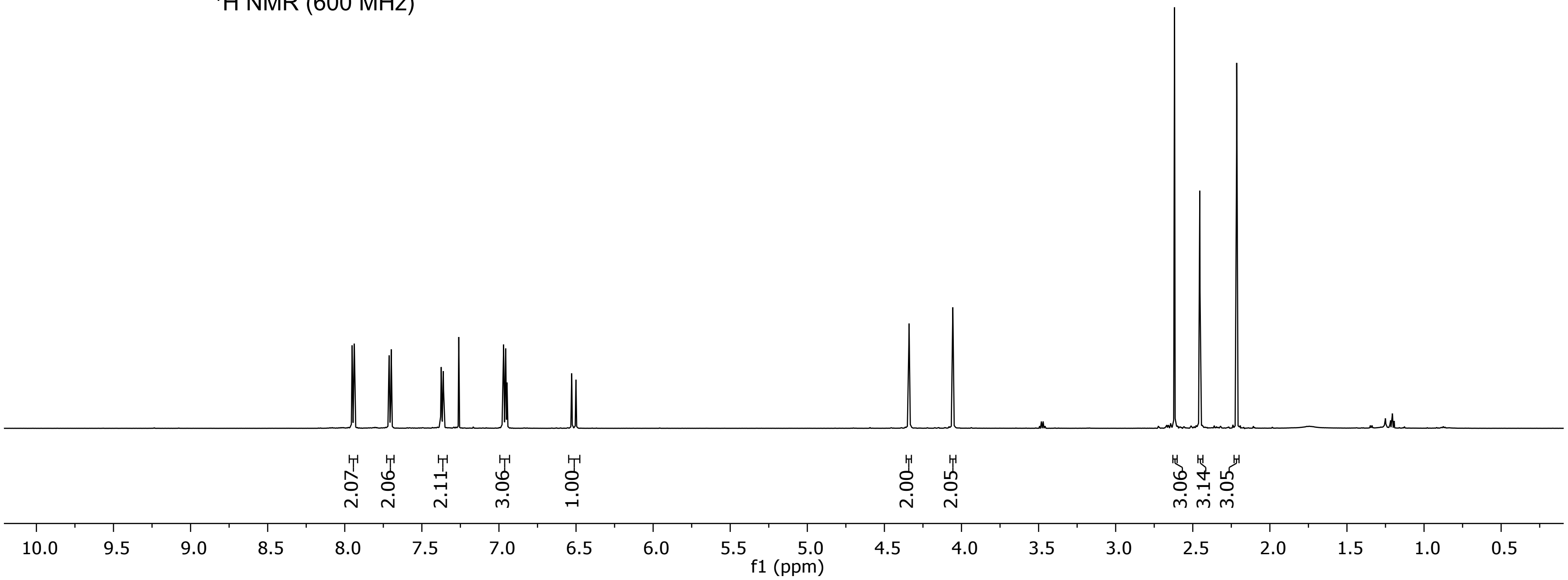

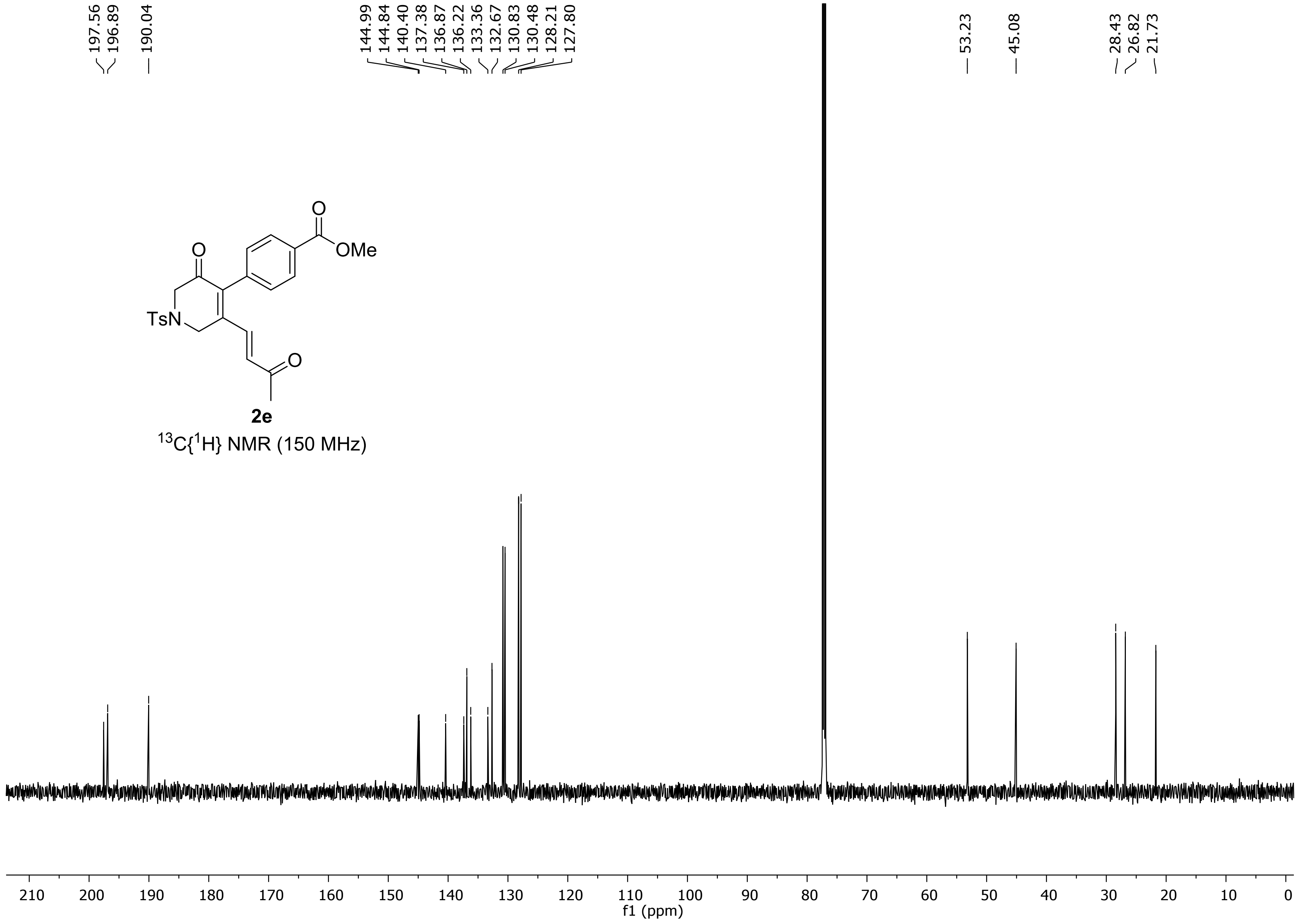
197.56
196.89
190.04
144.99
144.84
140.40
137.38
136.87
136.22
133.36
132.67
130.83
130.48
128.21
127.80
53.23
45.08
28.43
26.82
21.73
O
OMe
O
TsN
O
2e
$^{13}C\{^{1}H\}$ NMR (150 MHz)
210 200 190 180 170 160 150 140 130 120 110 100 90 80 70 60 50 40 30 20 10 0
f1 (ppm)

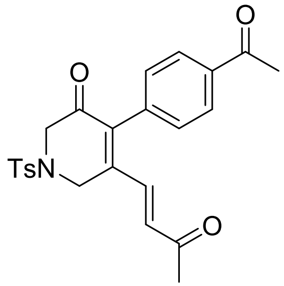

2f

^{1}H NMR (600 MHz)

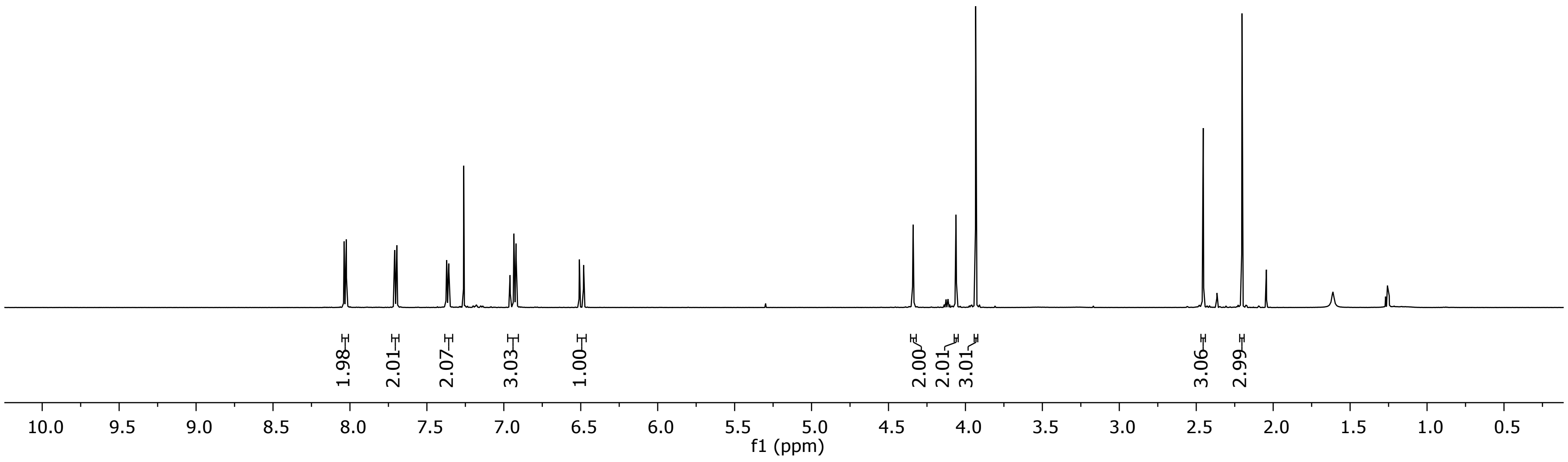

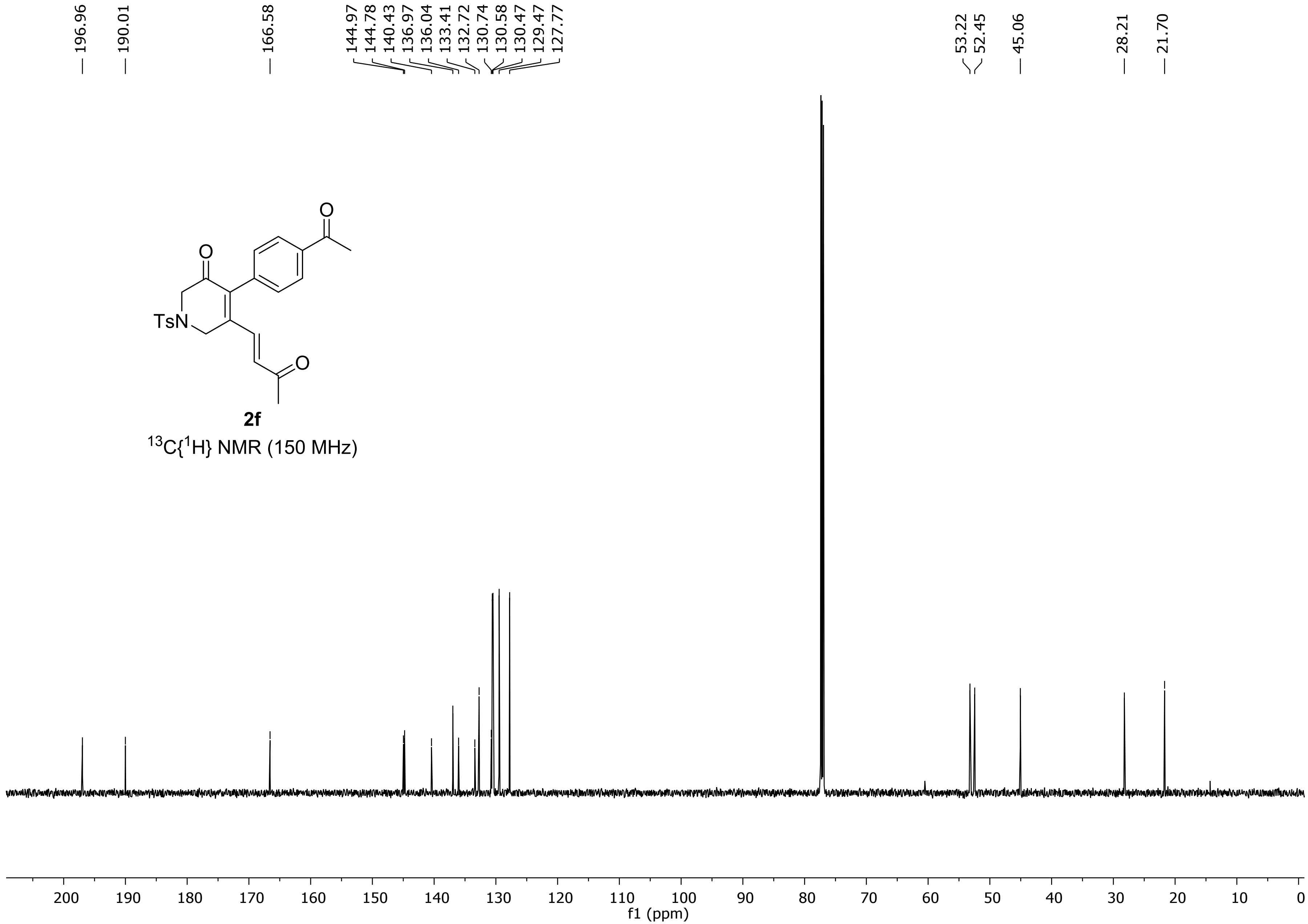
196.96
190.01
166.58
144.97
144.78
140.43
136.97
136.04
133.41
132.72
130.74
130.58
130.47
129.47
127.77
53.22
52.45
45.06
28.21
21.70
TsN
O
2f
$^{13}C\{^{1}H\}$ NMR (150 MHz)
200 190 180 170 160 150 140 130 120 110 100 90 80 70 60 50 40 30 20 10 0
f1 (ppm)

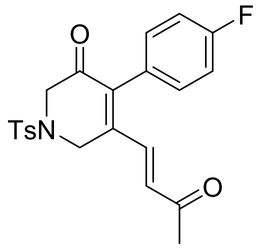

2g

^{1}H NMR (600 MHz)

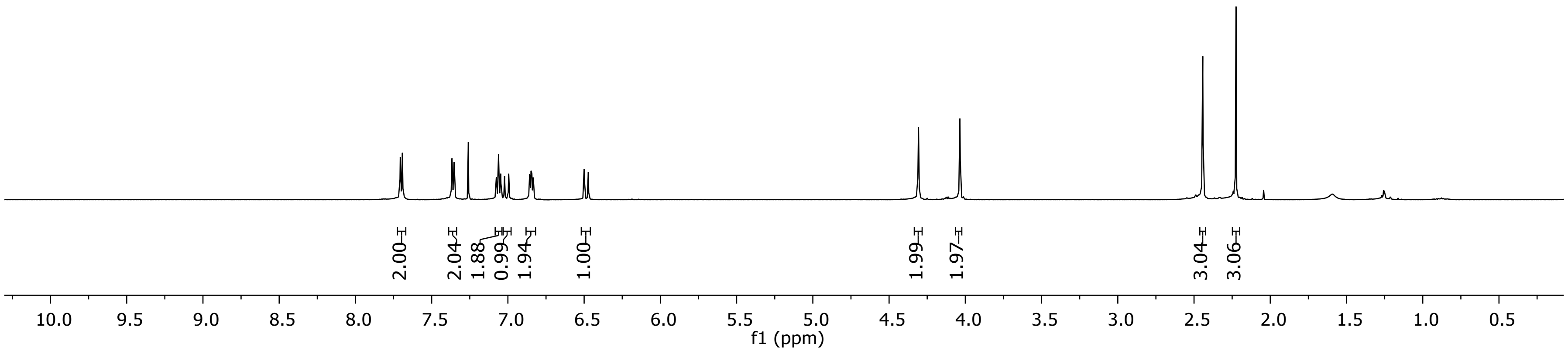

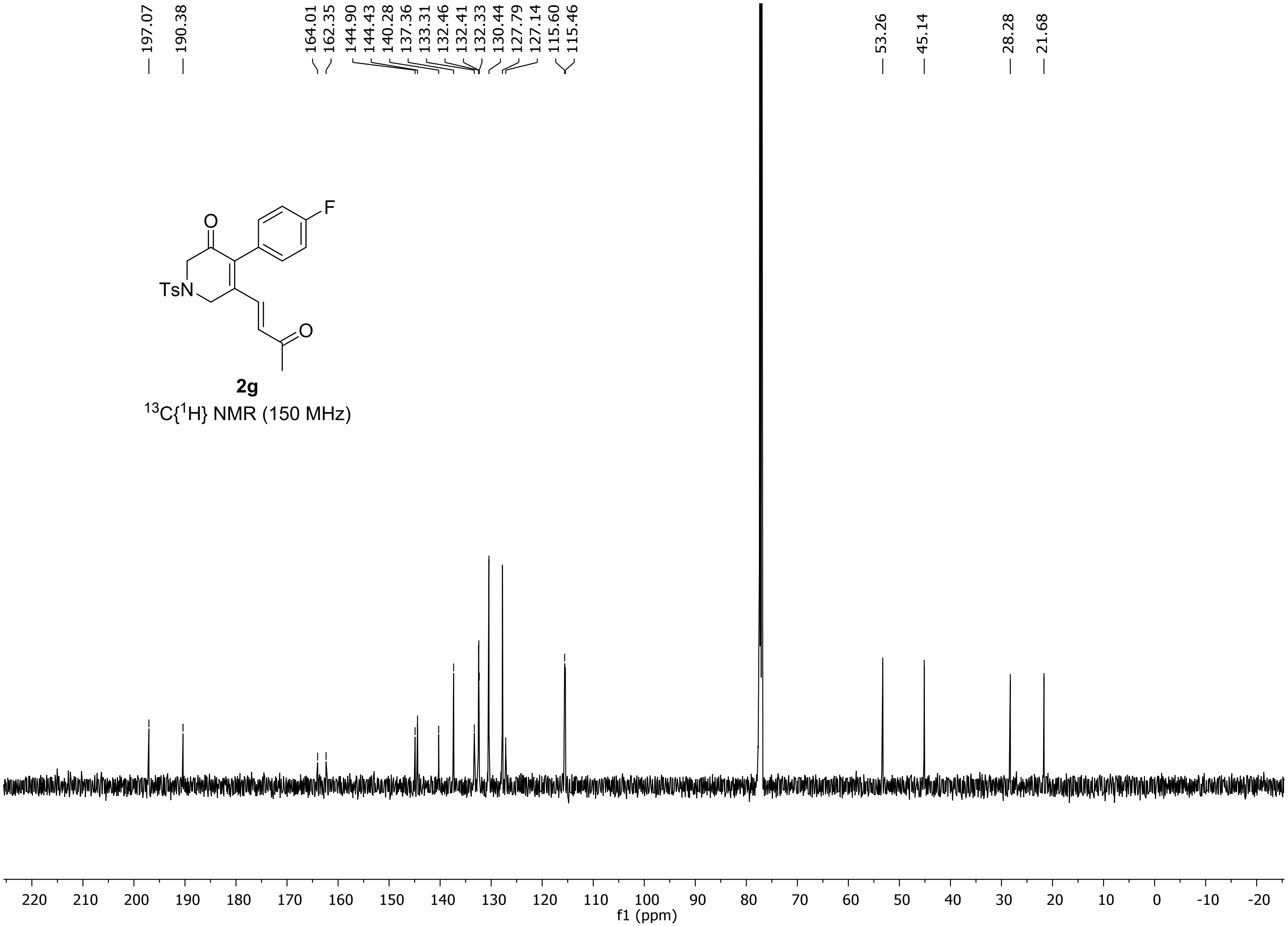
197.07
190.38
164.01
162.35
144.90
144.43
140.28
137.36
133.31
132.46
132.41
132.33
130.44
127.79
127.14
115.60
115.46
53.26
45.14
28.28
21.68
O
F
TsN
O
2g
$^{13}C\{^{1}H\}$ NMR (150 MHz)
220 210 200 190 180 170 160 150 140 130 120 110 100 90 80 70 60 50 40 30 20 10 0 -10 -20
f1 (ppm)

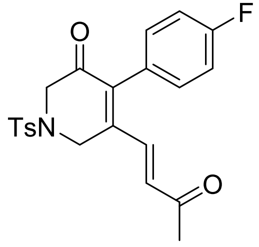

2g

^{19}F NMR (564 MHz)

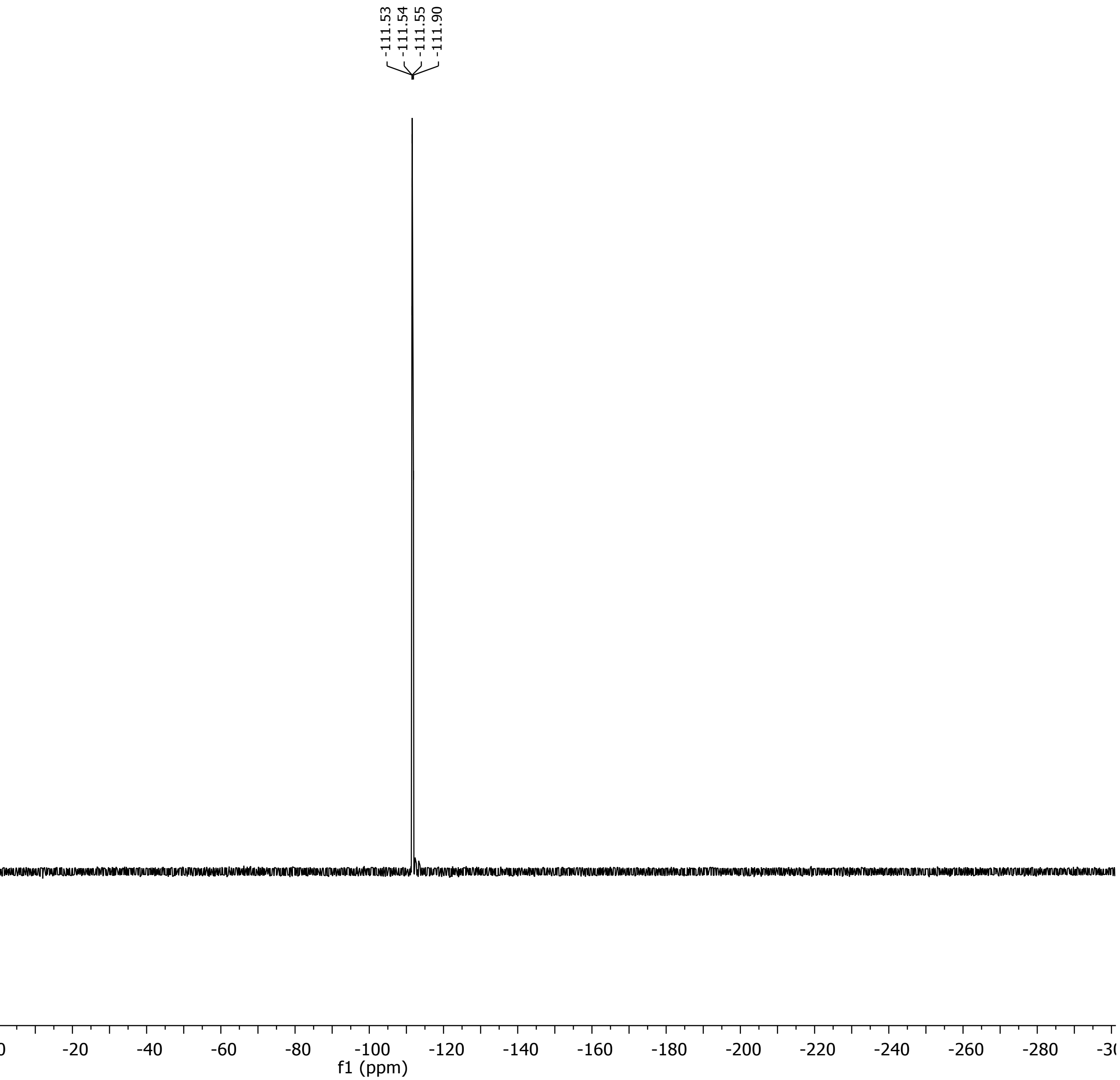

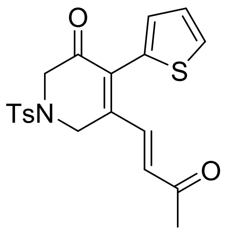

2h

^{1}H NMR (600 MHz)

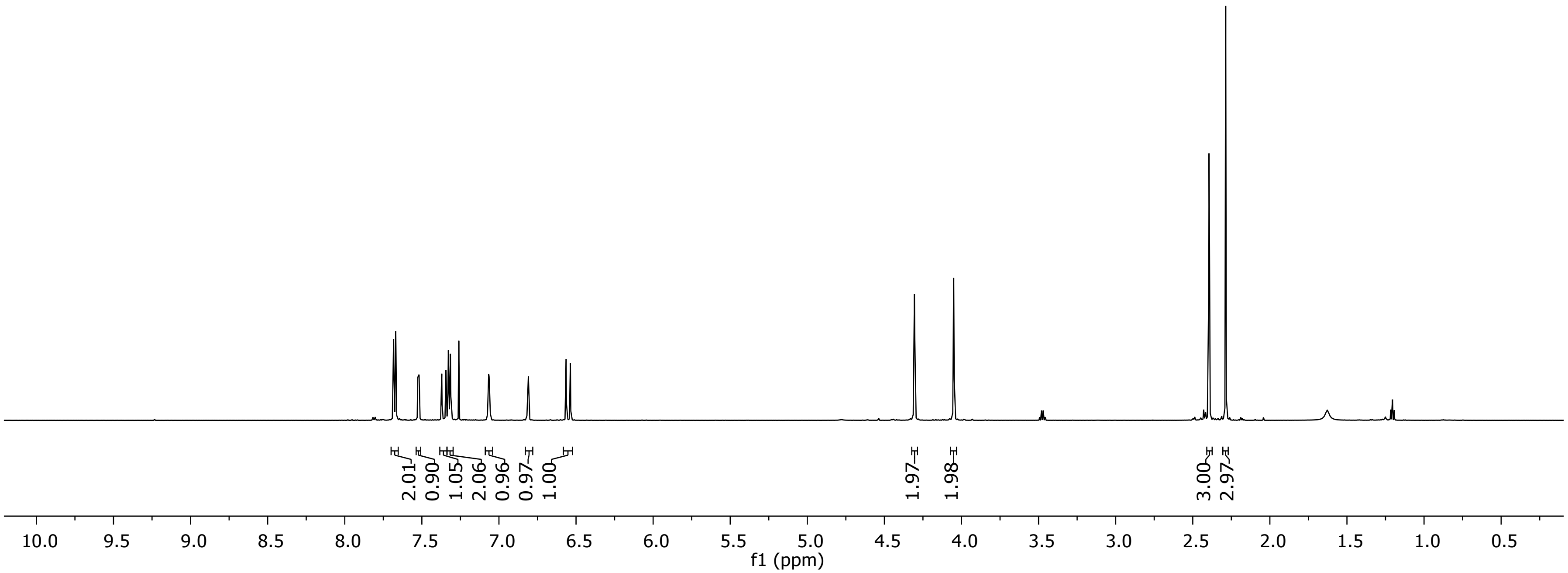

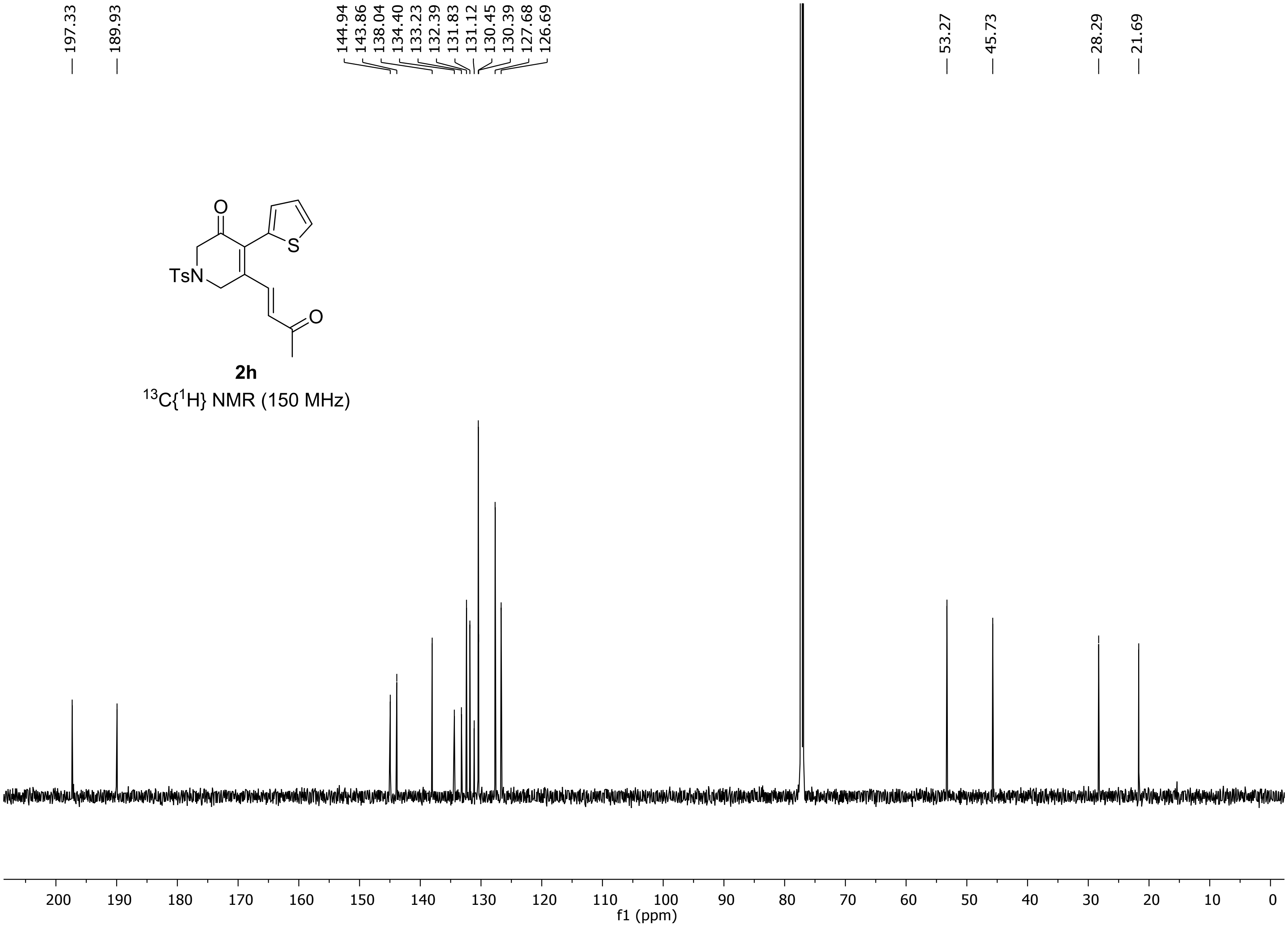

2h

$^{13}C\{^{1}H\}$ NMR (150 MHz)

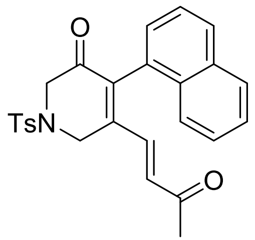

2i

^{1}H NMR (600 MHz)

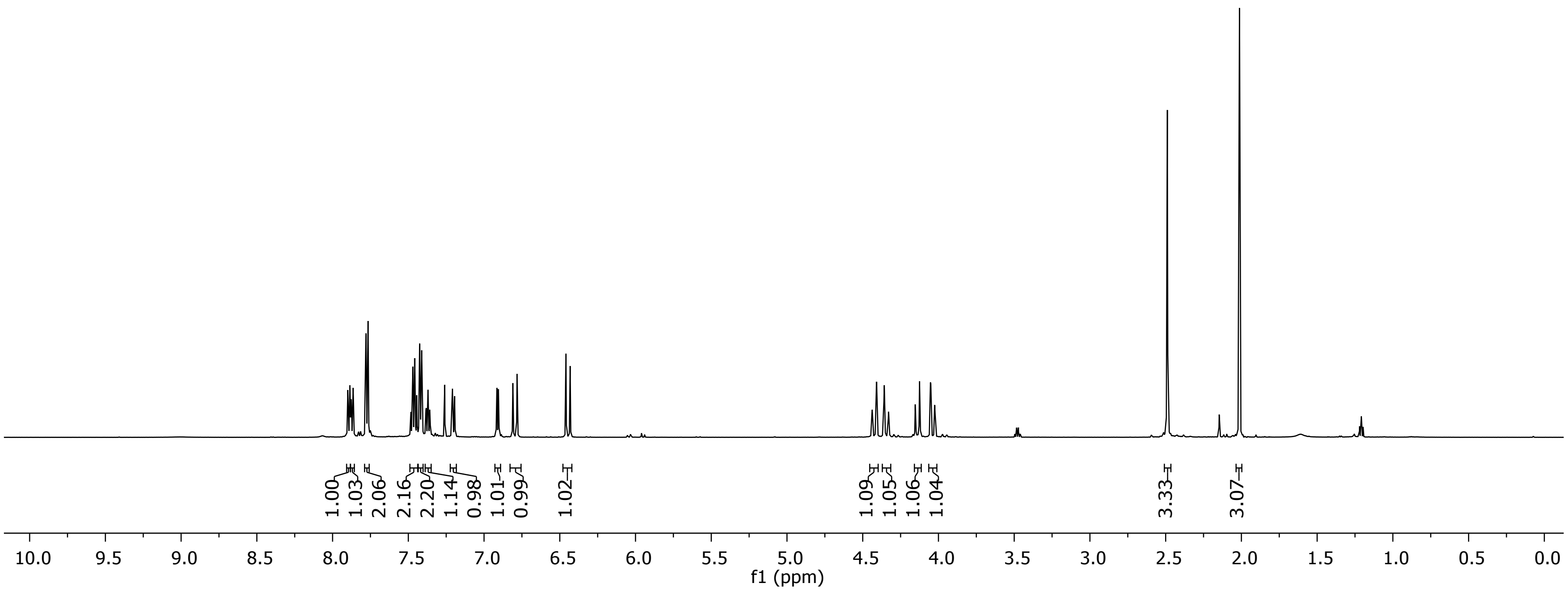

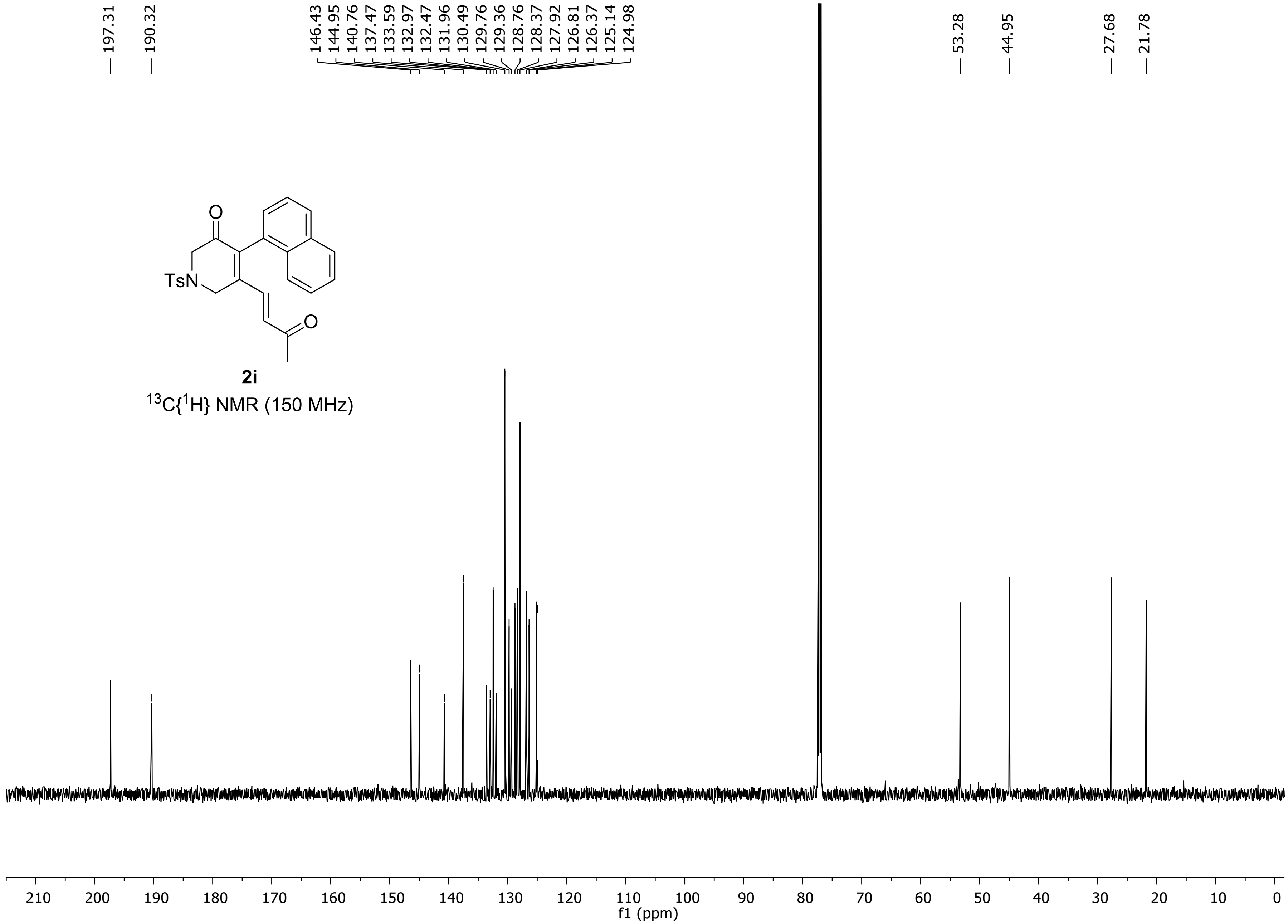
197.31
190.32
146.43
144.95
140.76
137.47
133.59
132.97
132.47
131.96
130.49
129.76
129.36
128.76
128.37
127.92
126.81
126.37
125.14
124.98
53.28
44.95
27.68
21.78
O
TsN
O
2i
$^{13}C\{^1H\}$ NMR (150 MHz)
210 200 190 180 170 160 150 140 130 120 110 100 90 80 70 60 50 40 30 20 10 0
f1 (ppm)

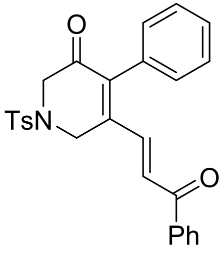

2j

^{1}H NMR (600 MHz)

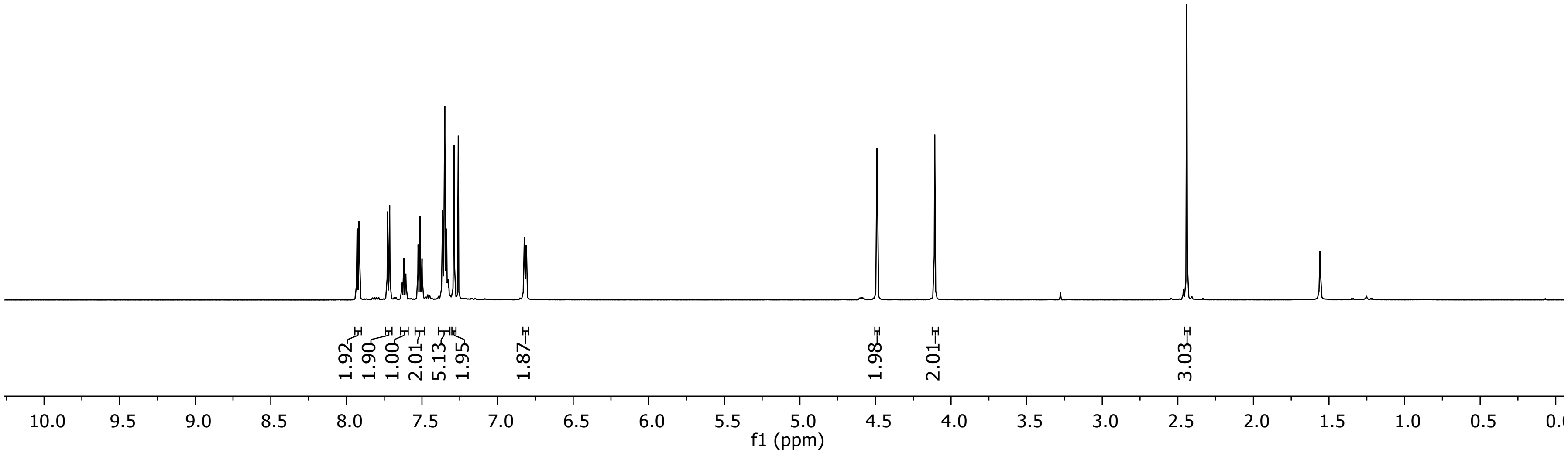

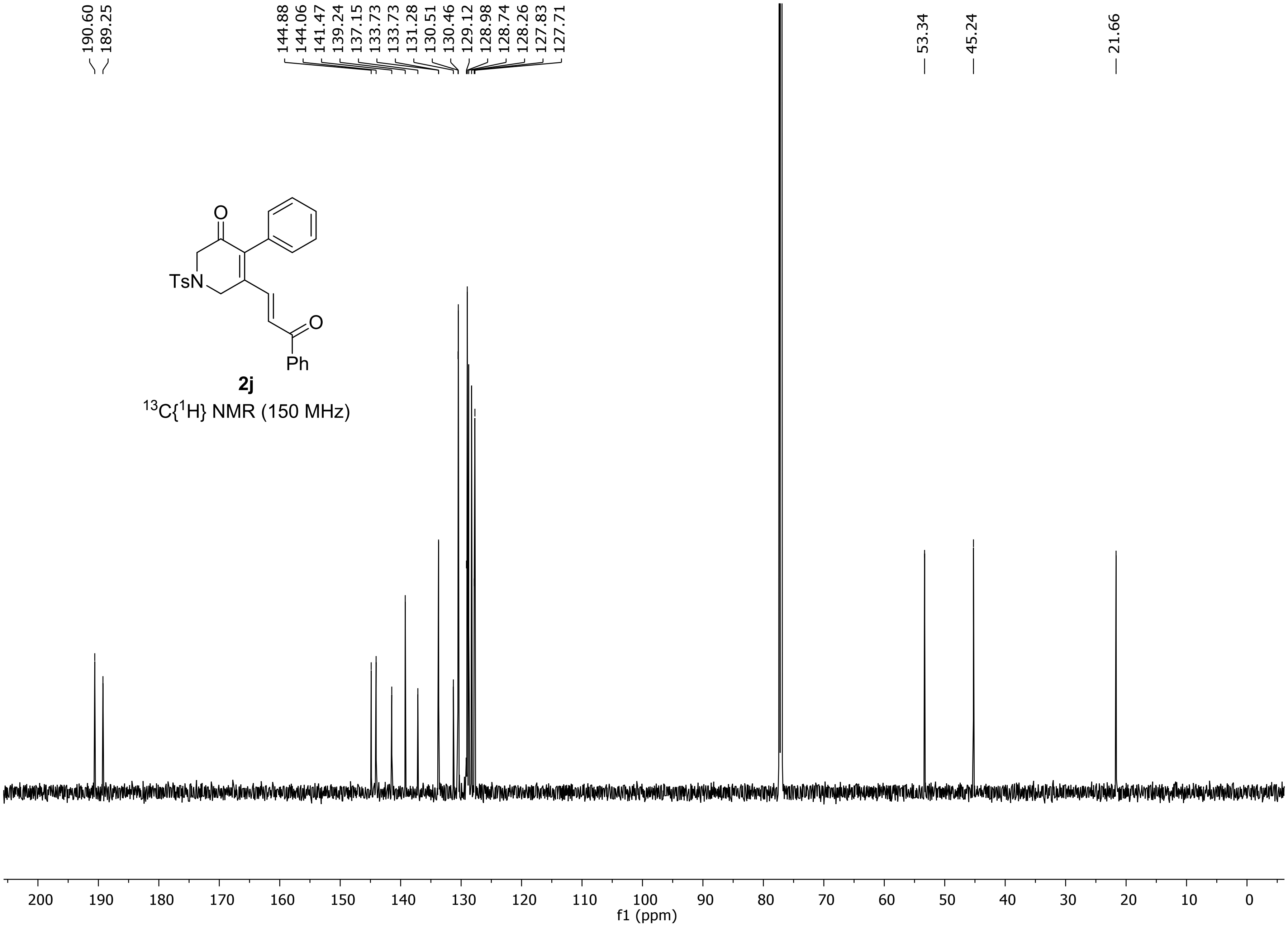

2j

$^{13}C\{^{1}H\}$ NMR (150 MHz)

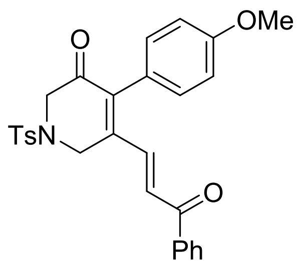

2k

^{1}H NMR (600 MHz)

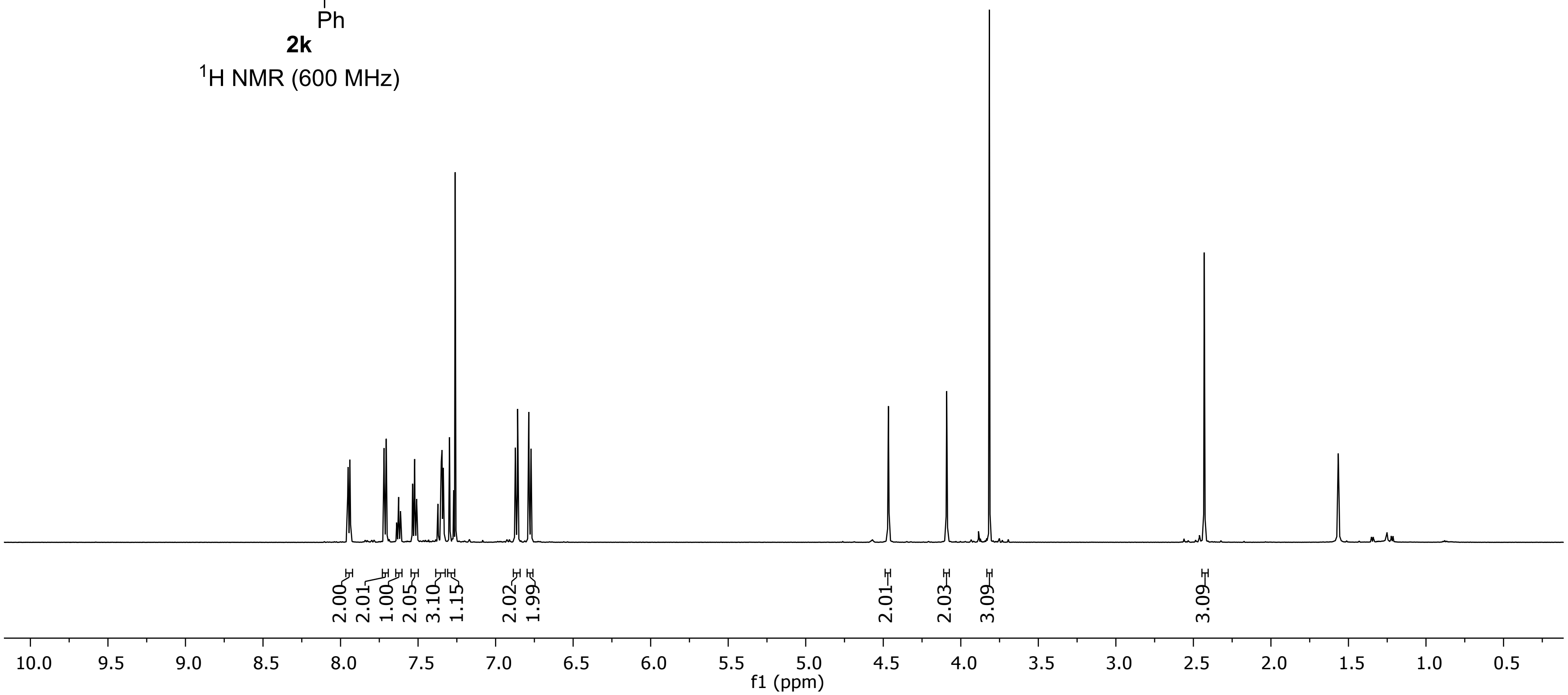

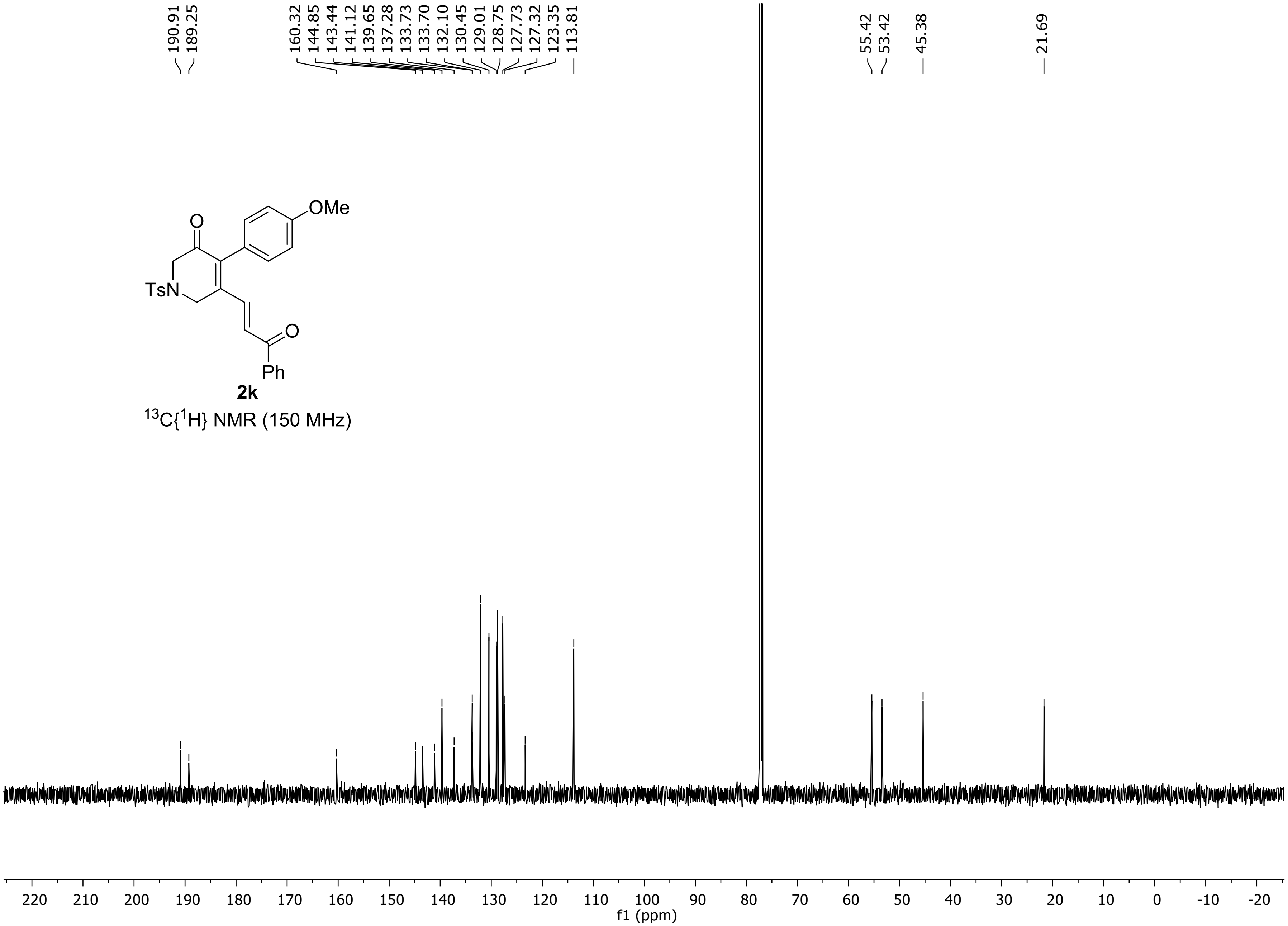

2k

$^{13}C\{^{1}H\}$ NMR (150 MHz)

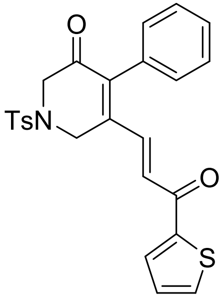

2l

^{1}H NMR (600 MHz)

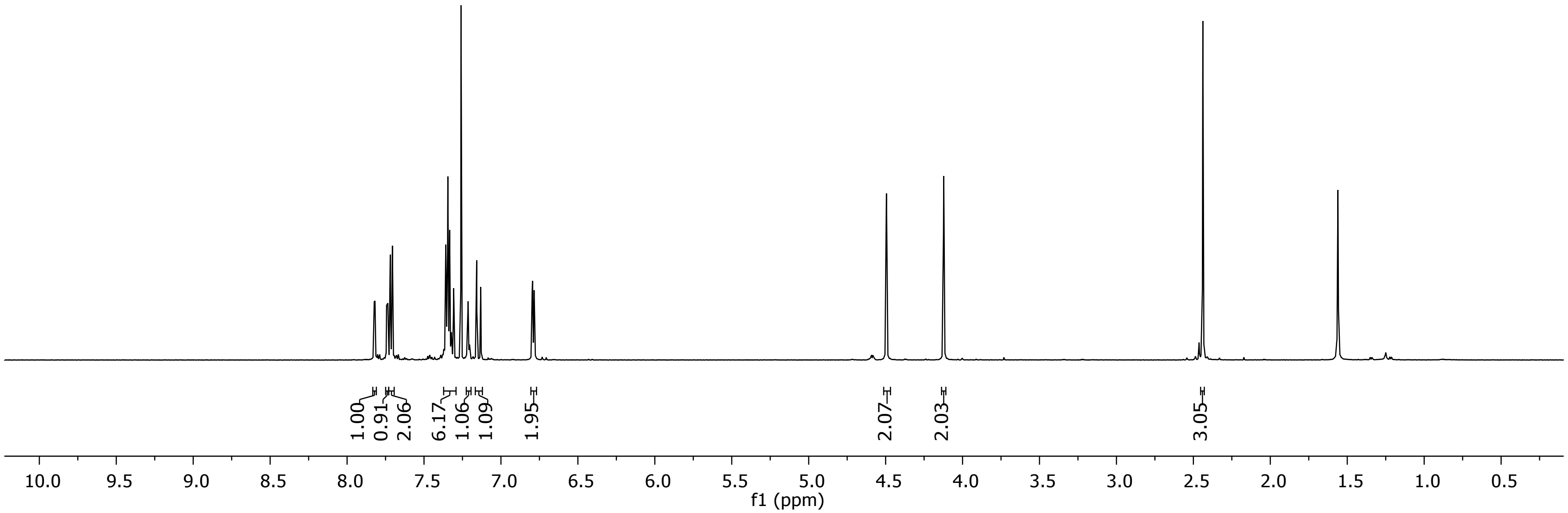

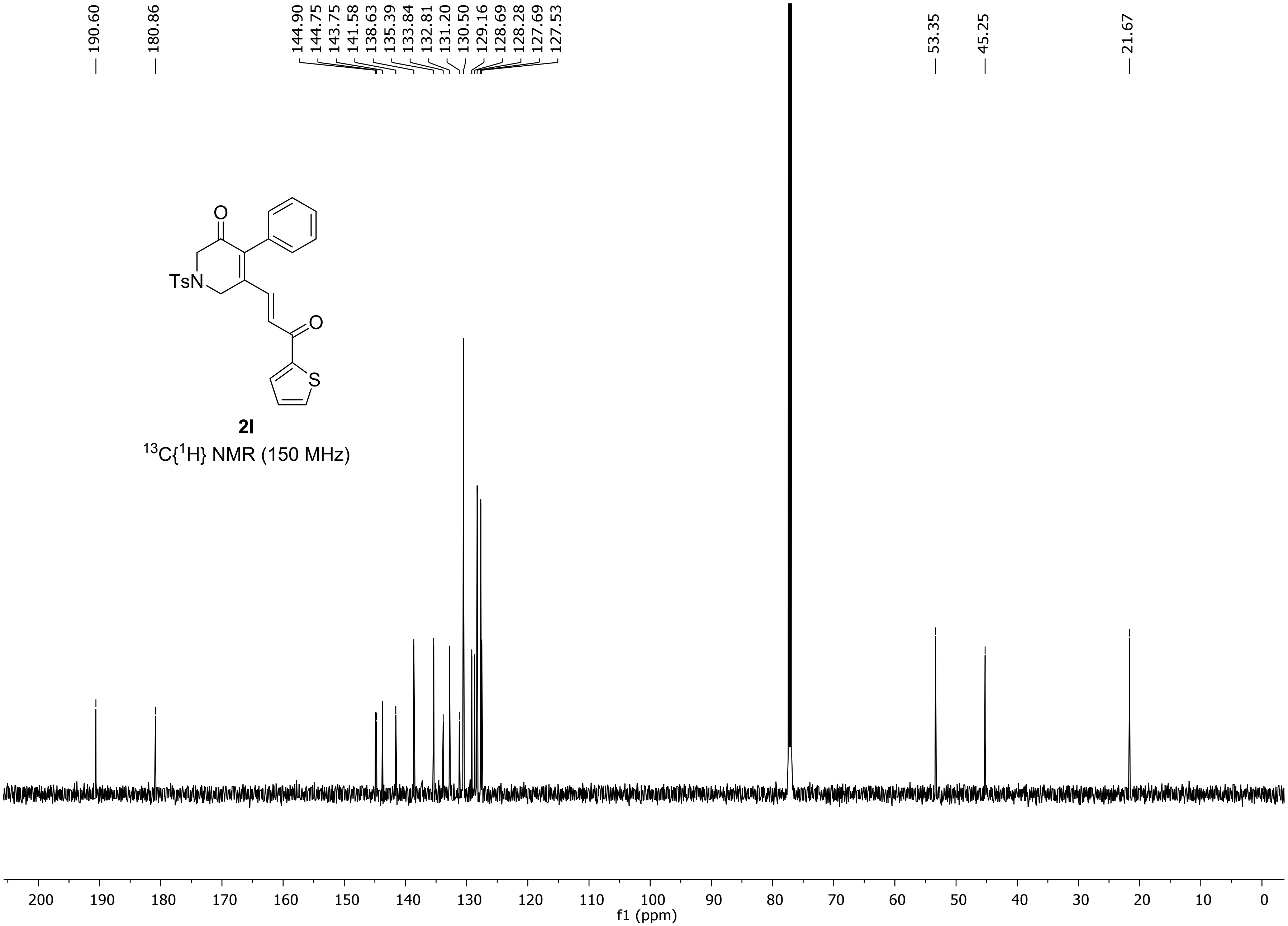

2l

$^{13}C\{^1H\}$ NMR (150 MHz)

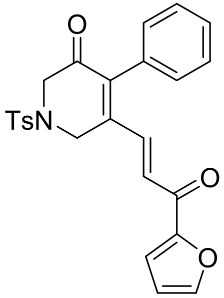

2m

^{1}H NMR (600 MHz)

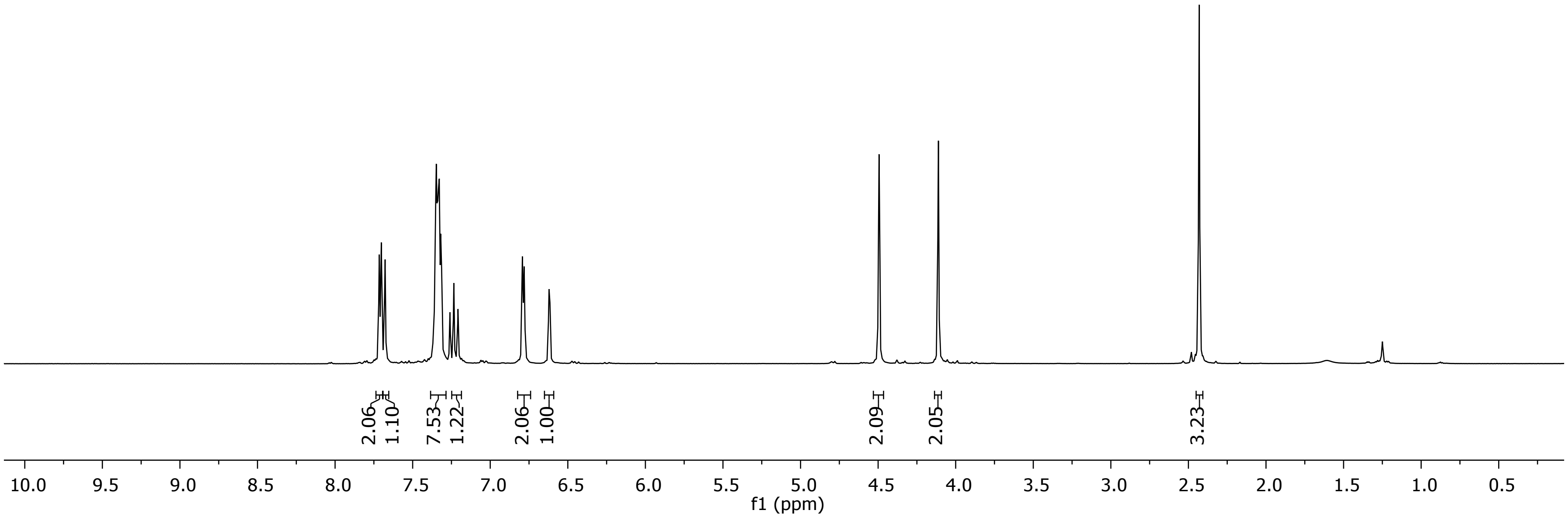

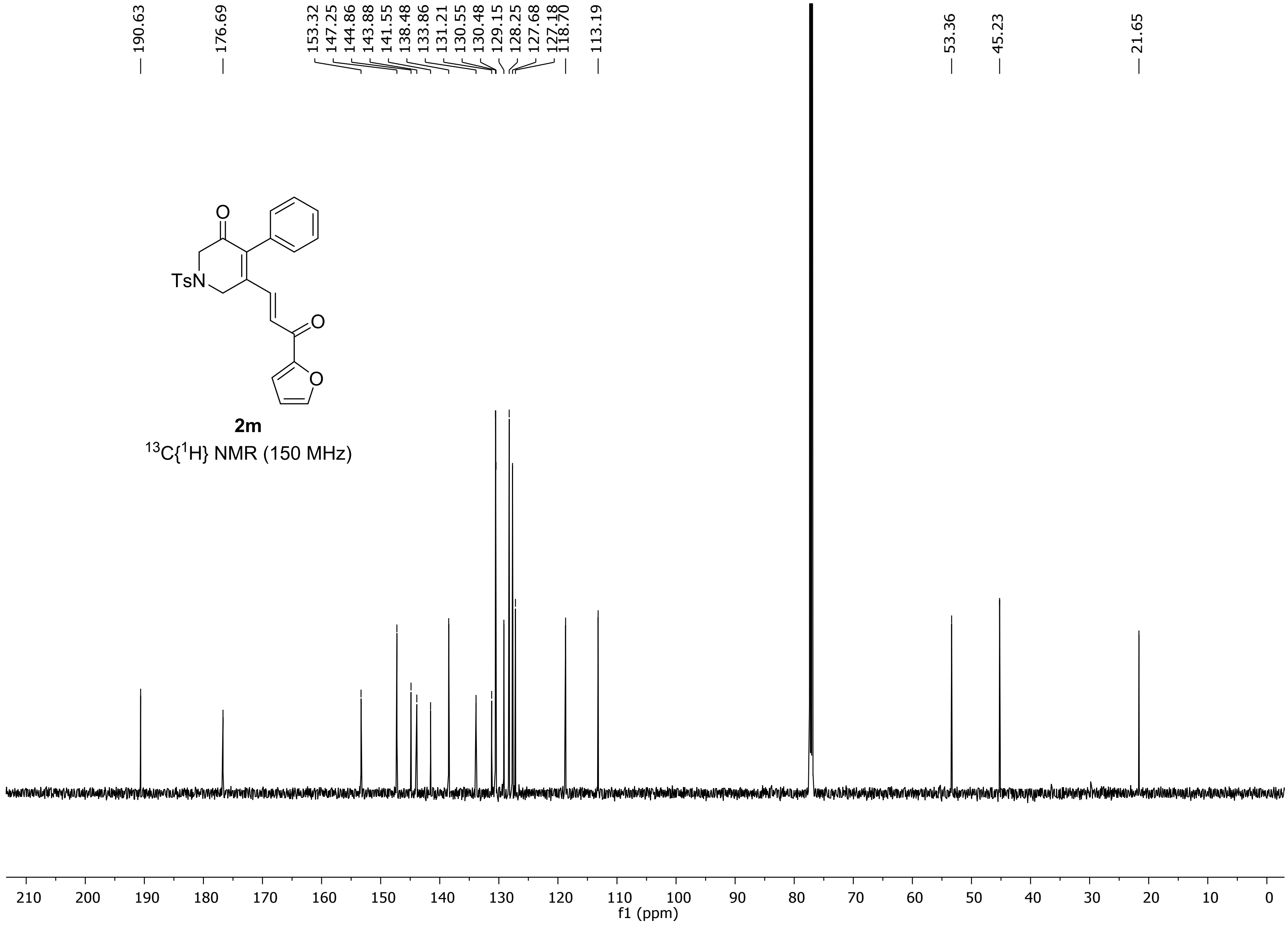
190.63
176.69
153.32
147.25
144.86
143.88
141.55
138.48
133.86
131.21
130.55
130.48
129.15
128.25
127.68
127.18
118.70
113.19
53.36
45.23
21.65
TsN
O
2m
$^{13}C\{^1H\}$ NMR (150 MHz)
210 200 190 180 170 160 150 140 130 120 110 100 90 80 70 60 50 40 30 20 10 0
f1 (ppm)

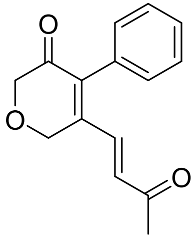

2n
^{1}H NMR (600 MHz)

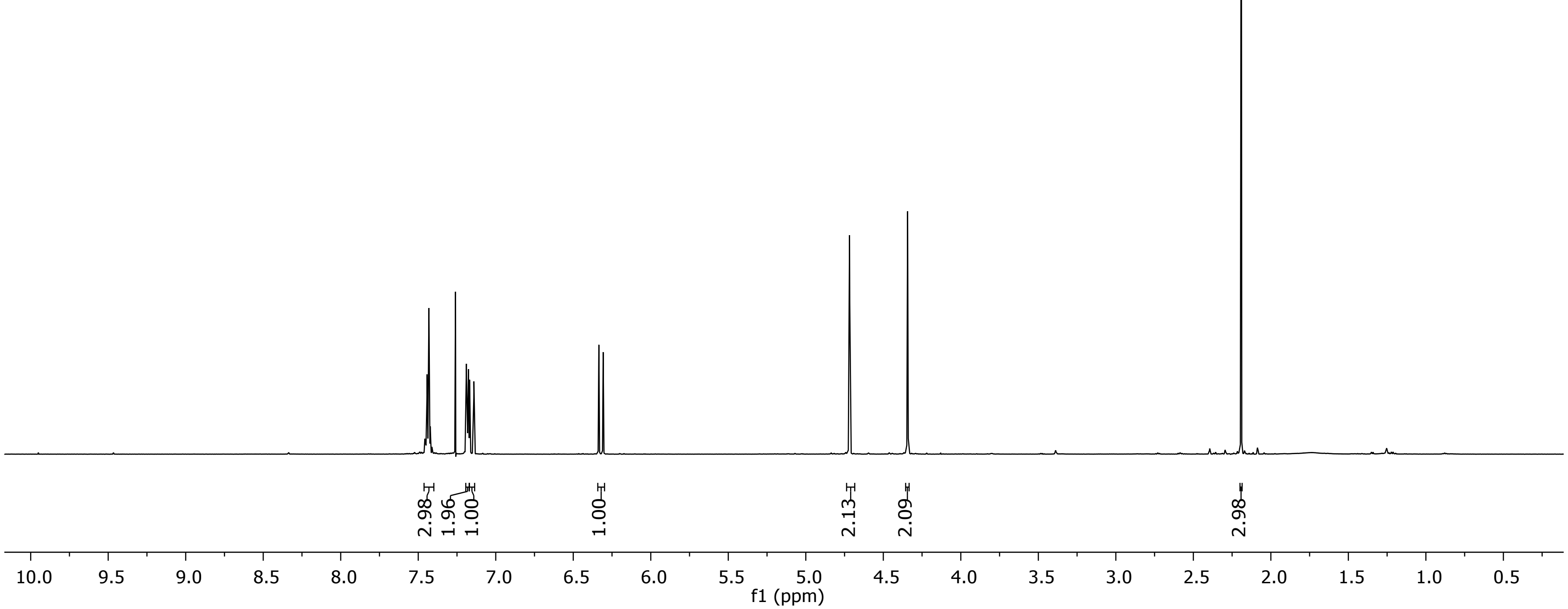

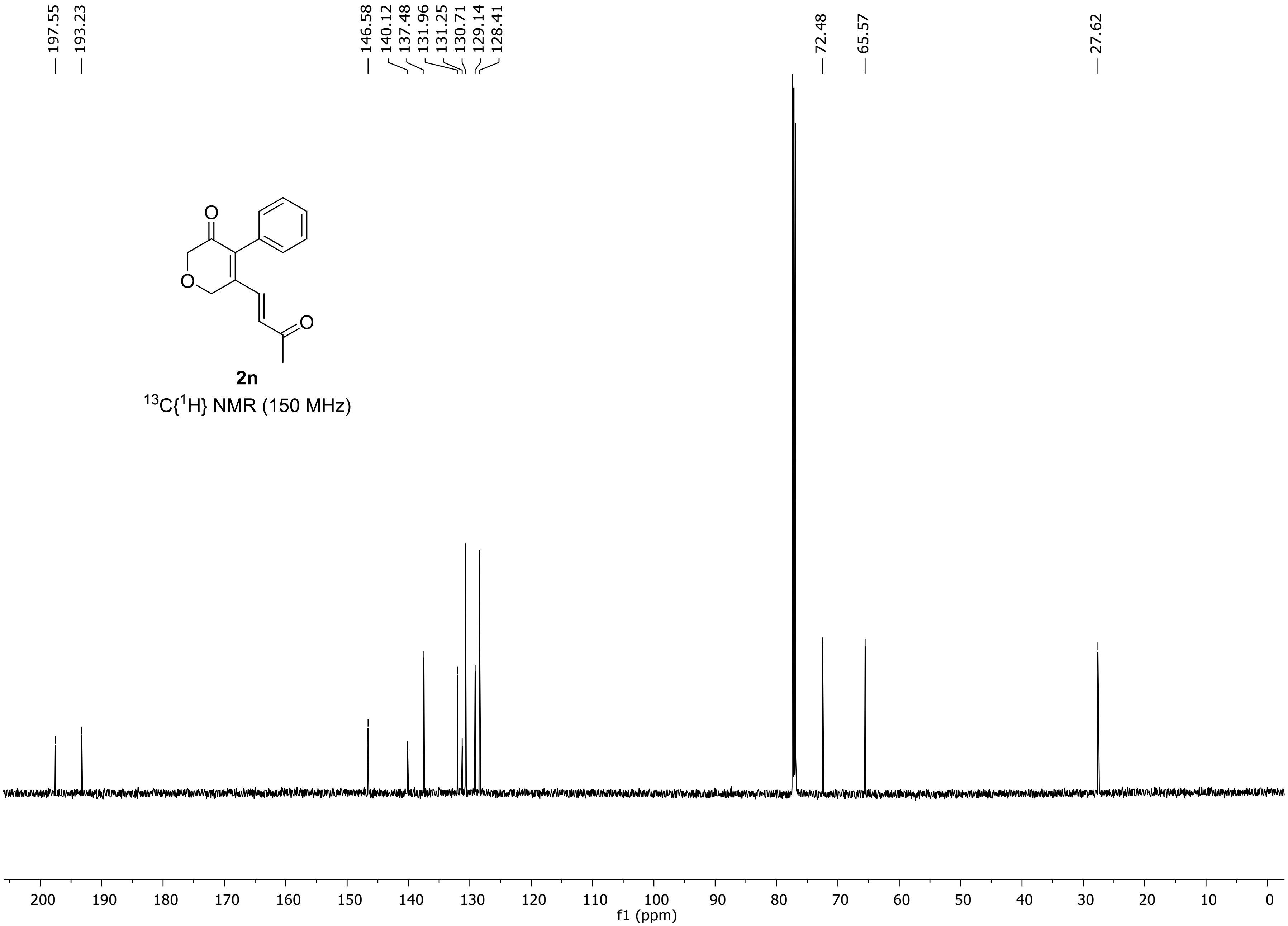
197.55
193.23
146.58
140.12
137.48
131.96
131.25
130.71
129.14
128.41
72.48
65.57
27.62
O
O
O
2n
^{13}C{^{1}H} NMR (150 MHz)
200 190 180 170 160 150 140 130 120 110 100 90 80 70 60 50 40 30 20 10 0
f1 (ppm)

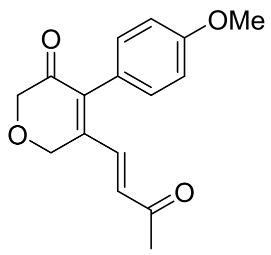

2o

^{1}H NMR (600 MHz)

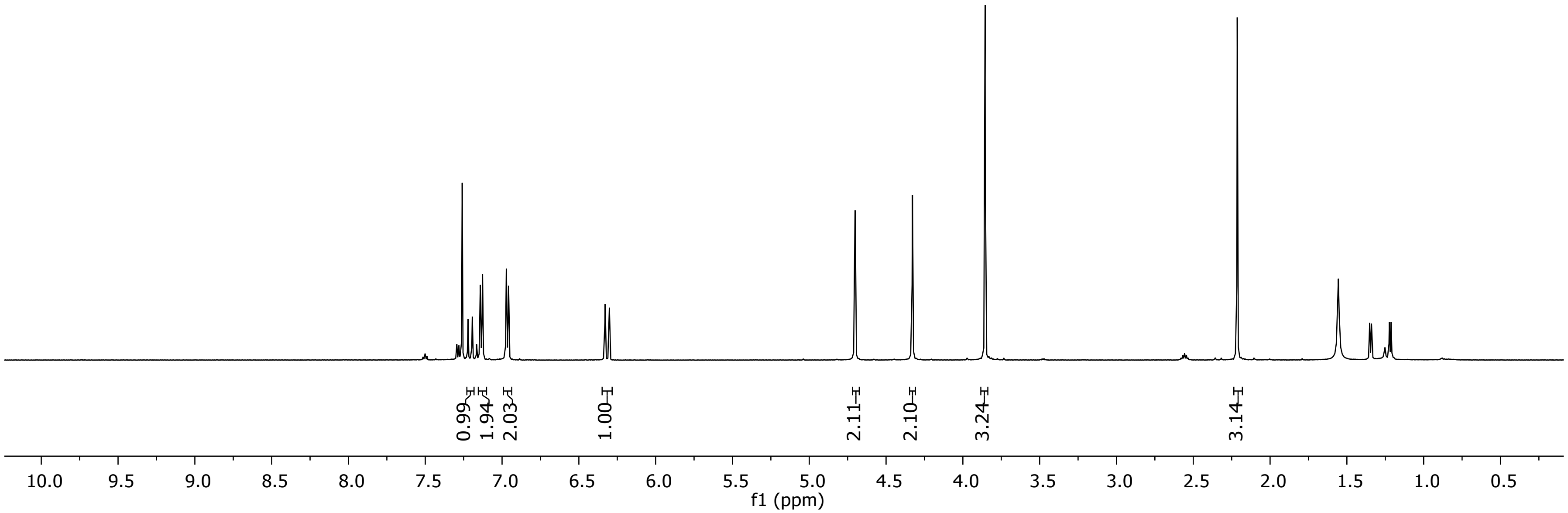

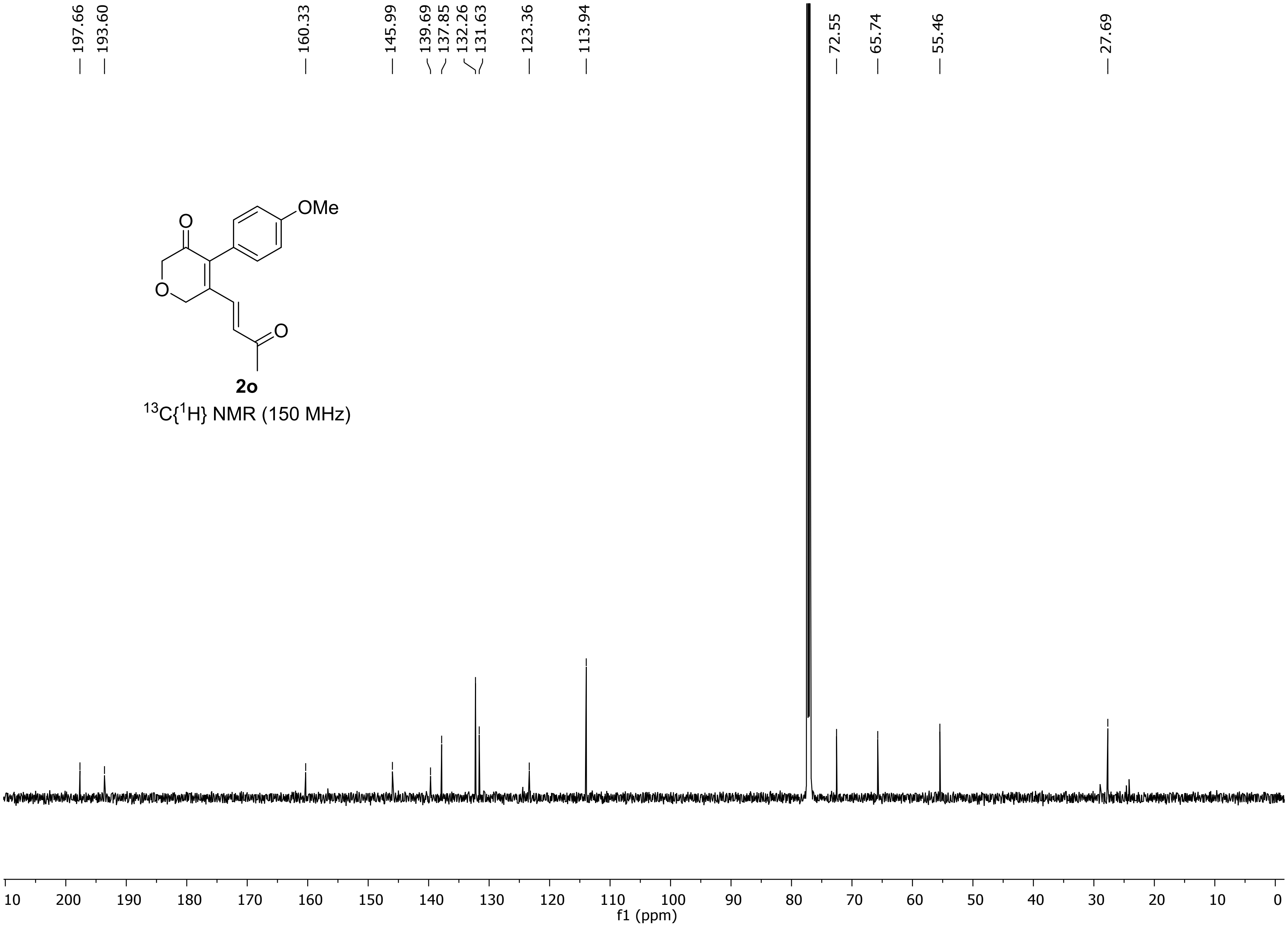
197.66
193.60
160.33
145.99
139.69
137.85
132.26
131.63
123.36
113.94
72.55
65.74
55.46
27.69
OMe
O
O
O
2o
13C{1H} NMR (150 MHz)
10 200 190 180 170 160 150 140 130 120 110 100 90 80 70 60 50 40 30 20 10 0
f1 (ppm)

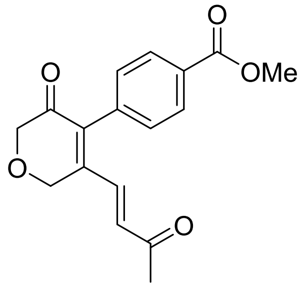

2p

^{1}H NMR (600 MHz)

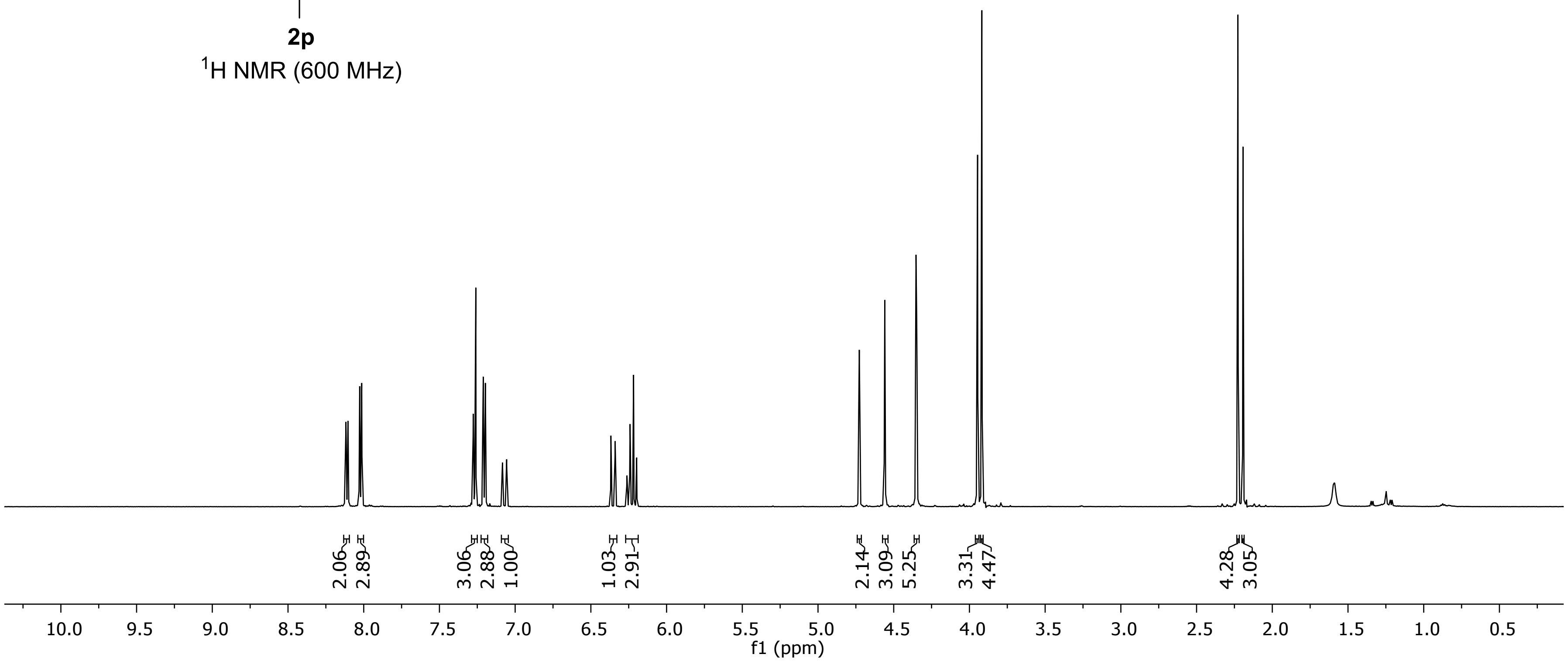

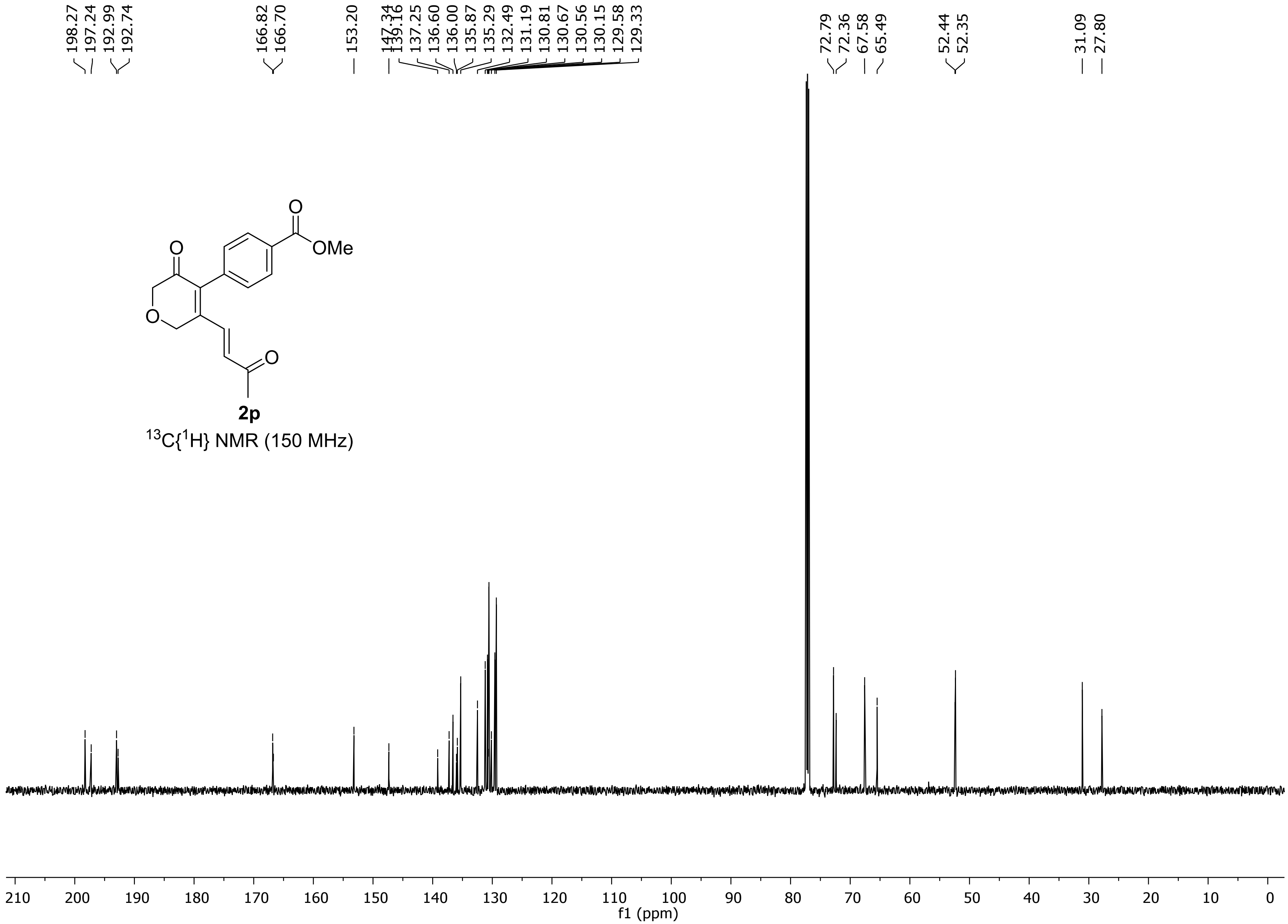

2p

$^{13}C\{^{1}H\}$ NMR (150 MHz)

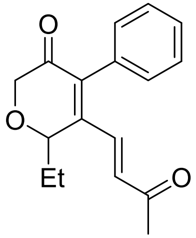

2q
^{1}H NMR (600 MHz)

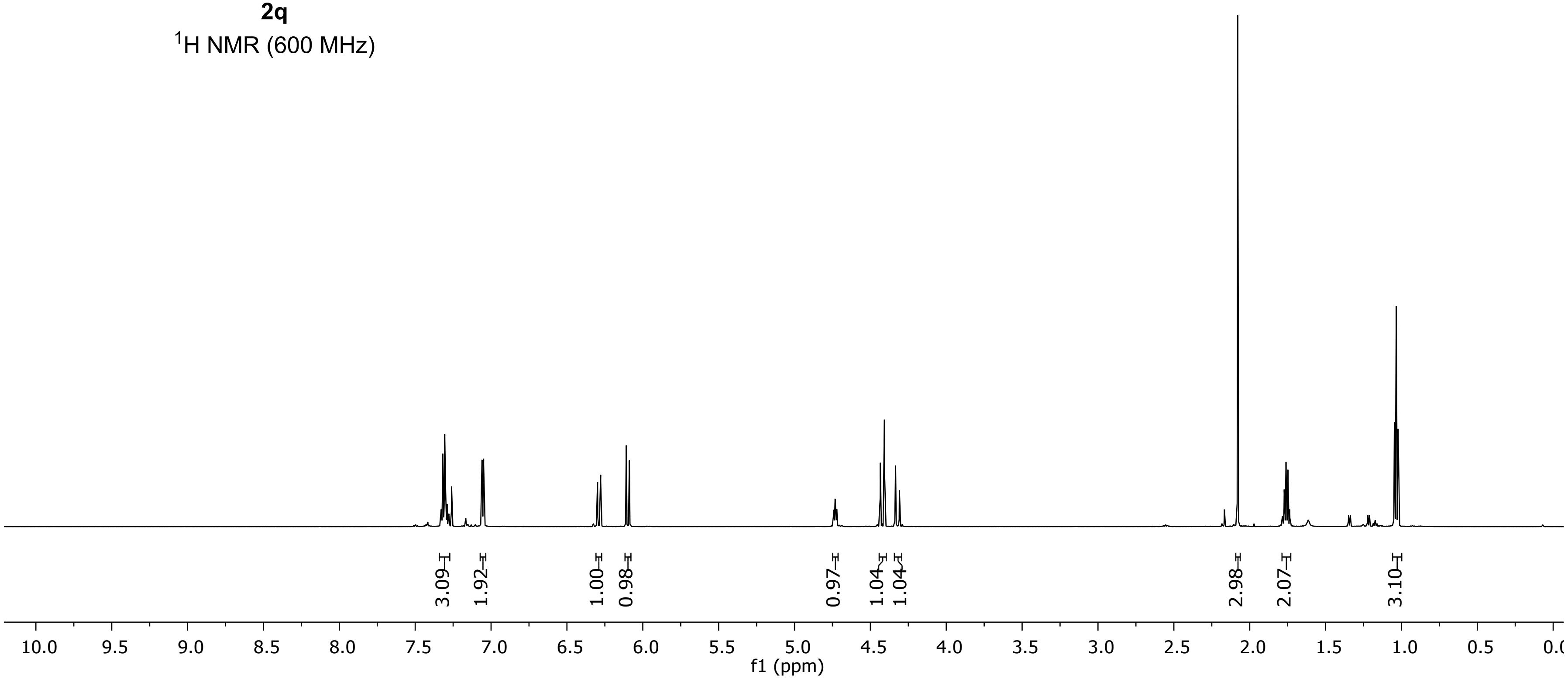

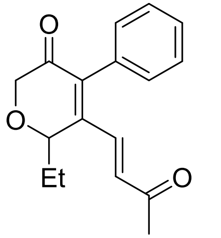

2q

$^{13}C\{^1H\}$ NMR (150 MHz)

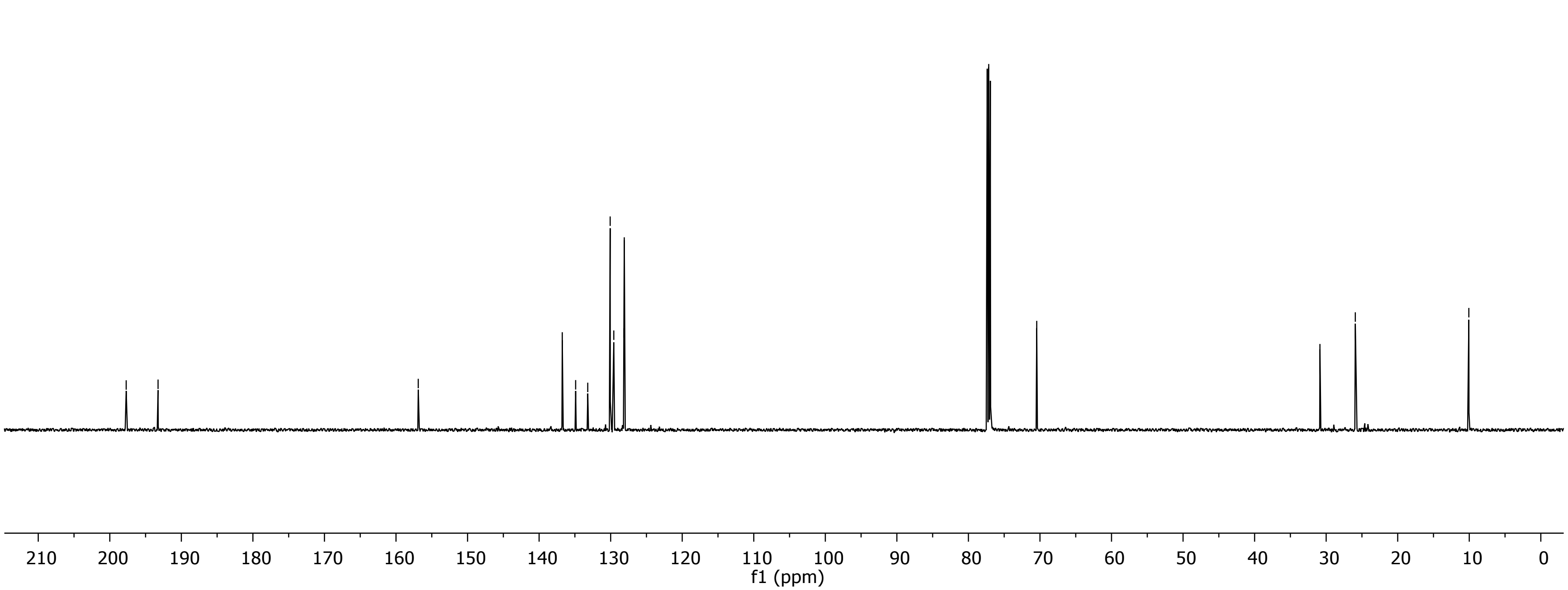

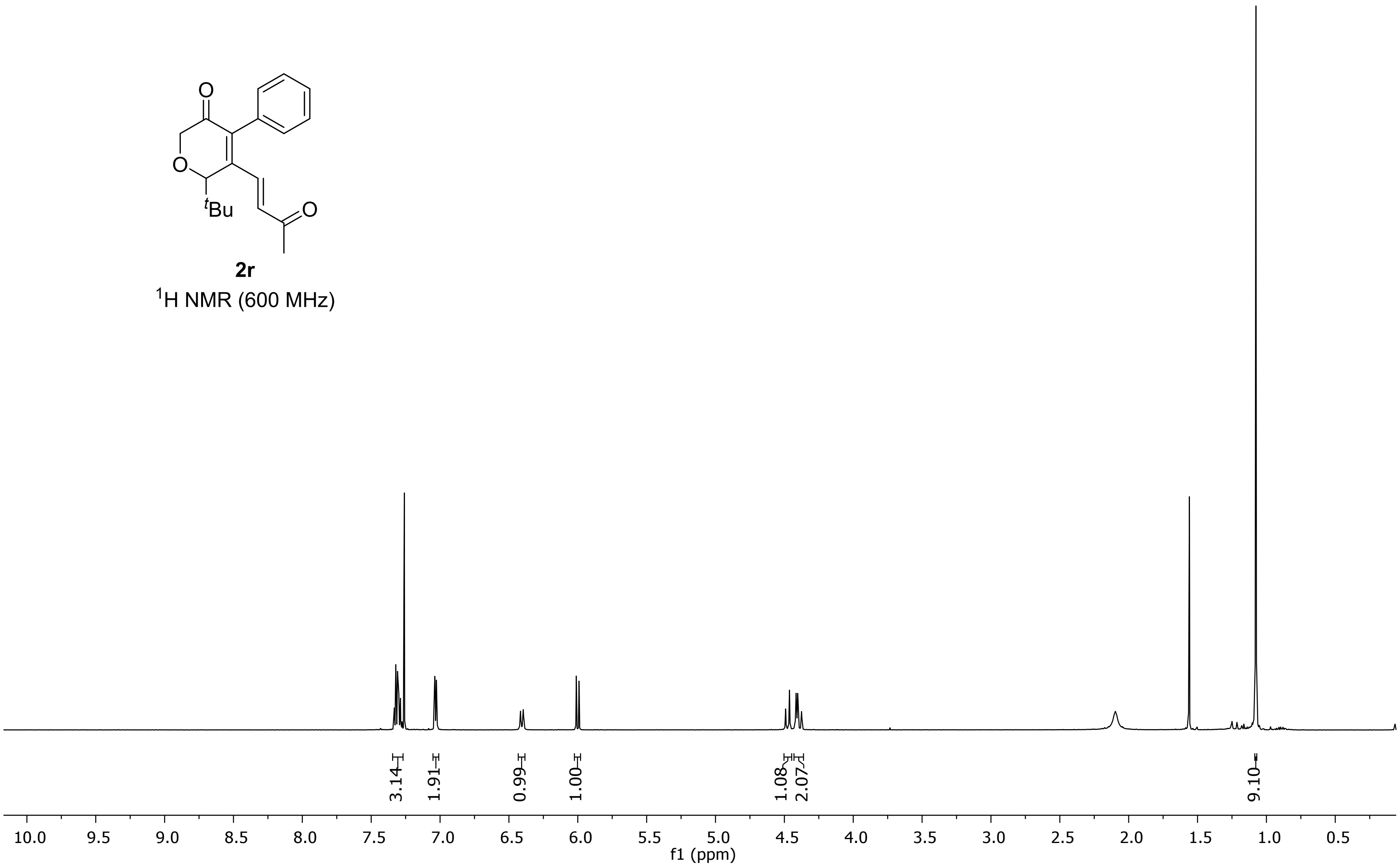
O
O
O
tBu
2r
1H NMR (600 MHz)
3.14
1.91
0.99
1.00
1.08
2.07
9.10
10.0 9.5 9.0 8.5 8.0 7.5 7.0 6.5 6.0 5.5 5.0 4.5 4.0 3.5 3.0 2.5 2.0 1.5 1.0 0.5
f1 (ppm)

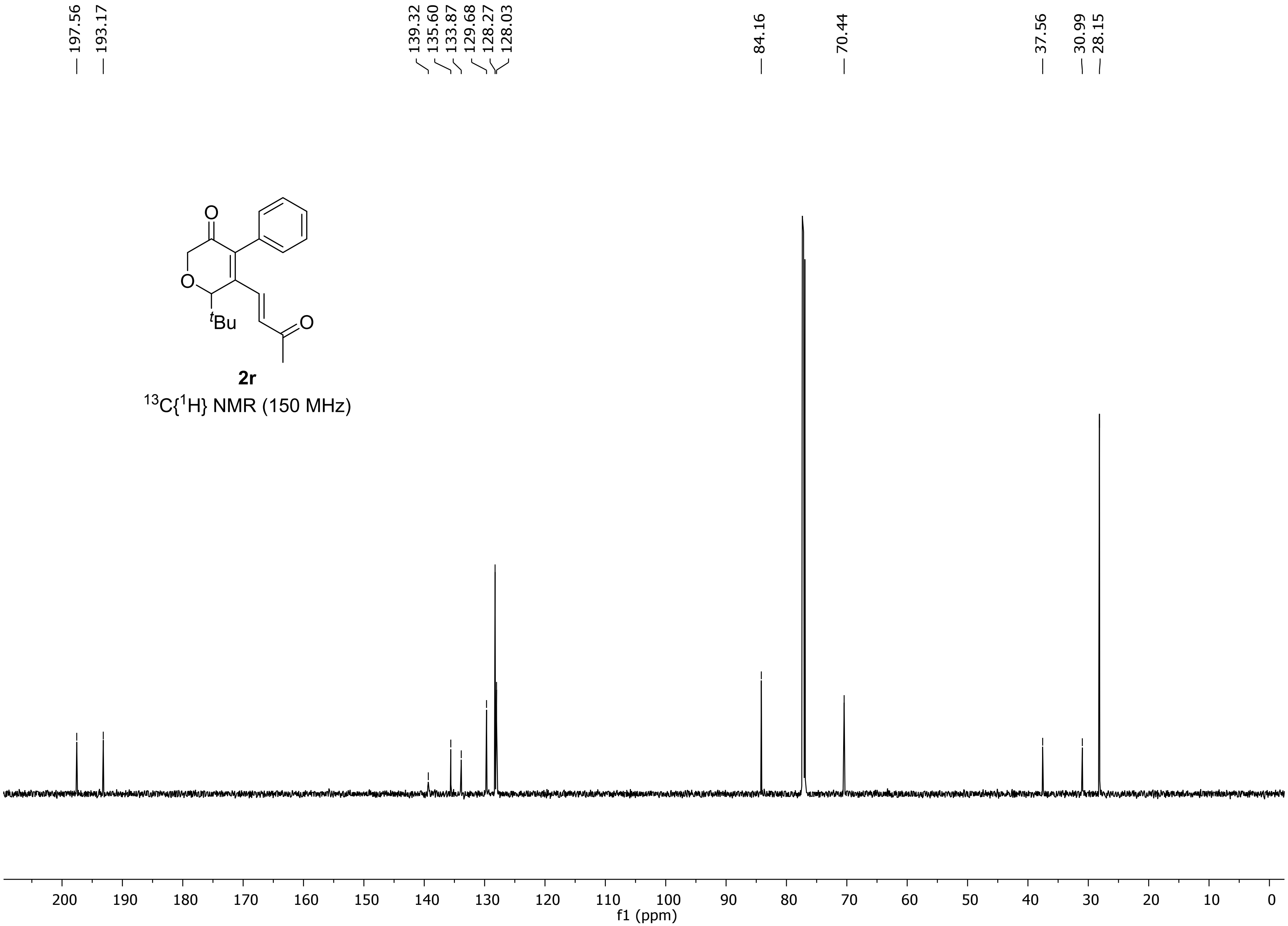
197.56
193.17
139.32
135.60
133.87
129.68
128.27
128.03
84.16
70.44
37.56
30.99
28.15
O
O
tBu
O
2r
13C{1H} NMR (150 MHz)
200 190 180 170 160 150 140 130 120 110 100 90 80 70 60 50 40 30 20 10 0
f1 (ppm)

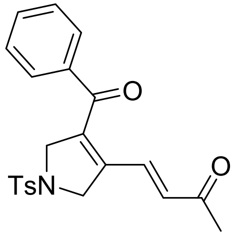

3a

^{1}H NMR (600 MHz)

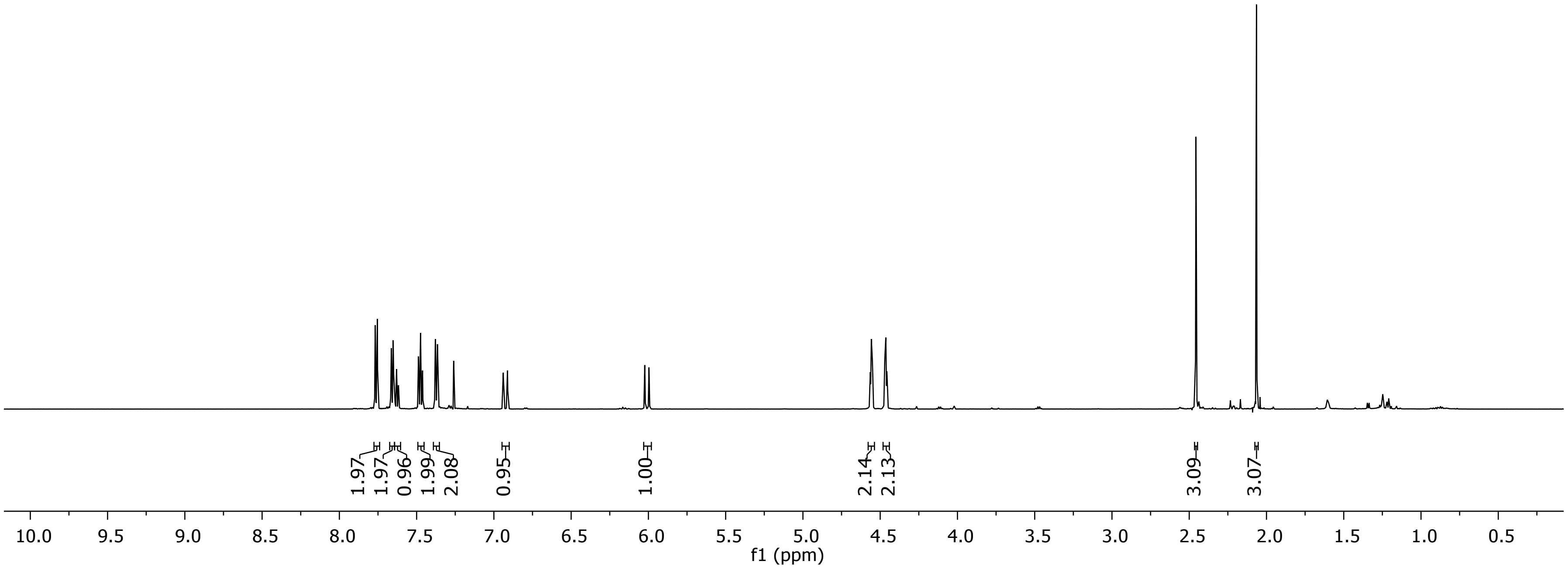

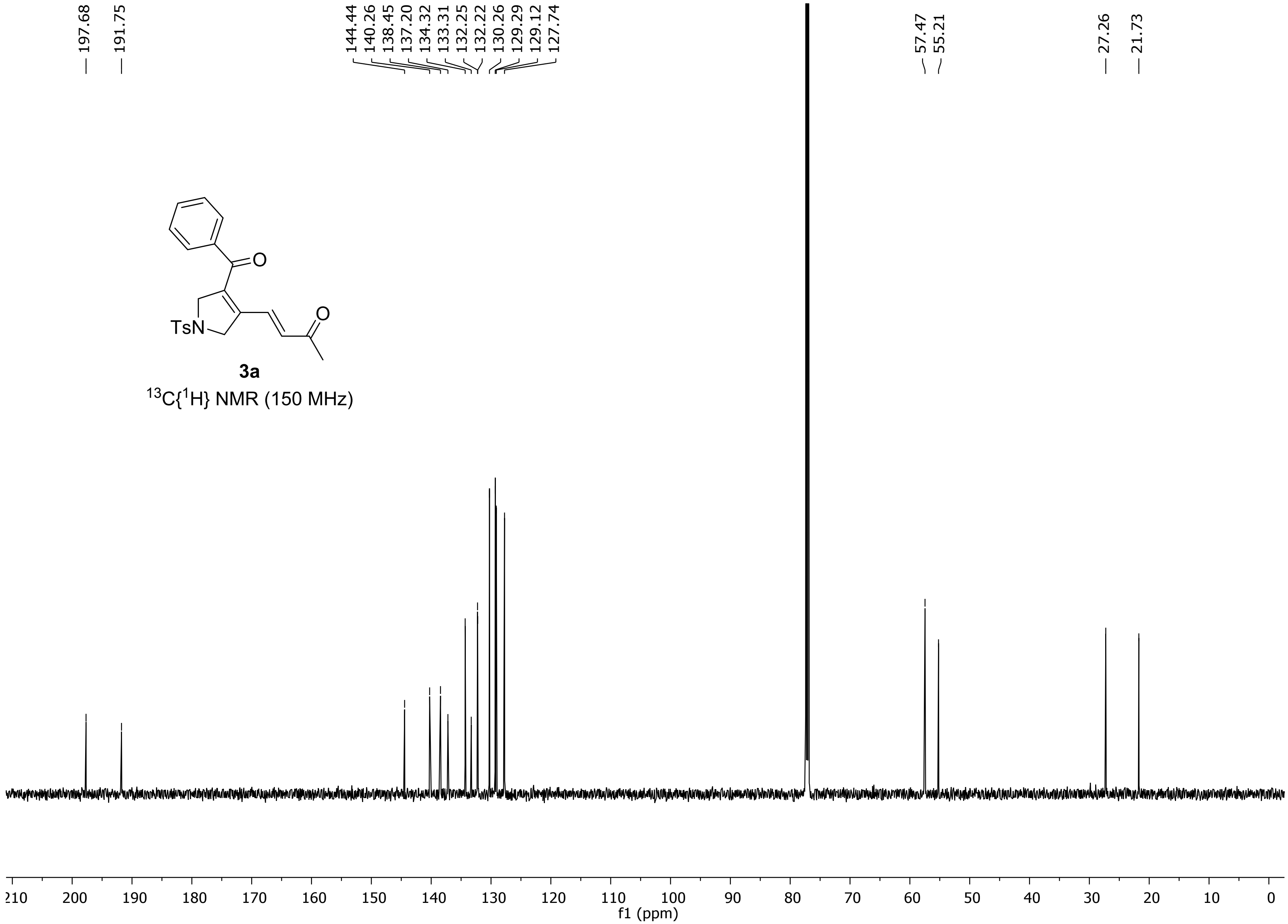
197.68
191.75
144.44
140.26
138.45
137.20
134.32
133.31
132.25
132.22
130.26
129.29
129.12
127.74
57.47
55.21
27.26
21.73
O
O
TsN
3a
$^{13}C\{^1H\}$ NMR (150 MHz)
210 200 190 180 170 160 150 140 130 120 110 100 90 80 70 60 50 40 30 20 10 0
f1 (ppm)

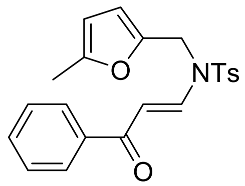

4a
^1H NMR (600 MHz)

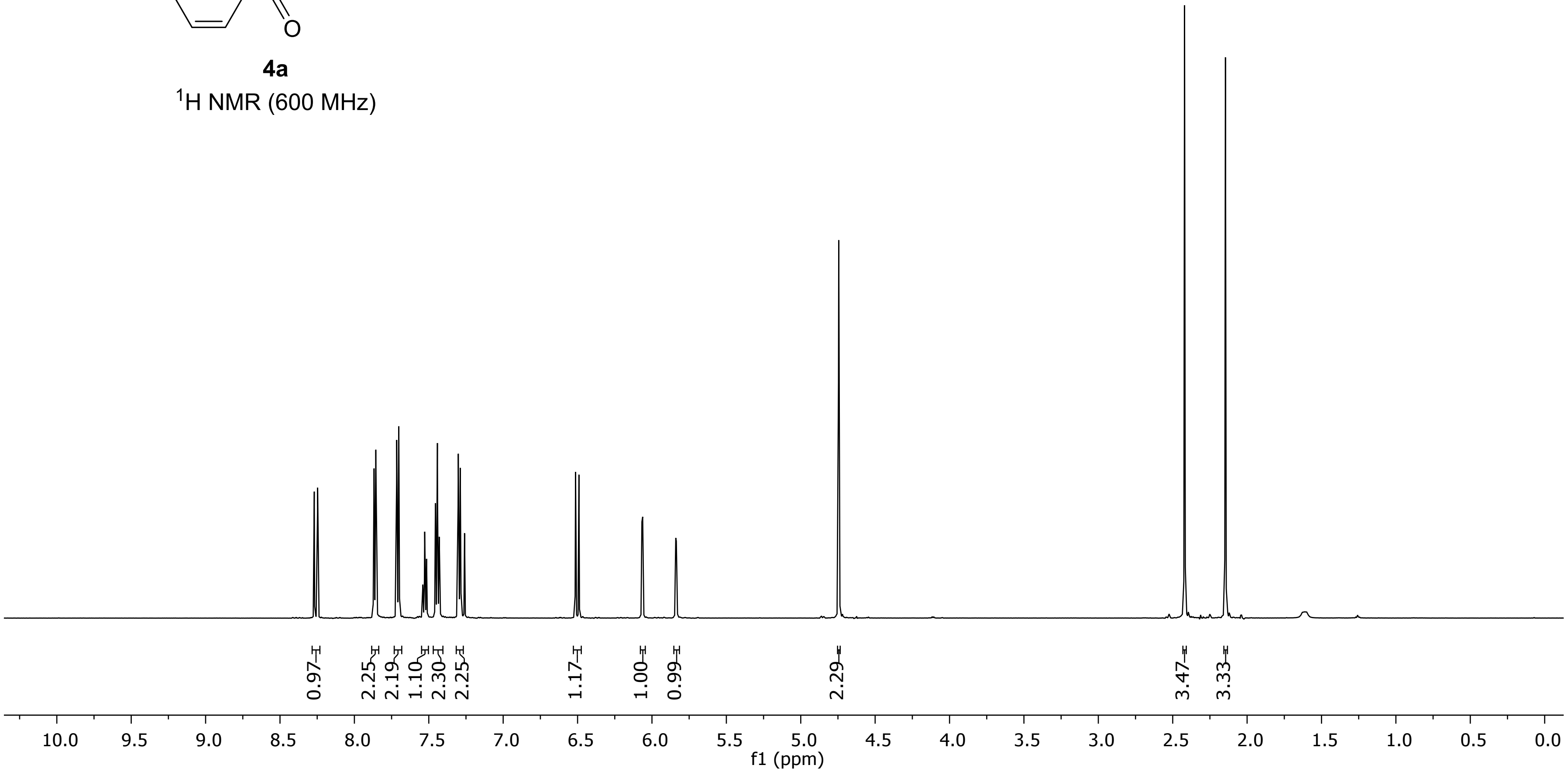

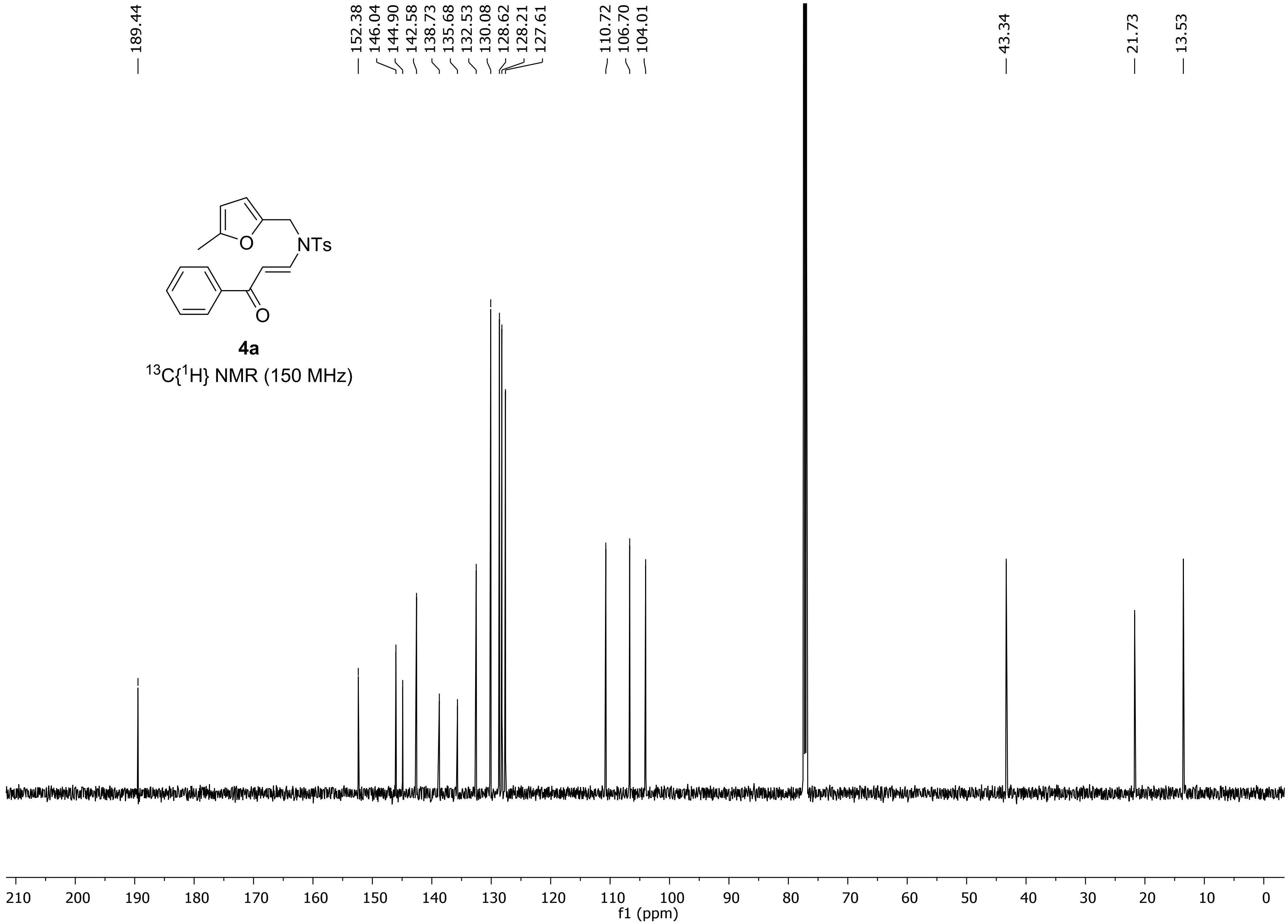
189.44
152.38
146.04
144.90
142.58
138.73
135.68
132.53
130.08
128.62
128.21
127.61
110.72
106.70
104.01
43.34
21.73
13.53
NTs
O
O
4a
$^{13}C\{^{1}H\}$ NMR (150 MHz)
210 200 190 180 170 160 150 140 130 120 110 100 90 80 70 60 50 40 30 20 10 0
f1 (ppm)

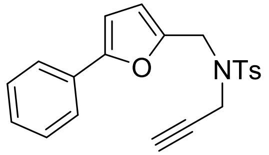

int-7

^{1}H NMR (600 MHz)

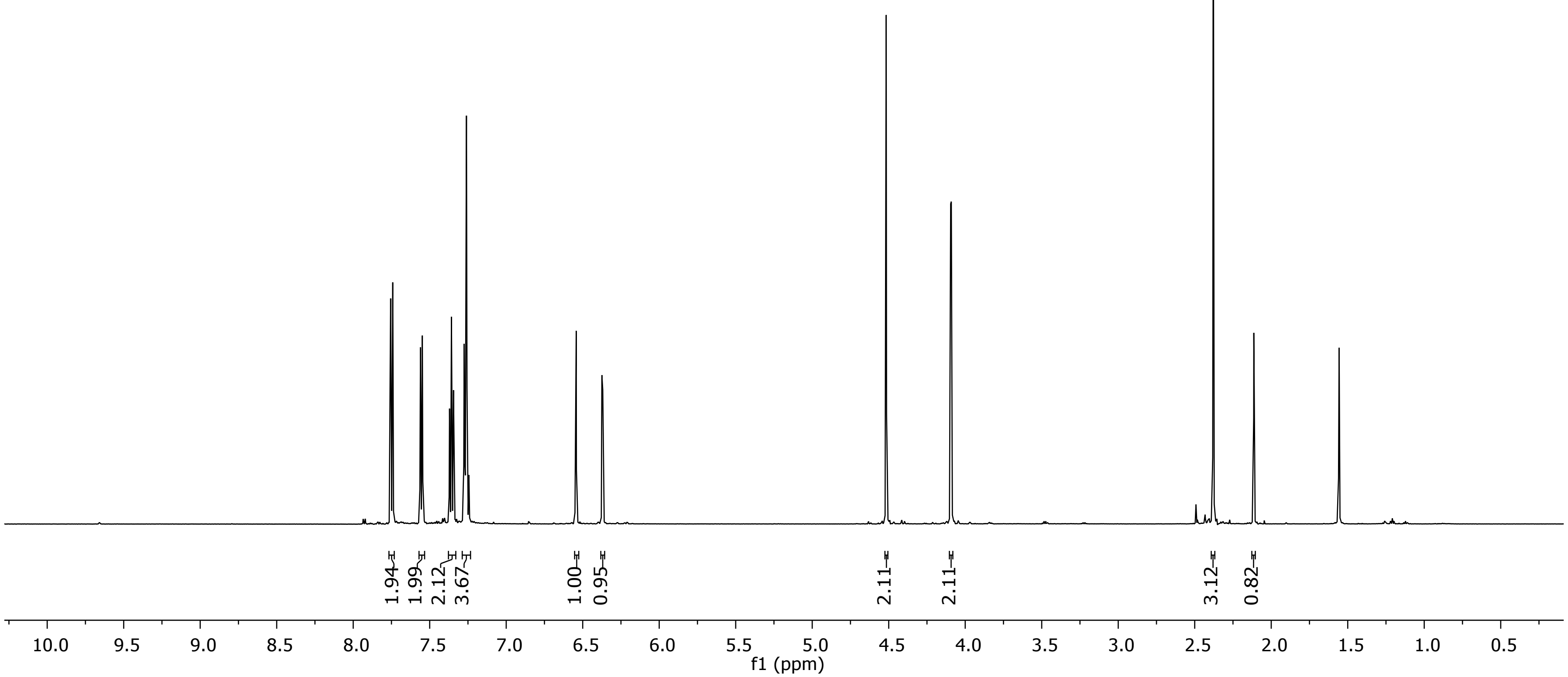

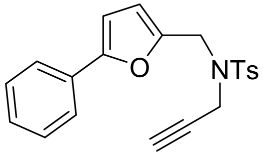

int-7

$^{13}C\{^1H\}$ NMR (150 MHz)

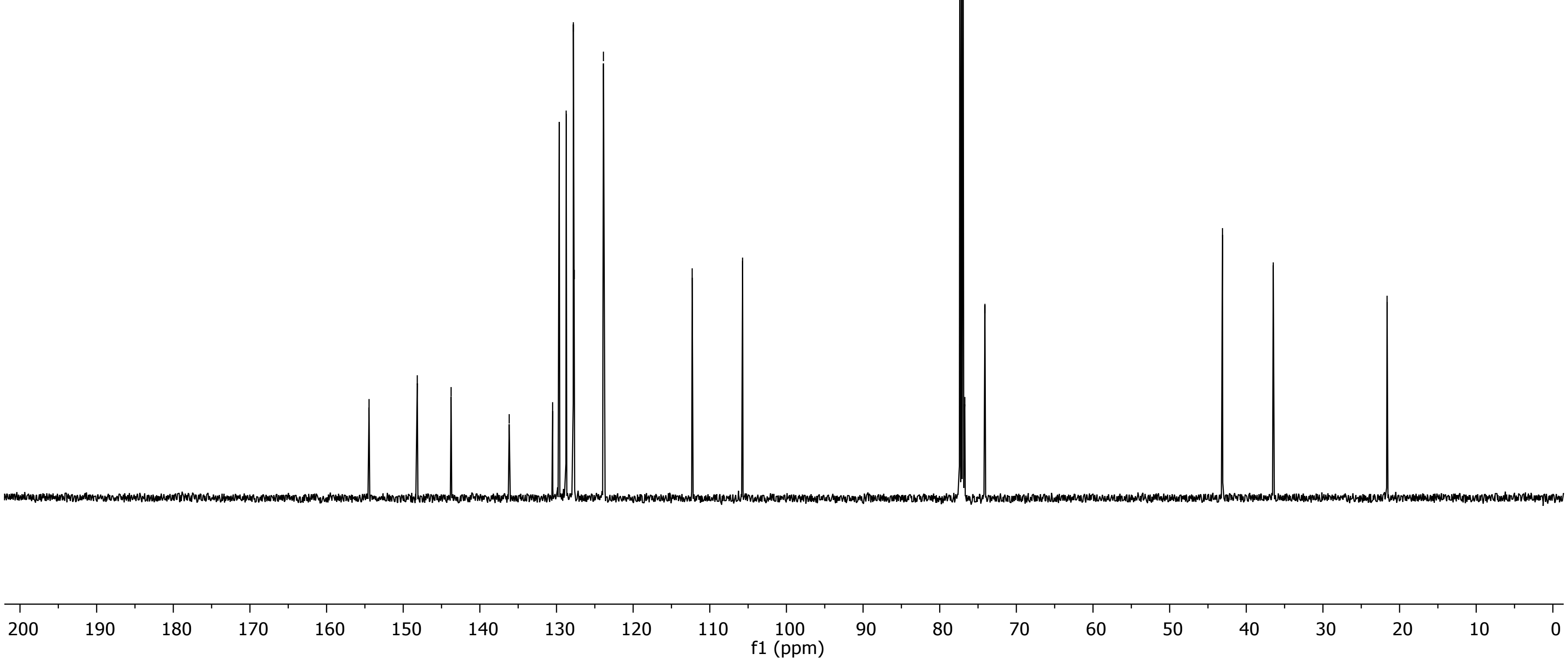

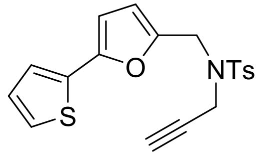

int-8

^{1}H NMR (600 MHz)

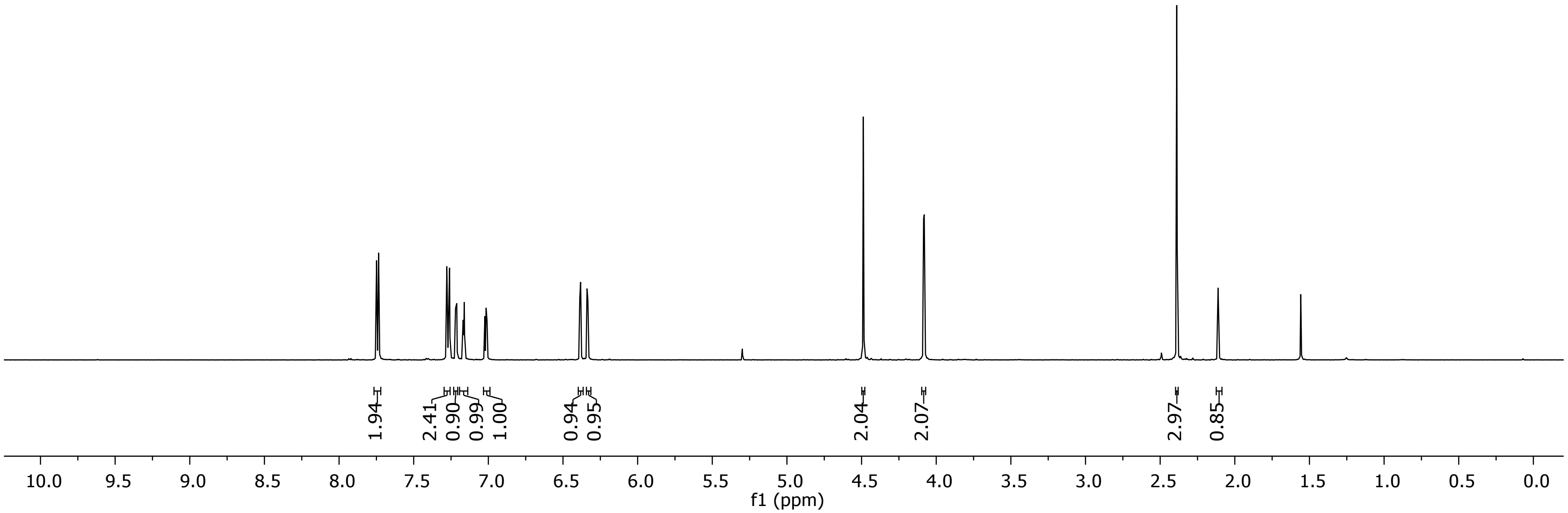

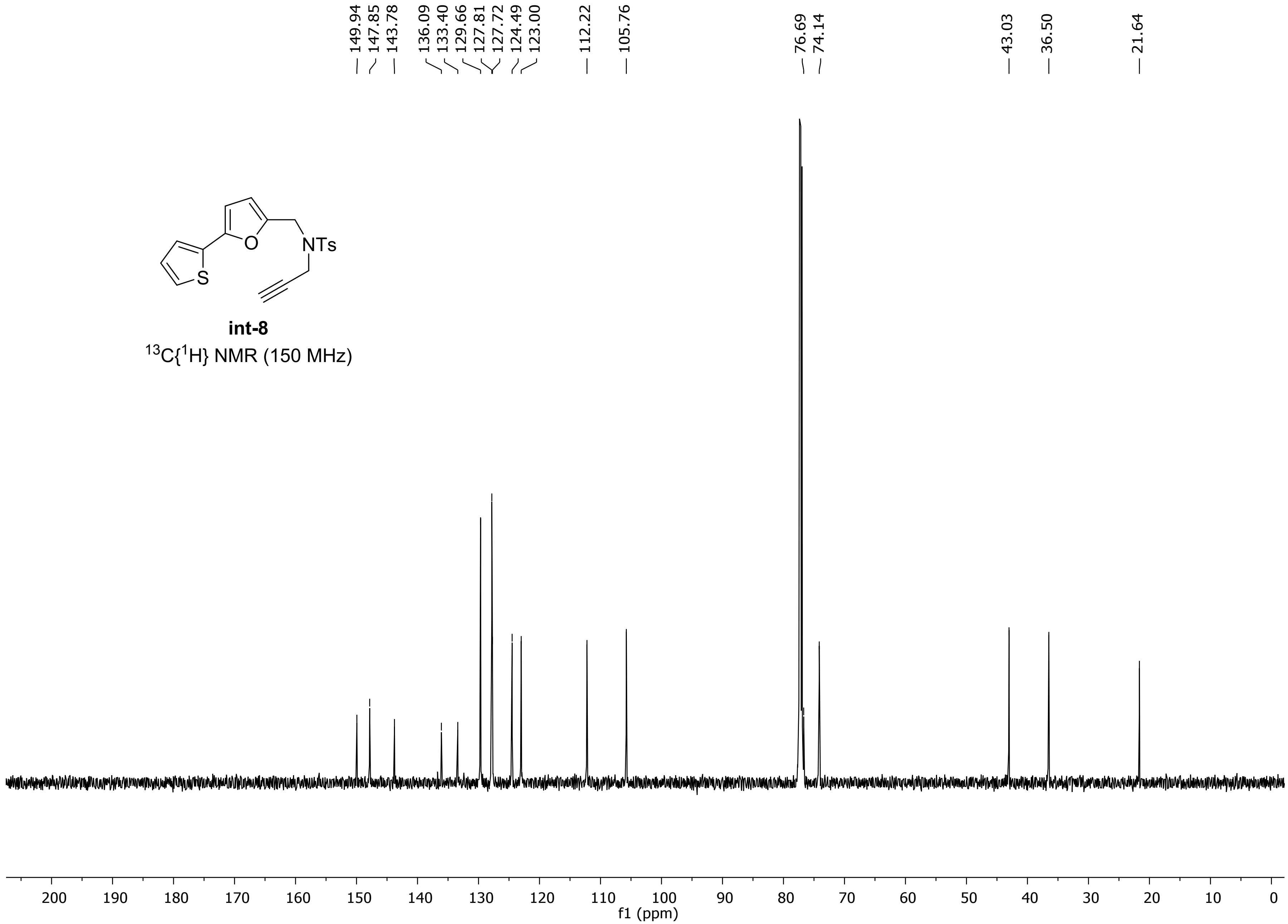
149.94
147.85
143.78
136.09
133.40
129.66
127.81
127.72
124.49
123.00
112.22
105.76
76.69
74.14
43.03
36.50
21.64
NTs
O
S
int-8
$^{13}C\{^1H\}$ NMR (150 MHz)
200
190
180
170
160
150
140
130
120
110
100
90
80
70
60
50
40
30
20
10
0
f1 (ppm)

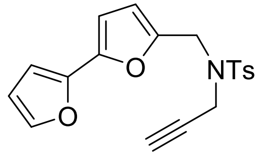

int-9
^{1}H NMR (600 MHz)

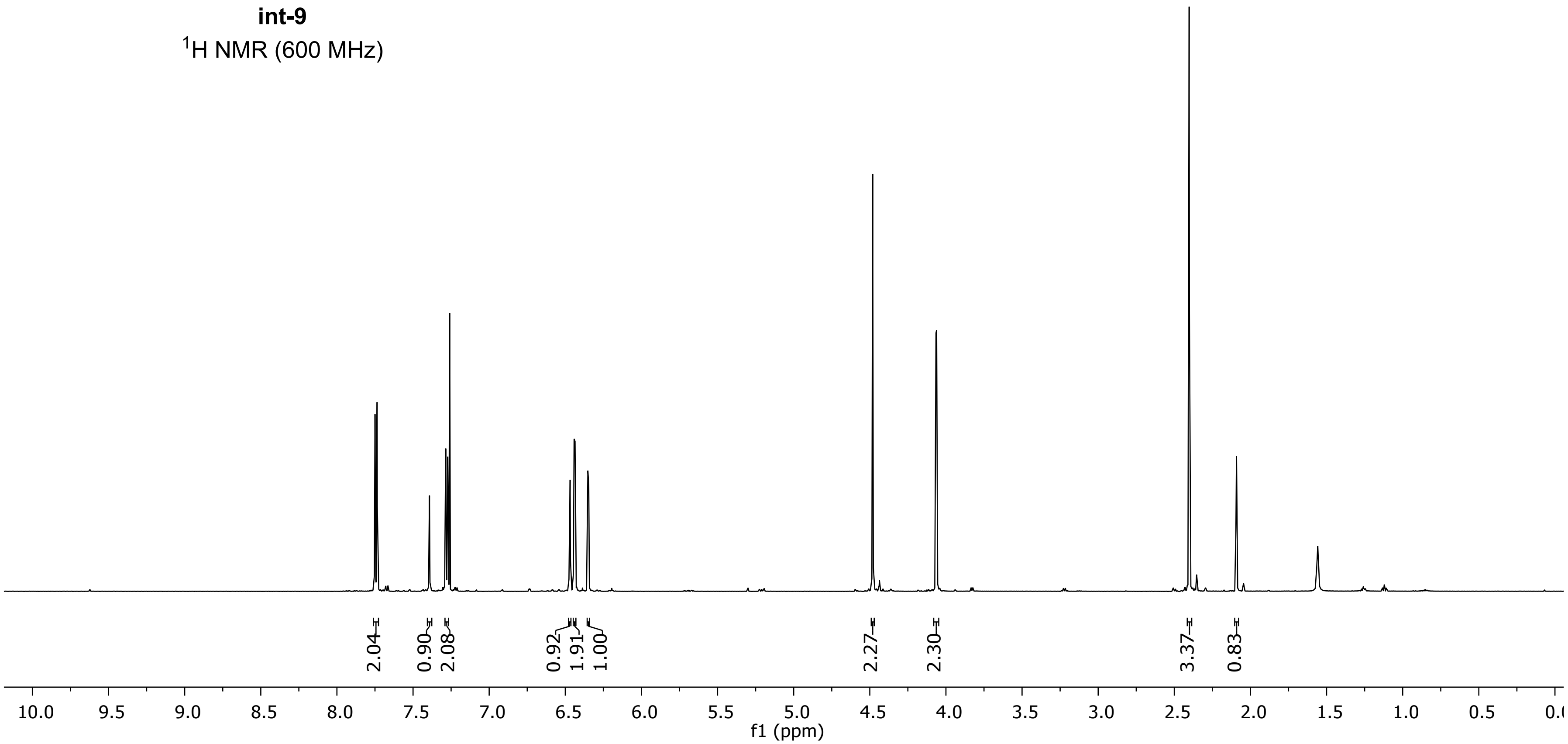

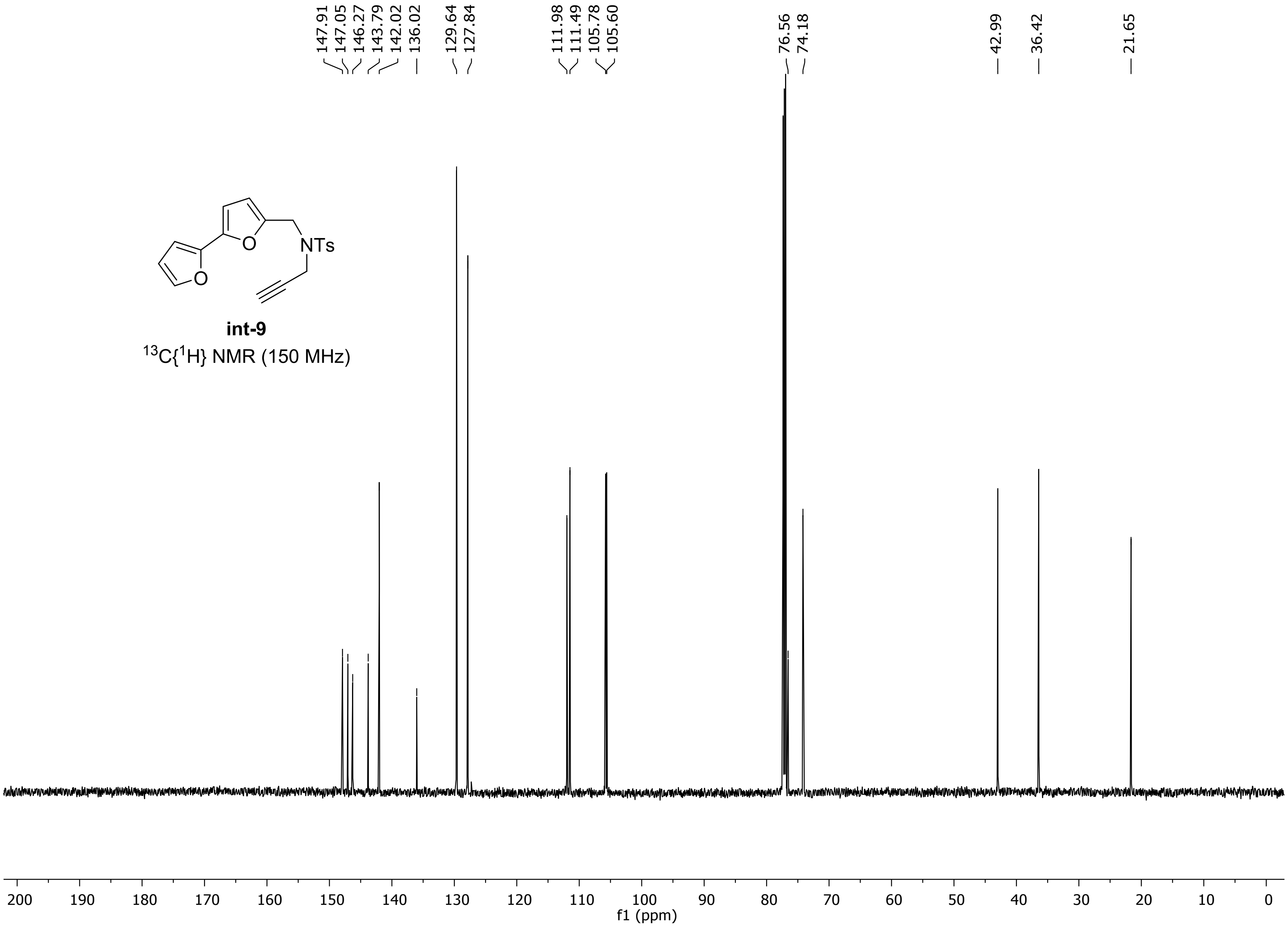

int-9

$^{13}C\{^{1}H\}$ NMR (150 MHz)

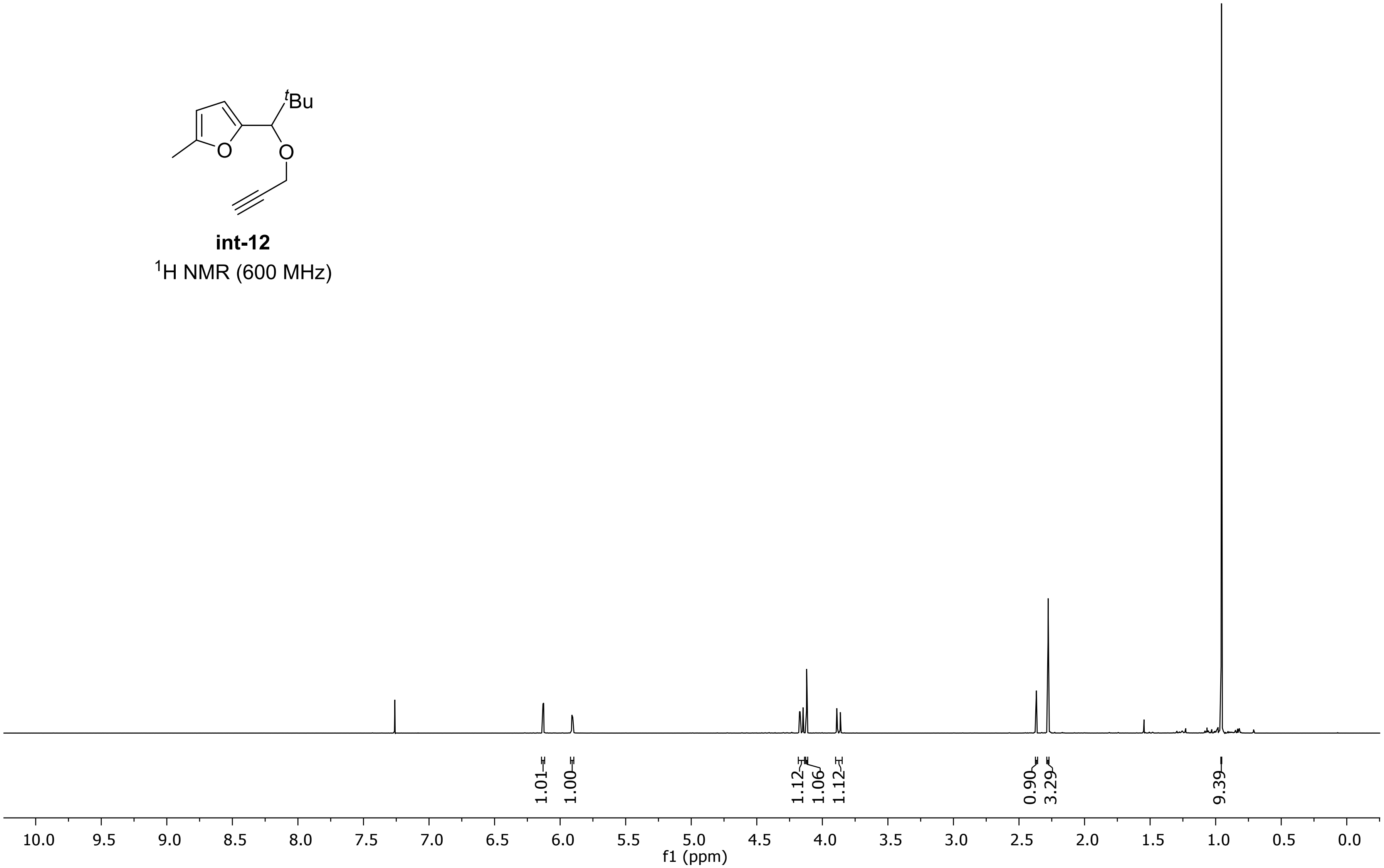

int-12

^{1}H NMR (600 MHz)

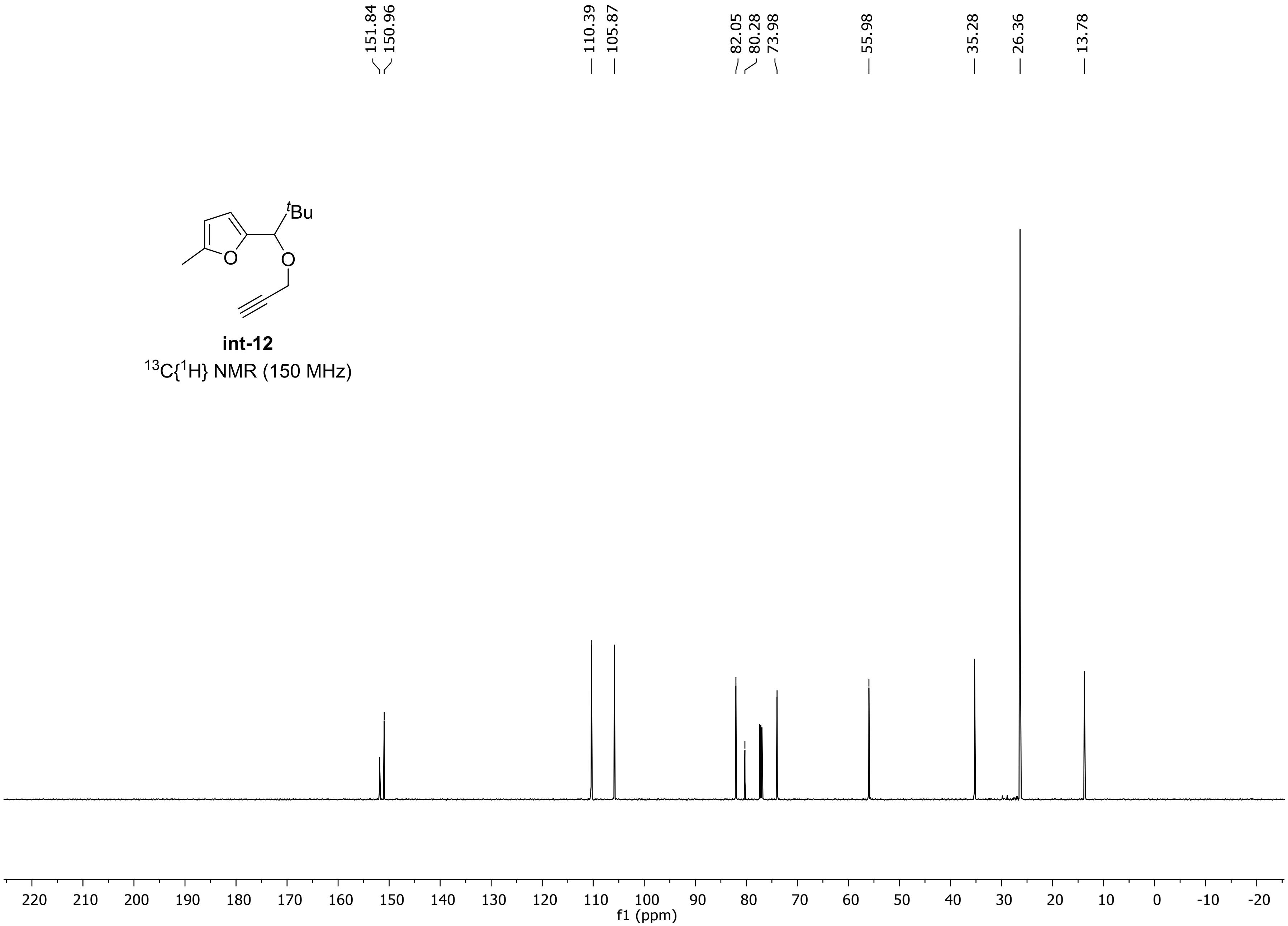

int-12

$^{13}C\{^{1}H\}$ NMR (150 MHz)

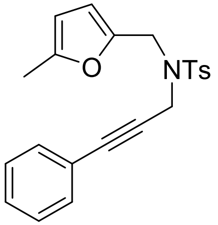

1a

^{1}H NMR (600 MHz)

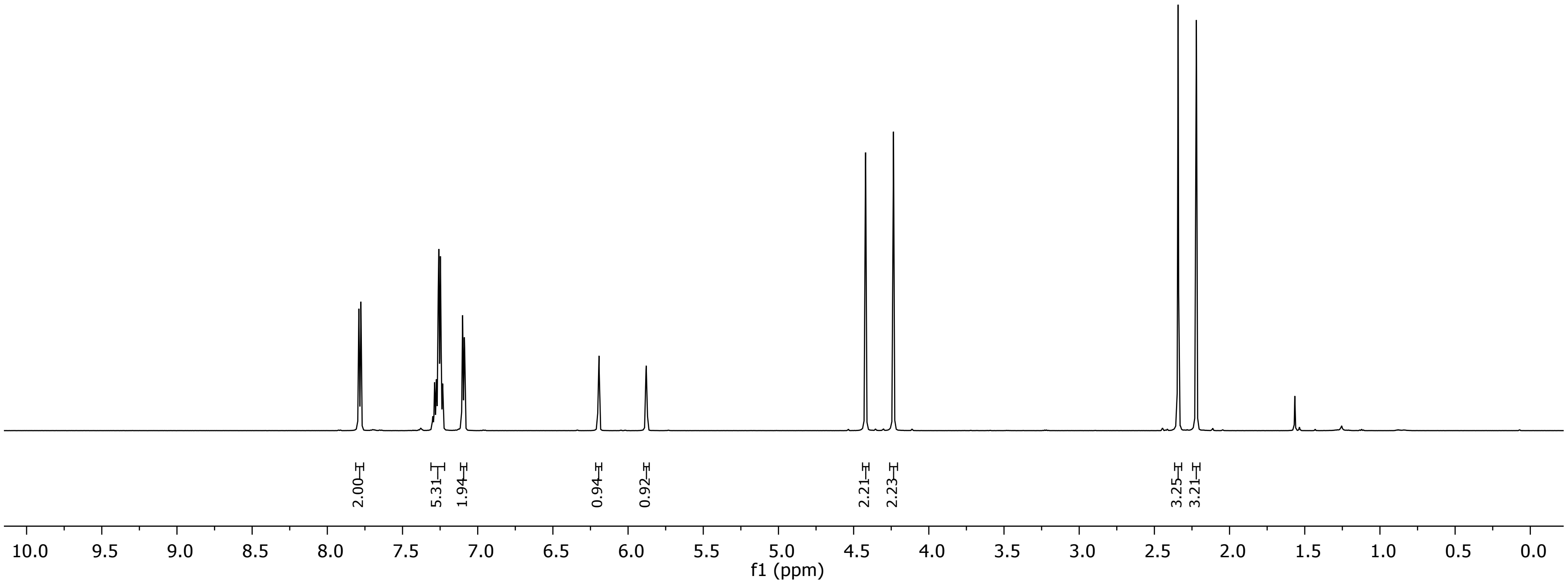

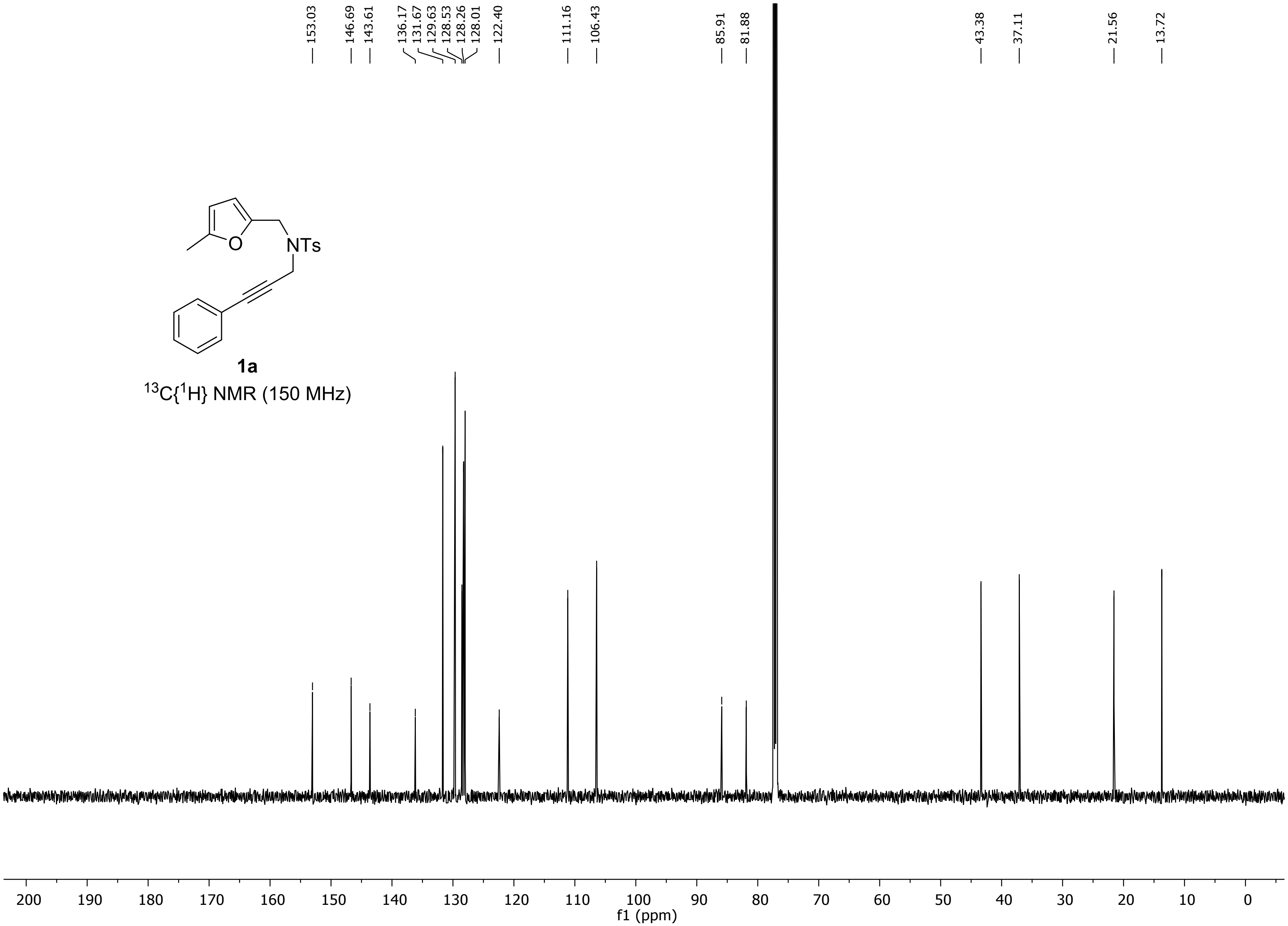

1a

$^{13}C\{^{1}H\}$ NMR (150 MHz)

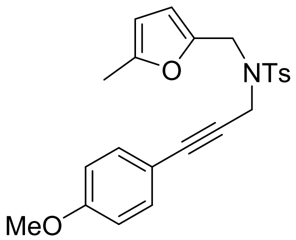

1b

^{1}H NMR (600 MHz)

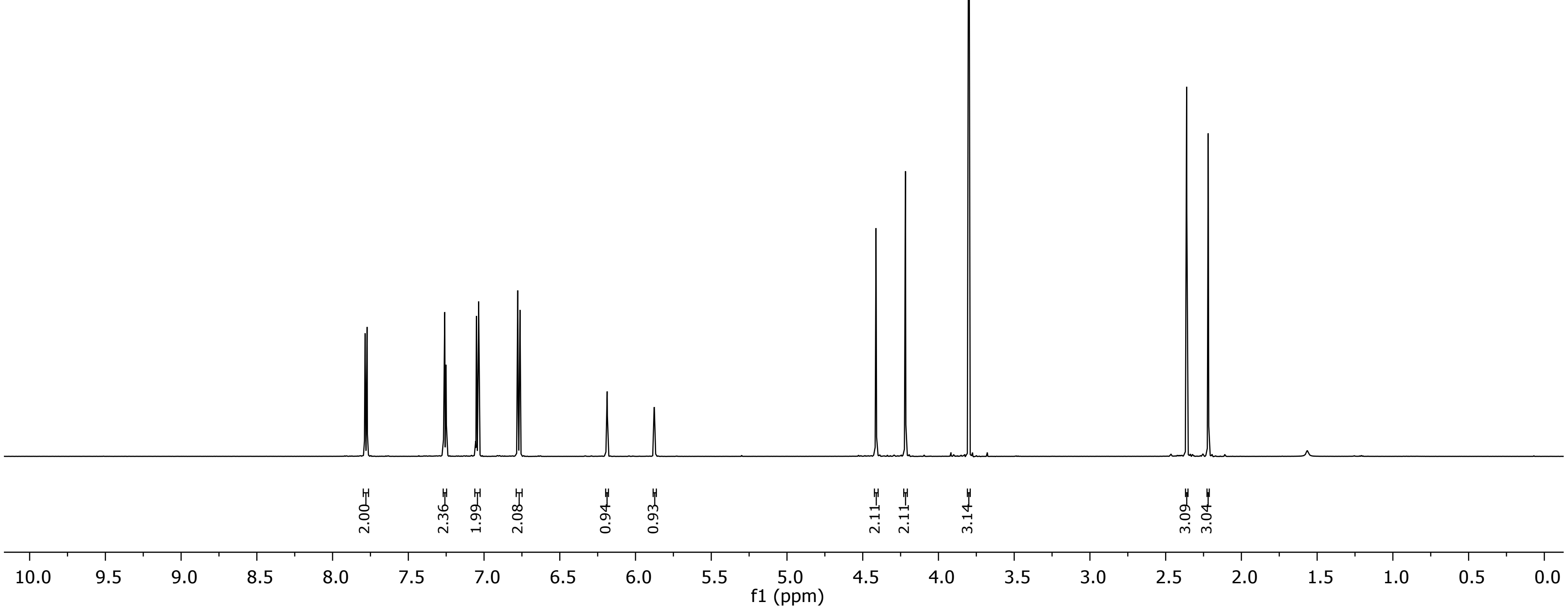

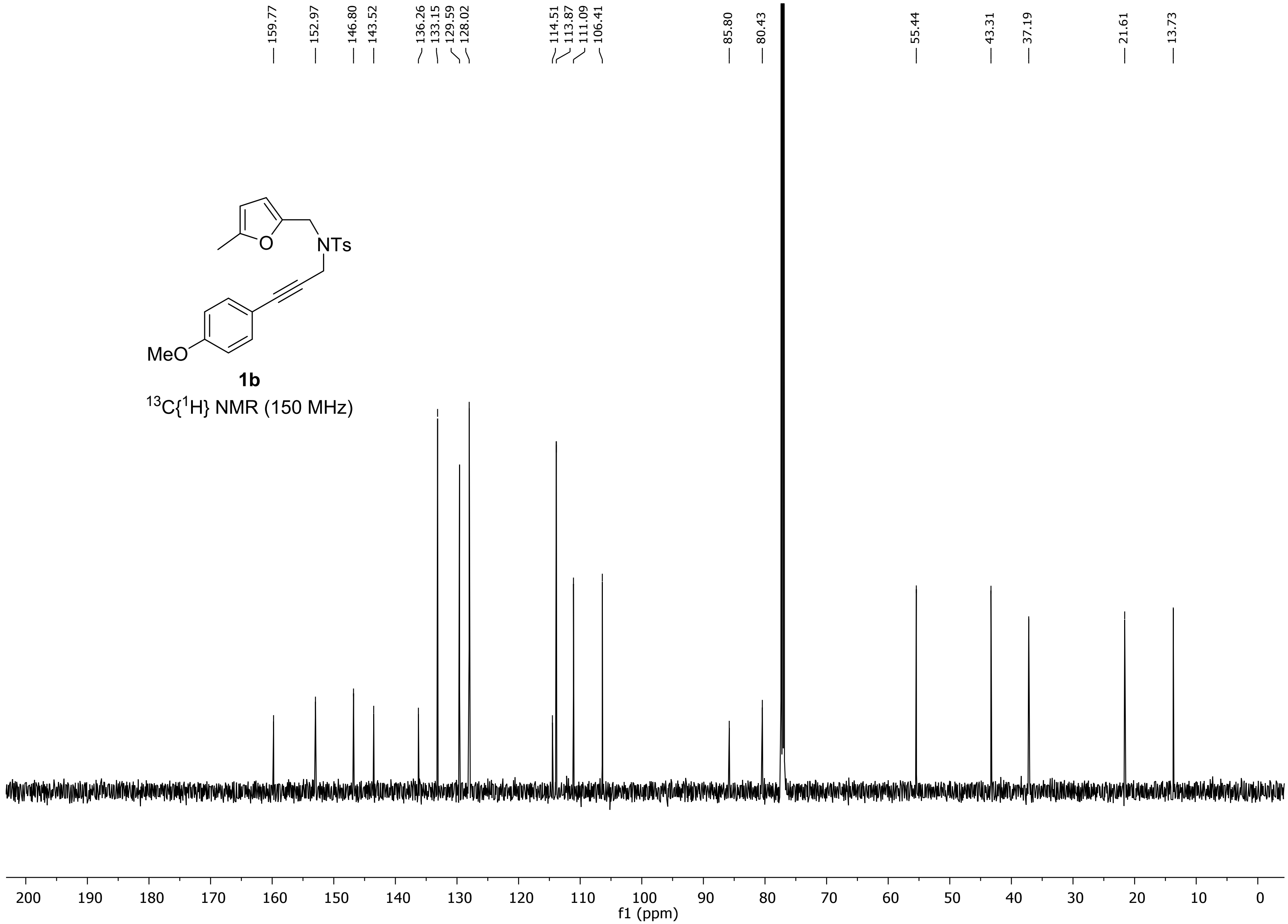
159.77
152.97
146.80
143.52
136.26
133.15
129.59
128.02
114.51
113.87
111.09
106.41
85.80
80.43
55.44
43.31
37.19
21.61
13.73
O
NTs
MeO
1b
13C{1H} NMR (150 MHz)
200 190 180 170 160 150 140 130 120 110 100 90 80 70 60 50 40 30 20 10 0
f1 (ppm)

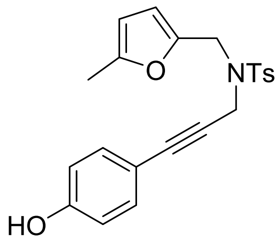

1c

^{1}H NMR (600 MHz)

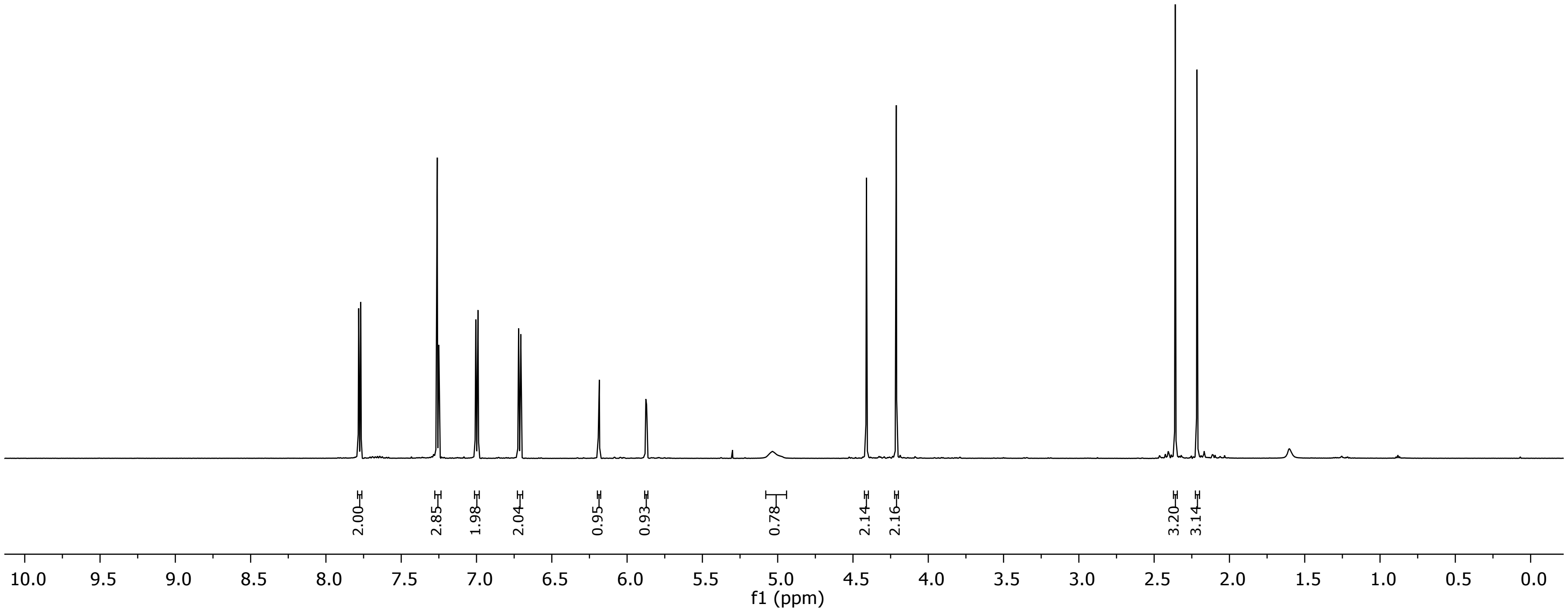

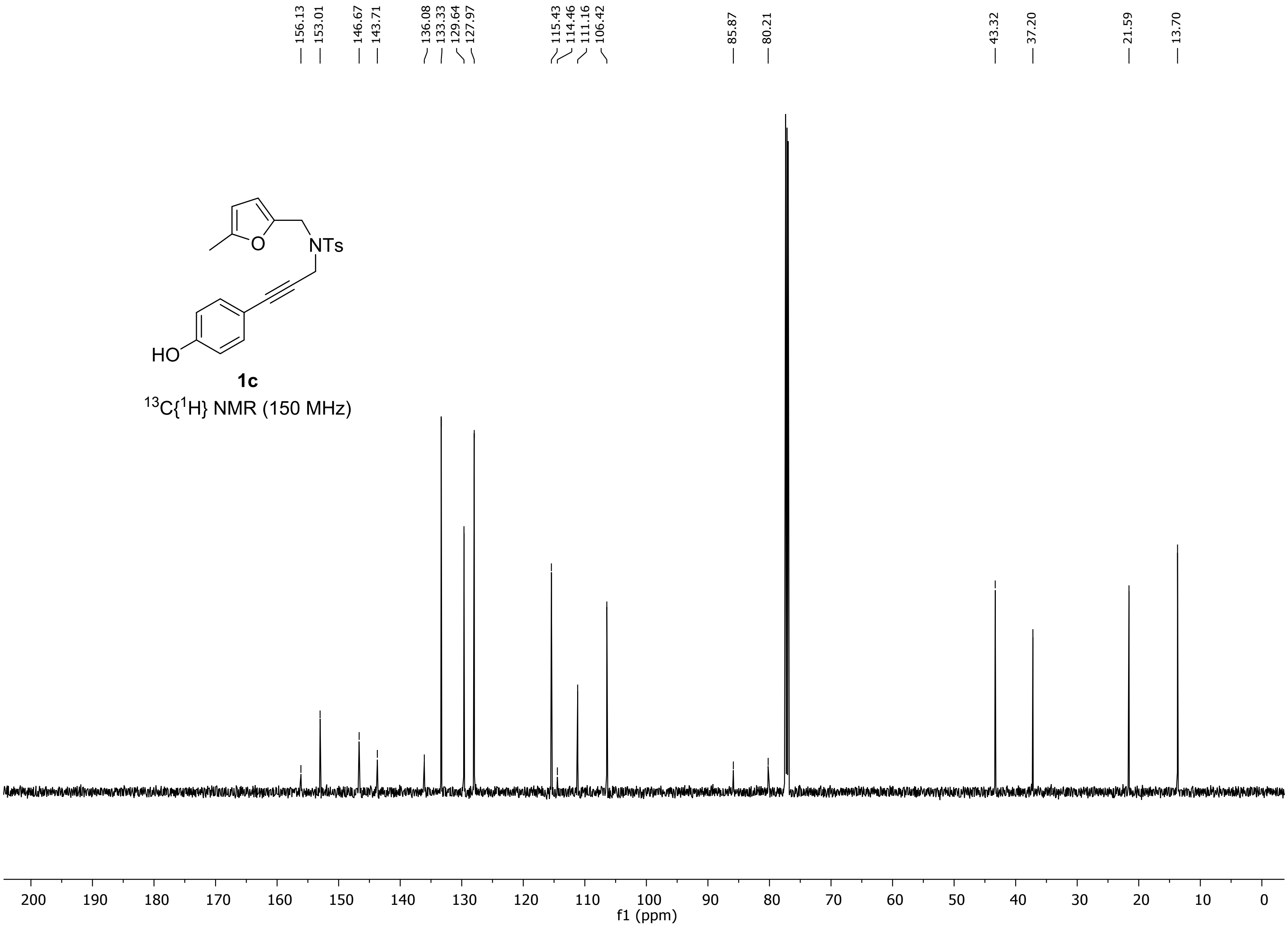

1c

^{13}C{^{1}H} NMR (150 MHz)

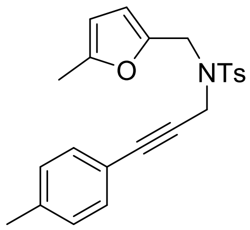

1d

^{1}H NMR (600 MHz)

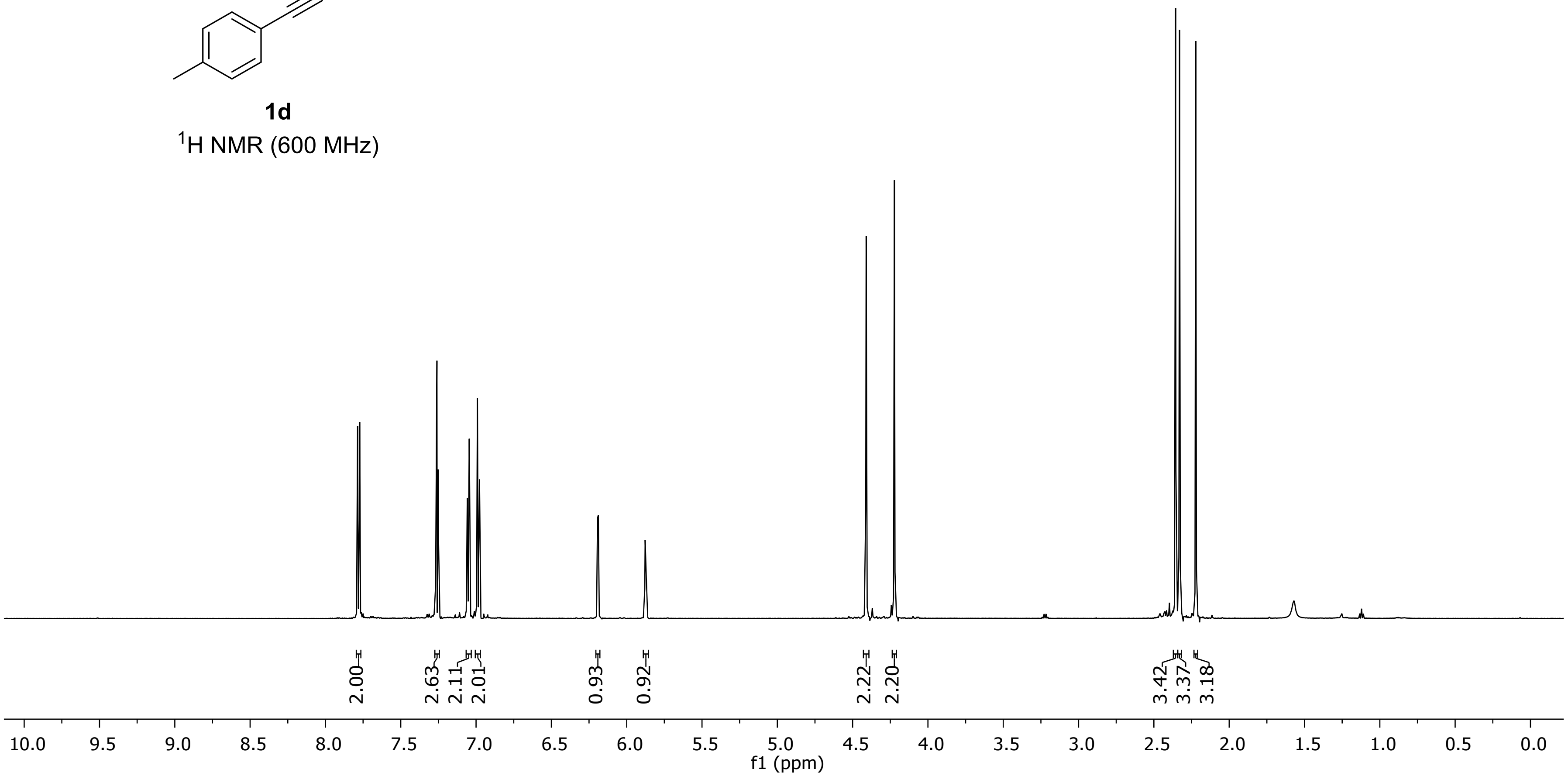

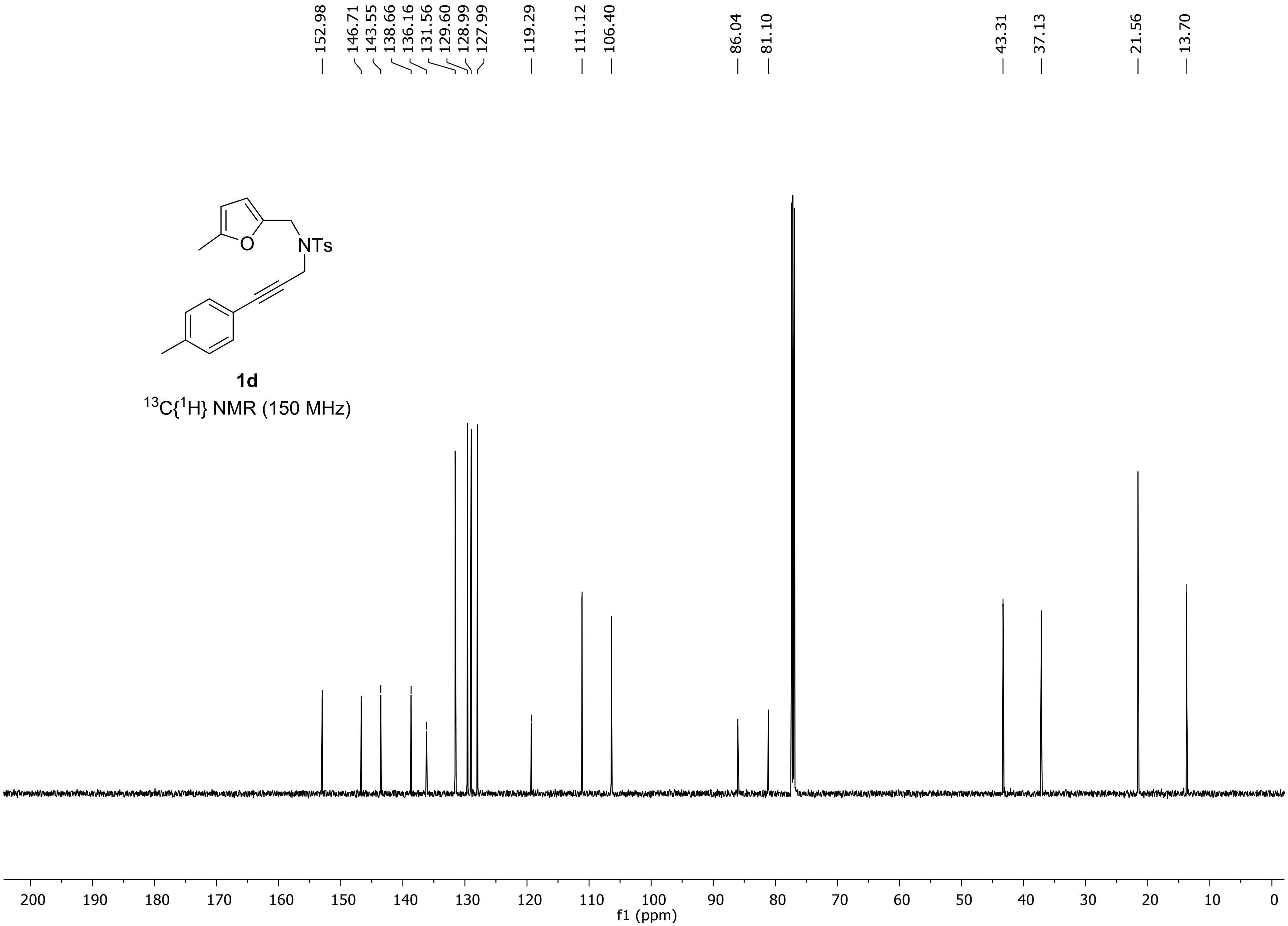

1d

$^{13}C\{^{1}H\}$ NMR (150 MHz)

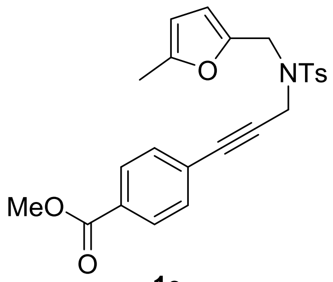

1e

^{1}H NMR (600 MHz)

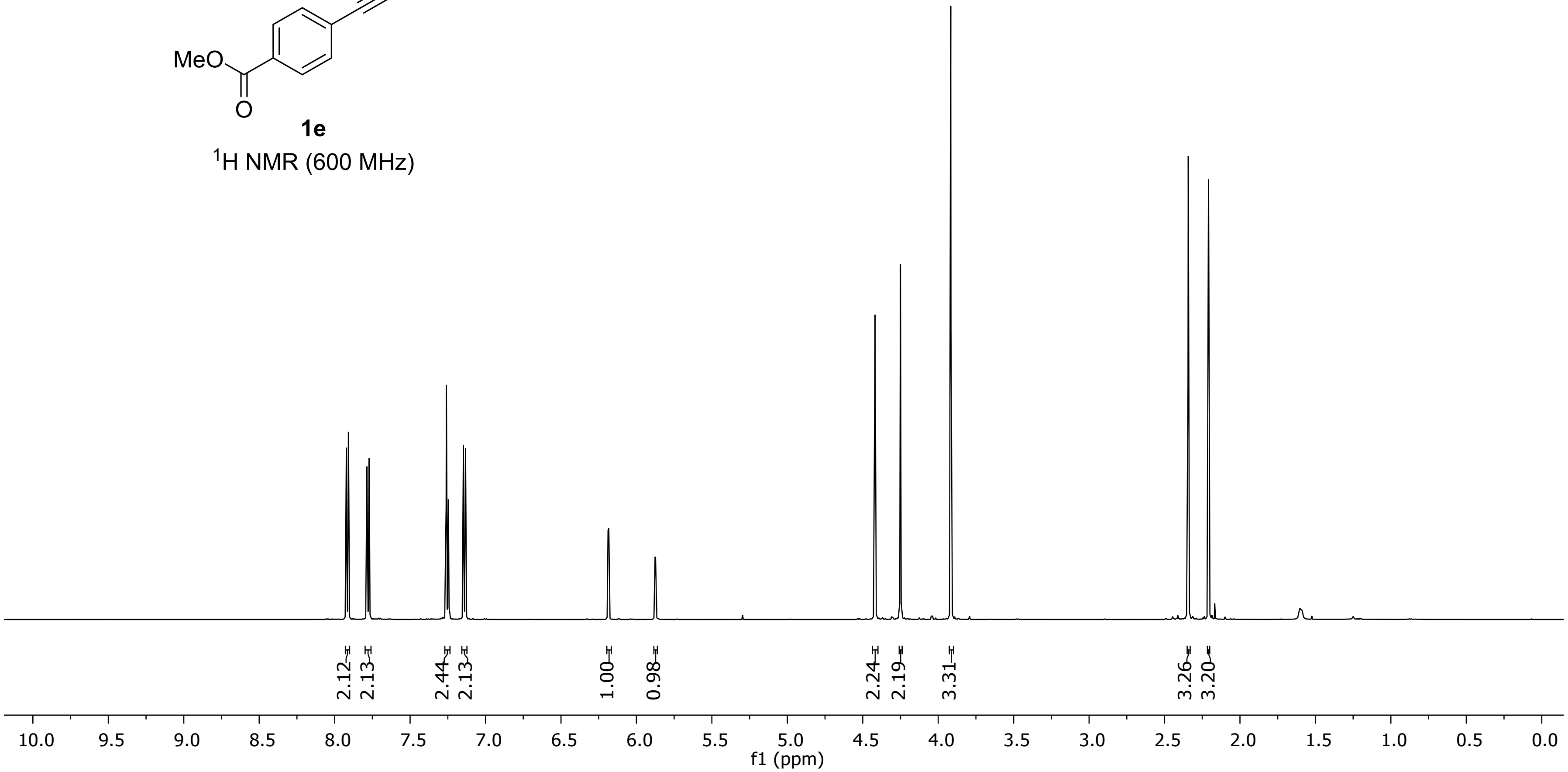

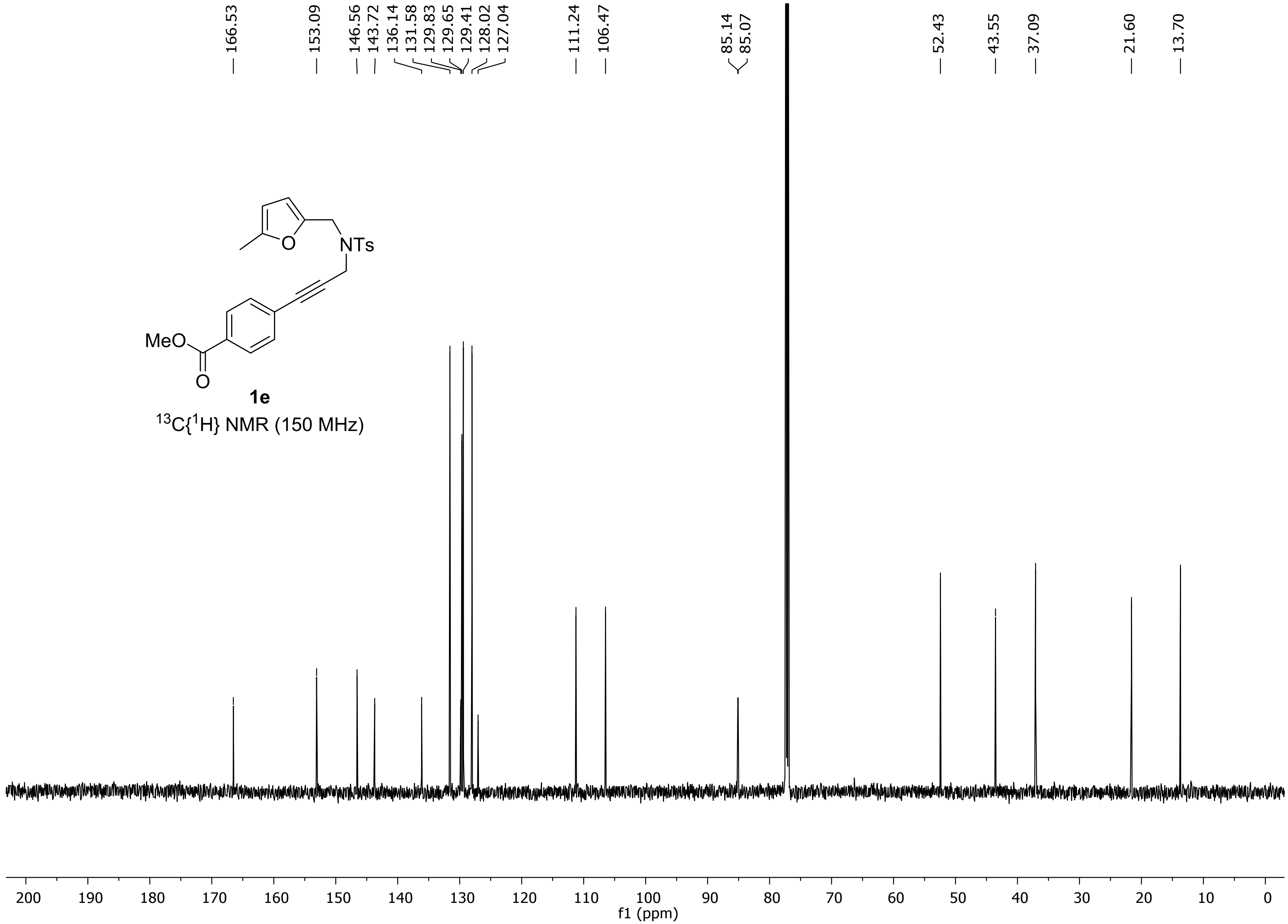

1e

$^{13}C\{^{1}H\}$ NMR (150 MHz)

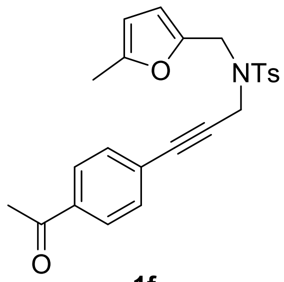

1f

^{1}H NMR (600 MHz)

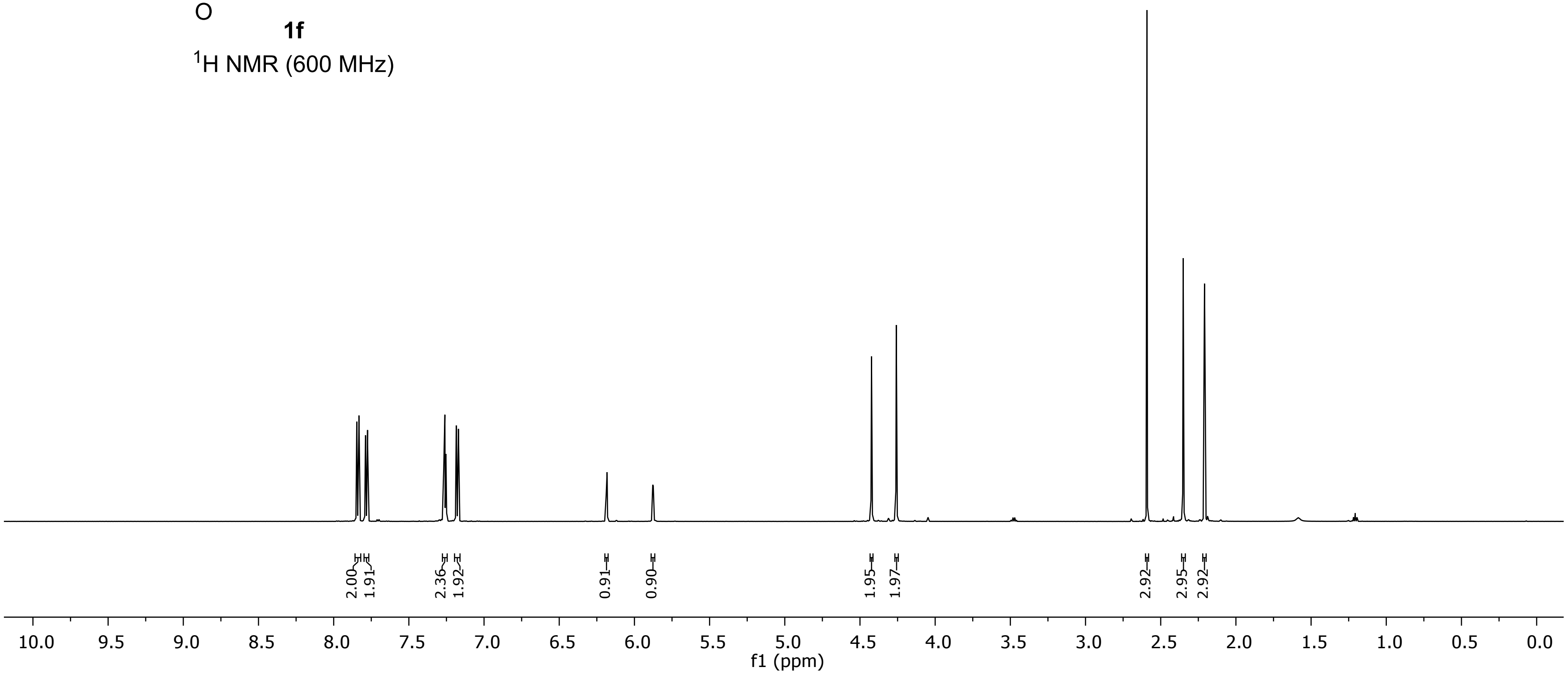

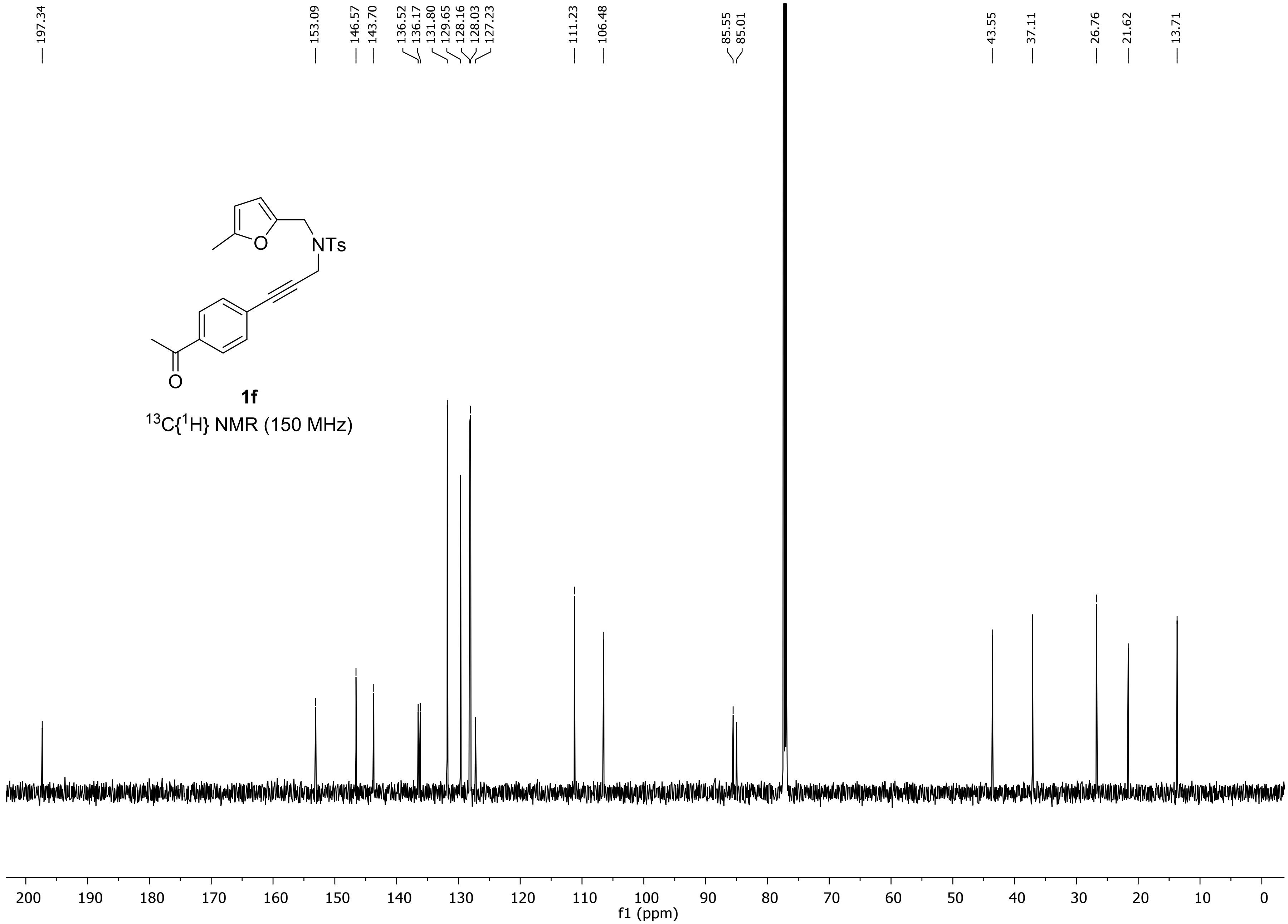
197.34
153.09
146.57
143.70
136.52
136.17
131.80
129.65
128.16
128.03
127.23
111.23
106.48
85.55
85.01
43.55
37.11
26.76
21.62
13.71
NTs
O
1f
$^{13}C\{^{1}H\}$ NMR (150 MHz)
200
190
180
170
160
150
140
130
120
110
100
90
80
70
60
50
40
30
20
10
0
f1 (ppm)

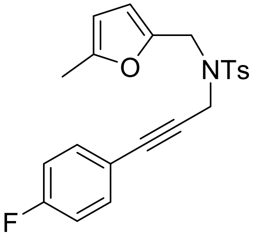

1g

^{1}H NMR (600 MHz)

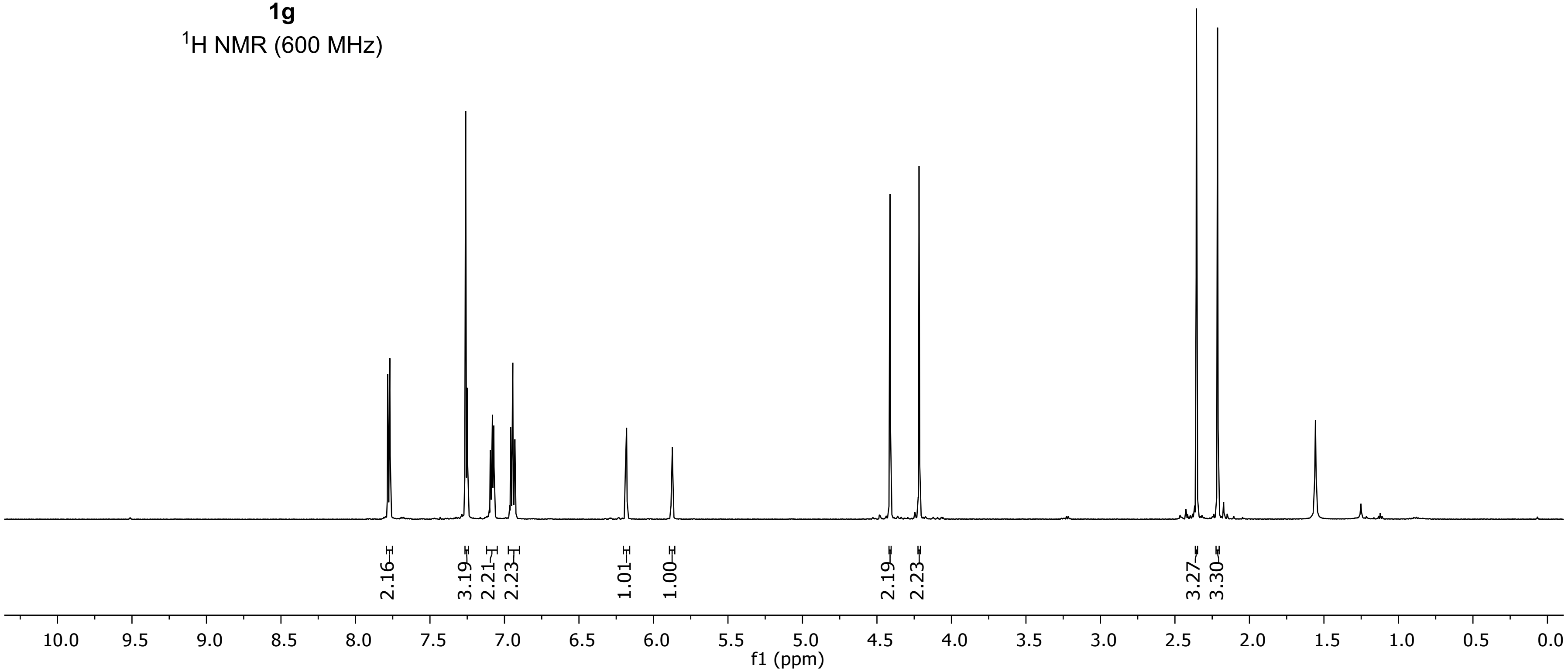

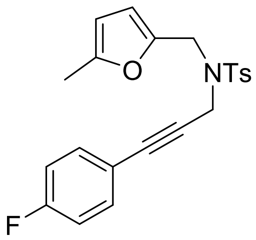

1g

$^{13}C\{^1H\}$ NMR (150 MHz)

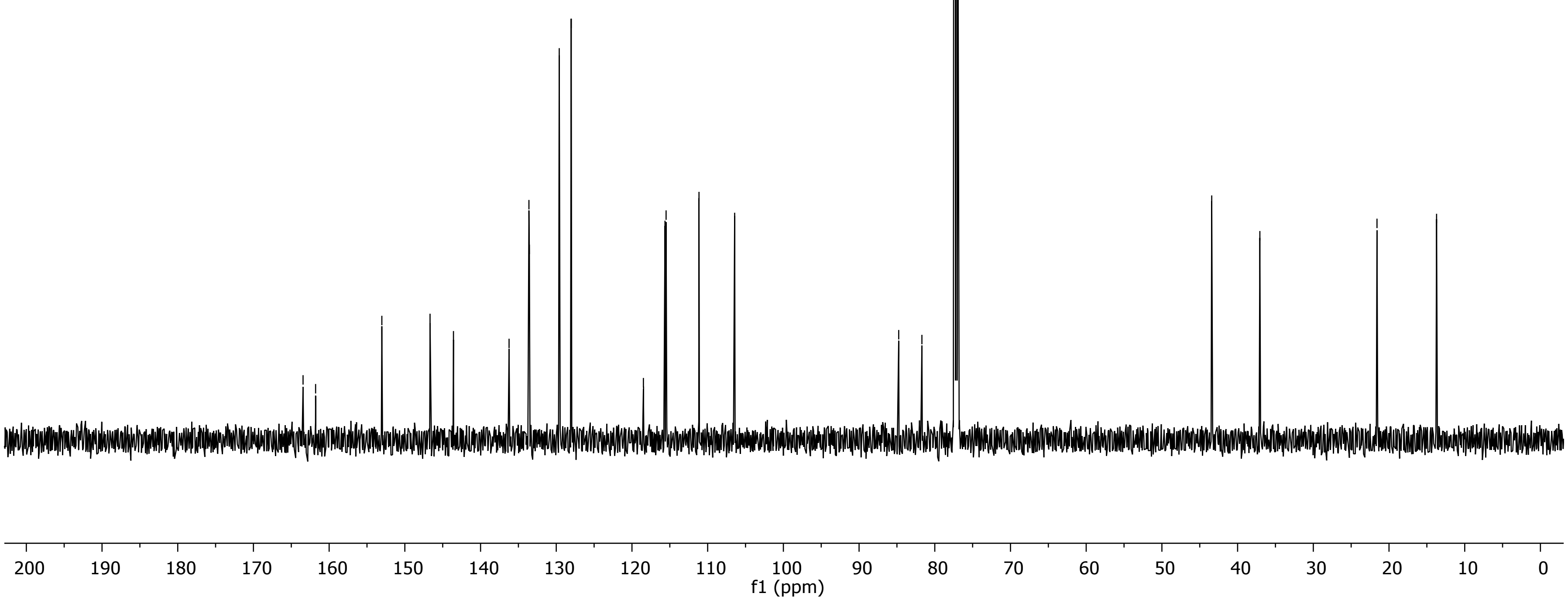

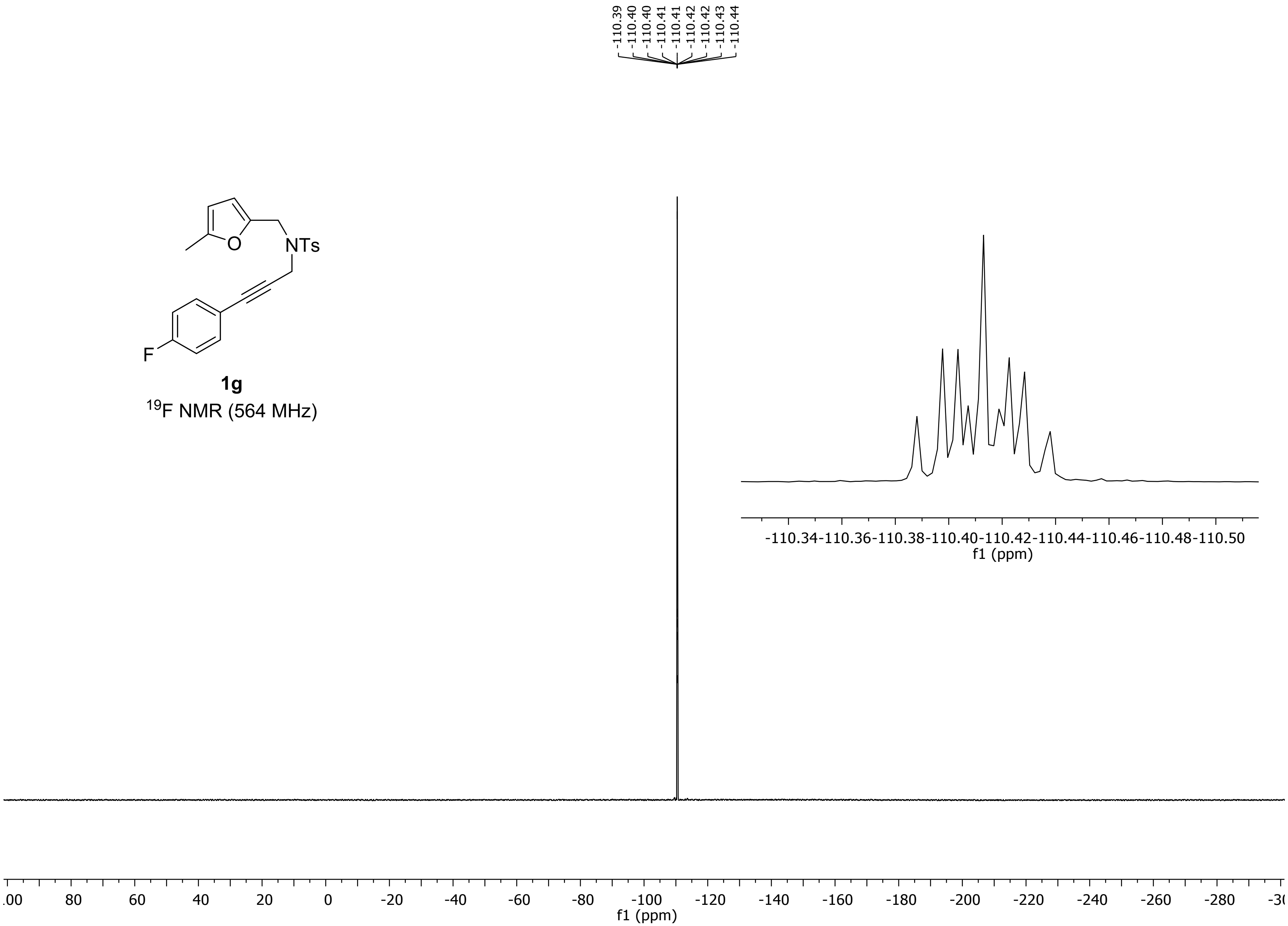

1g

^{19}F NMR (564 MHz)

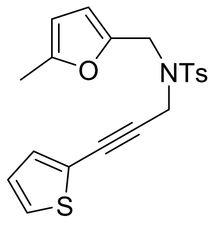

1h

^{1}H NMR (600 MHz)

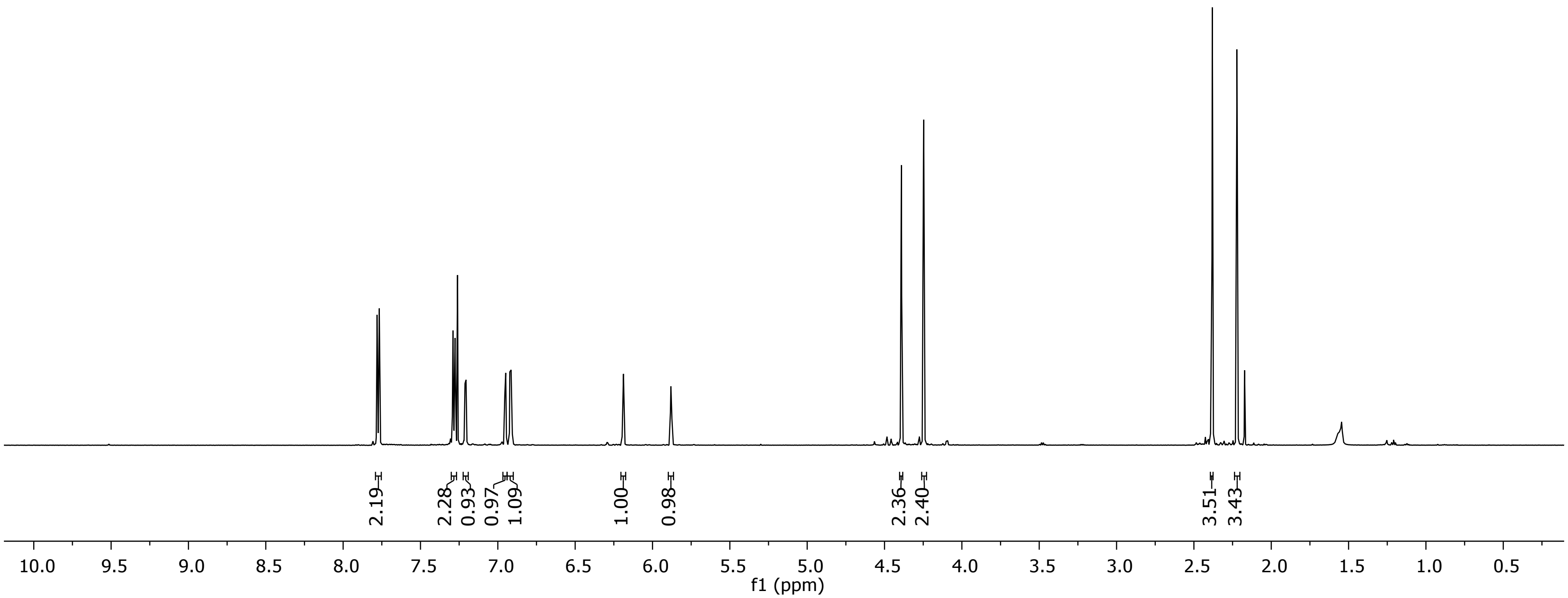

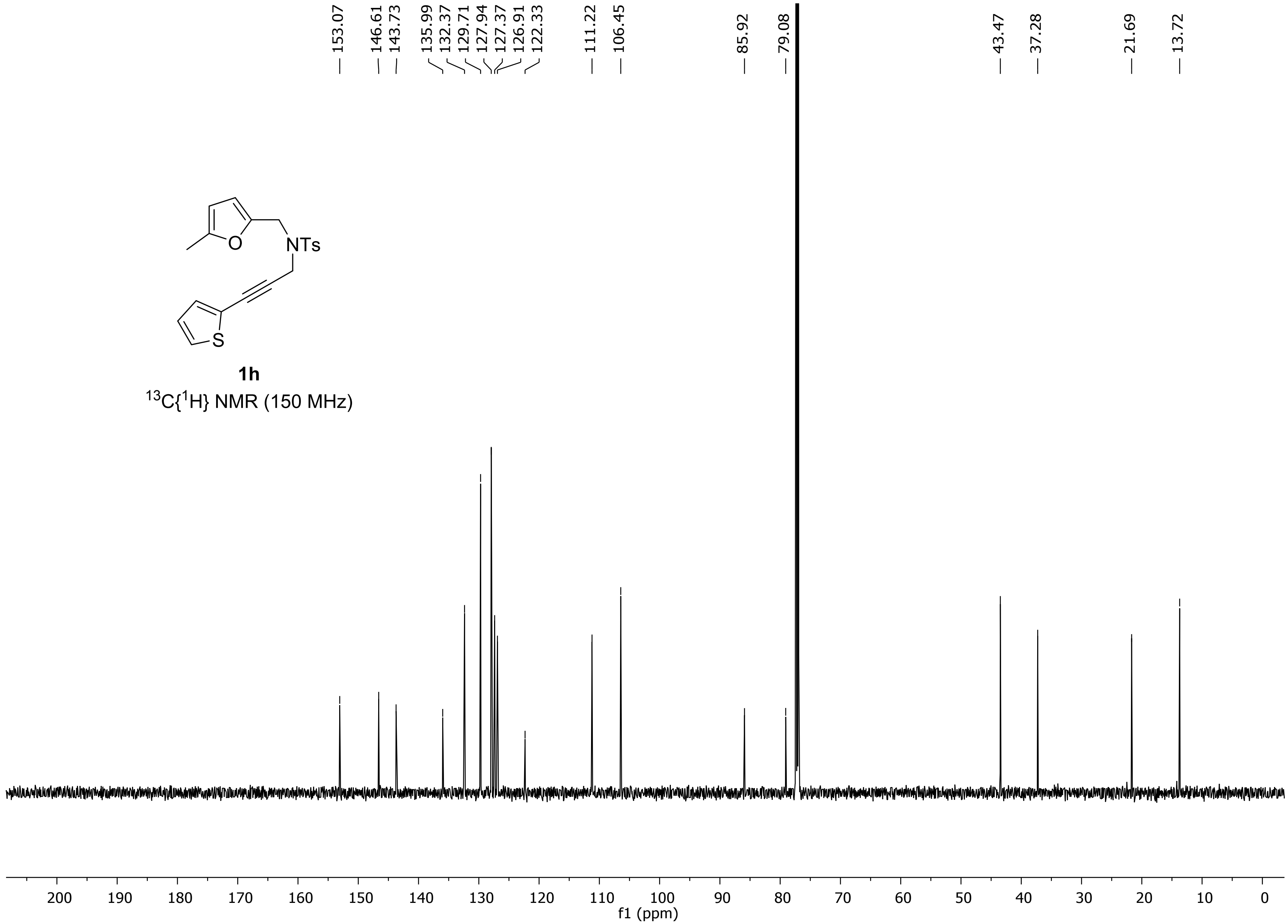

153.07
146.61
143.73
135.99
132.37
129.71
127.94
127.37
126.91
122.33
111.22
106.45
85.92
79.08
43.47
37.28
21.69
13.72
NTs
O
S
1h
$^{13}C\{^1H\}$ NMR (150 MHz)
200
190
180
170
160
150
140
130
120
110
100
90
80
70
60
50
40
30
20
10
0
f1 (ppm)

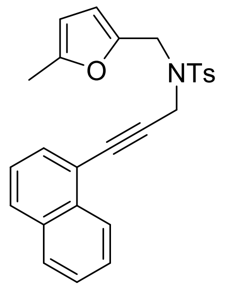

1i

^{1}H NMR (600 MHz)

traces of minor rotamer are present

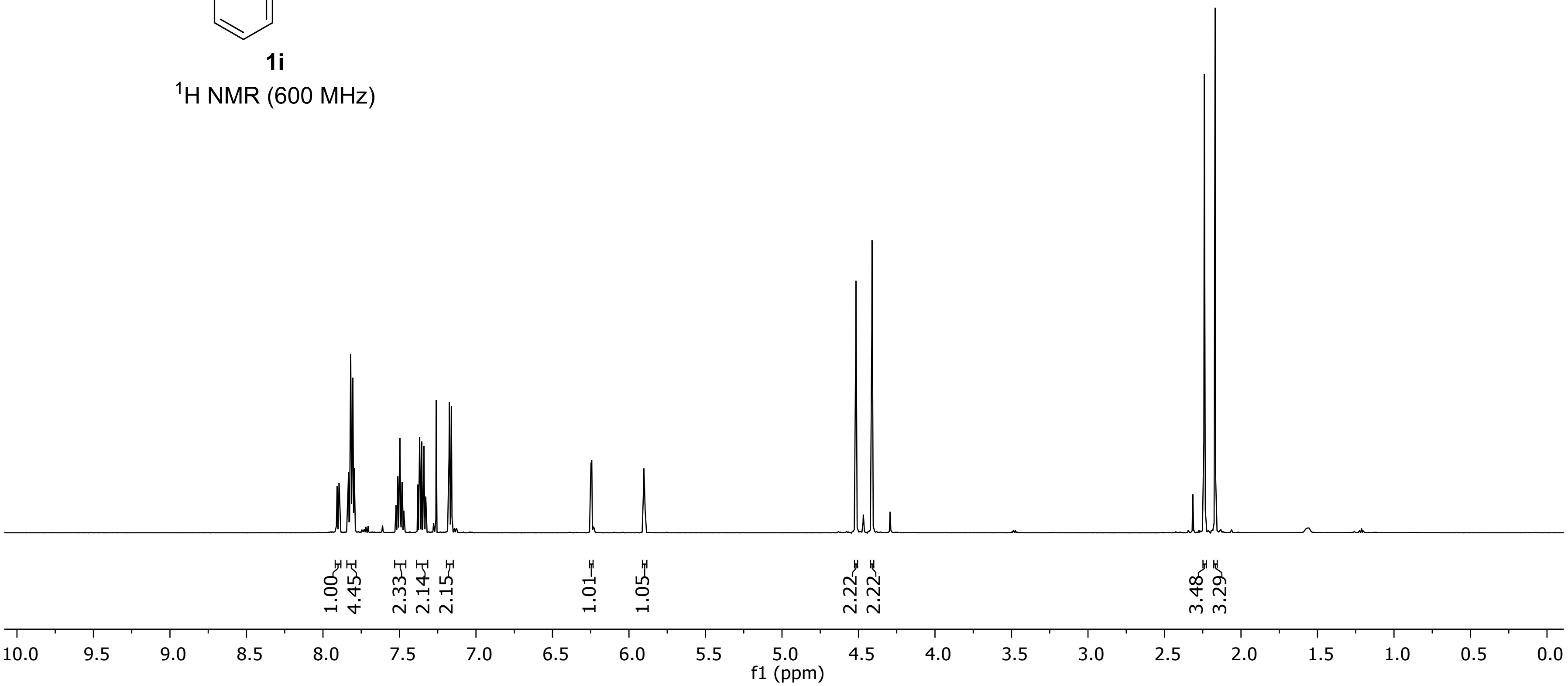

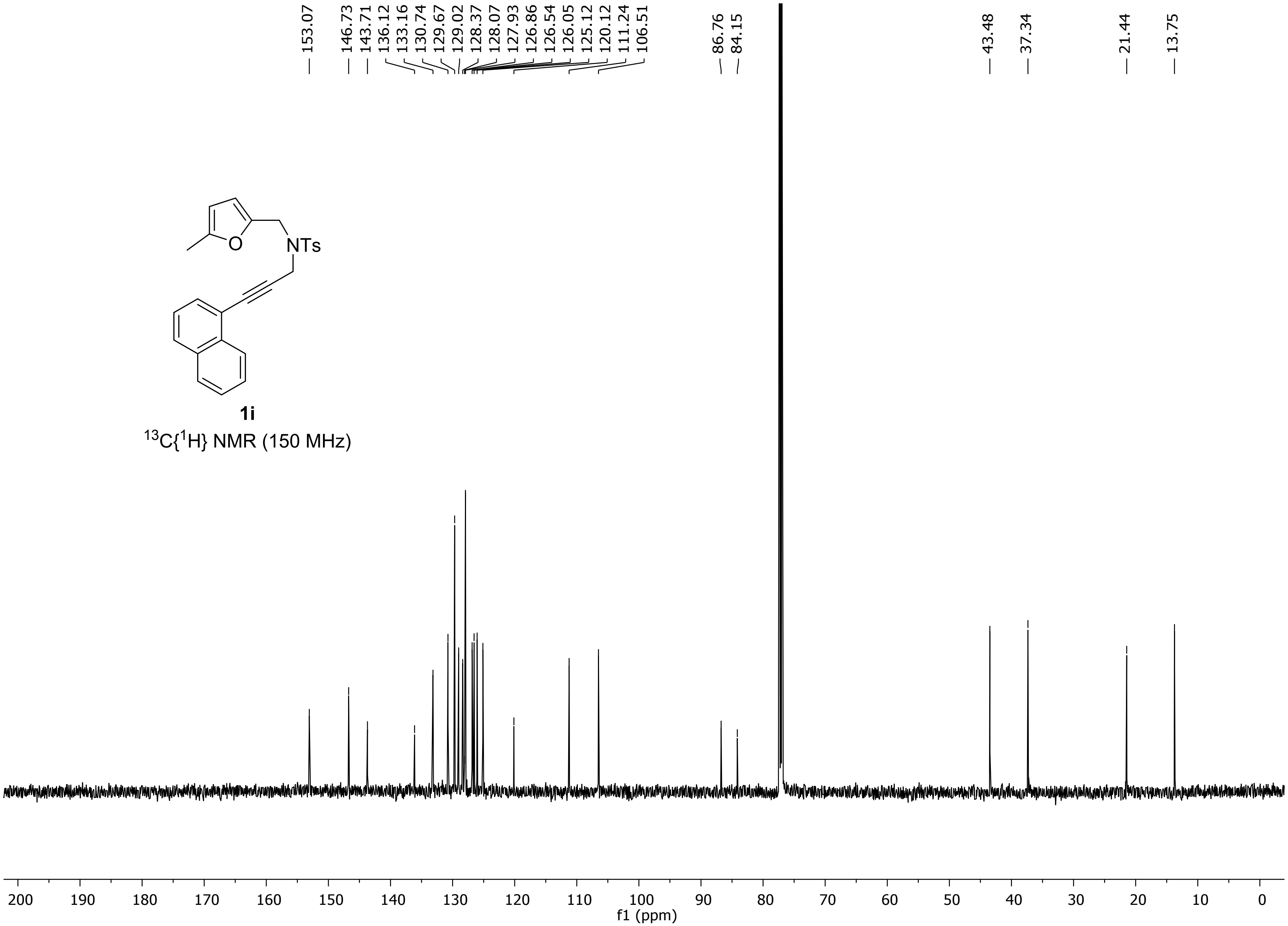

1i

$^{13}C\{^{1}H\}$ NMR (150 MHz)

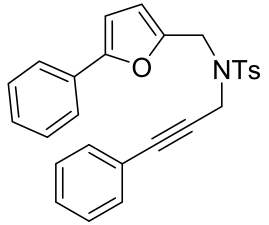

1j
^{1}H NMR (600 MHz)

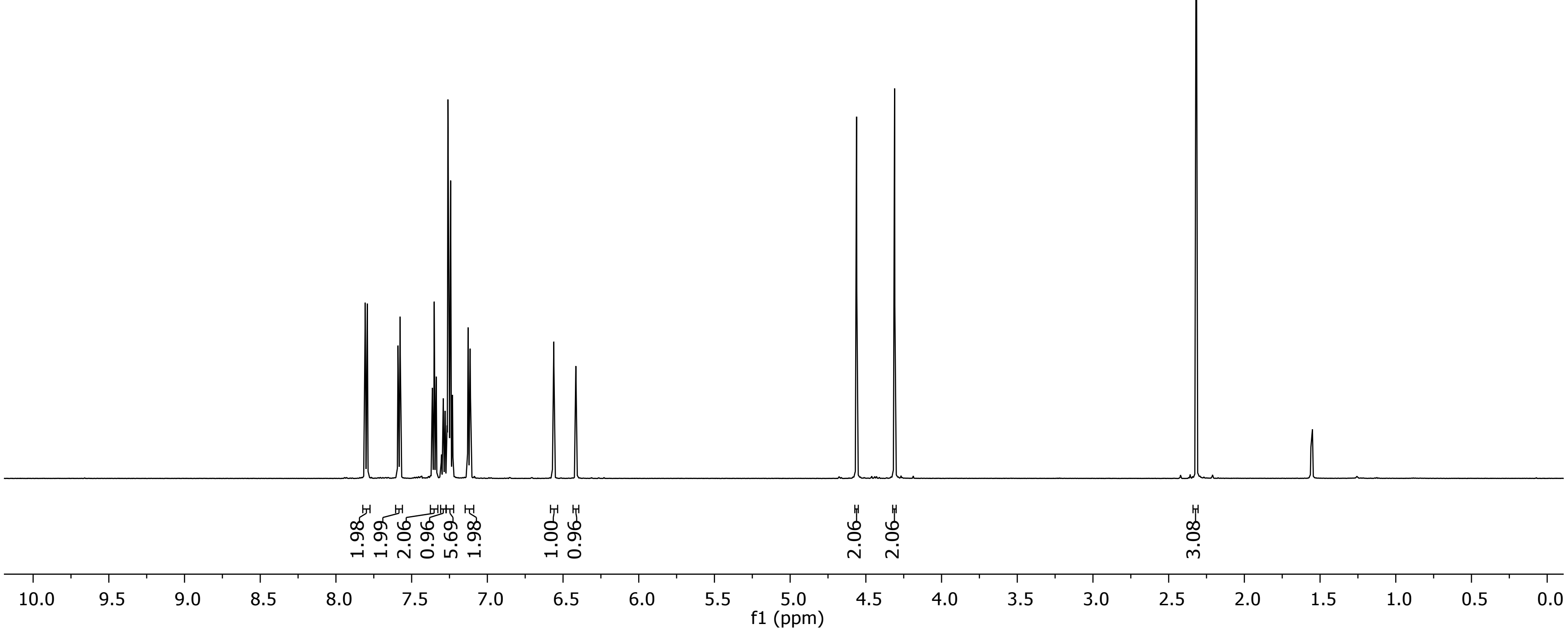

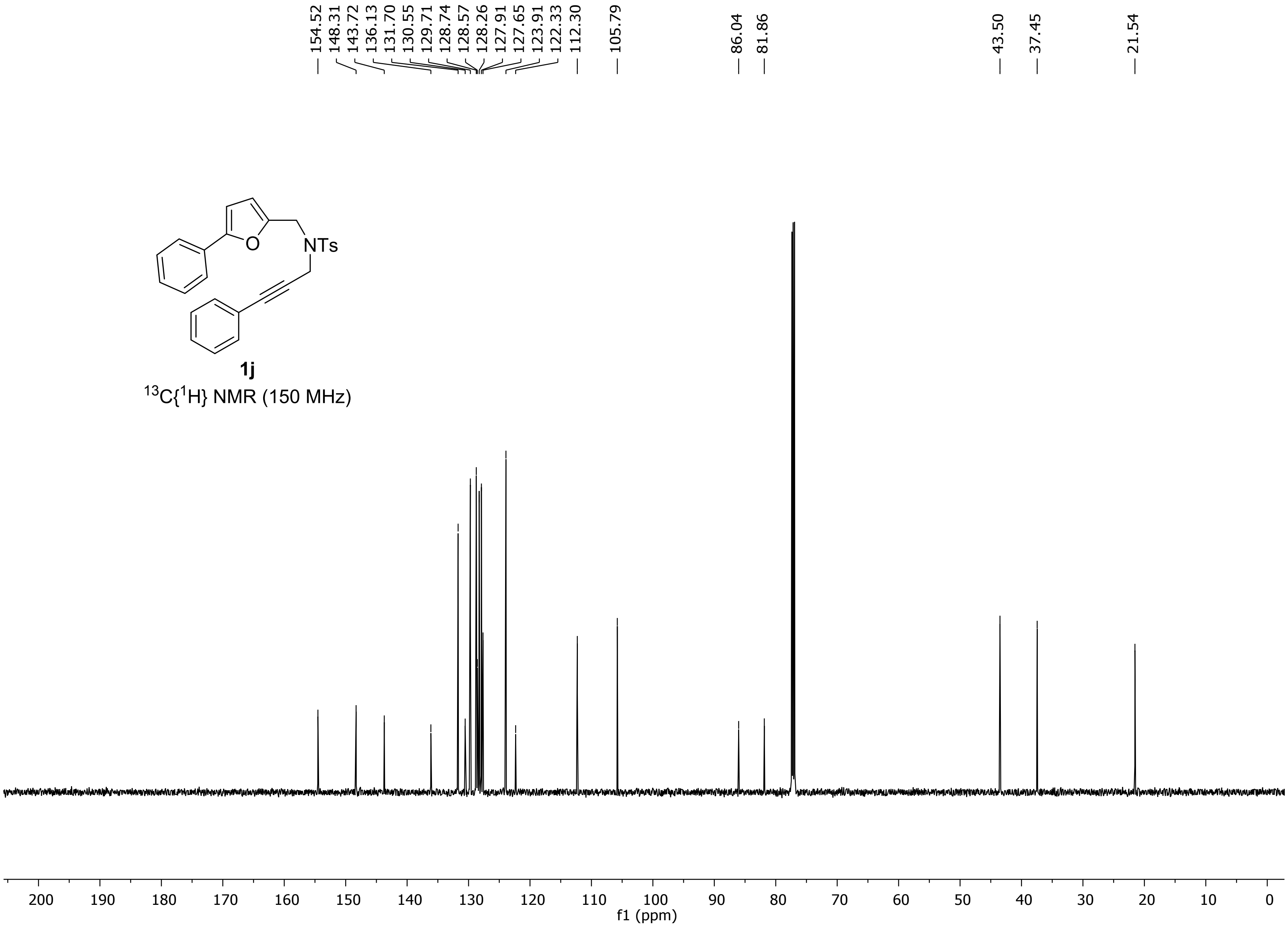
154.52
148.31
143.72
136.13
131.70
130.55
129.71
128.74
128.57
128.26
127.91
127.65
123.91
122.33
112.30
105.79
86.04
81.86
43.50
37.45
21.54
O
NTs
1j
$^{13}C\{^{1}H\}$ NMR (150 MHz)
200
190
180
170
160
150
140
130
120
110
100
90
80
70
60
50
40
30
20
10
0
f1 (ppm)

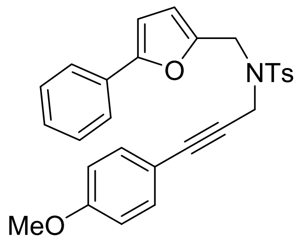

1k

^{1}H NMR (600 MHz)

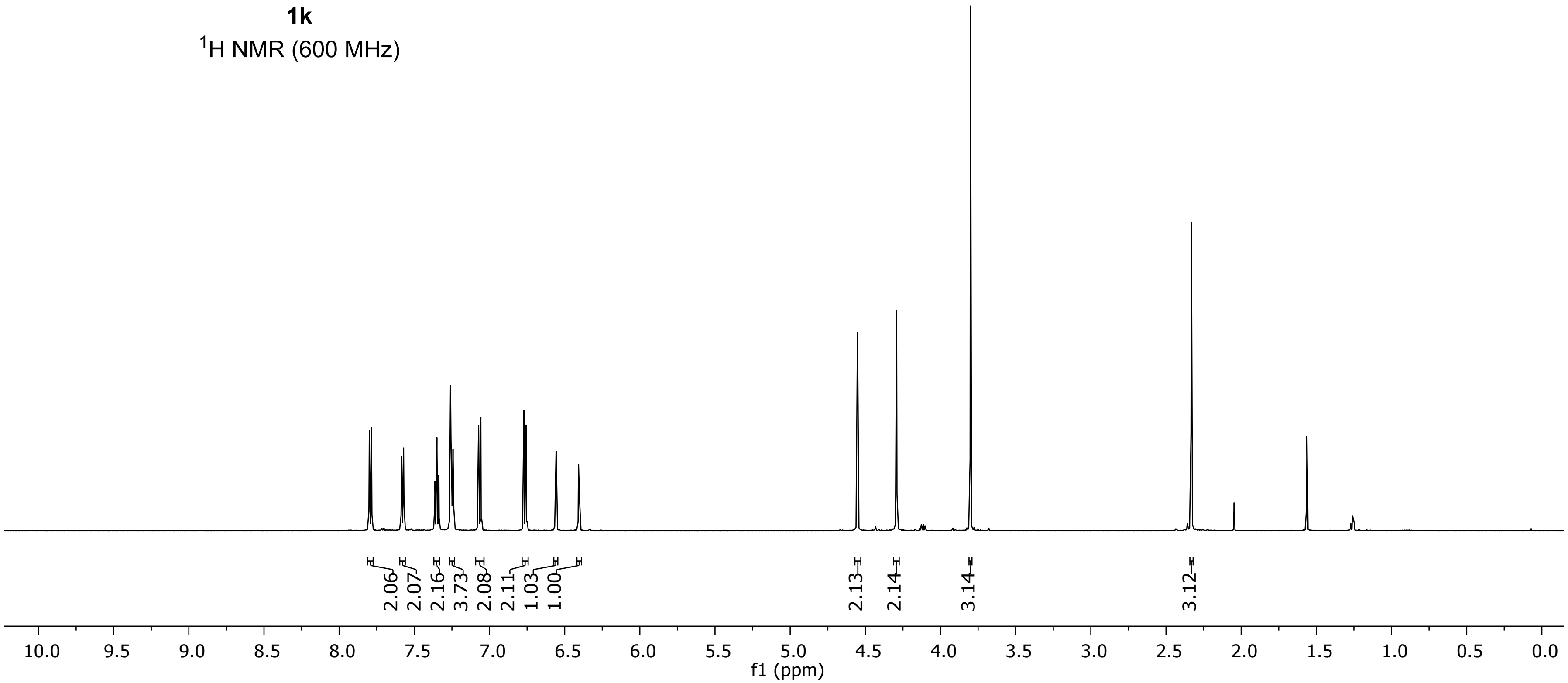

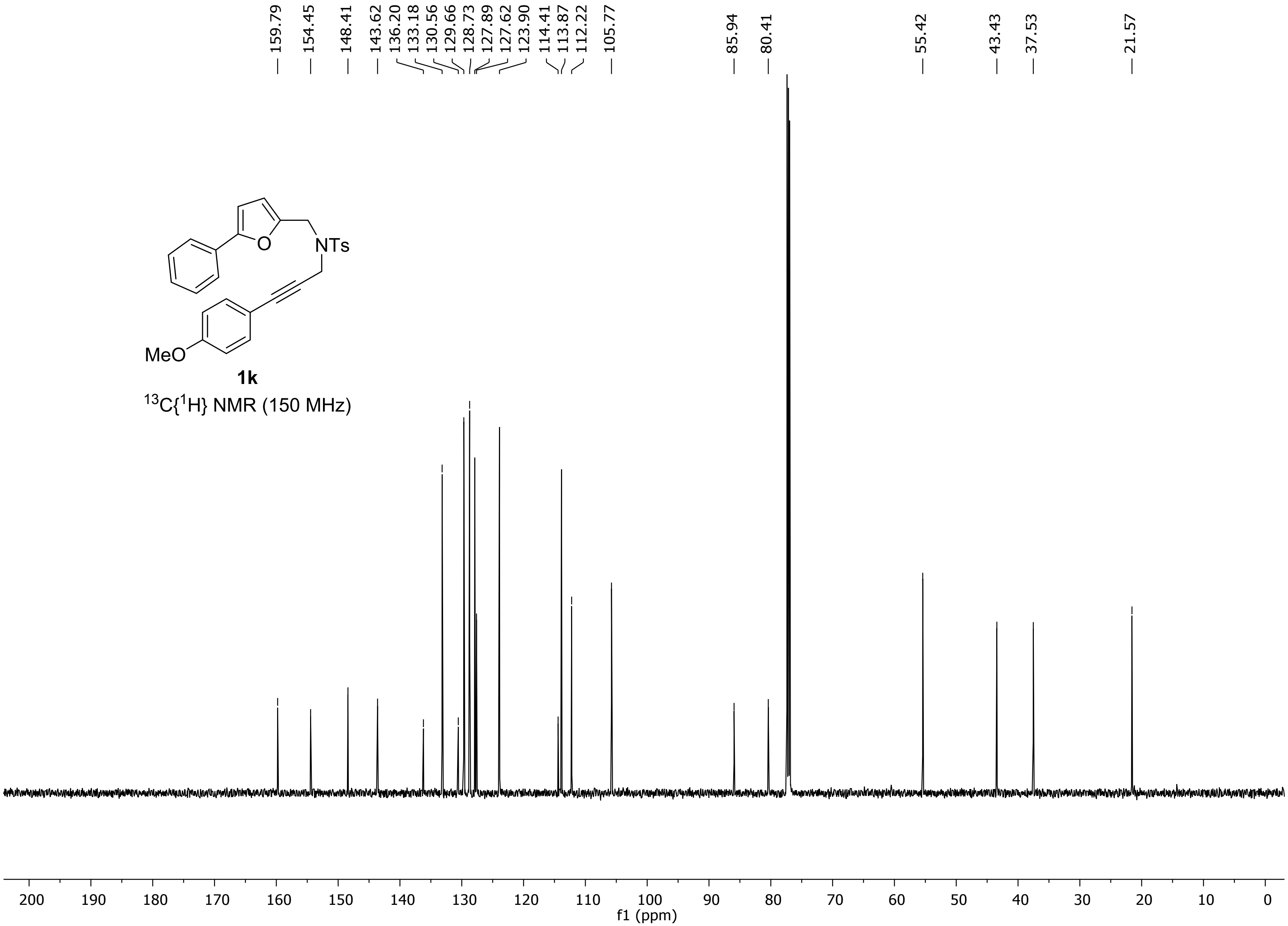
159.79
154.45
148.41
143.62
136.20
133.18
130.56
129.66
128.73
127.89
127.62
123.90
114.41
113.87
112.22
105.77
85.94
80.41
55.42
43.43
37.53
21.57
NTs
O
MeO
1k
13C{1H} NMR (150 MHz)
200
190
180
170
160
150
140
130
120
110
100
90
80
70
60
50
40
30
20
10
0
f1 (ppm)

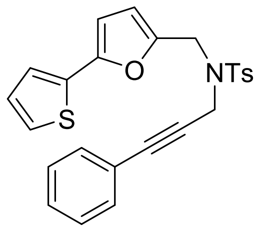

1l

^{1}H NMR (600 MHz)

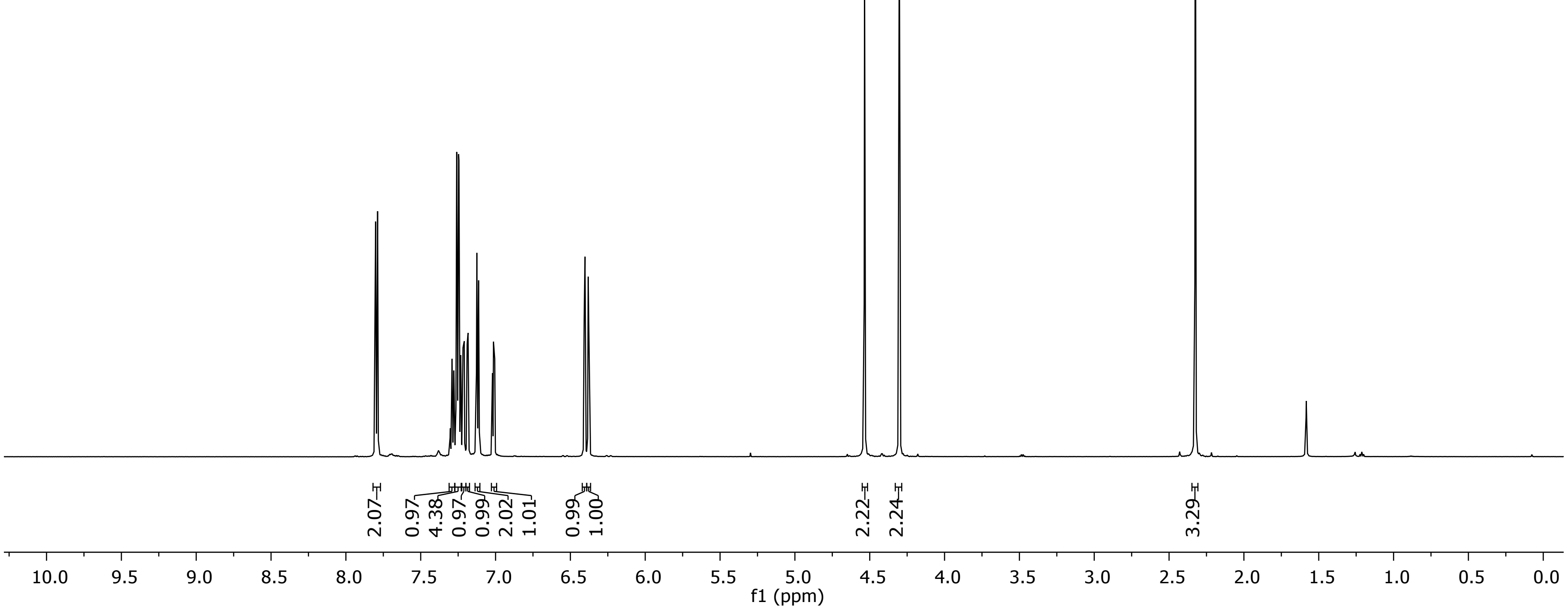

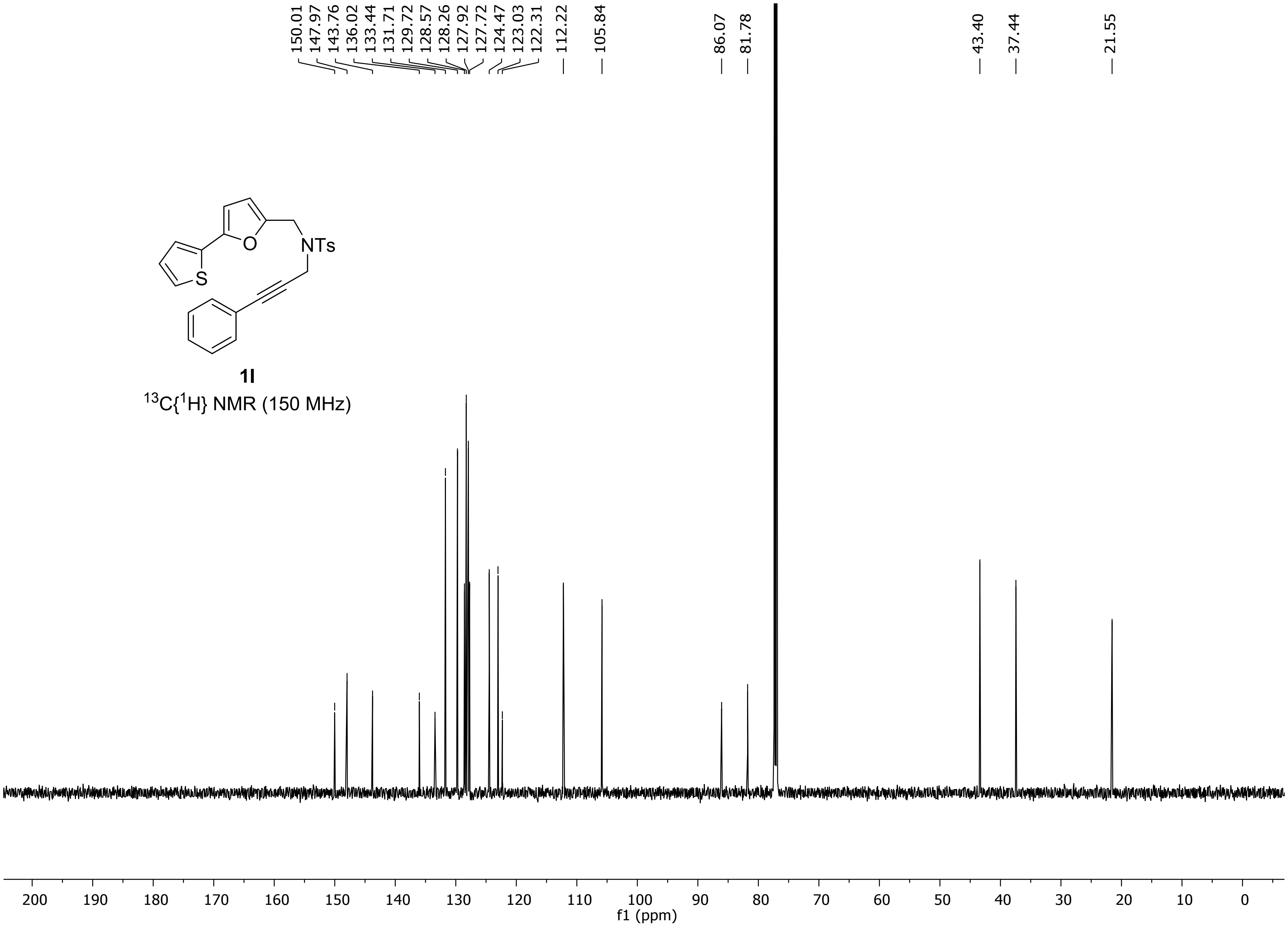
150.01
147.97
143.76
136.02
133.44
131.71
129.72
128.57
128.26
127.92
127.72
124.47
123.03
122.31
112.22
105.84
86.07
81.78
43.40
37.44
21.55
NTs
O
S
1l
$^{13}C\{^1H\}$ NMR (150 MHz)
f1 (ppm)

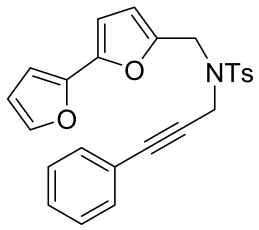

1m

^{1}H NMR (600 MHz)

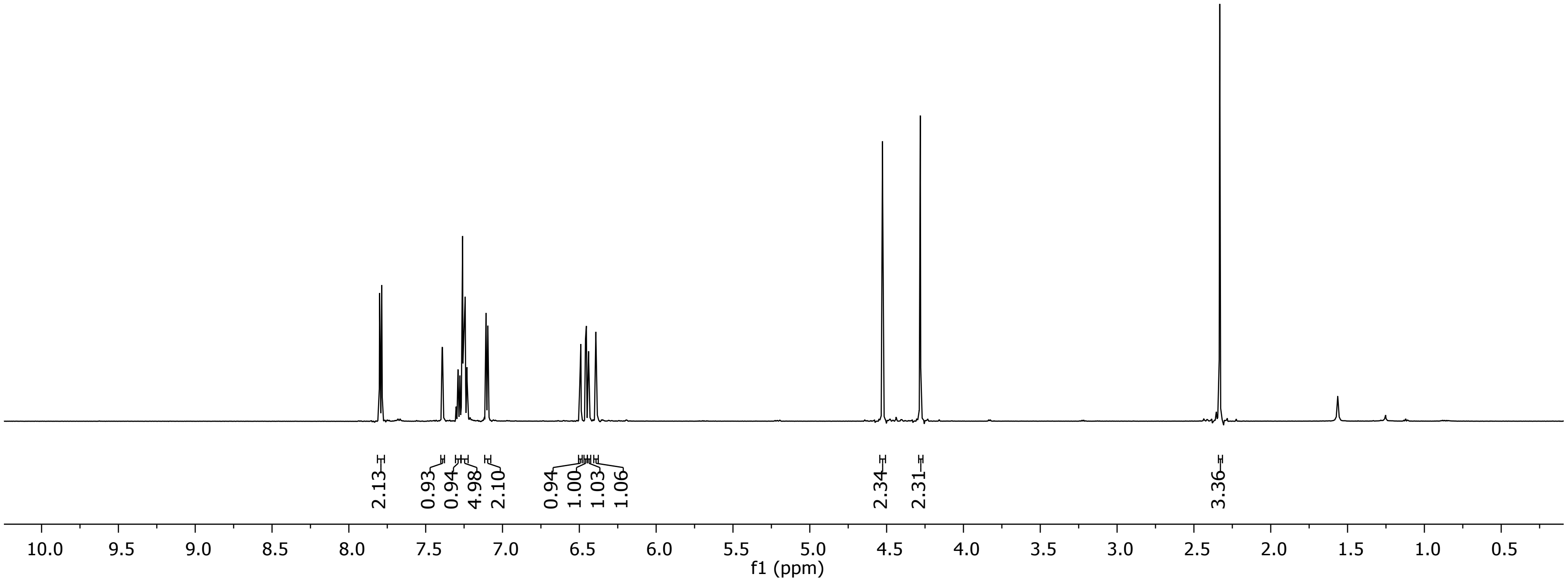

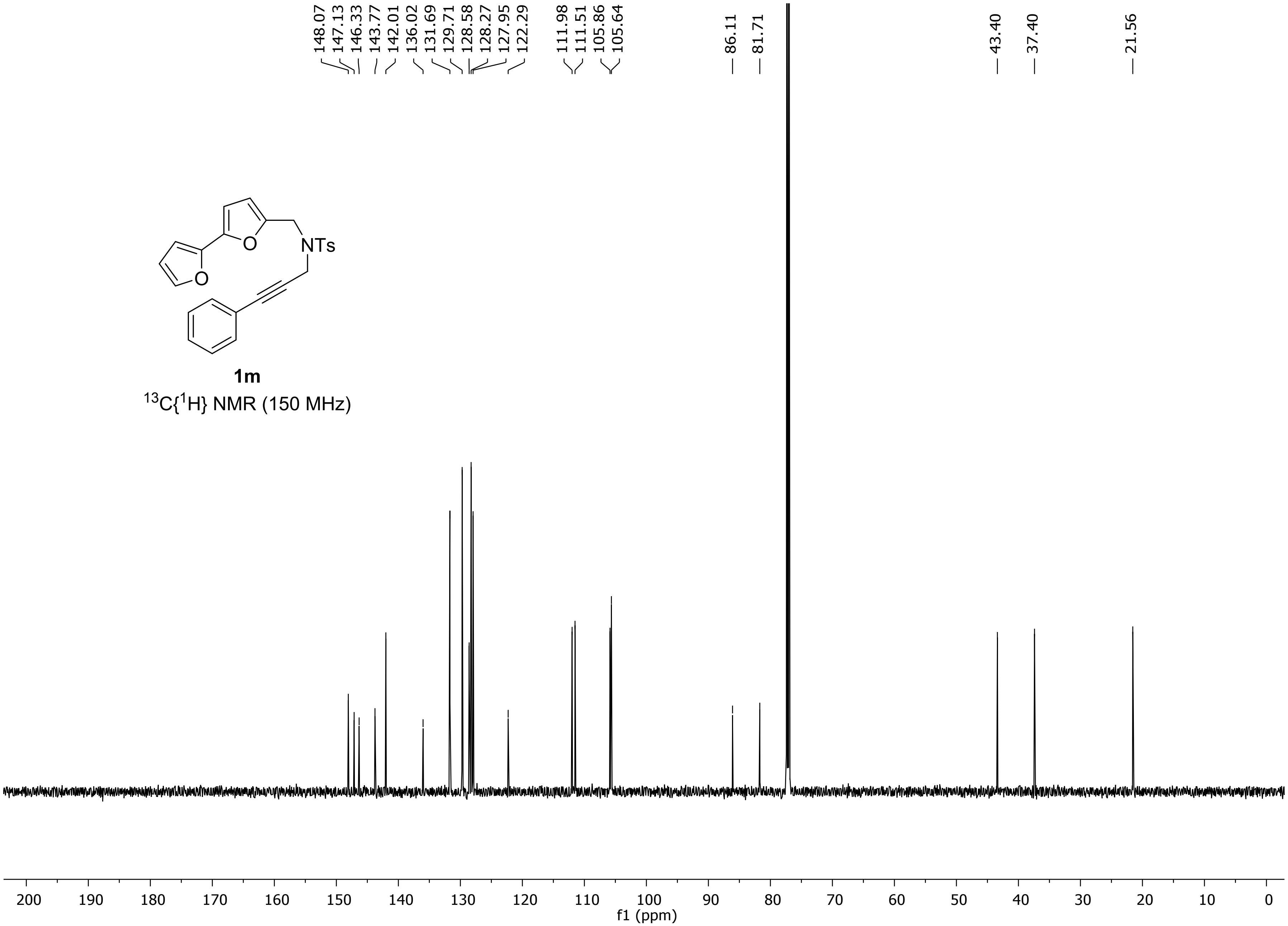
148.07
147.13
146.33
143.77
142.01
136.02
131.69
129.71
128.58
128.27
127.95
122.29
111.98
111.51
105.86
105.64
86.11
81.71
43.40
37.40
21.56
NTs
O
O
1m
$^{13}C\{^{1}H\}$ NMR (150 MHz)
200 190 180 170 160 150 140 130 120 110 100 90 80 70 60 50 40 30 20 10 0
f1 (ppm)

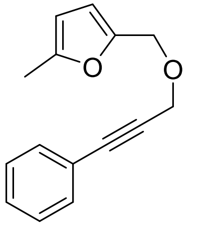

1n

^{1}H NMR (600 MHz)

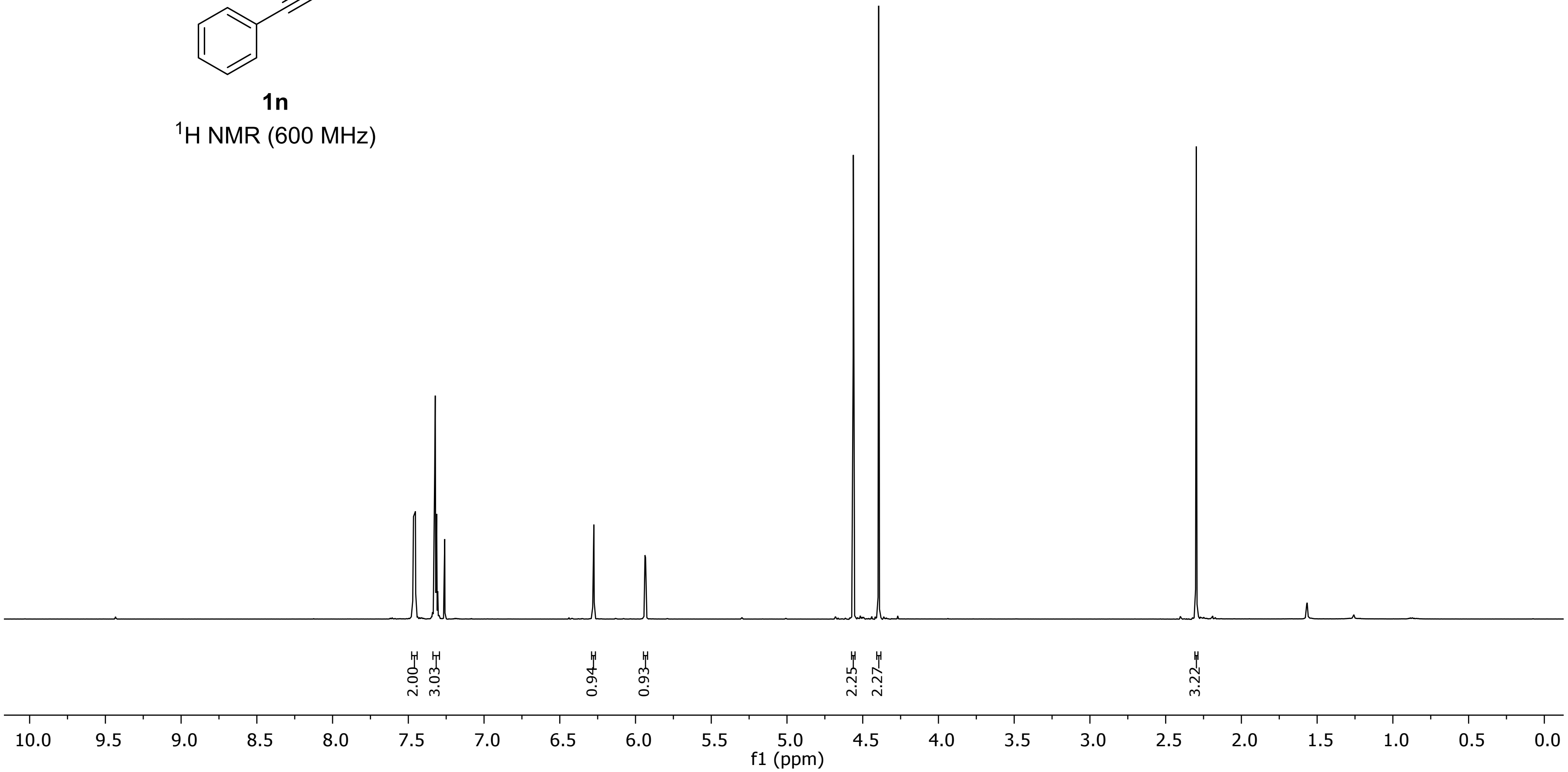

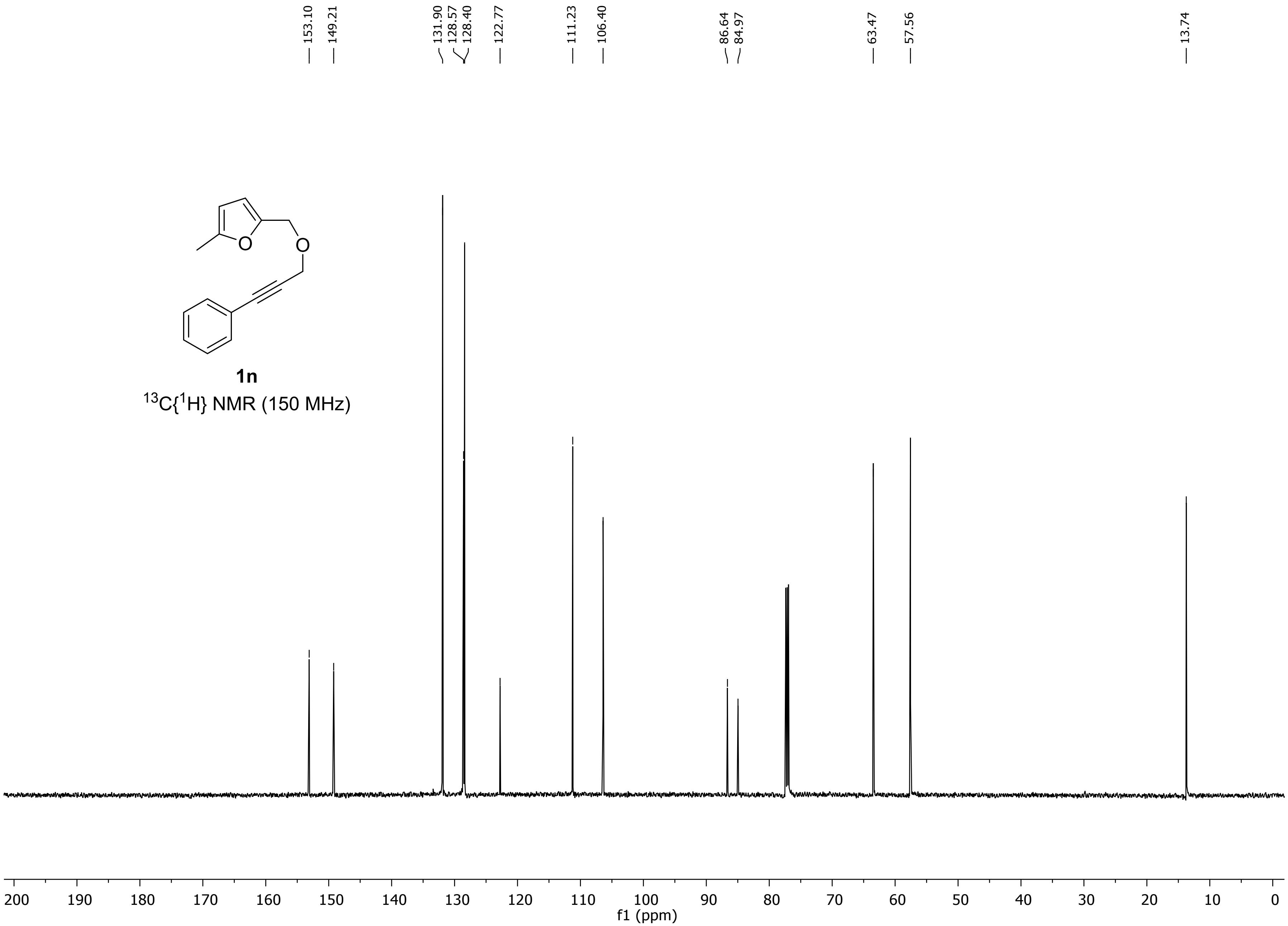

1n

$^{13}C\{^{1}H\}$ NMR (150 MHz)

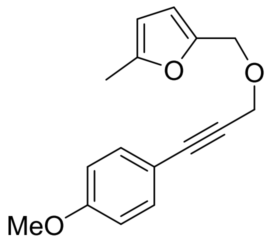

1o

^{1}H NMR (600 MHz)

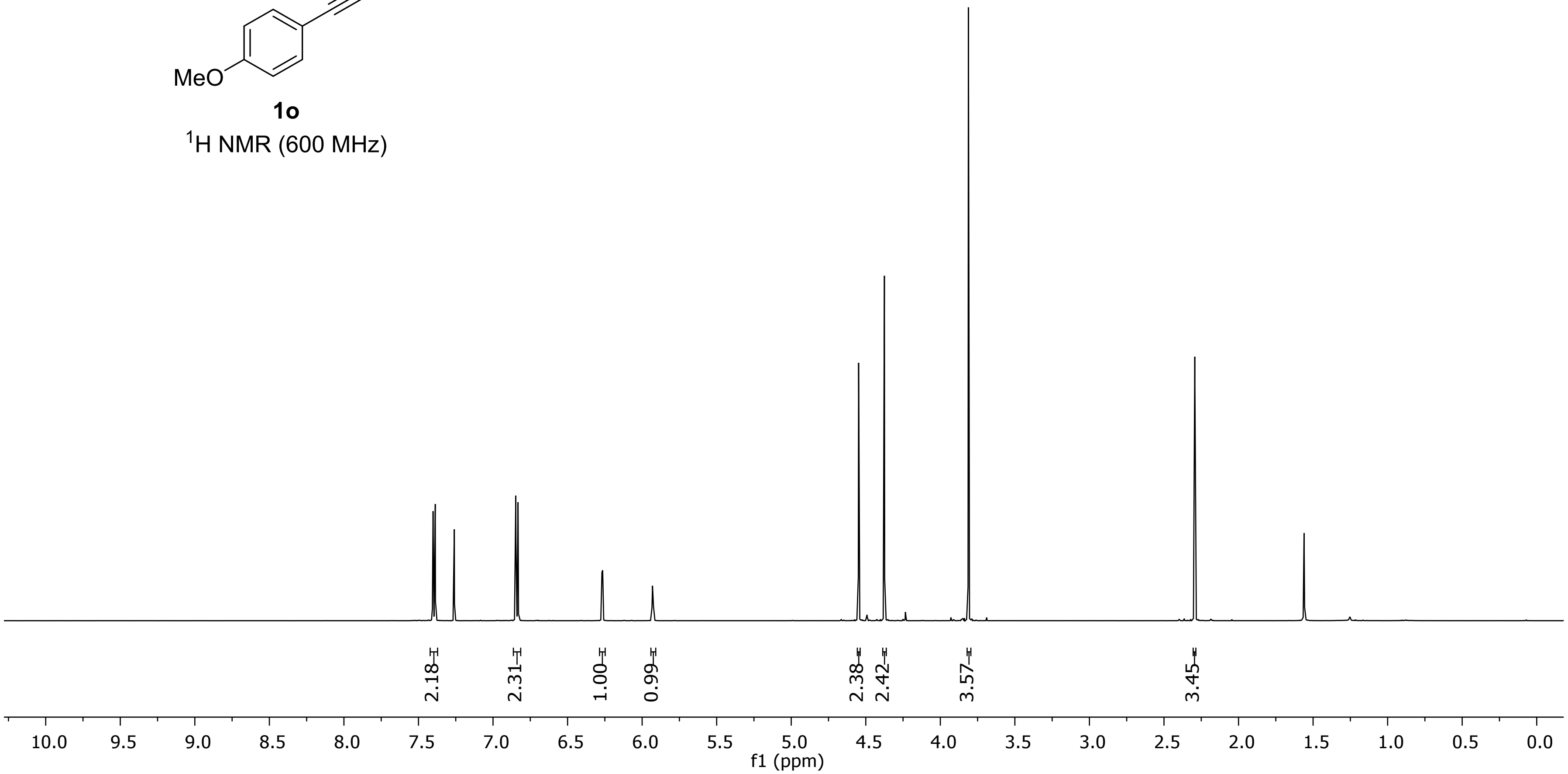

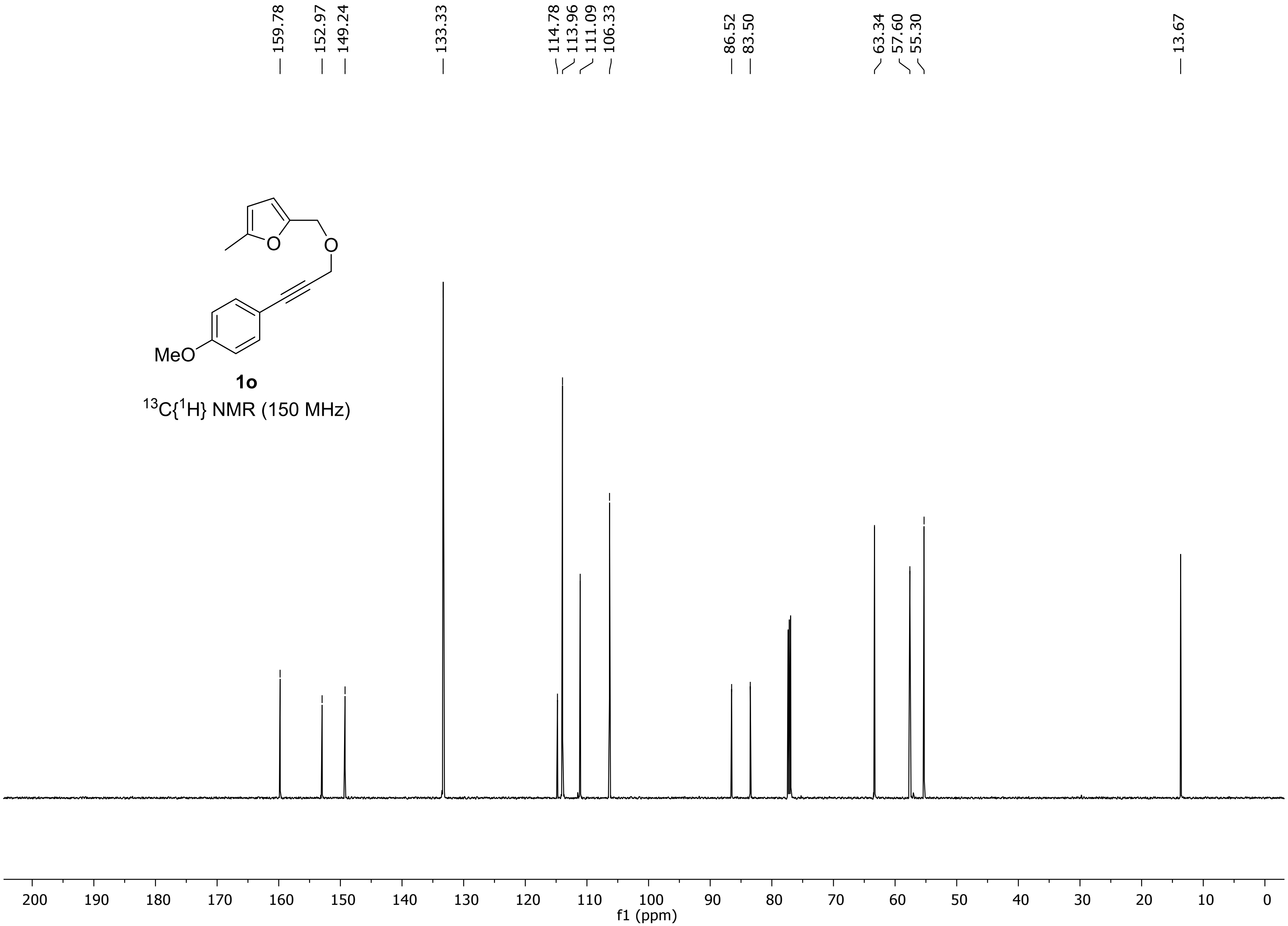

1o

$^{13}C\{^{1}H\}$ NMR (150 MHz)

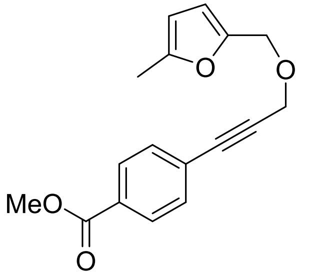

1p

^{1}H NMR (600 MHz)

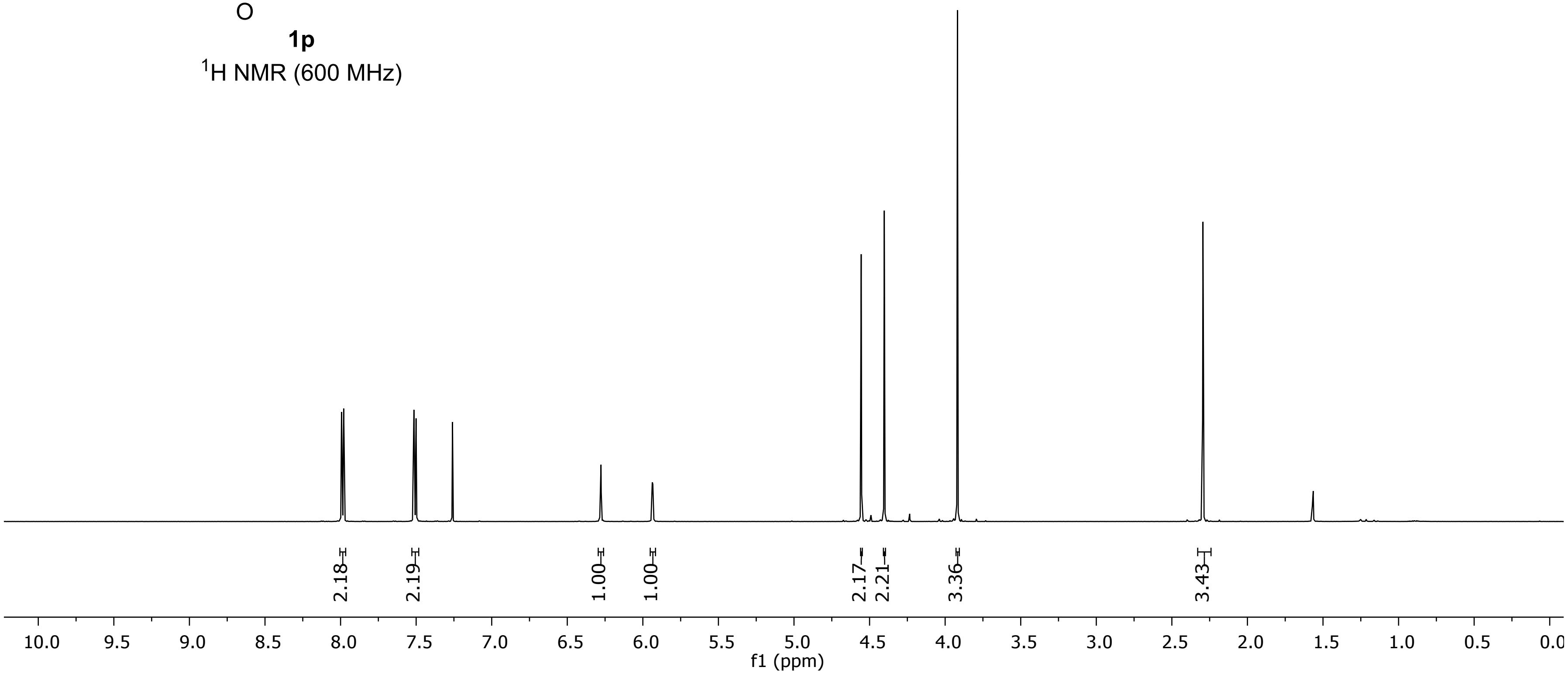

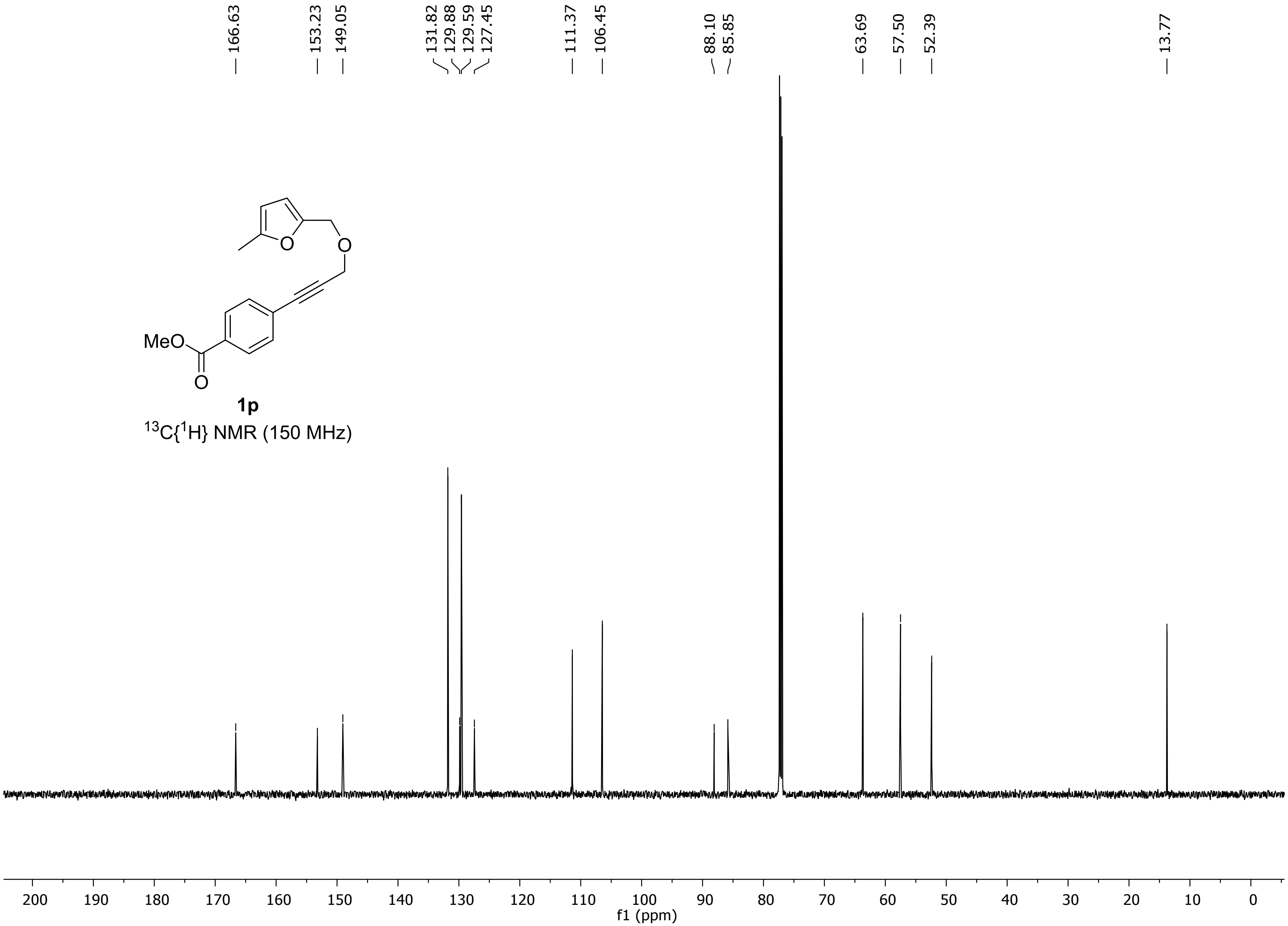
166.63
153.23
149.05
131.82
129.88
129.59
127.45
111.37
106.45
88.10
85.85
63.69
57.50
52.39
13.77
MeO
O
1p
$^{13}C\{^{1}H\}$ NMR (150 MHz)
200 190 180 170 160 150 140 130 120 110 100 90 80 70 60 50 40 30 20 10 0
f1 (ppm)

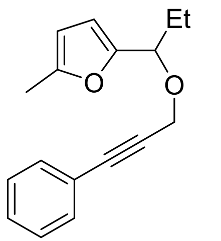

1q

^{1}H NMR (600 MHz)

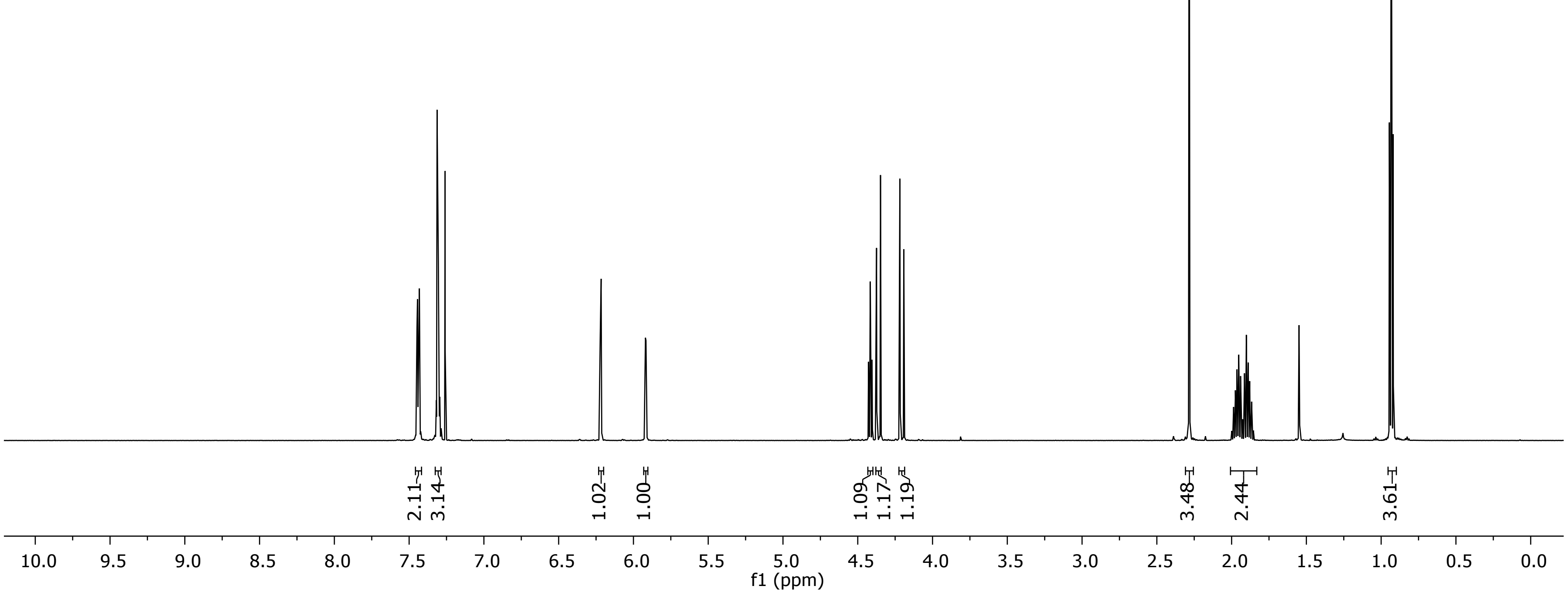

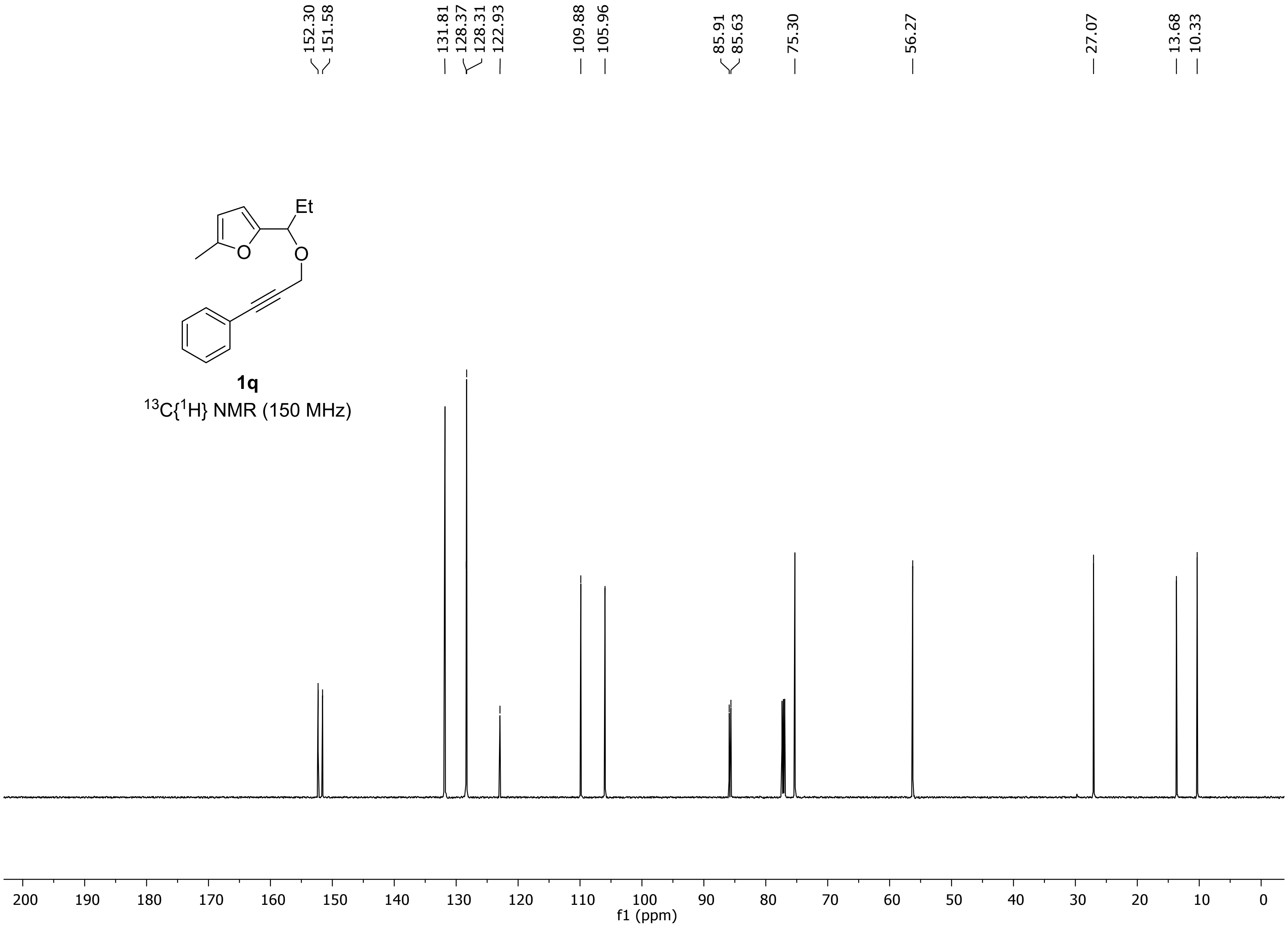
152.30
151.58
131.81
128.37
128.31
122.93
109.88
105.96
85.91
85.63
75.30
56.27
27.07
13.68
10.33
Et
O
O
1q
$^{13}C\{^{1}H\}$ NMR (150 MHz)
200 190 180 170 160 150 140 130 120 110 100 90 80 70 60 50 40 30 20 10 0
f1 (ppm)

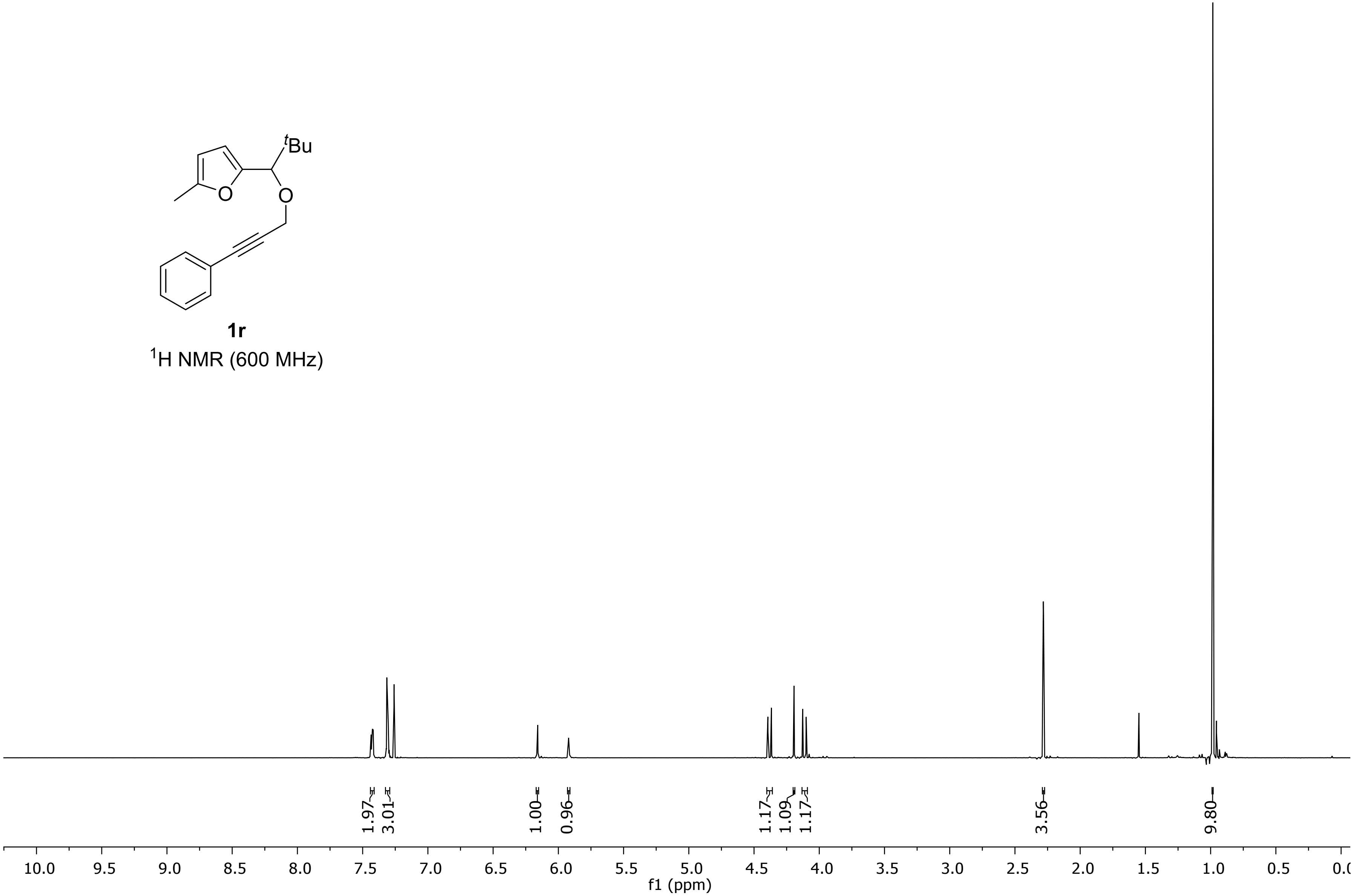

1r

^{1}H NMR (600 MHz)

1r

$^{13}C\{^1H\}$ NMR (150 MHz)

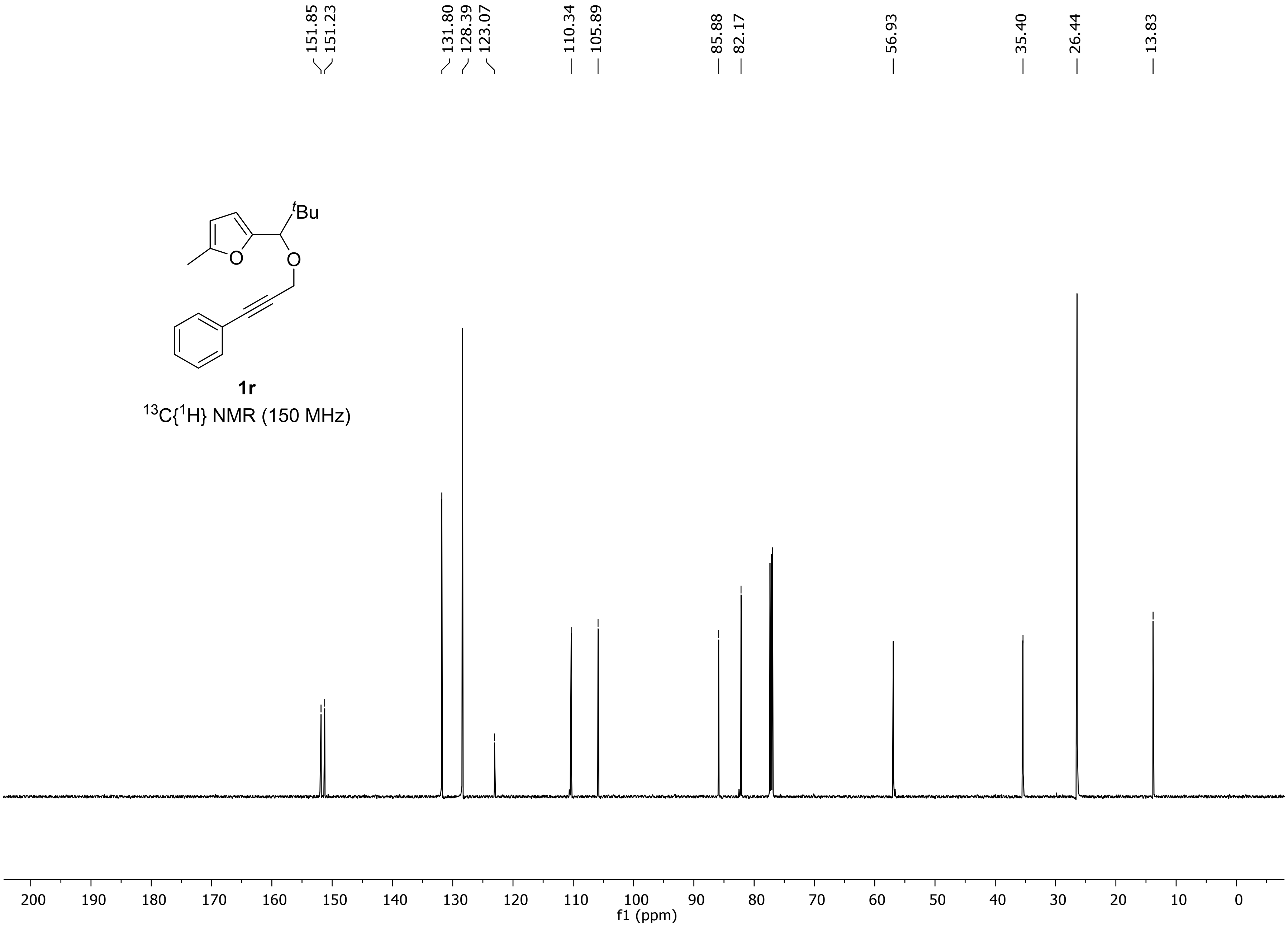

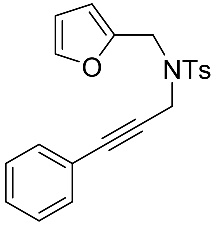

1s

^{1}H NMR (600 MHz)

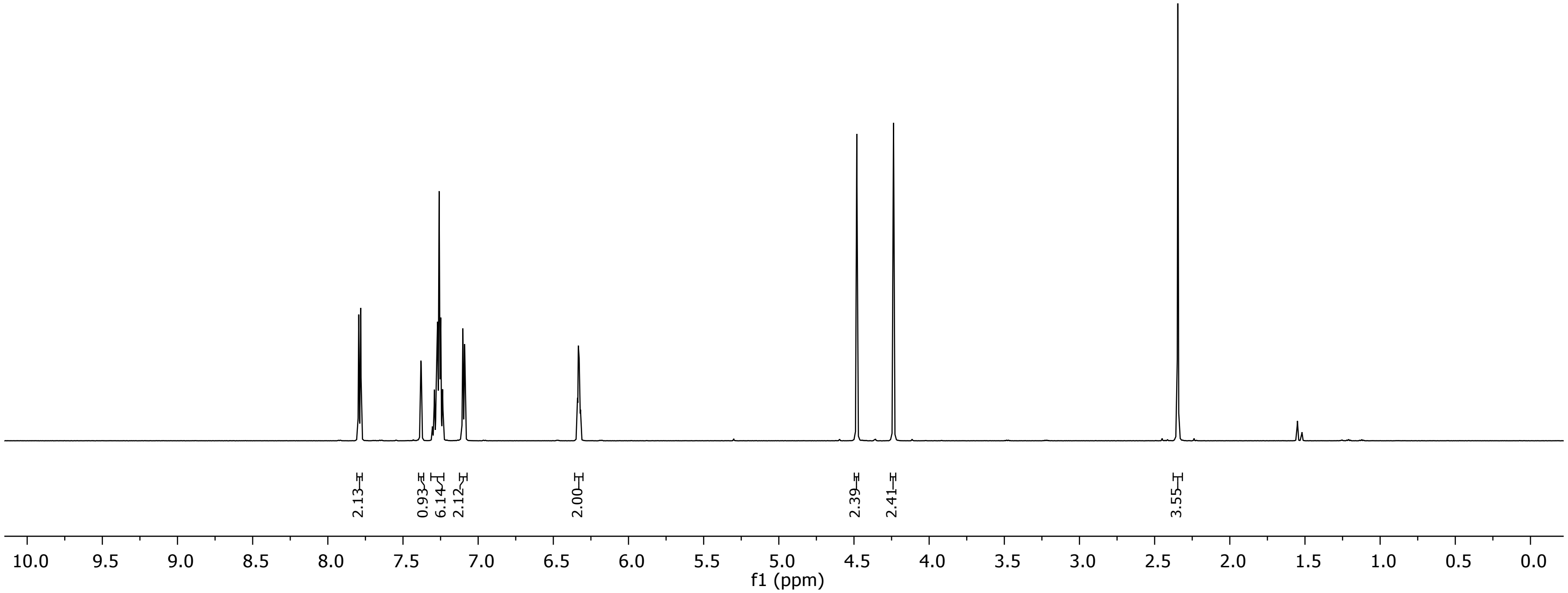

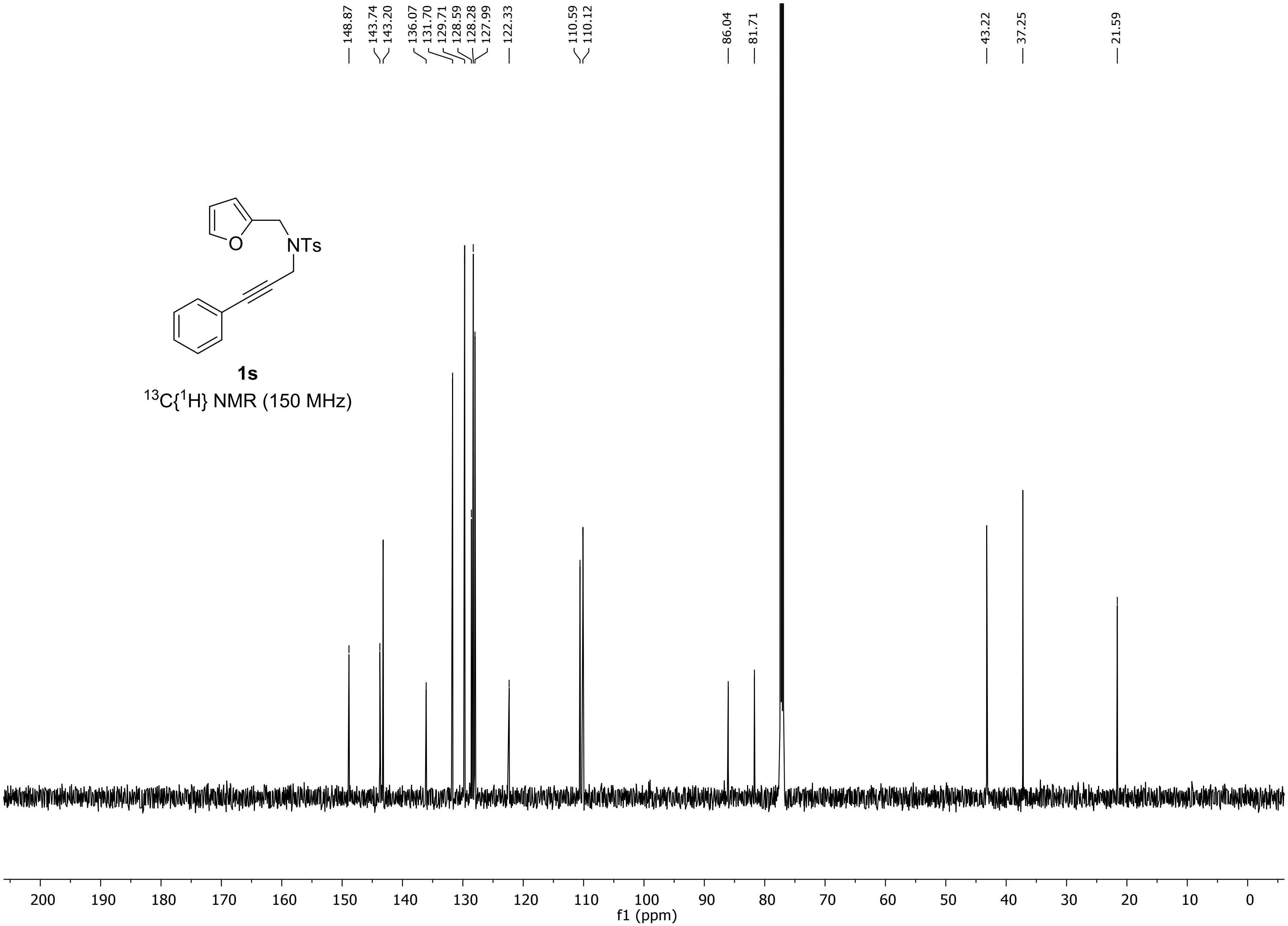

1s

$^{13}C\{^{1}H\}$ NMR (150 MHz)

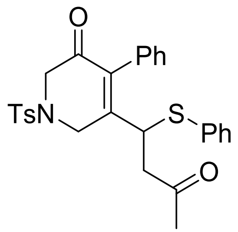

5-D1

^{1}H NMR (600 MHz)

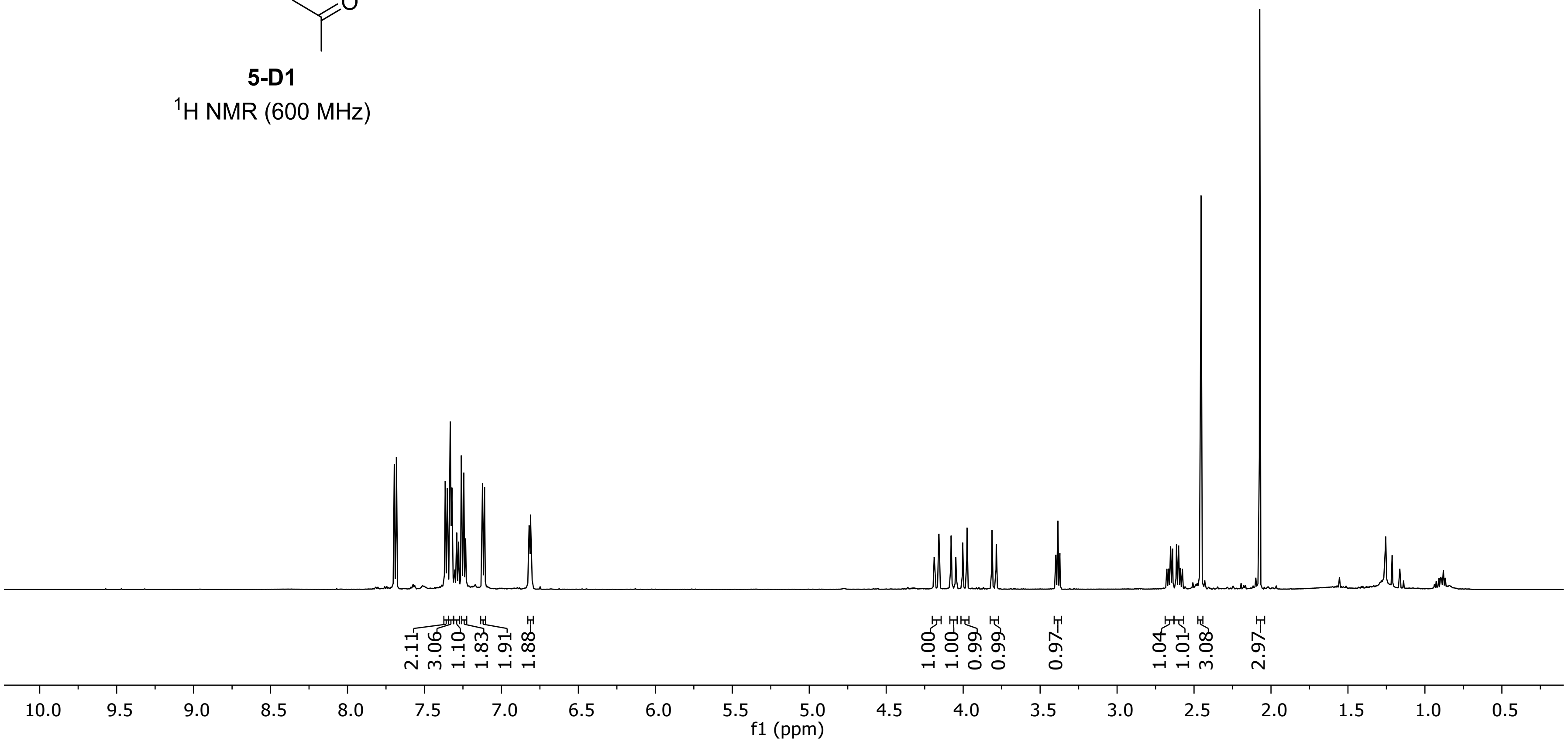

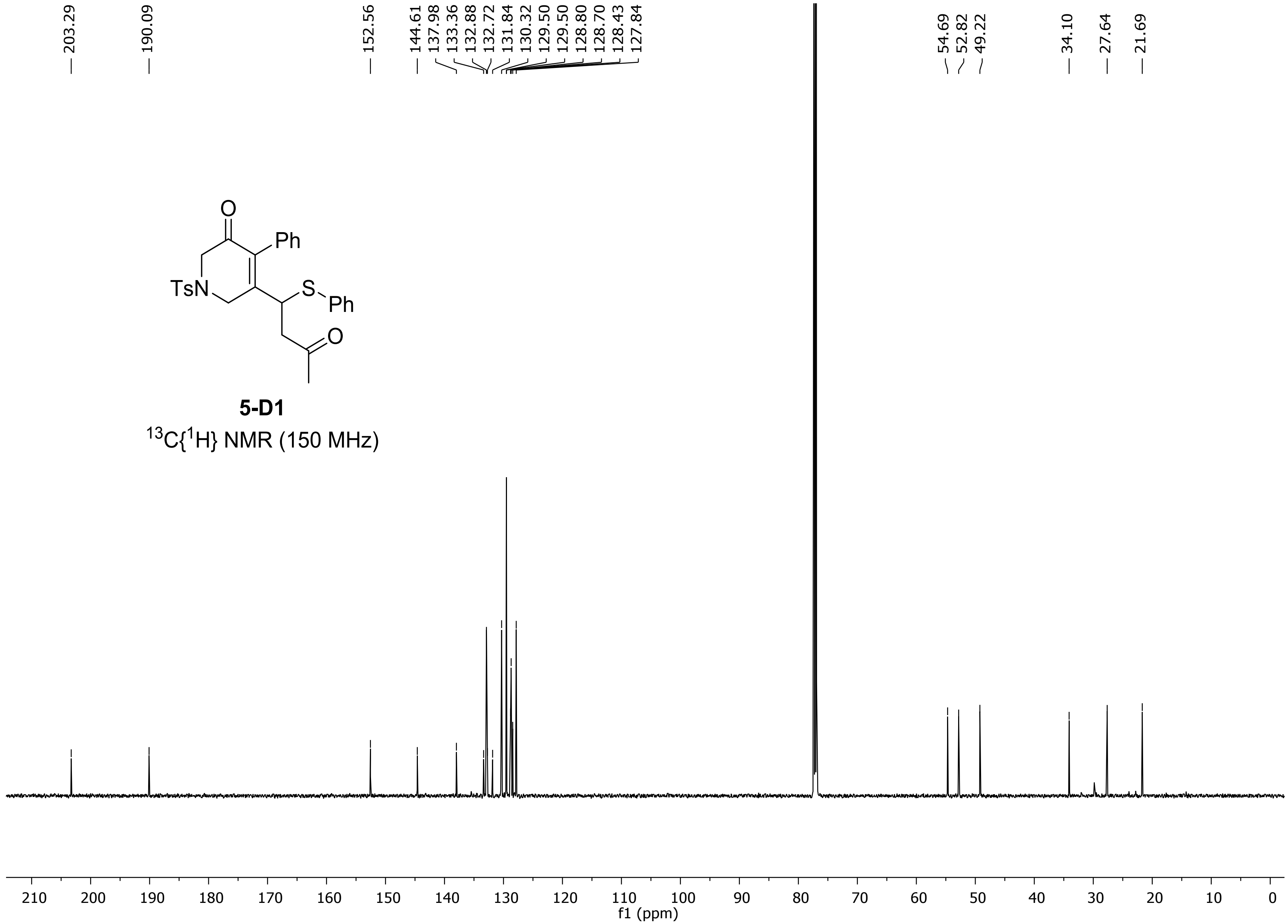
203.29
190.09
152.56
144.61
137.98
133.36
132.88
132.72
131.84
130.32
129.50
129.50
128.80
128.70
128.43
127.84
54.69
52.82
49.22
34.10
27.64
21.69
O
Ph
TsN
S
Ph
O
5-D1
13C{1H} NMR (150 MHz)
210 200 190 180 170 160 150 140 130 120 110 100 90 80 70 60 50 40 30 20 10 0
f1 (ppm)

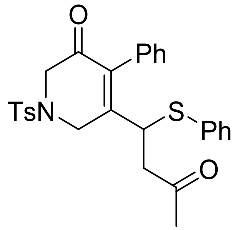

5-D2
^{1}H NMR (600 MHz)

coelution with unreacted starting material **2a**

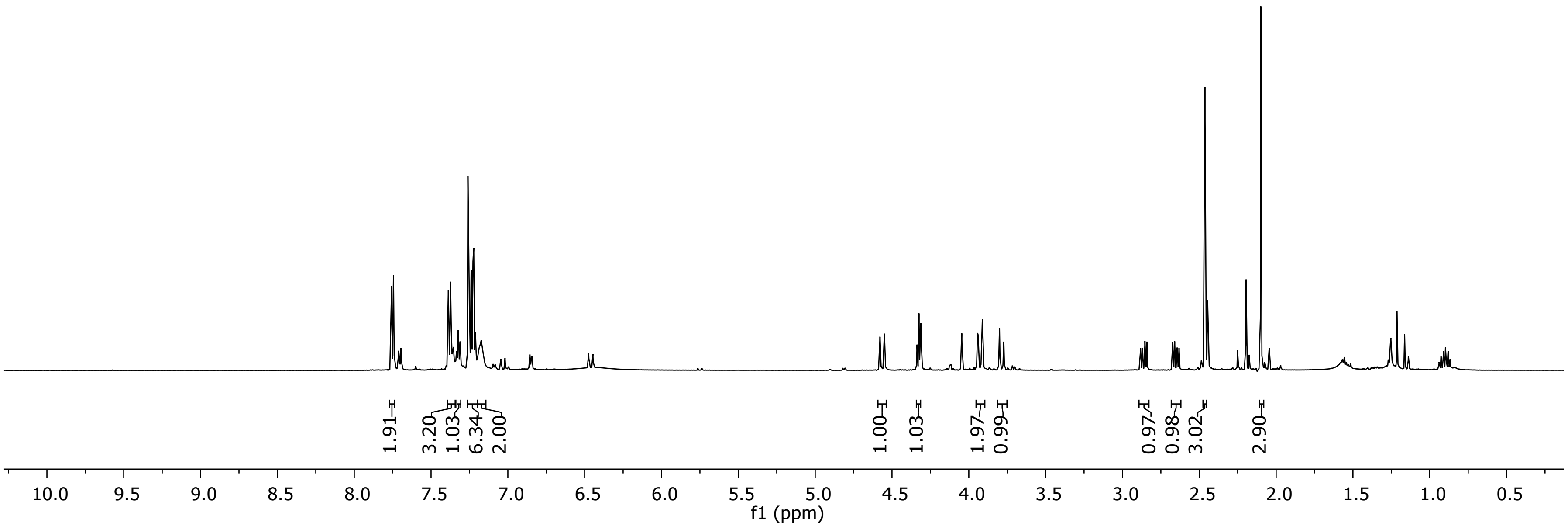

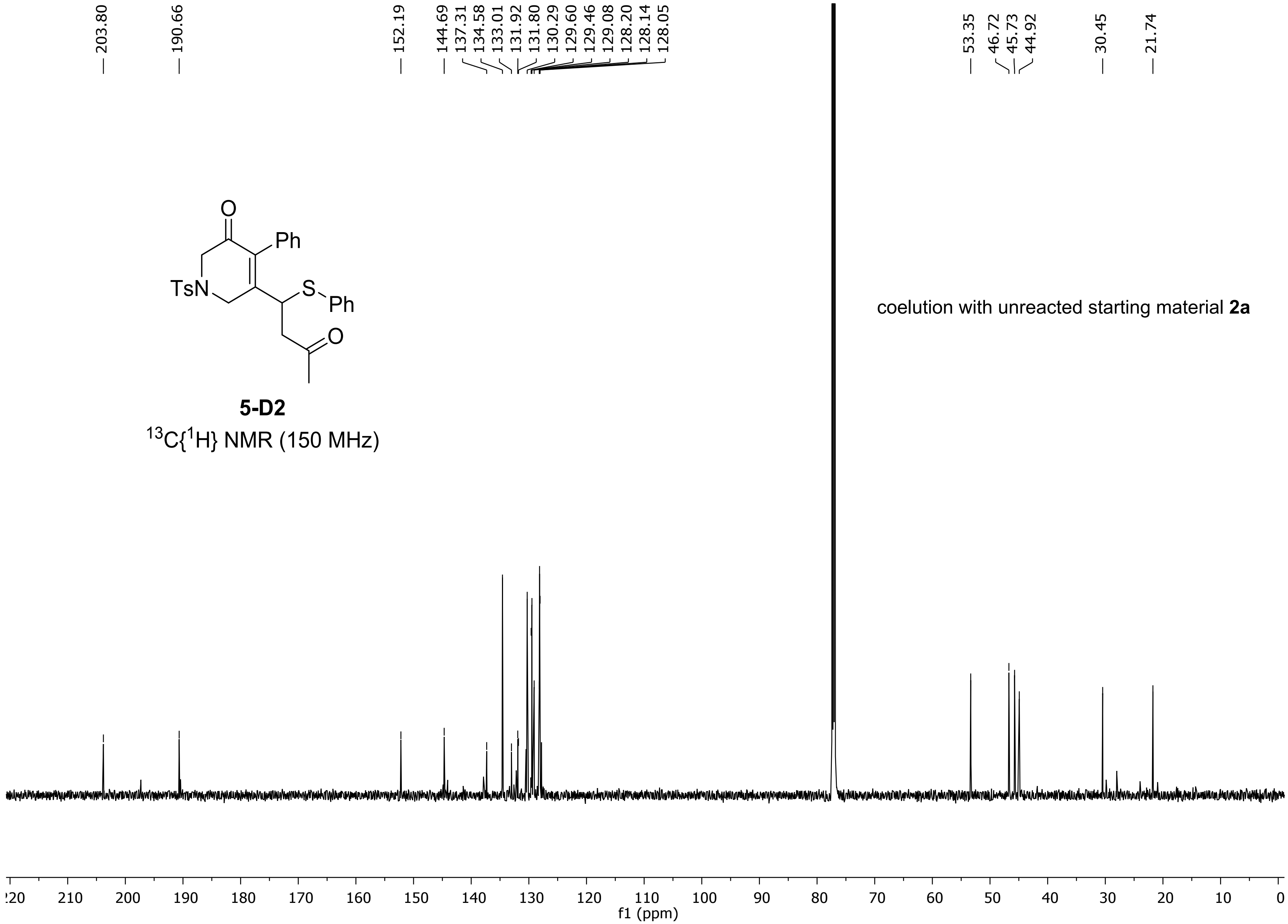
203.80
190.66
152.19
144.69
137.31
134.58
133.01
131.92
131.80
130.29
129.60
129.46
129.08
128.20
128.14
128.05
53.35
46.72
45.73
44.92
30.45
21.74
TsN
Ph
S
Ph
O
O
5-D2
$^{13}C\{^1H\}$ NMR (150 MHz)
coelution with unreacted starting material **2a**
220 210 200 190 180 170 160 150 140 130 120 110 100 90 80 70 60 50 40 30 20 10 0
f1 (ppm)

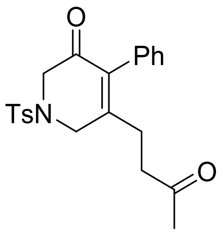

6

^{1}H NMR (600 MHz)

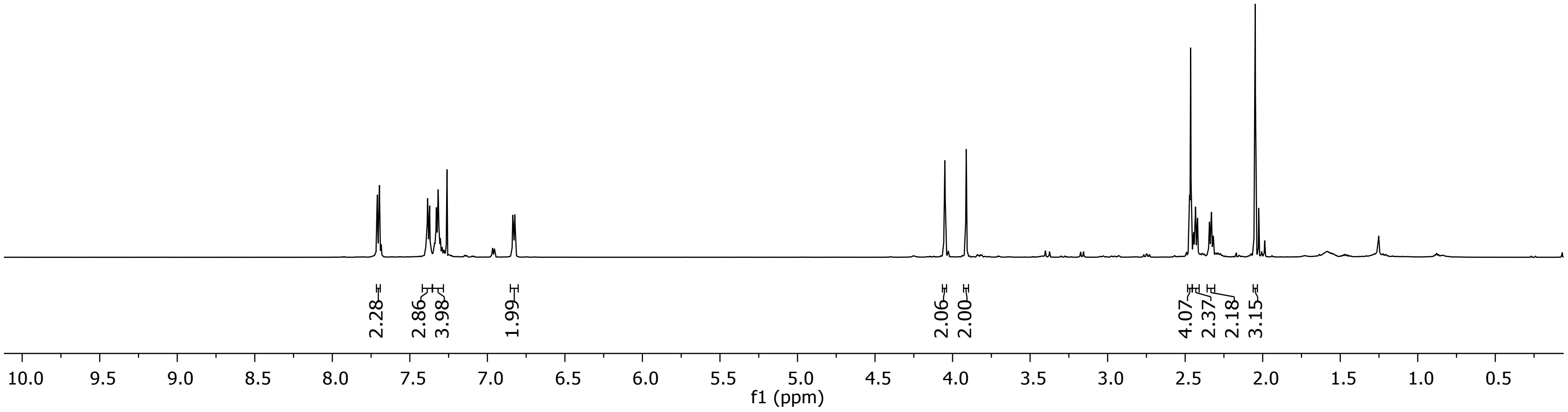

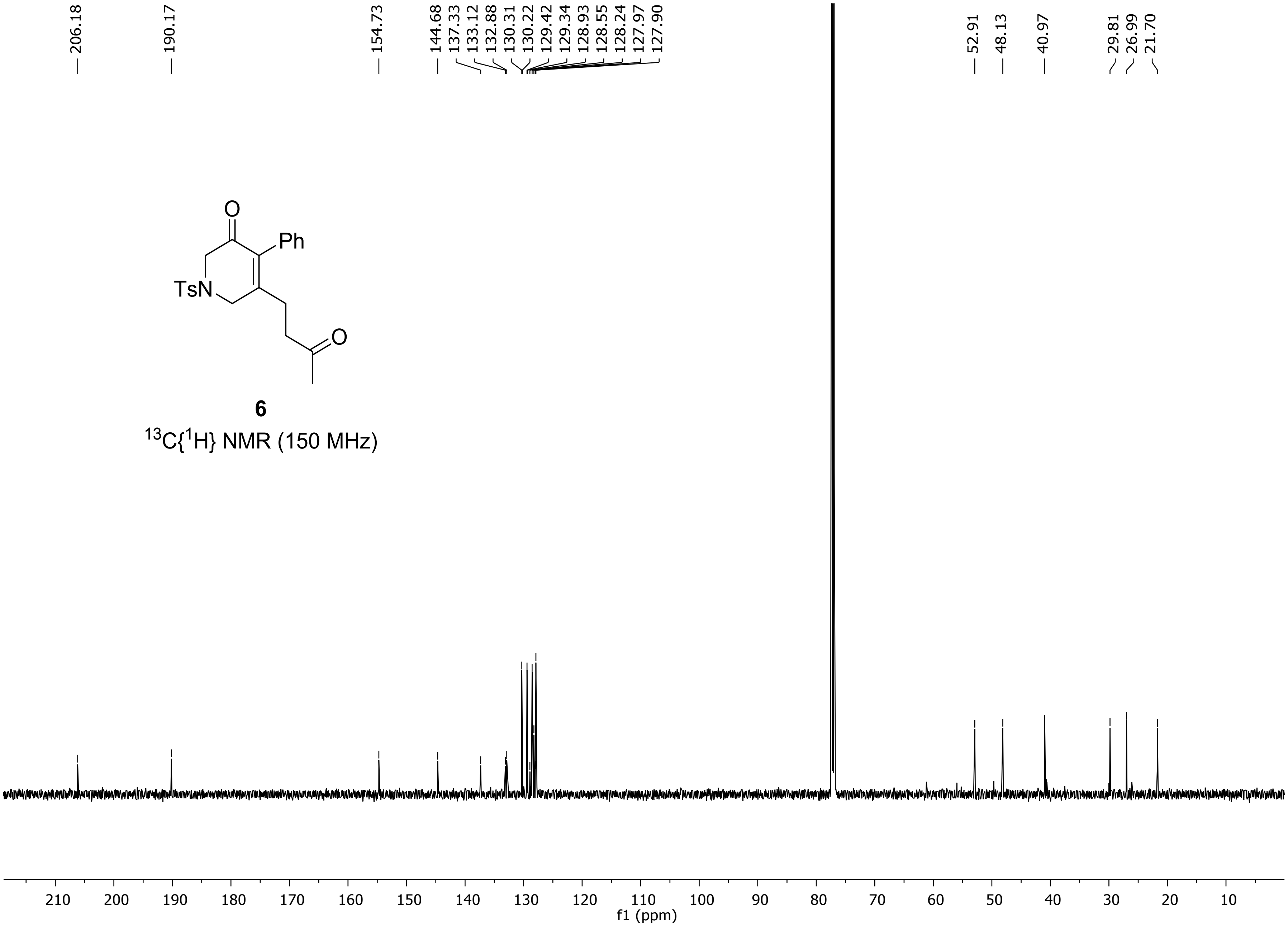
206.18
190.17
154.73
144.68
137.33
133.12
132.88
130.31
130.22
129.42
129.34
128.93
128.55
128.24
127.97
127.90
52.91
48.13
40.97
29.81
26.99
21.70
O
Ph
TsN
O
6
$^{13}C\{^{1}H\}$ NMR (150 MHz)
210
200
190
180
170
160
150
140
130
120
110
100
90
80
70
60
50
40
30
20
10
f1 (ppm)

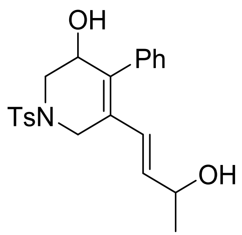

7

^{1}H NMR (600 MHz)

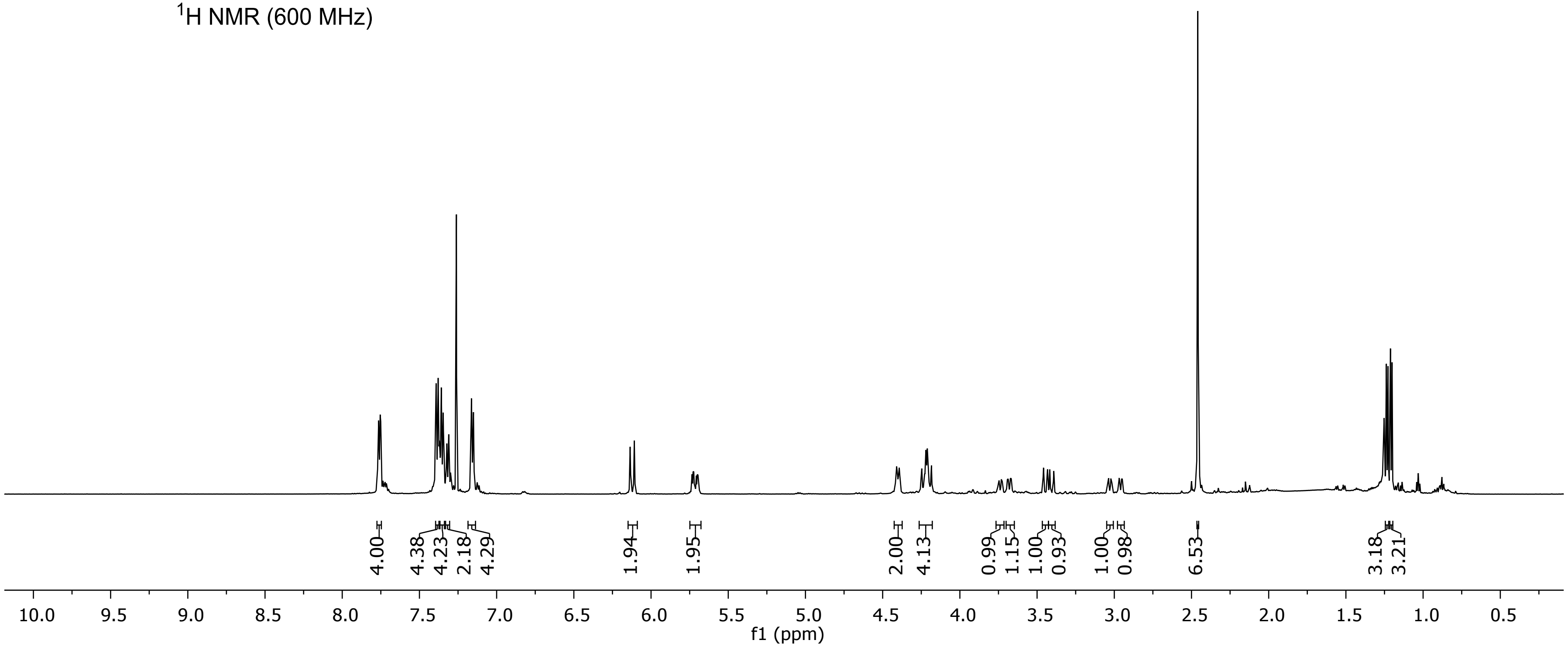

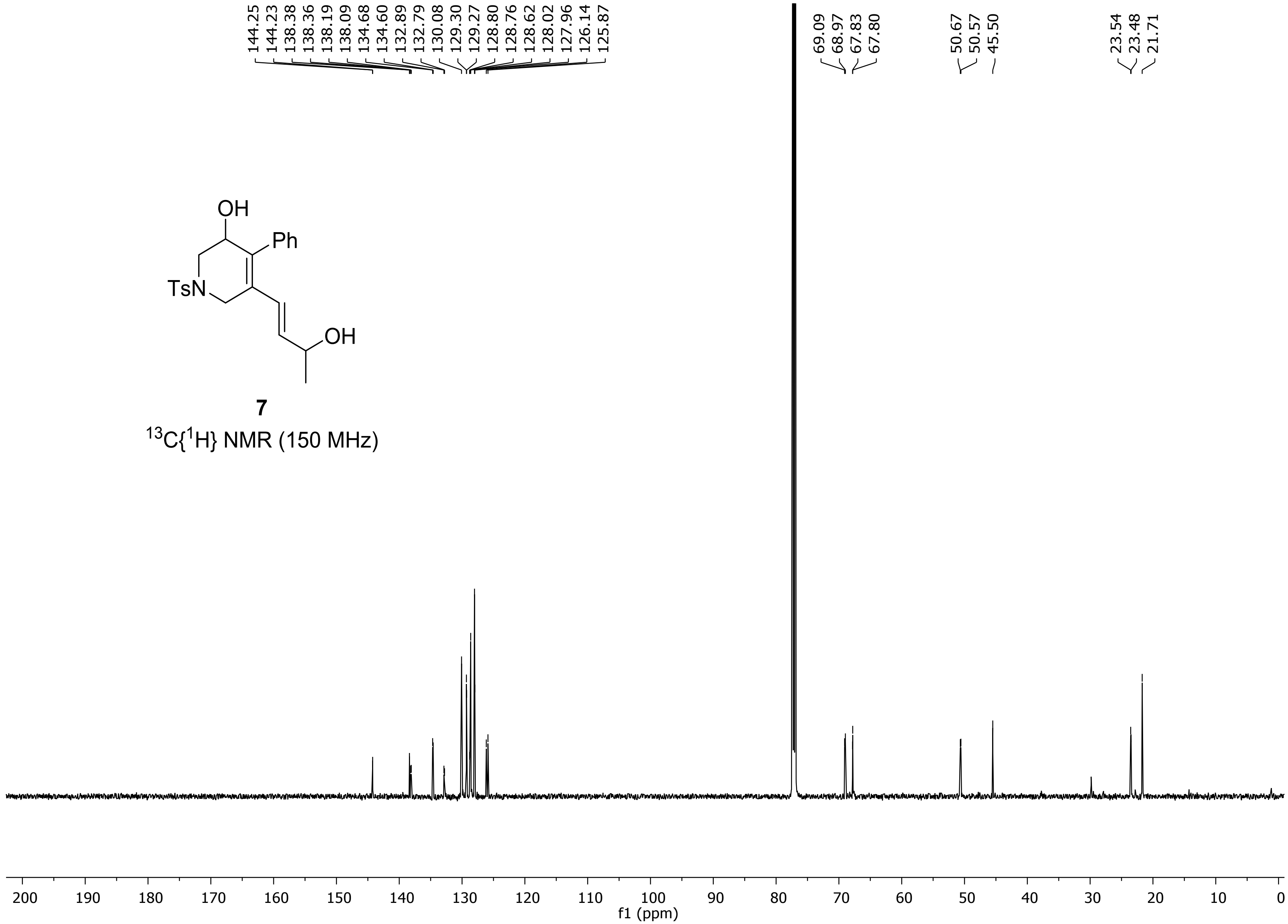
144.25
144.23
138.38
138.36
138.19
138.09
134.68
134.60
132.89
132.79
130.08
129.30
129.27
128.80
128.76
128.62
128.02
127.96
126.14
125.87
69.09
68.97
67.83
67.80
50.67
50.57
45.50
23.54
23.48
21.71
OH
Ph
TsN
OH
7
$^{13}C\{^{1}H\}$ NMR (150 MHz)
200 190 180 170 160 150 140 130 120 110 100 90 80 70 60 50 40 30 20 10 0
f1 (ppm)

Computational details

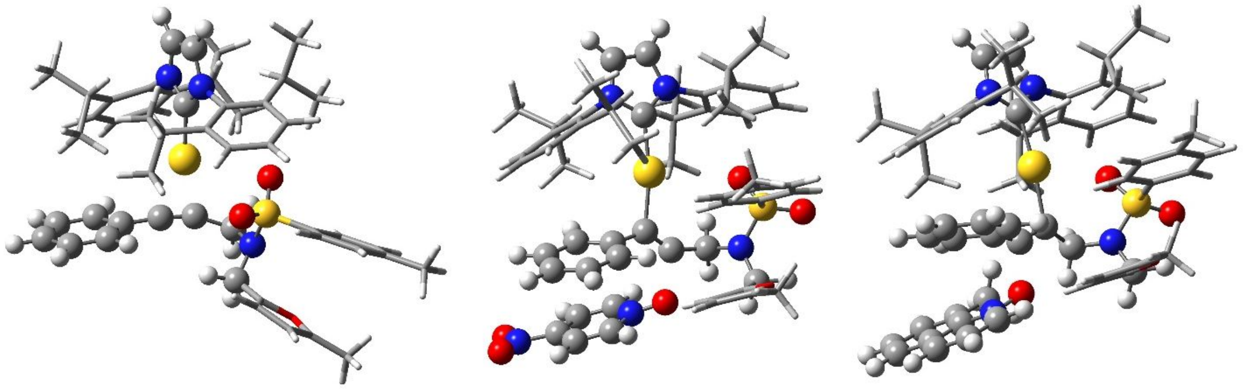

Reactant TS **F** (addition to C1) TS **B** (addition to C2)

Figure S4. Optimized geometries of reactant and TS's for B and F additions. Tube: low level layer (M06/STO-3G); ball-and-stick: high level layer (M06/def2-SVP). See Method section for more details. Figures created with GaussView, Version 6, Dennington, Roy; Keith, Todd A.; Millam, John M. Semichem Inc., Shawnee Mission, KS, 2016

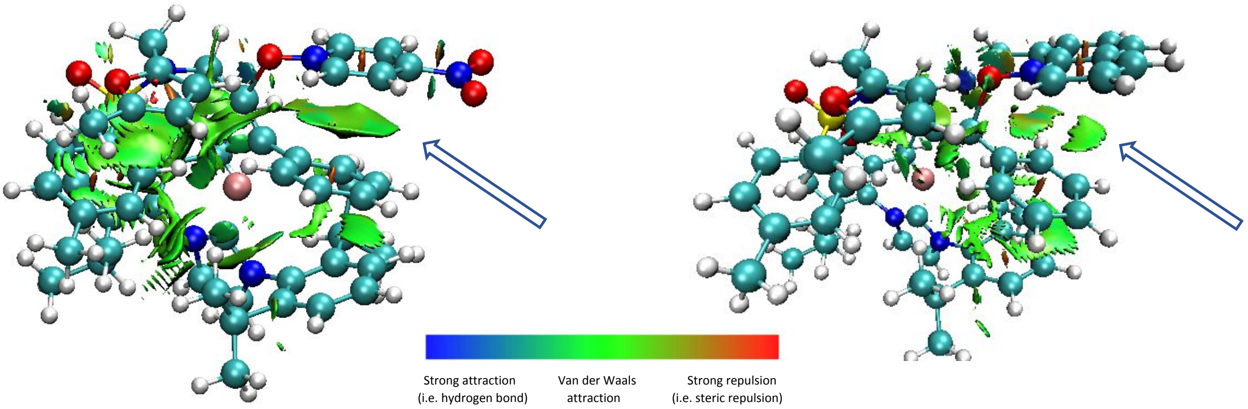

TS **F** (addition to C1) TS **B** (addition to C2)

Figure S5. Noncovalent interaction analysis. The blue arrows highlight a Van der Waals interaction between the aromatic rings of pyridine and phenyl groups. No strong steric repulsion is present. Figures created with VMD software: Humphrey, W., Dalke, A. and Schulten, K., "VMD - Visual Molecular Dynamics", *J. Molec. Graphics*, 1996, vol. 14, pp. 33-38

Geometries. Optimized geometries and energies

Reactant

Atom	X	Y	Z (Angstrom)
6	-2.137325	-2.583590	2.902348
6	-1.854929	-3.382655	1.781629
6	-0.582681	-3.354749	1.174999
6	0.395672	-2.505585	1.758660
6	0.142239	-1.686495	2.889840
6	-1.154252	-1.743389	3.447109
7	1.716031	-2.457046	1.139417
6	2.108460	-1.463116	0.321329
7	3.340053	-1.795103	-0.118131
6	3.735752	-3.005059	0.427735
6	2.712235	-3.422334	1.216947
79	1.228643	0.275910	-0.293505
6	0.089427	1.908572	-1.349434
6	-1.121906	1.571458	-2.132704

7	-2.311570	1.482830	-1.303580
16	-2.410727	0.098183	-0.343172
6	-4.171901	-0.378104	-0.515547
6	-4.906579	-0.603346	0.655392
6	-6.244301	-1.013855	0.544892
6	-6.840955	-1.198292	-0.725001
6	-6.071160	-0.961275	-1.886441
6	-4.730372	-0.550613	-1.789027
6	-8.302825	-1.640663	-0.826597
6	4.019011	-0.873878	-1.023874
6	4.781930	0.181612	-0.450907
6	5.238255	1.193588	-1.322523
6	4.934118	1.154529	-2.694495
6	4.185431	0.094519	-3.234218
6	3.706365	-0.948419	-2.410858
6	5.082708	0.242407	1.061260
6	6.614487	0.297783	1.325440
6	2.872324	-2.104408	-3.004022
6	1.475869	-1.595591	-3.462086
6	-0.267527	-4.245993	-0.046568
6	-0.050896	-5.718312	0.413423
6	1.220214	-0.777519	3.517889
6	0.804466	0.714976	3.402793
6	0.907055	2.569518	-0.696207
6	1.784791	3.481195	-0.022780
6	1.471787	3.926557	1.270863
6	2.306768	4.835820	1.907420
6	3.451140	5.303594	1.262814
6	3.765422	4.862758	-0.022015
6	2.938069	3.952381	-0.667683
8	-2.160891	0.430627	1.048210
8	-1.584763	-0.891283	-1.022878
6	-2.741326	2.739261	-0.666984
6	-4.122301	2.670547	-0.056850
6	-4.560242	2.674108	1.251641
6	-5.994741	2.638713	1.204504
6	-6.351775	2.618826	-0.130006
8	-5.210893	2.645528	-0.944178
6	-7.694174	2.581458	-0.837350
6	1.474671	-1.159538	5.004649
6	-1.389324	-4.179626	-1.118690
6	3.620065	-2.778576	-4.189783
6	4.372714	1.465811	1.703966
1	0.566916	3.556377	1.762088
1	3.169964	3.597369	-1.675458
1	2.061365	5.187613	2.912325
1	4.660480	5.236066	-0.525439
1	4.103263	6.021669	1.766144
1	-0.989658	0.631394	-2.684548
1	-1.286611	2.368700	-2.877427
1	-2.035227	3.046983	0.132009
1	-2.716094	3.505599	-1.464547
1	-3.924607	2.696820	2.146009
1	-6.684101	2.630661	2.056804
1	-7.753818	1.723443	-1.532843
1	-8.494492	2.485039	-0.084120
1	-7.867711	3.506854	-1.418693
1	-4.428720	-0.445203	1.638690
1	-4.131433	-0.360801	-2.696880
1	-6.841489	-1.192552	1.454455
1	-6.530291	-1.100180	-2.879204
1	-8.608734	-1.743656	-1.881116
1	-8.449144	-2.611746	-0.318425

1	-8.964122	-0.902538	-0.335642
1	4.697637	-3.459012	0.210923
1	2.600385	-4.316344	1.822344
1	5.837789	2.023836	-0.916266
1	3.970646	0.069531	-4.314491
1	5.301332	1.955154	-3.358322
1	-2.633293	-4.042731	1.368371
1	-1.387397	-1.126391	4.329110
1	-3.137872	-2.623931	3.363966
1	2.166366	-0.914574	2.955166
1	2.281500	-0.529364	5.417383
1	0.565182	-0.997314	5.608026
1	1.772297	-2.218419	5.095410
1	-0.131551	0.902677	3.957739
1	1.601069	1.360893	3.813759
1	0.637447	0.982225	2.339797
1	0.667778	-3.873413	-0.513016
1	-1.062308	-4.717892	-2.024860
1	-2.313375	-4.656946	-0.750985
1	-1.603992	-3.128652	-1.378947
1	0.768859	-5.793980	1.148207
1	-0.973567	-6.105697	0.879888
1	0.193428	-6.352862	-0.456169
1	2.718937	-2.864479	-2.210564
1	4.626628	-3.112526	-3.884382
1	3.047201	-3.655257	-4.538065
1	3.722899	-2.075957	-5.034333
1	0.886023	-1.246846	-2.590731
1	1.585853	-0.759786	-4.176022
1	0.919859	-2.415177	-3.952351
1	4.679287	-0.676454	1.533602
1	3.280554	1.414724	1.525072
1	4.555174	1.477175	2.793177
1	4.755881	2.404896	1.269066
1	7.126459	-0.566275	0.867958
1	7.046612	1.223712	0.908825
1	6.804390	0.285929	2.412651

Energy -2804.569004 (Hartree)
Thermal correction to Enthalpy 1.066805 (Hartree)
Thermal correction to Gibbs Free Energy 0.915999 (Hartree)

Ox F

Atom	X	Y	Z (Angstrom)
6	1.404264	-1.185398	0.000025
6	0.029194	-1.205147	0.000151
6	-0.667049	0.000000	0.000229
6	0.029194	1.205147	0.000148
6	1.404264	1.185398	0.000021
7	2.107758	0.000000	-0.000005
1	2.044288	-2.068540	-0.000058
1	-0.515172	-2.151260	0.000192
1	-0.515172	2.151260	0.000187
1	2.044288	2.068540	-0.000064
7	-2.127482	0.000000	0.000338
8	-2.682471	-1.077986	-0.000276
8	-2.682471	1.077986	-0.000267
8	3.350021	0.000000	-0.000210

Energy -527.241772 (Hartree)
Thermal correction to Enthalpy 0.104147 (Hartree)
Thermal correction to Gibbs Free Energy 0.062826 (Hartree)

TS F addition to C1

Atom	X	Y	Z (Angstrom)
6	2.841198	-2.953077	3.161974
6	1.686799	-2.380876	3.720764
6	0.424395	-2.558386	3.114632
6	0.376838	-3.338875	1.927262
6	1.533117	-3.908194	1.325695
6	2.767745	-3.701359	1.974770
7	-0.899255	-3.487156	1.241482
6	-1.322929	-2.566631	0.346674
7	-2.496449	-3.031300	-0.129467
6	-2.806908	-4.248789	0.466697
6	-1.802142	-4.535298	1.330644
79	-0.164260	-0.932464	-0.093459
6	1.108138	1.296488	-1.457036
6	0.171195	1.648006	-2.544326
7	-0.816647	2.658827	-2.241897
16	-2.382781	2.209586	-1.866456
6	-2.606488	2.776541	-0.126969
6	-3.750778	3.533452	0.147147
6	-3.989513	3.965747	1.462556
6	-3.079459	3.653536	2.497606
6	-1.921364	2.902231	2.189009
6	-1.683651	2.457617	0.878148
6	-3.339352	4.120690	3.932292
6	-3.314989	-2.354964	-1.129162
6	-2.966442	-2.490834	-2.496446
6	-3.790966	-1.837432	-3.438892
6	-4.903788	-1.089558	-3.025690
6	-5.223522	-0.974285	-1.661293
6	-4.439294	-1.612100	-0.680157
6	-1.749151	-3.318354	-2.960750
6	-2.162912	-4.396227	-4.003274
6	-4.743075	-1.472779	0.827446
6	-3.989481	-0.232691	1.390293
6	-0.835993	-1.905204	3.717345
6	-1.067516	-2.369472	5.183501
6	1.459595	-4.693186	-0.000694
6	2.158740	-3.889493	-1.133337
6	1.323851	0.579649	-0.408411
6	2.392887	0.603430	0.597731
6	2.443127	1.638725	1.543096
6	3.486325	1.697911	2.463626
6	4.499409	0.739703	2.445444
6	4.439365	-0.306704	1.525135
6	3.379030	-0.393477	0.626761
8	-2.389112	0.754177	-1.916742
8	-3.309992	2.995405	-2.655063
6	-0.487143	4.081029	-2.286256
6	-0.088904	4.685879	-0.944507
6	1.008760	4.508303	-0.128605
6	0.781529	5.297007	1.050526
6	-0.442559	5.915965	0.886951
8	-1.012425	5.555587	-0.343297
6	-1.224191	6.898509	1.740305
6	2.090956	-6.107208	0.138644
6	-0.717250	-0.357215	3.644461
6	-6.262169	-1.365371	1.130947
6	-0.656470	-2.383436	-3.549659
1	1.658065	2.401449	1.542067
1	3.311752	-1.232487	-0.074984
1	3.512991	2.507626	3.198447
1	5.220319	-1.073768	1.518604
1	5.324959	0.796311	3.159781

1	-0.363275	0.722433	-2.811981
1	0.741434	1.963599	-3.433796
1	0.337798	4.199678	-3.012998
1	-1.359186	4.631996	-2.682609
1	1.871613	3.881076	-0.384250
1	1.445979	5.406517	1.915580
1	-0.807217	6.904703	2.761857
1	-1.160710	7.925274	1.331233
1	-2.291573	6.617054	1.791154
1	-4.437995	3.786038	-0.681170
1	-0.771497	1.877954	0.640562
1	-4.887710	4.562529	1.692732
1	-1.189956	2.678749	2.984069
1	-2.392686	4.393890	4.430869
1	-4.016023	4.992313	3.943201
1	-3.812420	3.312100	4.521985
1	-3.715086	-4.791411	0.220998
1	-1.647933	-5.382087	1.992635
1	-3.556507	-1.924862	-4.511512
1	-6.100677	-0.384455	-1.354639
1	-5.535943	-0.589052	-3.777930
1	1.760574	-1.792784	4.649171
1	3.682743	-4.137326	1.543456
1	3.814764	-2.812836	3.660467
1	0.392117	-4.811339	-0.278158
1	1.971306	-6.663348	-0.807210
1	3.169018	-6.031734	0.361390
1	1.604080	-6.678283	0.947864
1	2.081765	-4.441127	-2.087182
1	1.675626	-2.898691	-1.254046
1	3.226886	-3.740506	-0.895126
1	-1.712113	-2.210548	3.108942
1	-1.645565	0.107952	4.020951
1	0.133428	-0.004497	4.254223
1	-0.557603	-0.034154	2.595648
1	-1.154150	-3.468246	5.240901
1	-0.232100	-2.048384	5.829018
1	-1.998070	-1.921484	5.572875
1	-4.351008	-2.373340	1.342609
1	-6.667679	-0.405835	0.767637
1	-6.822532	-2.188813	0.655657
1	-6.423232	-1.411763	2.221779
1	-4.146313	-0.154765	2.480689
1	-2.904183	-0.314280	1.186252
1	-4.367792	0.687284	0.912442
1	-1.317090	-3.833862	-2.077857
1	0.202082	-2.982645	-3.902251
1	-1.061169	-1.802452	-4.397479
1	-0.302810	-1.678191	-2.770542
1	-1.288586	-5.017861	-4.263099
1	-2.954615	-5.051272	-3.600708
1	-2.536090	-3.921606	-4.926761
8	2.487891	2.510700	-1.852212
6	4.510899	2.516173	-0.785604
6	4.063349	0.989845	-2.539135
6	5.753908	1.957622	-0.579783
1	4.100325	3.348824	-0.213797
6	5.300109	0.399582	-2.376670
1	3.324797	0.688212	-3.283729
6	6.125925	0.877868	-1.369971
1	6.420111	2.334791	0.197747
1	5.621193	-0.429084	-3.010075
7	3.676540	2.009214	-1.729045

7	7.412898	0.204033	-1.109011
8	7.750120	-0.646972	-1.896425
8	8.010018	0.549424	-0.117287

Energy -3331.804311 (Hartree)
Thermal correction to Enthalpy 1.171384 (Hartree)
Thermal correction to Gibbs Free Energy 1.005183 (Hartree)

TS F addition to C2

Atom	X	Y	Z (Angstrom)
6	2.391264	-3.311815	3.336081
6	1.326678	-2.543045	3.831944
6	0.082702	-2.515137	3.165827
6	-0.041346	-3.292160	1.984717
6	1.030081	-4.050556	1.440968
6	2.250429	-4.046237	2.146590
7	-1.313311	-3.298340	1.278874
6	-1.649470	-2.366668	0.357756
7	-2.849323	-2.754940	-0.126986
6	-3.265685	-3.928850	0.488389
6	-2.299635	-4.271520	1.374688
79	-0.469243	-0.800890	-0.249566
6	0.831162	0.671835	-1.071226
6	0.444314	1.384915	-2.342396
7	-0.403608	2.547852	-2.144976
16	-2.056904	2.356193	-2.029072
6	-2.448115	3.005222	-0.346649
6	-3.521745	3.898862	-0.261432
6	-3.886790	4.420262	0.990776
6	-3.170779	4.058686	2.153931
6	-2.076728	3.170323	2.037002
6	-1.714231	2.635660	0.789322
6	-3.573098	4.617116	3.521722
6	-3.586200	-2.035704	-1.159440
6	-3.241933	-2.252535	-2.518252
6	-3.981071	-1.544107	-3.491280
6	-5.005856	-0.662141	-3.116093
6	-5.325931	-0.470813	-1.760426
6	-4.628949	-1.162605	-0.750340
6	-2.122488	-3.226317	-2.942300
6	-2.662909	-4.314992	-3.913356
6	-4.947924	-0.954642	0.745780
6	-4.149081	0.270157	1.275078
6	-1.078022	-1.654417	3.702744
6	-1.447862	-2.043688	5.162192
6	0.888401	-4.815140	0.108612
6	1.704163	-4.087396	-0.997341
6	1.948979	0.797934	-0.423117
6	2.689396	0.387914	0.761522
6	2.694112	1.201155	1.904653
6	3.487900	0.864792	2.997090
6	4.292112	-0.274424	2.954162
6	4.284301	-1.094527	1.826855
6	3.480114	-0.770451	0.737712
8	-2.285547	0.919032	-2.085699
8	-2.724275	3.255890	-2.950491
6	0.135343	3.902692	-2.127494
6	0.498422	4.469795	-0.757591
6	1.524199	4.219927	0.131786
6	1.299856	5.065815	1.271545
6	0.153822	5.791889	1.012619
8	-0.369986	5.445325	-0.241006
6	-0.582306	6.871650	1.785561
6	1.338186	-6.297400	0.239315

6	-0.701585	-0.149577	3.613098
6	-6.466357	-0.768935	1.015557
6	-0.947863	-2.446984	-3.595508
1	2.067377	2.098926	1.917441
1	3.449218	-1.416730	-0.146656
1	3.482692	1.496469	3.889418
1	4.901882	-1.996471	1.799848
1	4.920991	-0.530821	3.810978
1	-0.068061	0.681327	-3.015249
1	1.346634	1.745753	-2.866314
1	1.026706	3.914148	-2.781832
1	-0.610697	4.574381	-2.591113
1	2.349487	3.517775	-0.042960
1	1.917858	5.142154	2.173769
1	-0.256381	6.852555	2.839566
1	-0.370018	7.876994	1.373305
1	-1.674748	6.710285	1.745373
1	-4.051527	4.188550	-1.187483
1	-0.852834	1.944226	0.706835
1	-4.731527	5.124356	1.070818
1	-1.494088	2.907746	2.935587
1	-4.258699	3.917420	4.037306
1	-2.685425	4.756478	4.163272
1	-4.093489	5.584177	3.412006
1	-4.210344	-4.404048	0.240404
1	-2.219483	-5.112189	2.057534
1	-3.748054	-1.692836	-4.557475
1	-6.136322	0.220423	-1.482689
1	-5.570264	-0.118477	-3.891924
1	1.453939	-1.961365	4.758526
1	3.099513	-4.630834	1.759156
1	3.347374	-3.337170	3.884511
1	-0.178920	-4.798728	-0.191688
1	1.156531	-6.826043	-0.712404
1	2.415689	-6.360441	0.468154
1	0.778379	-6.810593	1.040119
1	1.580221	-4.610286	-1.962085
1	1.349018	-3.042999	-1.106091
1	2.776717	-4.076843	-0.734295
1	-1.963956	-1.823141	3.057161
1	-1.556514	0.467773	3.939821
1	0.165794	0.071349	4.259924
1	-0.441804	0.116391	2.568666
1	-1.717236	-3.111856	5.229838
1	-0.600873	-1.851136	5.842797
1	-2.308261	-1.441101	5.501185
1	-4.605664	-1.852388	1.299657
1	-6.821505	0.194347	0.611811
1	-7.054692	-1.582473	0.557313
1	-6.649604	-0.770036	2.103909
1	-4.334693	0.410535	2.354788
1	-3.063702	0.122816	1.111851
1	-4.462751	1.182891	0.739610
1	-1.733825	-3.730723	-2.033571
1	-0.147955	-3.149711	-3.890363
1	-1.295334	-1.902367	-4.491264
1	-0.533077	-1.715151	-2.873944
1	-1.858751	-5.031514	-4.154857
1	-3.503084	-4.867540	-3.458509
1	-3.012521	-3.857252	-4.854490
8	3.040933	1.955851	-1.169960
6	5.194130	1.948036	-0.387526
6	4.555874	0.588638	-2.221510

6	6.484995	1.460152	-0.441685
1	4.833345	2.686452	0.330627
6	5.830745	0.069117	-2.315644
1	3.716712	0.312138	-2.864175
6	6.785621	0.513266	-1.410252
1	7.250153	1.799261	0.258837
1	6.089418	-0.671854	-3.074200
7	4.264331	1.510575	-1.270844
7	8.158021	-0.041589	-1.478124
8	8.361938	-0.867331	-2.333782
8	8.948046	0.378128	-0.668963

Energy -3331.798661 (Hartree)
Thermal correction to Enthalpy 1.171997 (Hartree)
Thermal correction to Gibbs Free Energy 1.004330 (Hartree)

Ox B

Atom	X	Y	Z (Angstrom)
6	-2.342382	0.521977	-0.000004
6	-2.586309	-0.851560	-0.000009
6	-1.535921	-1.733061	-0.000005
6	-0.213202	-1.227768	-0.000000
6	0.022445	0.179682	-0.000000
7	-1.094330	1.044231	0.000004
1	-3.120250	1.286898	-0.000000
1	-3.623503	-1.194617	-0.000014
8	-0.944718	2.290353	0.000022
1	-1.692968	-2.814851	-0.000006
6	0.894351	-2.109463	0.000005
1	0.701017	-3.186320	0.000007
6	2.172451	-1.613907	0.000008
1	3.030055	-2.292350	0.000013
6	2.386319	-0.224144	0.000002
6	1.352411	0.699620	-0.000004
1	3.413021	0.155155	-0.000001
6	1.705121	2.156326	-0.000017
1	2.800008	2.269801	-0.000043
1	1.289463	2.678812	-0.872832
1	1.289507	2.678815	0.872819

Energy -515.655138 (Hartree)
Thermal correction to Enthalpy 0.177062 (Hartree)
Thermal correction to Gibbs Free Energy 0.132898 (Hartree)

TS B addition to C2

Atom	X	Y	Z (Angstrom)
6	2.158114	-2.882953	3.692400
6	1.039477	-2.147151	4.114258
6	-0.176225	-2.206198	3.400278
6	-0.214805	-3.036952	2.250282
6	0.910378	-3.767782	1.782726
6	2.099310	-3.675618	2.533803
7	-1.450064	-3.118735	1.488037
6	-1.757479	-2.244504	0.502465
7	-2.925421	-2.685536	-0.015690
6	-3.348369	-3.833339	0.642889
6	-2.420092	-4.106763	1.591267
79	-0.547099	-0.710069	-0.132377
6	0.832318	0.676564	-0.999570
6	0.514528	1.351094	-2.312772
7	-0.413001	2.470520	-2.247592
16	-2.057117	2.238592	-2.165988
6	-2.491890	3.006210	-0.543297
6	-3.578889	3.888965	-0.538361
6	-3.956089	4.509489	0.663119

6	-3.242450	4.255641	1.857240
6	-2.138276	3.374726	1.821366
6	-1.757766	2.747749	0.621710
6	-3.668714	4.925360	3.166678
6	-3.626277	-2.044268	-1.121018
6	-3.220777	-2.339875	-2.447395
6	-3.922200	-1.700116	-3.493238
6	-4.964672	-0.802706	-3.216953
6	-5.344744	-0.531409	-1.890689
6	-4.691024	-1.156977	-0.811014
6	-2.064766	-3.312666	-2.761152
6	-2.533075	-4.458272	-3.703478
6	-5.081161	-0.869825	0.655141
6	-4.291332	0.367314	1.169687
6	-1.395294	-1.373369	3.845598
6	-1.780235	-1.666514	5.323253
6	0.854618	-4.602635	0.486577
6	1.704332	-3.915183	-0.619329
6	1.971223	0.628578	-0.383328
6	2.644091	0.293538	0.857773
6	2.441879	1.108438	1.982951
6	3.164539	0.885107	3.150991
6	4.103594	-0.144059	3.203522
6	4.297012	-0.971828	2.097672
6	3.565769	-0.761021	0.932927
8	-2.247341	0.796201	-2.138818
8	-2.727234	3.056459	-3.159328
6	0.071123	3.842721	-2.279395
6	0.666646	4.324479	-0.963165
6	1.801092	3.938063	-0.284542
6	1.828754	4.699334	0.935345
6	0.710676	5.510764	0.926807
8	-0.038117	5.299407	-0.241003
6	0.178124	6.544810	1.902085
6	1.333909	-6.062995	0.720258
6	-1.098074	0.138826	3.644110
6	-6.608024	-0.650320	0.837195
6	-0.869755	-2.542831	-3.389605
1	1.717777	1.927142	1.913486
1	3.705979	-1.411662	0.063260
1	3.000786	1.522775	4.023862
1	5.017892	-1.792840	2.146869
1	4.679438	-0.311628	4.117848
1	0.117262	0.593698	-3.009095
1	1.434144	1.758349	-2.762284
1	0.823359	3.929828	-3.087382
1	-0.769455	4.500671	-2.563633
1	2.505596	3.177036	-0.652506
1	2.588603	4.667232	1.725331
1	0.850591	6.597145	2.775091
1	0.127839	7.546990	1.435956
1	-0.837295	6.279309	2.251442
1	-4.108576	4.092127	-1.487118
1	-0.883886	2.067743	0.599924
1	-4.809752	5.207221	0.680589
1	-1.562108	3.186610	2.743147
1	-4.551130	4.411003	3.593024
1	-2.855160	4.885248	3.910926
1	-3.946723	5.980262	2.993691
1	-4.270560	-4.341621	0.376509
1	-2.357252	-4.906998	2.322781
1	-3.641015	-1.909993	-4.537292
1	-6.169683	0.169583	-1.691785

1	-5.496358	-0.310196	-4.048042
1	1.103227	-1.520884	5.017930
1	2.989163	-4.236041	2.206672
1	3.092548	-2.837192	4.275398
1	-0.197090	-4.632147	0.136891
1	1.220838	-6.645110	-0.210701
1	2.397684	-6.082785	1.012488
1	0.742532	-6.550212	1.514570
1	1.620965	-4.482116	-1.563560
1	1.344579	-2.880054	-0.789089
1	2.765909	-3.880211	-0.317119
1	-2.255251	-1.640278	3.197564
1	-1.989112	0.734022	3.910780
1	-0.253477	0.453698	4.282211
1	-0.837469	0.336518	2.584999
1	-1.985916	-2.740475	5.472794
1	-0.965783	-1.366540	6.004609
1	-2.684497	-1.093669	5.592381
1	-4.782189	-1.744646	1.268003
1	-6.926291	0.296077	0.367909
1	-7.185581	-1.477872	0.390477
1	-6.846010	-0.593707	1.913404
1	-4.534299	0.559882	2.229656
1	-3.202072	0.193988	1.074746
1	-4.560001	1.258536	0.576898
1	-1.717404	-3.762594	-1.808214
1	-0.048653	-3.245387	-3.619651
1	-1.182110	-2.038591	-4.321210
1	-0.497722	-1.777009	-2.679867
1	-1.705937	-5.171455	-3.863481
1	-3.389062	-5.002422	-3.268431
1	-2.836138	-4.054484	-4.684586
8	3.258431	1.511277	-1.329143
6	4.951642	2.155987	0.035106
6	5.304819	0.372274	-1.524223
6	6.238547	2.042556	0.555075
1	4.226858	2.889307	0.388483
6	6.623458	0.224897	-0.988909
6	7.067097	1.071410	0.051687
1	6.549568	2.719387	1.352078
7	4.506311	1.361984	-0.947336
6	4.861059	-0.461120	-2.594724
6	7.482319	-0.773878	-1.503509
6	7.050640	-1.591259	-2.514335
1	7.703275	-2.366754	-2.921886
6	5.759384	-1.419064	-3.043302
1	5.440536	-2.066615	-3.865408
1	8.485677	-0.869075	-1.079861
6	3.530917	-0.377938	-3.281673
1	2.696825	-0.644637	-2.612013
1	3.324898	0.632859	-3.660196
1	3.516066	-1.072853	-4.132834
1	8.081003	0.939039	0.439013

Energy -3320.231031 (Hartree)
Thermal correction to Enthalpy 1.244784 (Hartree)
Thermal correction to Gibbs Free Energy 1.075481 (Hartree)

TS B addition to C1

Atom	X	Y	Z (Angstrom)
6	3.981885	2.022883	3.065050
6	3.078381	1.140639	3.679148

6	2.789756	-0.116742	3.106123
6	3.459268	-0.446329	1.896904
6	4.349185	0.441646	1.234031
6	4.599929	1.685070	1.849331
7	3.195303	-1.735644	1.277106
6	2.157737	-1.937218	0.433934
7	2.276730	-3.214236	0.013655
6	3.390302	-3.813082	0.590290
6	3.969652	-2.881601	1.386031
79	0.808700	-0.457326	-0.010855
6	-0.960670	1.396316	-1.392517
6	-1.534784	0.647818	-2.538442
7	-2.740252	-0.124511	-2.312357
16	-2.666482	-1.692115	-1.750461
6	-3.453192	-1.606523	-0.083809
6	-4.642586	-2.323466	0.082591
6	-5.271187	-2.327570	1.338688
6	-4.710190	-1.617457	2.424576
6	-3.511326	-0.894146	2.225146
6	-2.881192	-0.882934	0.969890
6	-5.382526	-1.643225	3.800049
6	1.341632	-3.869628	-0.890198
6	1.510207	-3.694844	-2.286589
6	0.576710	-4.333262	-3.131733
6	-0.471903	-5.098633	-2.597451
6	-0.614143	-5.249226	-1.207210
6	0.296489	-4.641621	-0.319510
6	2.637599	-2.819177	-2.872741
6	3.459563	-3.585195	-3.947586
6	0.130180	-4.753276	1.211176
6	-0.926161	-3.716289	1.689322
6	1.774249	-1.070330	3.769718
6	2.188939	-1.409773	5.229708
6	4.983489	0.089817	-0.127926
6	4.340359	0.963790	-1.241991
6	-0.335855	1.352514	-0.271218
6	-0.217703	2.278694	0.862817
6	-1.359978	2.552297	1.631644
6	-1.278429	3.380248	2.747032
6	-0.062558	3.958637	3.107804
6	1.073306	3.695397	2.344498
6	1.004974	2.848349	1.240958
8	-1.246848	-1.991350	-1.632090
8	-3.546946	-2.524124	-2.546429

6	-4.066237	0.453982	-2.497543
6	-4.552101	1.249565	-1.295917
6	-4.023347	2.373105	-0.705443
6	-4.850205	2.664298	0.434212
6	-5.837434	1.698248	0.468706
8	-5.678642	0.794811	-0.595008
6	-7.014438	1.459471	1.396799
6	6.527538	0.269205	-0.110057
6	0.353194	-0.439959	3.735610
6	-0.269322	-6.185003	1.663426
6	2.033663	-1.517411	-3.470293
1	-2.316284	2.107229	1.339223
1	1.906132	2.624366	0.656838
1	-2.176812	3.576428	3.339647
1	2.035061	4.137981	2.619899
1	0.001031	4.607673	3.985181
1	-0.748839	-0.036875	-2.900374
1	-1.746765	1.351120	-3.357881
1	-4.029696	1.096978	-3.397681
1	-4.774179	-0.364626	-2.718081
1	-3.130908	2.885914	-1.097702
1	-4.740176	3.492645	1.144398
1	-6.994934	2.211535	2.203698
1	-7.975755	1.548886	0.856134
1	-6.969851	0.451537	1.849866
1	-5.059965	-2.871874	-0.782152
1	-1.941553	-0.317328	0.821446
1	-6.210264	-2.886869	1.484346
1	-3.070382	-0.327174	3.062969
1	-4.971233	-2.467332	4.414175
1	-5.205473	-0.696765	4.339924
1	-6.470019	-1.804090	3.700852
1	3.658478	-4.844985	0.383278
1	4.855725	-2.925595	2.012351
1	0.674814	-4.224772	-4.223362
1	-1.444575	-5.849170	-0.803957
1	-1.192266	-5.586297	-3.275200
1	2.587799	1.427529	4.622720
1	5.293447	2.394017	1.370516
1	4.206189	2.992012	3.540673
1	4.756489	-0.971417	-0.357705
1	6.950676	-0.054905	-1.076568
1	6.795782	1.327982	0.046101
1	6.985550	-0.331537	0.694530

1	4.780344	0.708012	-2.222132
1	3.247078	0.785946	-1.282016
1	4.520105	2.034416	-1.039628
1	1.743113	-2.011992	3.182768
1	-0.373939	-1.131993	4.196468
1	0.338493	0.516091	4.288344
1	0.045741	-0.245444	2.687971
1	3.198801	-1.854092	5.260515
1	2.185256	-0.501025	5.855716
1	1.473214	-2.130502	5.661560
1	1.098934	-4.496829	1.686558
1	-1.288761	-6.433240	1.322626
1	0.429991	-6.938298	1.261635
1	-0.254647	-6.241432	2.765567
1	-1.029084	-3.757054	2.788167
1	-0.622364	-2.692788	1.392515
1	-1.906813	-3.939913	1.233832
1	3.321578	-2.527906	-2.048088
1	2.835583	-0.888674	-3.897136
1	1.305540	-1.760559	-4.264573
1	1.515455	-0.946228	-2.674257
1	4.298121	-2.956161	-4.293215
1	3.868724	-4.524367	-3.537167
1	2.828921	-3.828588	-4.819582
8	-1.453622	3.168100	-1.979830
6	-1.538143	4.926666	-0.551322
6	0.500448	4.465138	-1.708125
6	-0.973986	5.982977	0.157256
1	-2.575283	4.618949	-0.424333
6	1.122008	5.490906	-0.927693
6	0.363394	6.240646	-0.002857
1	-1.599565	6.549081	0.848919
7	-0.838788	4.182890	-1.419039
1	0.860166	7.028995	0.569135
6	2.500667	5.761229	-1.088822
6	1.243265	3.799750	-2.728407
6	2.590507	4.115420	-2.827809
1	3.172287	3.613017	-3.606710
6	3.232938	5.061511	-2.010979
1	4.299141	5.258937	-2.144550
1	2.953791	6.541969	-0.471780
6	0.687808	2.840908	-3.736387
1	0.609900	1.824661	-3.318768
1	-0.307392	3.133810	-4.092303

1	1.366495	2.785223	-4.599233

Energy -3320.227662 (Hartree)

Thermal correction to Enthalpy 1.245397 (Hartree)

Thermal correction to Gibbs Free Energy 1.077814 (Hartree)
